# MicroRNA profiling of rats with ochratoxin A nephrotoxicity

**DOI:** 10.1186/1471-2164-15-333

**Published:** 2014-05-05

**Authors:** Qiu Dai, Jue Zhao, Xiaozhe Qi, Wentao Xu, Xiaoyun He, Mingzhang Guo, Harsh Dweep, Wen-Hsing Cheng, Yunbo Luo, Kai Xia, Norbert Gretz, Kunlun Huang

**Affiliations:** Laboratory of food safety and molecular biology, College of Food Science and Nutritional Engineering, China Agricultural University, 302 box, No.17, Qinghua East Rd, Beijing, Haidian District, 100083 P R China; Medical Faculty Mannheim, Medical Research Center, University of Heidelberg, Mannheim, D-68167 Germany; Department of Food Science, Nutrition and Health Promotion, Mississippi State University, Mississippi State, MS 39762 USA

**Keywords:** High throughput sequencing, Ochratoxin A, miRNA biogenesis, miRNA expression, Nephrotoxicity

## Abstract

**Background:**

Nephrotoxicity is the most prominent one among the various toxicities of ochratoxin A (OTA). MicroRNAs (miRNAs) are small non-coding RNAs that have an impact on a wide range of biological processes by regulating gene expression at post-transcriptional level or protein systhesis level. The objective of this study is to analyze miRNA profiling in the kidneys of rats gavaged with OTA.

**Results:**

To profile miRNAs in the kidneys of rats with OTA nephrotoxicity, high-throughput sequencing and bioinformatics approaches were applied to analyze the miRNAs in the kidney of rats following OTA treatment. A total of 409 known miRNAs and 8 novel miRNAs were identified in the kidney and the levels of the novel miRNAs were varied in response to different doses of OTA. Expression of miR-129, miR-130a, miR-130b, miR-141, miR-218b and miR-3588 were uniquely suppressed in mid dose but then elevated in high dose, with opposite expression to their target genes. The expression pattern was closely related with the “MAPK signaling pathway”. *Dicer1* and *Drosha* were significantly suppressed, indicating an impairment of miRNA biogenesis in response to OTA.

**Conclusions:**

The abrogation of miRNA maturation process suggests a new target of OTA toxicity. Moreover, the identification of the differentially expressed miRNAs provides us a molecular insight into the nephrtoxicity of OTA.

**Electronic supplementary material:**

The online version of this article (doi:10.1186/1471-2164-15-333) contains supplementary material, which is available to authorized users.

## Background

Ochratoxin A (OTA) is an ubiquitous mycotoxin produced by several species of *Aspergillus* and *Penicillium*[1]. Humans are chronically and continuously exposed to OTA because of its widespread existence in cereals (barley, oats, rye, corn and wheat), beans, dried fruits, tea, coffee, cocoa, wine, beer, herbs, poultry, fish, pork, eggs, cheese and milk [2]. When consuming in excess, OTA is known to exert a diverse range of toxicological effects including nephrotoxicity, hepatotoxicity, teratogenicity, mutagenicity, neurotoxicity and immunotoxicity [2, 3].

It has been shown that mitogen-activated protein kinase (MAPK) signaling pathway plays an important role in mediating OTA toxicity *in vivo* and *in vitro*. MAPK activation is proved to induce renal carcinoma in rats chronically fed with OTA [4]. Although the link between MAPK signaling pathway and OTA toxicity has been studied, the key miRNAs involved in the nephrotoxicity are unknown. Understanding the linkage between miRNAs and OTA nephrotoxicity is crucial to fill the knowledge gap.

miRNAs are short non-coding RNAs that typically regulate gene expression at post-transcriptional level by binding to partially complementary sites of their target mRNAs. A miRNA can regulate hundreds of mRNAs and impact on crucial biological processes, including cell growth, apoptosis, development and differentiation [5]. Moreover, various miRNAs have been established to play important roles in the development of renal carcinoma [6–8].

The mechanism of miRNA biogenesis has become clear. The majority of primary miRNAs (pri-miRNAs) transcripts are generated by RNA polymerase II. Pri-miRNAs are then cleaved into precursor miRNAs (pre-miRNAs) by microprocessor and subsequently exported to the cytoplasm by exportin 5 (Exp 5). The microprocessors contain *Drosha*, a nuclear protein, and its cofactor, the DiGeorge syndrome critical region gene 8 protein (*DGCR8*), and play a pivotal role in recognizing and trimming pre-miRNAs. Pre-miRNAs are further cleaved into single-stranded mature miRNAs by one of the RNase III family enzymes – *Dicer*. Mature miRNAs are then transferred to the RNA-induced silencing complex (RISC) to mediate the degradation and/or translational inhibition of their target RNA sequences [9]. Furthermore, the stability of miRNA biogenesis is crucial for maintaining the cellular homeostasis. Impaired miRNA processing has been found in various tumors [10–13], suggesting a strong connection between miRNA processing and cancer.

The objective of this study is to analyze miRNA profiling in the kidneys of rats gavaged with OTA. The results showed that miRNA biogenesis were impaired and that the expression profiles of several miRNAs were altered in association with OTA nephrotoxicity.

## Results

### Body and organ weights

**Figure 1:**
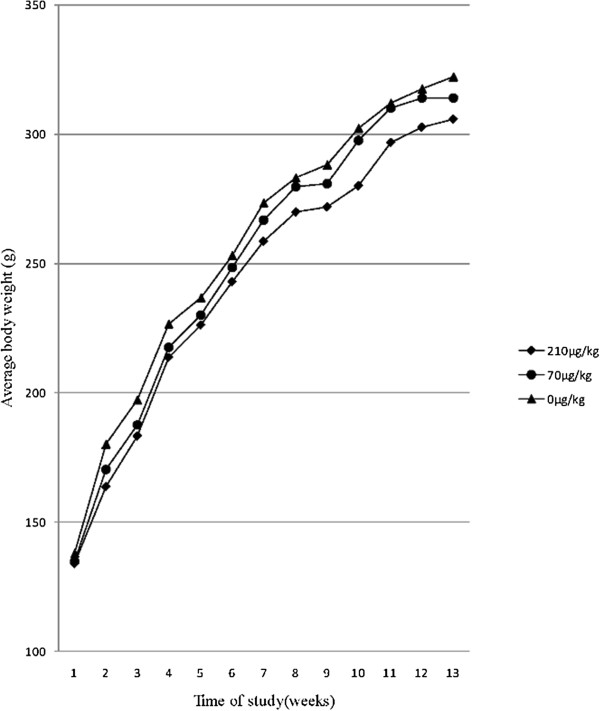
**Body weight of the male rats after OTA administration at the indicated doses.**

Mean body weights were slightly reduced at 2, 3, 5, 7 and 10 (*p* < 0.05, data not shown) weeks after administration of OTA at 210 μg/kg (Figure 1). Kidney weights were significently (*p* < 0.05) reduced 4–26 weeks after OTA administration (Table 1). Liver weights were increased after OTA administration (70 μg/kg) at week 2 and decreased (210 vs. 70 μg/kg) at week 26. OTA administration did not impact on spleen and testis weights.

**Table 1:** **Organ weights after administration of OTA (0, 70, 210 μg/kg body weight) for 2, 4, 13 and 26 weeks**

Time (weeks)	Dose (μg/kg)	Liver (g)	Kidney (g)	Spleen (g)	Testicle (g)
2	0	5.59 ± 0.44	1.40 ± 0.12	0.46 ± 0.02	2.28 ± 0.09
	70	6.35 ± 0.43 a	1.56 ± 0.09 a	0.44 ± 0.04	2.37 ± 0.20
	210	5.83 ± 0.75	1.40 ± 0.11 b	0.43 ± 0.06	2.49 ± 0.35
4	0	6.19 ± 0.80	1.57 ± 0.14	0.45 ± 0.04	2.55 ± 0.12
	70	6.18 ± 1.33	1.57 ± 0.21	0.46 ± 0.07	2.56 ± 0.28
	210	5.98 ± 0.79	1.43 ± 0.12	0.43 ± 0.04	2.63 ± 0.07
13	0	8.06 ± 1.43	2.03 ± 0.10	0.56 ± 0.79	2.93 ± 0.06
	70	6.84 ± 0.68	1.83 ± 0.12 a	0.54 ± 0.05	3.00 ± 0.20
	210	6.86 ± 0.70	1.51 ± 0.74 ab	0.50 ± 0.46	2.83 ± 0.17
26	0	7.48 ± 0.72	2.02 ± 0.24	0.54 ± 0.06	3.13 ± 0.25
	70	8.11 ± 0.96	2.04 ± 0.14	0.58 ± 0.06	3.20 ± 0.11
	210	6.72 ± 0.68 b	1.67 ± 0.19 ab	0.54 ± 0.07	3.10 ± 0.16

a: *p* < 0.05, compared to 0 μg/kg b.w. group; b: *p* < 0.05, compared to 70 μg/kg b.w. group.

### Serum clinical chemistry and histopathology

The blood clinical chemistry was not affected by OTA doses or administration time. Blood urea nitrogen and creatinine (BUN), common indexes of kidney damage, did not reflect the nephrotoxicity induced by OTA. Interestingly, aspartate transaminase (AST) level was higher in the high dose than that in the mid dose group at four weeks, but was lower in the high dose than that in the control group at 13 weeks. The same is true for high-density lipoprotein (HDL) and lactate dehydrogenase (LDH) (Table 2).

**Table 2:** **Serum clinical chemistry after administration of OTA (0, 70, 210 μg/kg body weight) for 2, 4 or 13 weeks**

Time (weeks)	Dose (μg/kg)	ALT (U/L)	AST (U/L)	ALB (g/L)	ALP(U/L)	GLU (mmol/L)	BUN (mmol/L)	CREA (umol/L)	HDL(mmol/L)	LDL(mmol/L)	LDH (U/L)
2	0	45.17 ± 5.04	283 ± 63	52.4 ± 0.9	310 ± 31	3.37 ± 1.61	6.2 ± 0.5	37 ± 16	2.52 ± 0.31	0.59 ± 0.17	3484 ± 696
	70	45.2 ± 6.30	241 ± 53	51.7 ± 2.9	315 ± 33	4.71 ± 1.07	6.2 ± 0.4	29 ± 2	2.91 ± 0.46	0.96 ± 0.42	3062 ± 632
	210	42.67 ± 6.44	208 ± 448	50.1 ± 2.1	305 ± 41	4.3 ± 1.03	6.2 ± 0.7	31 ± 10	2.37 ± 0.23	0.72 ± 0.18	2644 ± 506a
4	0	50.00 ± 3.08	240 ± 25	75.0 ± 3.5	288 ± 36	4.99 ± 1.1	5.7 ± 0.7	28 ± 3	2.96 ± 0.34	0.64 ± 0.2	3370 ± 338
	70	52.83 ± 6.79	225 ± 61	76.5 ± 4.2	367 ± 78a	5.76 ± 1.04	6.7 ± 0.8	32 ± 7	2.89 ± 0.47	0.82 ± 0.14	2965 ± 769
	210	52.33 ± 5.39	305 ± 61b	72.4 ± 2.7	275 ± 14b	3.71 ± 1.12b	6.1 ± 0.9	34 ± 4	3.42 ± 0.18b	0.83 ± 0.22	3842 ± 727b
13	0	56.50 ± 7.61	167 ± 17	49.2 ± 1.5	152 ± 17	7.91 ± 1.02	8.5 ± 0.5	43 ± 2	3.42 ± 0.55	0.77 ± 0.16	2043 ± 237
	70	52.17 ± 2.99	152 ± 7	51.1 ± 2.4	153 ± 10	7.34 ± 0.63	8.7 ± 0.5	43 ± 4	2.93 ± 0.25	0.69 ± 0.09	1974 ± 153
	210	52.33 ± 4.23	140 ± 13a	51.2 ± 2.4a	157 ± 11	7.76 ± 0.99	8.5 ± 0.5	43 ± 9	2.69 ± 0.13a	0.74 ± 0.15	1763 ± 208a

Data are presented as Mean ± SD of six rats per group. a: *p* < 0.05 when compared to 0 μg/kg b.w. group; b: *p* < 0.05 when compared to 70 μg/kg body weight group.

**Figure 2:**
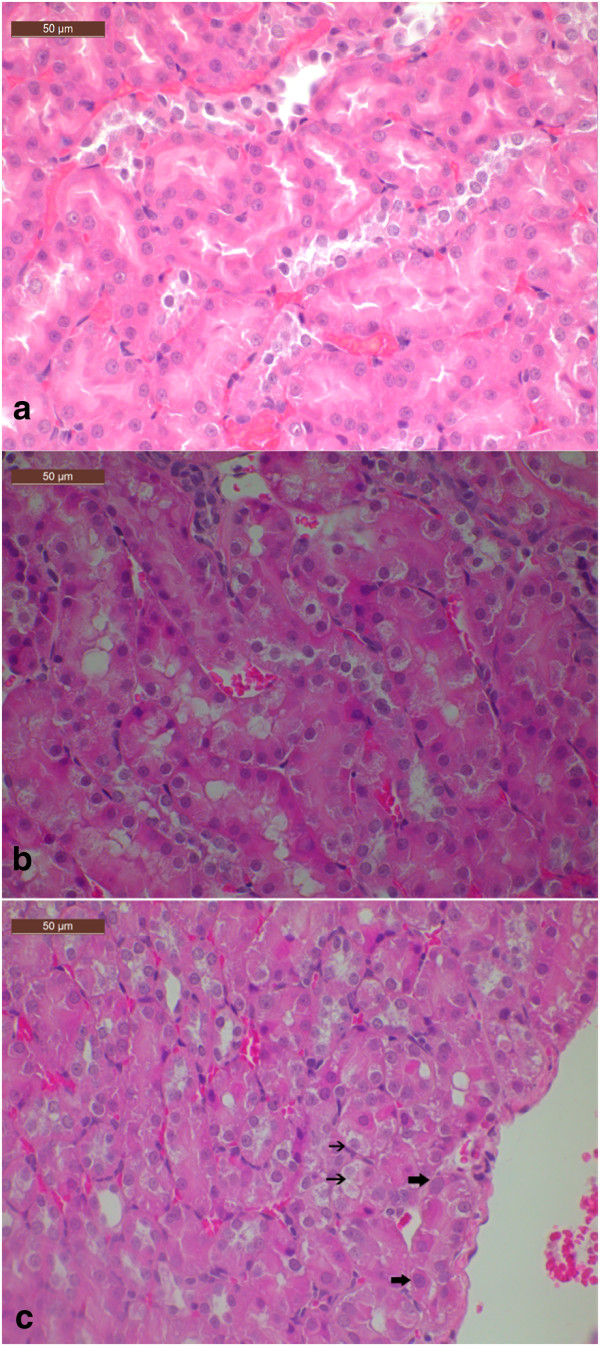
**H & E stained kidney sections of a control rat (a) and of rats treated with 70 (b) and 210 (c) μg/kg b.w.** OTA for 13 weeks. At 70 and 210 μg/kg b.w., OTA induced severe cytoplasmic vacuolization (thin arrow) and karyomegaly (thick arrow) in proximal tubular epithelial cells in outer stripe of outer medulla (OSOM).

In the rats administrated of OTA for 13 and 26 weeks, cytoplasmic vacuolization was observed in the outer stripe of outer medulla (OSOM) in both 70 μg/kg and 210 μg/kg groups. Karyomegaly (enlargement of the nuclei in the tubular epithelium) was prominent in tubular epithelium and the severity was OTA dose- and time-dependent. The structure of tubular epithelium in the rats gavaged with OTA at a dose of 210 μg/kg for 26 weeks was severely damaged. Histopathology of the kidneys in the rats treated with OTA for 2 and 4 weeks were generally indistinguishable. Renal lesions were not found in the vehicle control rats (Figure 2, Additional file 1: Figure S1).

In summary, we found distinguishable (compared to 4 weeks) but not severe (compared to 26 weeks) histopathological damage in groups of 13 weeks. Because thirteen-week was a turning point where OTA started to show its nephrotoxicity, this time point was selected for the study of OTA nephrotoxicity and miRNA profiling and analysis in the kidneys.

### miRNA profiling

**Figure 3:**
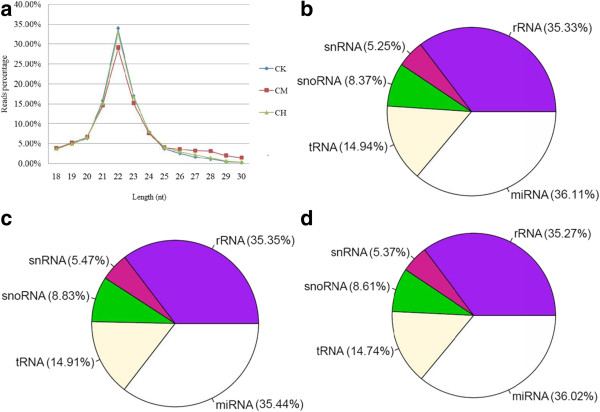
**RNA length and types distribution in CK, CM and CH. (a)** Length distribution of small RNA reads percentage in CK, CM and CH. The distribution of RNA types in CK **(b)**, CM **(c)** and CH **(d)** were summarized. CK, group of rats gavaging 0 μg/kg OTA for 13 weeks; CM, group of rats gavaging 70 μg/kg OTA for 13 weeks; CH, group of rats gavaging 210 μg/kg OTA for 13 weeks.

We used A, B, C and D to denote the groups of 2, 4, 13, and 26 weeks, respectively. K (control goup), M (mid-dose group) and H (high-dose group) represent 0, 70,and 210 μg/kg groups, respectively. Total RNA was pooled from rats in each group and thus three datasets with a total of 24361780, 21752336 and 24515287 reads were obtained from CK, CM and CH, respectively. Clean reads (about 90% of total reads) were retained for further analysis after removing the adapters, low quality reads and small sequences (sizes < 18 nt) (Table 3). Analyses of small RNA length distribution (Figure 3a) indicated that the three samples peaked at the size of 22 nt, suggesting the homogeneity of the samples. CK and CH were almost superimposed. Next, clean reads were compared with the Rfam database (ftp://selab.janelia.org/pub/Rfam) to match with the known rRNA, snRNA, snoRNA and tRNAsequences (Figure 3b – d). After these non-coding RNAs (ncRNAs) were removed, the remaining clean reads were further compared with the pre-miRNAs database in miRbase. The matched reads were used to identify mature miRNAs, and the number of their reads were accounted. The reads that did not yield a match were used to predict novel miRNAs using MIREAP. The numbers of miRNA reads were normalized by Tags per million (TPM) values (TPM = (miRNA total reads/total clean reads) × 10^6^) to express miRNAs in CK, CM, CH comaprable in one table.

**Table 3:** **Parameters of small RNA sequences from CK, CM and CH**

Dose of gavage	Reads types	Reads number	Percentage
CK	Total reads number	24361780	100%
	Low quality	18020	0.07%
	Adaptor3 null	1258473	5.17%
	Insert null	0	0%
	5′ adaptor contaminants	7165	0.03%
	small.txt, size < 18 nt	1107061	4.54%
	polyA	4660	0.02%
	High Quality (size ≥ 18 nt)	21966401	90.17%
CM	Total reads number	21752336	100%
	Low quality	15999	0.07%
	Adaptor3 null	1838914	8.45%
	Insert null	0	0%
	5′ adaptor contaminants	4953	0.02%
	small.txt, size < 18 nt	756277	3.48%
	polyA	4045	0.02%
	High Quality (size ≥ 18 nt)	19132148	87.95%
CH	Total reads number	24515287	100%
	Low quality	20684	0.08%
	Adaptor3 null	1179046	4.81%
	Insert null	0	0%
	5′ adaptor contaminants	5293	0.02%
	small.txt, size < 18 nt	865014	3.53%
	polyA	3918	0.02%
	High Quality (size ≥ 18 nt)	22441332	91.54%

### Novel miRNA analysis

**Figure 4:**
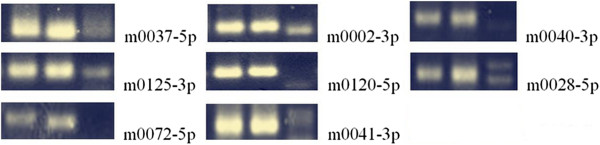
**The validation of 8 identified novel miRNAs in the kidney by PCR.** The two left lanes indicated miRNAs and the right depicts the negative control.

A number of criterions were used for evaluating whether a small RNA was a genuine miRNA, such as formation of a stable hairpin structure, lower minimal free energies for hairpin structure of its precursors, and detection of miRNA*s [14]. Given these analyses, 8 novel miRNAs were identified and examined by PCR (Additional file 1: Table S1, Additional file 1: Figure S2, Figure 4).

### Impairment of miRNA processing

**Figure 5:**
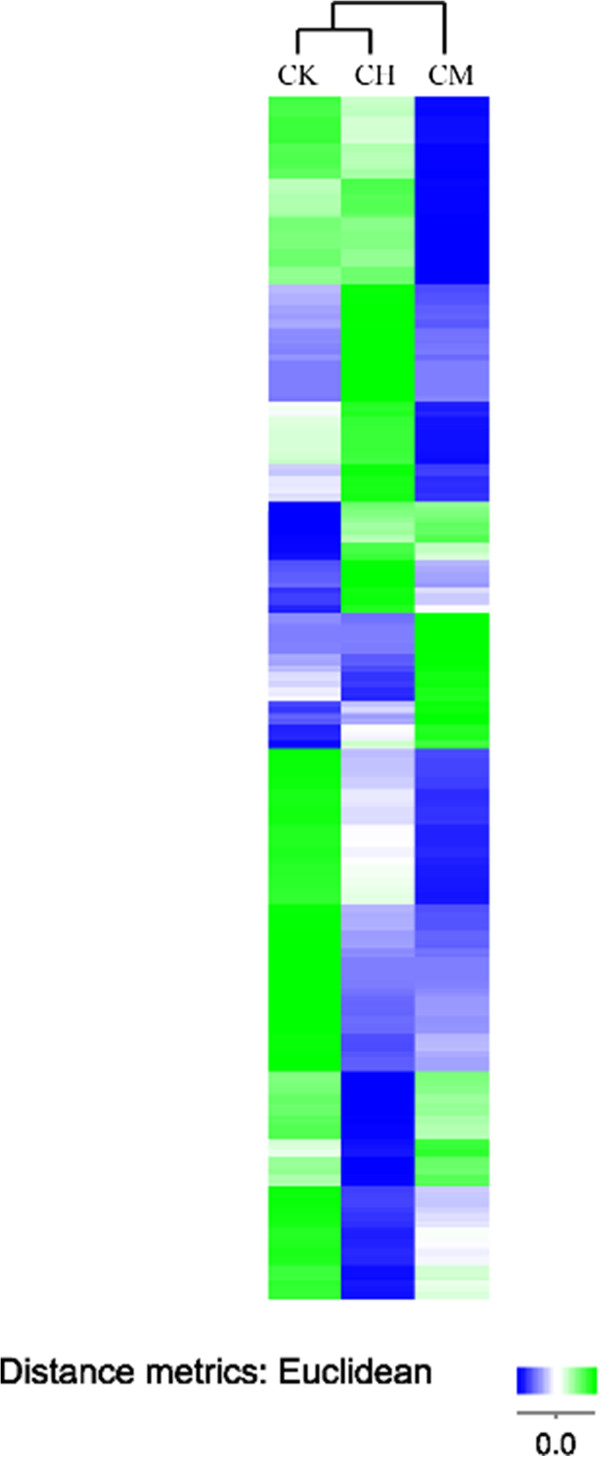
**Hierachical clustering for the differentially expressed miRNAs.** The color scale illustrates the relative expression level of the identified miRNAs across the three samples. The blue denotes expression < 0 and the green denotes expression > 0. The euclidean distance measure was used to calculate the distance. The CK, CM and CH groups, rats gavaging 0, 70, and 210 μg/kg OTA, respectively, for 13 weeks.

A total of 409 known miRNAs were found in CK, CM, and CH kidneys, 394 of which were differentially (*p* <0.05) expressed among the three groups (Additional file 1: Table S2). We further mapped the distribution of the miRNA length (Additional file 1: Figure S3), which was consistent with the pattern shown in Figure 3a. Total miRNAs in CM was slightly lower than those in CH and CK (Figure 3b – d). Results from hierachical clustering analysis depicted that miRNA expression was similar between CK and CH, and they were different from CM kidneys (Figure 5). Therefore, differences of the expression pattern in the three groups are likely due to the impairment of miRNA processing after OTA nephrotoxicity.

**Figure 6:**
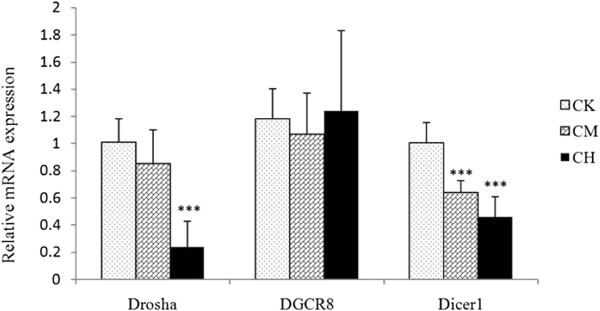
**qRT-PCR analyses of** ***Drosha*** **,** ***DRCG8*** **and** ***Dicer1*** **mRNA levels in the kidneys of the rats in CK, CM and CH.** Expression levels were normalized using β-actin. Values are mean ± s.d. (n = 6). ***, *p* < 0.01.

To further understand the nature of the defective miRNA processing after OTA toxicity, we determined the expression of key regulators of miRNA processing: *Drosha*, *Dicer1* and *DRCG8*. As shown in Figure 6, mRNA levels of *Drosha* and *Dicer1*, but not *DRCG8*, were significantly reduced after OTA administrtaion.

### Analysis of known miRNA expression pattern

**Figure 7:**
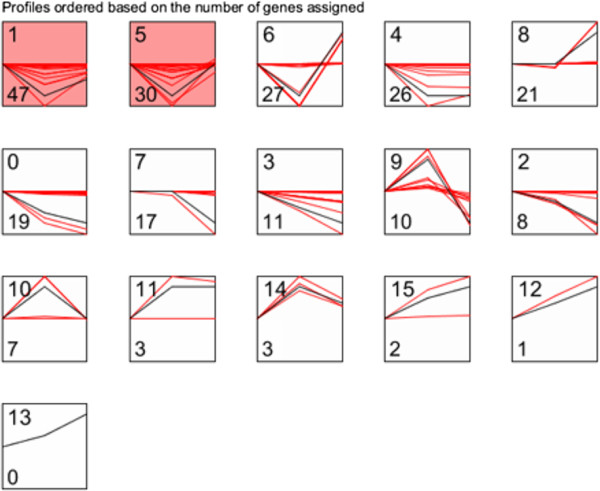
**The expression pattern of miRNAs in response to various doses of OTA.** Two significant profiles (profile 1 and profile 5) out of 16 profiles were detected as significant using STEM analysis. Red lines represent individual miRNA whose expression correlates with OTA doses. Black curve represents the model of the profile to which the miRNA expression pattern are similar. The numbers in the upper and lower left corners are the identifier (ID) of the profile and the number of miRNAs in a that profile.

Expression pattern of all known miRNAs was examined by using STEM software. Two significant profiles (profile 1 and 5) containing 77 miRNAs were identified (Figure 7). Expression of these 77 miRNAs in both profiles were repressed in CM and reversed in CH kidneys. Among these 77 miRNAs, those with ≥ 2-fold down-regulation in CM compared to CK (miR-129, miR-130a, miR-130b, miR-141, miR-218b and miR-3588) were selected for bioinformatic analysis.

The information on the putative target genes of these 6 miRNAs were collected using the comparative platform of miRWalk [15] by considering six different algorithms, including miRanda, miRDB, miRWalk, PITA, TargetScan and RNAhybrid. Demanding a target gene to be predicted by different miRNA-target prediction algorithms can be helpful in minimizing the number of putative and maybe false positive targets [16–18]. Therefore, the targets predicted with at least 2 different programs were considered for further analysis. Both separative and collective KEGG/GO enrichment analysis were performed. In separative analysis, the predicted target genes were subjected to KEGG/GO enrichment analysis individually, following by a multiple testing method (Benjamini and holm, BH) to select for significantly (*p* < 0.05) over-represented KEGG pathways and GOBPs (Table 4A and B, full tables are given in Additional file 1: Table S3). In collective analysis, the identified targets were merged into one list, and non-redundant genes were removed followed to carry out KEGG/GO enrichment analysis (Additional file 1: Table S4 A and B). “phosphatidylinositol signaling system”, “pancreatic cancer” and “MAPK signaling pathway” were greatly enriched as evidenced by both separative and collective analyses. Notably, regulation of the pathways and GOBPs were strongly associated with miR-129, miR-130a and miR-130b. No relevant pathways and GOBPs were found to be regulated by miR-141 and miR-3588, which might be explained by the less putative targets prediction of the two miRNAs.

**Table 4:** **The brief table of 10 most significant KEGG pathways (A) and GOBPs (B) for the 6 miRNA targets in separative analysis**

Pathways	Targeted by different miRNAs	rno-miR-129	rno-miR-130a	rno-miR-130b	rno-miR-141	rno-miR-218b	rno-miR-3588
**A**							
rno04070 Phosphatidylinositol signaling system	4	1	1	1	0	1	0
rno05212 Pancreatic cancer	3	1	1	1	0	0	0
rno04971 Gastric acid secretion	3	1	1	1	0	0	0
rno04010 MAPK signaling pathway	3	1	1	1	0	0	0
rno04622 RIG-I-like receptor signaling pathway	3	1	1	1	0	0	0
rno00380 Tryptophan metabolism	3	1	1	1	0	0	0
rno04730 Long-term depression	3	1	1	1	0	0	0
rno00410 beta-Alanine metabolism	3	1	1	1	0	0	0
rno04110 Cell cycle	3	1	1	1	0	0	0
rno04114 Oocyte meiosis	3	1	1	1	0	0	0
…							
rno05410 Hypertrophic cardiomyopathy (HCM)	1	1	0	0	0	0	0
**Total pathways targeted by each miRNA**	47	48	47	0	1	0	
**B**							
GO:0016043 ~ cellular component organization and biogenesis	5	1	1	1	1	0	1
GO:0050789 ~ regulation of biological process	5	1	1	1	1	0	1
GO:0008283 ~ cell proliferation	5	1	1	1	1	0	1
GO:0065007 ~ biological regulation	5	1	1	1	1	0	1
GO:0048519 ~ negative regulation of biological process	5	1	1	1	1	0	1
GO:0006796 ~ phosphate metabolic process	4	1	1	1	0	0	1
GO:0030154 ~ cell differentiation	4	1	1	1	0	0	1
GO:0008104 ~ protein localization	4	1	1	1	0	0	1
GO:0051336 ~ regulation of hydrolase activity	4	1	1	1	0	0	1
GO:0051179 ~ localization	4	1	1	1	0	0	1
…							
GO:0043434 ~ response to peptide hormone stimulus	1	1	0	0	0	0	0
Different pathways predicted by each miRNA	162	157	156	5	0	27	

### Differently expressed miRNAs

We further analyzed the differentially expressed miRNAs in CH group (Table 5A). There were 10 up-regulated and 23 down-regulated miRNAs with ≥ 3-fold difference (*p* <0.05).

**Table 5:** **Deferentially expressed miRNAs in CH (A) or CM (B)**

	A			B		
	Mature miRNA	Fold change	P-vaule	Mature miRNA	Fold change	P-value
Up-regulated miRNAs	rno-miR-3065-3p	21.0	<0.0001	rno-miR-3556a	85.0	<0.0001
	rno-miR-653	17.0	<0.0001	rno-miR-653	20.3	<0.0001
	rno-miR-3596b	15.4	<0.0001	rno-miR-3596b	10.8	<0.0001
	rno-miR-3556b	10.1	<0.0001	rno-miR-30c	4.8	<0.0001
	rno-miR-504	8.4	<0.0001	rno-miR-145	4.2	<0.0001
	rno-miR-3596a	8.3	<0.0001	rno-miR-19a	4.1	<0.0001
	rno-miR-3596c	8.3	<0.0001	rno-miR-19b	4.1	<0.0001
	rno-miR-30c	4.3	<0.0001	rno-miR-3590-5p	4.0	<0.0001
	rno-miR-338	4.2	0.000126	rno-miR-3473	3.0	<0.0001
	rno-miR-200c	3.7	0.006459	rno-miR-338	3.0	0.001122
Down-regulated miRNAs	rno-miR-133a	−28.6	<0.0001	rno-miR-218b	−98.6	<0.0001
	rno-miR-133b	−28.6	<0.0001	rno-miR-130a	−63.9	<0.0001
	rno-miR-128	−13.7	<0.0001	rno-miR-130b	−63.9	<0.0001
	rno-miR-182	−13.3	<0.0001	rno-miR-3588	−31.3	<0.0001
	rno-miR-190*	−10.6	<0.0001	rno-miR-129	−24.2	<0.0001
	rno-miR-3587	−9.8	<0.0001	rno-miR-138	−11.5	<0.0001
	rno-miR-135a	−9.2	<0.0001	rno-miR-182	−11.3	0.000154
	rno-miR-135b	−9.2	<0.0001	rno-miR-133a	−6.7	<0.0001
	rno-miR-186	−8.7	<0.0001	rno-miR-133b	−6.7	<0.0001
	rno-miR-2964	−6.6	0.003196	rno-miR-136*	−5.7	0.022282
	rno-miR-96	−6.6	0.003196	rno-miR-141	−5.6	<0.0001
	rno-miR-378b	−5.7	<0.0001	rno-miR-3596c	−5.1	<0.0001
	rno-miR-378	−5.7	<0.0001	rno-miR-3596d	−5.1	<0.0001
	rno-miR-328b-3p	−4.4	0.009016	rno-miR-9b-5p	−4.8	0.005151
	rno-miR-3596d	−4.2	<0.0001	rno-miR-216a	−3.5	0.007073
	rno-miR-154*	−4.1	0.01503			
	rno-miR-466b-1-3p	−4.1	0.047095			
	rno-miR-151-3p	−3.8	0.000013			
	rno-miR-29a-5p	−3.7	0.024944			
	rno-miR-802-5p	−3.7	0.0001			
	rno-miR-1843-5p	−3.4	0.0001			
	rno-miR-138	−3.0	0.0001			
	rno-miR-802*	−3.0	0.000037			

“*”represents that a mature microRNA is expressed from both the 5'-arm and the 3'-arm.

**Figure 8:**
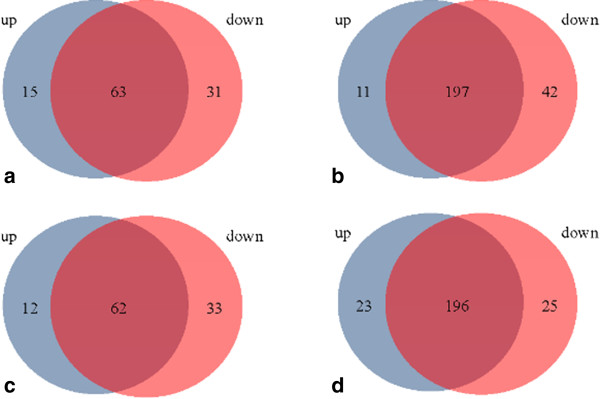
**Venn diagrams for the overlapping KEGG pathways (a and c) or GOBP analyses (b and d) in up- or down-regualted miRNAs in CH (a and b) or CM (c and d) groups.**

Putative target mRNAs of 31 miRNAs were predicted as previously mentioned (rno-mir-378b and mir-1843-5p are not found in the selected databases). Thereafter, separative and collective KEGG/GOBPs analyses were accomplished as a result of meta-analysis predictions (BH < 0.05). In separative analysis, seventy-eight pathways were enriched in the “10 up-regulated” and ninety-three were enriched in the “21 down-regulated” miRNAs, while 208 and 230 GOBPs were enriched in the “10 up-regulated” and “21 down-regulated” miRNAs, respectively (Additional file 1: Tables S5 A, B, C and D). Venn diagrams were constructed to determine the common pathways (Figures 8a and b). Interestingly, the majority of the 63 pathways were overlapped between the “10 up-regulated” and “21 down-regulated” miRNAs. Similarly, a total of 197 GOBPs were commonly identified between the two groups of miRNAs.

Twenty-five miRNAs that were deregulated in the CM group (10 were up-regulated and 15 were down-regulated, 3-fold difference, *p* < 0.05) were also subjected to KEGG/GO enrichment analysis (Additional file 1: Table S6 B). miR-3473 was not found in the selected database. In separative analysis, 73 and 95 pathways and 219 and 220 GOBPs were over-represented in the “9 up-regulated” and “15 down-regulated” miRNAs, respectively. Moreover, extensive overlaps were noted between pathways and GOBPs (Figures 8c and d). The target genes involved in these pathways or GOBPs were given in Additional file 1: Tables S6 A-D.

### PCR validation for Nrf2, Keap1 and miRNAs

**Figure 9:**
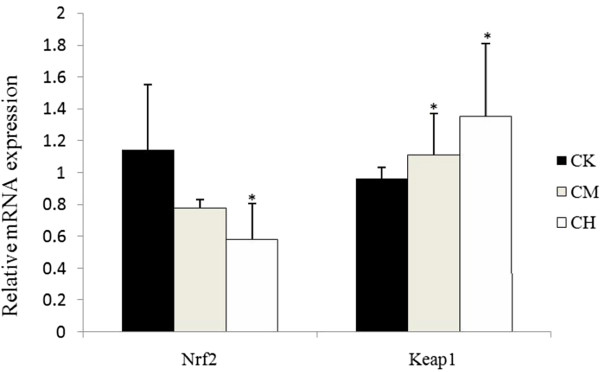
**qRT-PCR analyses of** ***Nrf2*** **and** ***Keap1*** **mRNA levels in the kidneys of the rats in CK, CM and CH groups.** Expression levels were normalized by β-actin. Values are mean ± s.d. *, *p* < 0.05.

We tested the gene expression of *Nrf2* and its negative regulator, *Keap1. Nrf2* mRNA level in the kidney was significantly decreased (*p* < 0.05) after OTA treatment (210 μg/kg), and the expression of *Keap1* was significantly increased by OTA treatment (Figure 9).

**Figure 10:**
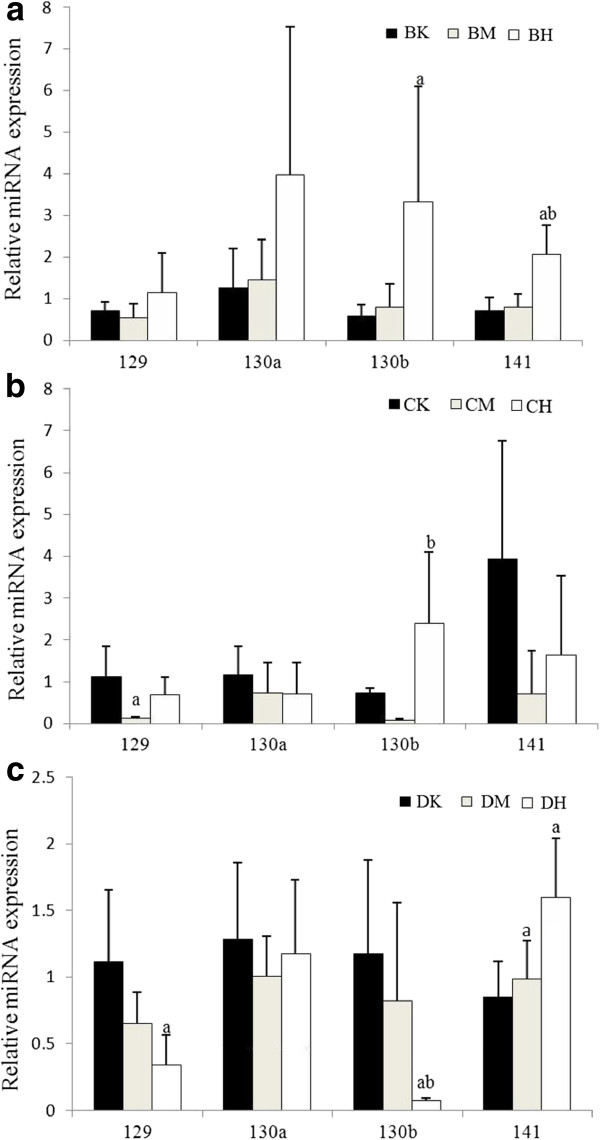
**qRT-PCR analysis of miR-129, miR-130a, miR-130b and miR-141 expression in the kidneys of the rats in 4 weeks (BK, BM and BHgroups, Figure** 10 **a), 13 weeks (CK, CM and CH groups, Figure** 10 **b) and 26 weeks (DK, DM and DH groups, Figure** 10 **c).** Expression levels were normalized by 5S. Values were mean ± s.d. a: *p* < 0.05 when compared to 0 μg/kg b.w. group; b: *p* < 0.05 when compared to 70 μg/kg body weight group.

Among the six miRNAs that selected by STEM analysis, 4 miRNAs (most of the pathways were enriched by the targets of miR-129, miR-130a and miR-130b. Moreover, miR-141 is valuable in disscussion part) were analyzed by qRT-PCR to validate the results of high throughput sequencing data. In agreement with sequencing data, miR-129, miR-130b and miR-141 were down-regulated in CM and up-regulated in CH, although no signicance was found in miR-141. No change was found in miR-130b according to qRT-PCR (Figure 10b).

The expression of miR-129, miR-130a, miR-130b and miR-141 was also examined in kidneys of rats in groups of 4 weeks and 26 weeks. Both of miR-130b and miR-141 were up-regulated after administrated with OTA for 4 weeks (Figure 10a). In the 26-week group, the expression of miR-129 and miR-130b were decreased, while miR-141 was increased (Figure 10c). There was no significant effect of OTA on the expression of miR-130a. Primers used in the qRT-PCR were listed in Additional file 1: Table S7.

#### qRT-PCR validation of the miRNA targets

The mRNA expression of *Smoc2/Dcn* (miR-129), *Emp1/Rapgef5* (miR-218b), *lgfbp3/sepp1* (miR-141), *lgfbp3/Sepp1/Col1a2/Edem1* (miR-130a/miR-130b) and *Edem1/Dpt* (miR-3588) at 13 weeks are strongly correlated with its corresponding miRNAs shown in the parentheses. *Smoc2/Dcn, lgfbp3/sepp1, lgfbp3/Sepp1/Col1a2/Edem1* and *Edem1/Dpt* mRNA levels were increased significantly in CM compared to CK group. *Emp1/Rapgef5* mRNA levels were increased in both CM and CH groups (Additional file 1: Figure S4). All the primers used in the qRT-PCR analyses were listed in Additional file 1: Table S8. The expression trendency of these mRNA targets is opposite to the expression of their corresponding miRNAs as shown in the profiling data.

*Dcn* mRNA, a target gene of miR-129, encodes a protein that regulates cell cycle. Furthermore, a surfeit in *Dcn* expression exists in diabetic renal damage. *Dcn* mRNA levels in the kidney of streptozotocin-induced diabetes in mice are rapidly elevated following the induction of diabetes [19]. *Sepp1*, a target of miR-141, is a major extracellular selenoprotein that is synthesized and secreted from the kidneys and plays critical roles in body selenium homeostasis.

## Discussion

The integrity of miRNA processing machanism plays a pivotal role in homeostasis and the regulation of various diseases. Origins of various forms of cancer including ovarian, lung, gastric and breast are accompanied with decreased expression of *Dicer* and *Drosha*[10–13]. Moreover, abrogation of *Drosha*, *DRCG8* or *Dicer* substantially increases the possibility of cellular transformation and tumorigenesis [20]. The observation that Drosha (~2-fold) and Dicer1 (~5-fold) were down-regulated in the kidneys of rats after OTA administration suggests that dysregulation of miRNA processing may attribute to OTA nephrotoxicity and renal carcinogenesis.

How miRNA processing might be associated with OTA-induced renal toxicity and carcinogenesis? It is known that Dicer can stimulate *p-Akt*, *PCNA*[21] and *c-Myc*[20] expression, which is associated with the enhanced proliferation and invasion in tumor cells. On the other hand, *Akt1, c-Myc*[22] and *PCNA*[23], proteins associated with cell survival and proliferation, are up-regulated in the kidney after OTA exposure. This suggests that *Dicer* might mediate the carcinogenicity of OTA by regulating *Akt*, *c-Myc* and *PCNA. Dicer* is also required for the maturation of short interfering RNAs (siRNAs) [24, 25], which specifically degrade their target mRNAs. Thus, OTA-induced down-regulation of *Dicer* might also disturb the RNA interfering (RNAi) system. Decreased expression of *Dicer* (both in the CM and CH groups) and *Drosha* (only in the CH group) in kidneys may position the OTA-gavaged rats at an extremely complex toxicological response, which might include the inhibition of both miRNA and siRNA. *DRCG8* is the only known protein that is specific to the miRNA processing pathway [26]. Because the expression of *DRCG8* is not affected by OTA administration, we speculate that an organism or a cell could initiate other regulatory pathways for the maturation or compensation of endogenous miRNAs, when down-regulation of global expression of miRNAs occurs due to a severe impairment in the classical miRNA processing pathway. Thus, further investigations are needed to better understand OTA-induced inhibitory mechanism of miRNA biogenesis.

Results from hierachical clustering analyses demonstrate that CK and CH groups are closer in euclidean distance. Interestingly, hierachical clustering analysis of the gene expression in rats fed with OTA for less than 12 months indicates that a cluster was noted between the early (7 days) and the late responses (12 months) [22]. These results suggest that OTA may mediate nephrotoxicity in an age-dependent manner. The hierachical clusterings, small RNA and miRNA length distribution pattern suggest that a series of signaling pathways are affected and orchestrated at the early response to OTA toxicity and that the transition period might be of paramount importance in the regulation of nephrotoxicity.

Based on the expression pattern, miRNA expression was decreased in CM and increased in CH, which was consistent with results from our hierachical clusterings. KEGG and GO enrichment analyses were further performed in the six differentially expressed miRNAs (miR-129, miR-130a, miR-130b, miR-141, miR-218b and miR-3588), demonstrating that “phosphatidylinositol signaling system”, “pancreatic cancer” and “MAPK signaling pathway” were mostly significantly enriched. Specifically, several previous studies addressed the involvement of MAPK pathway in the development of OTA-induced toxicity [4, 22, 27, 28]. After the male rats were fed with OTA at a concentration of 300 μg/kg for 12 months, the kidneys were used to analyse key players in the MAPK pathway [4]. OTA administration induces the phosphorylation of *PKCζ*. One of its selective downstream target, *ERK 1/2*, was markedly increased at day 7. The upstream mediator of *PKC* phosphorylation, *PKD*, is also activated by phosphoprotein at day 7. Substrates of *ERK 1/2, ELK 1/2* and *p90RSK* are also significantly activated at days 7 and 21. The expression of insulin-like growth factor 1 receptor (*IGF-1r*), an upstream molecule of *PDK1*, is increased at day 7 and 21, followed by a decline after 12 months of treatment. The aforementioned signals typically lead to cell proliferation. Analyses of the MAPK pathway have helped to confirm that this pathway is critical in the kidneys of rats after OTA administration. After a long-term gavaging, MAPK pathway tends to be progressively repressed. Nonetheless, further transcriptomic and proteomic analyses are needed to provide a full spectrum of the OTA-induced gene expression changes.

Interestingly, our results showed that OTA induced up-expression of miR-130b in the 4 and 13 weeks groups, then a down-expression in the 26 weeks groups, indicating the different effect of OTA on miR-130b between the short- and long-term treatment.

Overexpression of miR-133b in HeLa cells increases tumor necrosis factor-α-induced cell death [29]. Coincidently, OTA exacerbates renal cell proliferation, extending from the medullary rays into the OSOM in a dose- and time-dependent manner [30]. Down-regulation of miR-133b in the CH group implies an undermined programmed cell death mechanism in the kidney and offers a possible explanation of renal cell proliferation stimulated by OTA. Transgelin 2 (*TAGLN2*), an inhibitior of cell proliferation in renal carcinoma (RCC), is an intriguing target of miR-133a. *TAGLN2* is recognized as an oncogene and diminished expression of miR-133a is frequently shown in RCC [31]. Similar result is shown in bladder cancer cell lines [32]. Moreover, miR-133b reduces the expression of glutathione-S-transferase pi (GSTP, encode by *Gstp1* in human), one of the phase II enzymes involved in xenobiotic metabolism. After throughly scrutinizing a transcriptomics result from kidneys of rats after 2 years of daily dietary intake of OTA, we have previously shown that expression of GSTP (encode by *Gstp2* in rat) is impaired [22]. This reveals a possible linkage between miR-133b and GSTP in mediating OTA toxicity.

OTA increases miR-132 and miR-200c expression in porcine renal proximal tubular cells [33]. The induction of these two miRNAs may attenuate the expression of *Nrf2* and HO-1, resulting in the elevation of ROS level and the expression of the profibrotic TGFβ. Future *in vivo* studies are needed to verify the link between Nrf2 and miRNA following OTA toxicology.

*Keap1* is a negative regulator of *Nrf2*[34, 35]. We have herein found that Nrf2 expression was significantly decreased as OTA doses increased. A previous study has demonstrated that OTA induces the disruption of *Nrf2* expression and its corresponding downstream proteins [22]. This has been proved to be associated with an inhibition of *Nrf2* binding to the antioxidant responsive element at the promoter region *in vitro*[36]. According to our results, down-regulation of *Nrf2* might be due to the elevated *Keap1* expression. However, since OTA treatment induces miRNA-141, a regulator of *Keap1*[37], in 2 and 26 weeks after OTA adminstration, it seems impossible that an elevation in the expression of *Keap1* is due to down-regulation of miRNA-141.

## Conclusions

A high-throughput sequencing approach has been used to explore the differentially expressed miRNAs in the kidneys of rats in response to different doses of OTA. MAPK signaling pathway may play a vital role in mediating OTA toxicity. KEGG and GO enrichment analyses have been performed and the results are consistent with the previous OTA studies. Most importantly, we have shown herein that the miRNA processing machanism is severely hampered by OTA treatment. The differentially expressed miRNAs after OTA administration have been analyzed and validated, most of which are in agreement with the sequencing results. These findings provide the first and valuable information for exploring the toxicological mechanism of OTA in the perspective of miRNAs.

## Methods

### Animals

Male F344 rats (6–7 weeks old) were purchased from Vitalriver, Beijing, China. Animals were housed in a stainless steel (three rats/cage) with *ad libitum* access to filtered tap water and commercial feed in a specific pathogen free (SPF) animal room of The Supervision and Testing Center for GMOs food safety, Ministry of Agriculture (Beijing, China). All experimental procedures involving animals were approved (permission number: 120020) by the Ethics Committee of China Agricultural University.

### Study design

After a week of acclimatization, rats (six per group) were administered with OTA at doses of 0, 70 or 210 μg/kg body weight in corn oil (Aladin, Shanghai, China) by gavage for 2, 4, 13 or 26 weeks (5 days per week) [38]. Rats were anesthetized using chloral hydrate (6%, 5 ml/kg, ip) and decapitated. The Kidneys and the livers were weighted, snap-frozen immediately in liquid nitrogen, and stored at −80°C until further analysis.

### Serum clinical chemistry

The serum biochemical parameters were measured using a Hitachi 7020 automatic biochemical analyzer (Hitachi, Tokyo, Japan). They include ALT, alanine aminotransferase; AST, aspartate transaminase; ALB, albumin; ALP, alkaline phosphatase; GLU, glucose; BUN, blood urea nitrogen; CREA, creatinine; HDL, high-density lipoprotein; LDL, low-density lipoprotein; LDH, lactate dehydrogenas.

### Pathology

Samples from the kidneys and the livers were fixed in 4% buffered formaldehyde and embedded in paraffin. The tissue sections (5 μm thick) were affixed to slides and stained with haematoxylin and eosin (H & E) for microscopic examination. Histopathological examination of tissue sections was conducted at the Experimental Animal Research Center, China Agricultural University.

### miRNA library construction and sequencing procedures

Total RNA was extracted from the kidneys using mirVana™ miRNA isolation kit (Ambion, USA) following the manufacturer’s instruction. The quality of the purified RNA was assessed using a BioAnalyzer 2100 (Agilent Technology, Santa Clara, USA) with the parameters: RIN ≥ 7.5, concentration ≥ 200 ng/μl. RNA samples were stored at −80°C and were sequenced with the Solexa/Illumina platform.

For small RNA library construction and deep sequencing, small RNA was enriched by PEG8000 precipitation from 10 μg of total RNA, followed by DNA sequencing with an Illumina Hiseq 2000 (Illumina, San Diego, USA) according to manufacturer’s instruction. Briefly, proprietary adapters were ligated to the 5′ and 3′ termini of these small RNAs, of which the ligated small RNAs were then used as templates for cDNA synthesis. The cDNA was amplified with 15 PCR cycles to generate cDNA libraries. The libraries were quantified by ECO (Illumina, San Diego, USA) and sequenced using the Solexa’s proprietary sequencing-by-synthesis method. The image files generated by the sequencer were then processed to produce digital quality data. After masking of adaptor sequences and removal of contaminated reads, full-length small RNA sequences were selected for further analysis. For quality control, we calculated the average quality score of sites and reads of each sample.

### qRT-PCR

Quantitative real-time PCR (qRT-PCR) was performed on 200 ng of total RNA extracts that had been poly-adenylated and reverse transcribed into cDNA using an anchored oligo(dT) primer (Tiangen, Beijing, China). The miRNA was transcribed into first-strand cDNA using miRcute miRNA first-strand cDNA synthesis kit miRNA (Tiangen, Beijing, China). PCRs were run using the miRcute miRNA qPCR detection kit (Tiangen, Beijing, China). The miRNA primers were designed according to the instrution.

mRNA was transcribed into first-strand cDNA using Quantscript RT Kit (Tiangen, Beijing, China). qRT-PCR was run using the RealMasterMix (SYBR green I) (Tiangen, Beijing, China) with *β-actin* being the internal control. The genes include *Drosha, DRCG8, Dicer1, Nrf2, Keap1* and the target genes of some miRNAs. qRT-PCRs were run on the ABI 7500 Real-time PCR machine (Applied Biosystems, Foster City, USA). Data were analyzed using the delta–delta–Ct method.

### Statistics

Serum clinical chemistry and body weight are expressed as mean ± SD of six individual animals, while miRNA and mRNA are expressed as mean ± SD of four individual animals. Statistical analyses were performed using one-way ANOVA followed by LSD test. A *p* value < 0.05 was considered statistically significant.

### Availability of supporting data

All sequencing data are available through ArrayExpress. Accession number: E-MTAB-2475.

## Electronic supplementary material

Additional file 1: Figure S1: H & E stained kidney sections of a control rat (a) and of ratstreated with 70 (b) and 210 (c) μg/kg b.w. OTA for 26 weeks. **Figure S2.** The detail information of the 8 novel miRNAs. **Figure S3.** miRNA length distribution in CK,CM and CH. **Figure S4.** The expression of target genes of miR-129, miR-218b, miR-141,miR-130a, miR-130b, miR-3588 at 13 weeks. **Table S1.** The sequence, chromatin position and theexpression of the 8 novel miRNAs. **Table S2.** Differentially expressed miRNAs in CK, CMand CH. **Table S3.** The full table of the most significant KEGG pathways (A) and GOBPs (B)for the 6 miRNA targets in separative analysis. **Table S4.** The most significant KEGGpathways (A) and GOBPs (B) for the 6 miRNA targets in collective analysis. **Table S5.** A. The most significant KEGG pathways for the up-regulated miRNAs in CH in separativeanalysis. B. The most significant KEGG pathways for the down-regulated miRNAs in CH inseparative analysis. C. The most significant GOBPs for the up-regulated miRNAs in CH inseparative analysis. D. The most significant GOBPs for the down-regulated miRNAs in CHin separative analysis. **Table S6.** A. The most significant KEGG pathways for the upregulatedmiRNAs in CM in separative analysis. B. The most significant KEGG pathways forthe down-regulated miRNAs in CM in separative analysis. C. The most significant GOBPsfor the up-regulated miRNAs in CM in separative analysis. D. The most significant GOBPsfor the down-regulated miRNAs in CM in separative analysis. **Table S7.** Gene and miRNAspecific primers used in qRT-PCR analysis. **Table S8.** Primers used in qRT-PCR of targetgenes. (DOCX 1 MB)

## Abbreviations

OTA: Ochratoxin A
MAPK: Mitogen-activated protein kinase
ERK 1/2: Extracellular signal-regulated kinase 1 and 2
JNK: C-jun amino-terminal-kinase
pri-miRNAs: Primary miRNAs
pre-miRNAs: Precursor miRNAs
DRCG8: DiGeorge syndrome critical region gene 8 protein
RISC: RNA-induced silencing complex
OSOM: Outer stripe of outer medulla
ALT: Alanine aminotransferase
AST: Aspartate transaminase
ALB: Albumin
ALP: Alkaline phosphatase
GLU: Glucose
BUN: Blood urea nitrogen
CREA: Creatinine
HDL: High-density lipoprotein
LDL: Low-density lipoprotein
LDH: Lactate dehydrogenas
IGF-1r: Insulin-like growth factor 1 receptor
Nrf2: Nuclear factor (erythroid-derived 2)-like 2
Keap1: Kelch like-ECH-associated protein 1
RCC: Renal carcinoma
BC: Bladder cancer
GSTP: Glutathione-S-transferase pi.

## References

[1] van der Merwe KJ, Steyn PS, Fourie L, Scott DB, Theron JJ (1965). Ochratoxin A, a toxic metabolite produced by Aspergillus ochraceus Wilh. Nature.

[2] Bhatnagar D, Yu J, Ehrlich KC (2002). Toxins of filamentous fungi. Chem Immunol.

[3] Pfohl-Leszkowicz A, Manderville RA (2007). Ochratoxin A: an overview on toxicity and carcinogenicity in animals and humans. Mol Nutr Food Res.

[4] Marin-Kuan M, Nestler S, Verguet C, Bezencon C, Piguet D, Delatour T, Mantle P, Cavin C, Schilter B (2007). MAPK-ERK activation in kidney of male rats chronically fed ochratoxin A at a dose causing a significant incidence of renal carcinoma. Toxicol Appl Pharm.

[5] Izzotti A, Calin GA, Arrigo P, Steele VE, Croce CM, De Flora S (2009). Downregulation of microRNA expression in the lungs of rats exposed to cigarette smoke. FASEB J.

[6] Du Y, Xu Y, Ding L, Yao H, Yu H, Zhou T, Si J (2009). Down-regulation of miR-141 in gastric cancer and its involvement in cell growth. J Gastroenterol.

[7] Cui L, Zhou H, Zhao H, Zhou Y, Xu R, Xu X, Zheng L, Xue Z, Xia W, Zhang B (2012). MicroRNA-99a induces G1-phase cell cycle arrest and suppresses tumorigenicity in renal cell carcinoma. BMC Cancer.

[8] Wulfken LM, Moritz R, Ohlmann C, Holdenrieder S, Jung V, Becker F, Herrmann E, Walgenbach-Brünagel G, von Ruecker A, Müller SC (2011). MicroRNAs in renal cell carcinoma: diagnostic implications of serum miR-1233 levels. PLoS One.

[9] Garofalo M, Croce CM (2011). microRNAs: master regulators as potential therapeutics in cancer. Annu Rev Pharmacol Toxicol.

[10] Merritt WM, Lin YG, Han LY, Kamat AA, Spannuth WA, Schmandt R, Urbauer D, Pennacchio LA, Cheng J-F, Nick AM (2008). Dicer, Drosha, and outcomes in patients with ovarian cancer. N Engl J Med.

[11] Karube Y, Tanaka H, Osada H, Tomida S, Tatematsu Y, Yanagisawa K, Yatabe Y, Takamizawa J, Miyoshi S, Mitsudomi T (2005). Reduced expression of Dicer associated with poor prognosis in lung cancer patients. Cancer Sci.

[12] W-n W, Chen Y, Hu T-H (2013). The regulatory mechanism of CCR7 gene expression and its involvement in the metastasis and progression of gastric cancer. Tumor Biol.

[13] Noh H, Hong S, Dong Z, Pan ZK, Jing Q, Huang S (2011). Impaired microRNA processing facilitates breast cancer cell invasion by upregulating urokinase-type plasminogen activator expression. Genes Cancer.

[14] Ambros V, Bartel B, Bartel DP, Burge CB, Carrington JC, Chen X, Dreyfuss G, Eddy SR, Griffiths-Jones S, Marshall M, Matzke M, Ruvkun G, Tuschl T (2003). A uniform system for microRNA annotation. RNA (New York, NY).

[15] Dweep H, Sticht C, Pandey P, Gretz N (2011). miRWalk–database: prediction of possible miRNA binding sites by “walking” the genes of three genomes. J Biomed Inform.

[16] Dweep H, Sticht C, Kharkar A, Pandey P, Gretz N (2013). Parallel analysis of mRNA and microRNA microarray profiles to explore functional regulatory patterns in polycystic kidney disease: using PKD/Mhm rat model. PLoS One.

[17] Felekkis K, Voskarides K, Dweep H, Sticht C, Gretz N, Deltas C (2011). Increased number of microRNA target sites in genes encoded in CNV regions. Evidence for an evolutionary genomic interaction. Mol Biol Evol.

[18] Papagregoriou G, Erguler K, Dweep H, Voskarides K, Koupepidou P, Athanasiou Y, Pierides A, Gretz N, Felekkis KN, Deltas C (2012). A miR-1207-5p binding site polymorphism abolishes regulation of HBEGF and is associated with disease severity in CFHR5 nephropathy. PLoS One.

[19] Mogyorósi A, Ziyadeh FN (1999). What is the role of decorin in diabetic kidney disease?. Nephrol Dial Transpl.

[20] Kumar MS, Lu J, Mercer KL, Golub TR, Jacks T (2007). Impaired microRNA processing enhances cellular transformation and tumorigenesis. Nat Genet.

[21] Han L, Zhang A, Zhou X, Xu P, Wang G-X, Pu P-Y, Kang C-S (2010). Downregulation of Dicer enhances tumor cell proliferation and invasion. Int J Oncol.

[22] Marin-Kuan M, Nestler S, Verguet C, Bezencon C, Piguet D, Mansourian R, Holzwarth J, Grigorov M, Delatour T, Mantle P (2006). A toxicogenomics approach to identify new plausible epigenetic mechanisms of ochratoxin a carcinogenicity in rat. Toxicol Sci.

[23] Mally A, Voelkel W, Amberg A, Kurz M, Wanek P, Eder E, Hard G, Dekant W (2005). Functional, biochemical, and pathological effects of repeated oral administration of ochratoxin A to rats. Chem Res Toxicol.

[24] Hammond SM (2005). Dicing and slicing: the core machinery of the RNA interference pathway. FEBS Lett.

[25] Hutvagner G, McLachlan J, Pasquinelli AE, Bálint É, Tuschl T, Zamore PD (2001). A cellular function for the RNA-interference enzyme Dicer in the maturation of the let-7 small temporal RNA. Sci Signal.

[26] Wang Y, Medvid R, Melton C, Jaenisch R, Blelloch R (2007). DGCR8 is essential for microRNA biogenesis and silencing of embryonic stem cell self-renewal. Nat Genet.

[27] Gekle M, Schwerdt G, Freudinger R, Mildenberger S, Wilflingseder D, Pollack V, Dander M, Schramek H (2000). Ochratoxin A induces JNK activation and apoptosis in MDCK-C7 cells at nanomolar concentrations. J Pharmacol Exp Ther.

[28] Kumar R, Alam S, Chaudhari BP, Dwivedi PD, Jain SK, Ansari KM, Das M (2013). Ochratoxin A-induced cell proliferation and tumor promotion in mouse skin by activating the expression of cyclin-D1 and cyclooxygenase-2 through nuclear factor-kappa B and activator protein-1. Carcinogenesis.

[29] Patron JP, Fendler A, Bild M, Jung U, Müller H, Arntzen MØ, Piso C, Stephan C, Thiede B, Mollenkopf H-J (2012). MiR-133b targets antiapoptotic genes and enhances death receptor-induced apoptosis. PLoS One.

[30] Rached E, Hard GC, Blumbach K, Weber K, Draheim R, Lutz WK, Özden S, Steger U, Dekant W, Mally A (2007). Ochratoxin A: 13-week oral toxicity and cell proliferation in male F344/N rats. Toxicol Sci.

[31] Kawakami K, Enokida H, Chiyomaru T, Tatarano S, Yoshino H, Kagara I, Gotanda T, Tachiwada T, Nishiyama K, Nohata N (2012). The functional significance of miR-1 and miR-133a in renal cell carcinoma. Eur J Cancer.

[32] Yoshino H, Chiyomaru T, Enokida H, Kawakami K, Tatarano S, Nishiyama K, Nohata N, Seki N, Nakagawa M (2011). The tumour-suppressive function of miR-1 and miR-133a targeting TAGLN2 in bladder cancer. Br J Cancer.

[33] Stachurska A, Ciesla M, Kozakowska M, Wolffram S, Boesch‒Saadatmandi C, Rimbach G, Jozkowicz A, Dulak J, Loboda A (2013). Cross‒talk between microRNAs, nuclear factor E2‒related factor 2, and heme oxygenase‒1 in ochratoxin A‒induced toxic effects in renal proximal tubular epithelial cells. Mol Nutr Food Res.

[34] Slocum SL, Kensler TW (2011). Nrf2: control of sensitivity to carcinogens. Arch Toxicol.

[35] Wakabayashi N, Slocum SL, Skoko JJ, Shin S, Kensler TW (2010). When NRF2 talks, who’s listening?. Antioxid Redox Signal.

[36] Cavin C, Delatour T, Marin-Kuan M, Holzhäuser D, Higgins L, Bezencon C, Guignard G, Junod S, Richoz-Payot J, Gremaud E (2007). Reduction in antioxidant defenses may contribute to ochratoxin A toxicity and carcinogenicity. Toxicol Sci.

[37] van Jaarsveld MT, Helleman J, Boersma AW, van Kuijk PF, van Ijcken WF, Despierre E, Vergote I, Mathijssen RH, Berns EM, Verweij J, Pothof J, Wiemer EA (2013). miR-141 regulates KEAP1 and modulates cisplatin sensitivity in ovarian cancer cells. Oncogene.

[38] Peng X-L, Xu W-T, Wang Y, Huang K-L, Liang Z-H, Zhao W-W, Luo Y-B (2010). Mycotoxin ochratoxin A-induced cell death and changes in oxidative metabolism of Arabidopsis thaliana. Plant Cell Rep.

