# Supplementary material for: MicroRNA profiling of rats with ochratoxin A nephrotoxicity
Source: BMC Genomics. 2014 May 5;15(1):333. doi: 10.1186/1471-2164-15-333 (PMC4035064; doi:10.1186/1471-2164-15-333)
Supplement: Supplementary file 1 — Additional file 1: Figure S1: H & E stained kidney sections of a control rat (a) and of ratstreated with 70 (b) and 210 (c) μg/kg b.w. OTA for 26 weeks. Figure S2. The detail information of the 8 novel miRNAs. Figure S3. miRNA length distribution in CK,CM and CH. Figure S4. The expression of target genes of miR-129, miR-218b, miR-141,miR-130a, miR-130b, miR-3588 at 13 weeks. Table S1. The sequence, chromatin position and theexpression of the 8 novel miRNAs. Table S2. Differentially expressed miRNAs in CK, CMand CH. Table S3. The full table of the most significant KEGG pathways (A) and GOBPs (B)for the 6 miRNA targets in separative analysis. Table S4. The most significant KEGGpathways (A) and GOBPs (B) for the 6 miRNA targets in collective analysis. Table S5. A. The most significant KEGG pathways for the up-regulated miRNAs in CH in separativeanalysis. B. The most significant KEGG pathways for the down-regulated miRNAs in CH inseparative analysis. C. The most significant GOBPs for the up-regulated miRNAs in CH inseparative analysis. D. The most significant GOBPs for the down-regulated miRNAs in CHin separative analysis. Table S6. A. The most significant KEGG pathways for the upregulatedmiRNAs in CM in separative analysis. B. The most significant KEGG pathways forthe down-regulated miRNAs in CM in separative analysis. C. The most significant GOBPsfor the up-regulated miRNAs in CM in separative analysis. D. The most significant GOBPsfor the down-regulated miRNAs in CM in separative analysis. Table S7. Gene and miRNAspecific primers used in qRT-PCR analysis. Table S8. Primers used in qRT-PCR of targetgenes. (DOCX 1 MB) [file 12864_2014_6039_MOESM1_ESM.docx]

**Additional file 1**

**microRNA profiling of rats with ochratoxin A nephrotoxicity**

Qiu Dai^a^, Jue Zhao^a^, Xiaozhe Qi^a^, Xiaoyun He^a^, Mingzhang Guo^a^, Harsh Dweep^b^, [Wen-Hsing Cheng](http://www.sciencedirect.com/science/article/pii/S0955286311001951)^c^, Wentao Xu ^a,*^, YunBo Luo^a^, Kai Xia^a^, Norbert Gretz^b^, Kunlun Huang^a*^

E-mail address: [xuwentaoboy@sina.com](mailto:xuwentaoboy@sina.com) (W.T. Xu)

**Figure S1** H & E stained kidney sections of a control rat (a) and of rats treated with 70 (b) and 210 (c) μg/kg b.w. OTA for 26 weeks. At 70 and 210 μg/kg b.w., OTA induced much more severe cytoplasmic vacuolization (thin arrow) and karyomegaly (thick arrow) in proximal tubular epithelial cells in OSOM than that in 13 weeks.

**Figure S2** The detail information of the 8 novel miRNAs.

**Figure S3** miRNA length distribution in CK, CM and CH.

**Figure S4** The expression of target genes of miR-129, miR-218b, miR-141, miR-130a, miR-130b, miR-3588 at 13 weeks. * : It is significantly different compared to control group. C represents control group; M represents 70μg/kg body weight group; H represents 210μg/kg body weight group.

**Table S1** The sequence, chromatin position and the expression of the 8 novel miRNAs.

**Table S2** Differentially expressed miRNAs in CK, CM and CH.

**Table S3** The full table of the most significant KEGG pathways (A) and GOBPs (B) for the 6 miRNA targets in separative analysis.

**Table S4** The most significant KEGG pathways (A) and GOBPs (B) for the 6 miRNA targets in collective analysis.

**Table S5** A The most significant KEGG pathways for the up-regulated miRNAs in CH in separative analysis. B. The most significant KEGG pathways for the down-regulated miRNAs in CH in separative analysis. C. The most significant GOBPs for the up-regulated miRNAs in CH in separative analysis. D. The most significant GOBPs for the down-regulated miRNAs in CH in separative analysis.

**Table S6** A. The most significant KEGG pathways for the up-regulated miRNAs in CM in separative analysis. B. The most significant KEGG pathways for the down-regulated miRNAs in CM in separative analysis. C. The most significant GOBPs for the up-regulated miRNAs in CM in separative analysis. D. The most significant GOBPs for the down-regulated miRNAs in CM in separative analysis.

**Table S7** Gene and miRNA specific primers used in qRT-PCR analysis.

**Table S8** Primers used in qRT-PCR of target genes.

**Supplementary Figure 1**


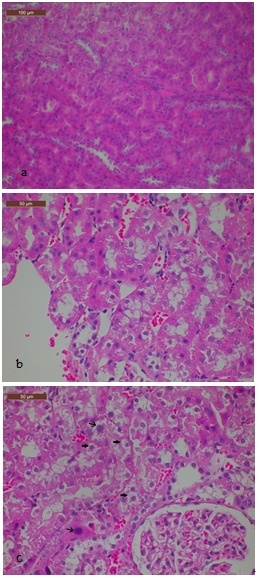


**Supplementary Figure 2**

Novel-m0002-3p

Novel-m0002-3p chr10_83968693_83968777_+ 85(nt) -27.10(kcal/mol)

CTTCTGTATATACCCTGTAGATCCGAATTTGTGTAAGGAATTTTGTGGTCACAAATTCGTATCTAGGGGAATATGTAGTTGACAT

...((((((((.((((.(((((.((((((((((................)))))))))).))))))))).)))))))).......

**********TACCCTGTAGATCCGAATTTGT*****************************************************

Novel-m0028-5p

Novel-m0028-5p chr3_9402734_9402811_+ 78(nt) -30.70(kcal/mol)

GGTGACAGCACATTATTACTTTTGGTACGCGCTGTGACACTTCAAACTCGTACCGTGAGTAATAATGCGTGGTCAACA

..((((.((.(((((((((((.(((((((.....(((....)))....))))))).))))))))))).)).))))...

***********************************************TCGTACCGTGAGTAATAATGC**********

Novel-m0037-5p

Novel-m0037-5p chr4_145680961_145681040_- 80(nt) -35.39(kcal/mol)

CTTTTGAGACAAAGTTCTGAGACACTCTGACTCTGAGTATGATAGAAGTCAGTGCACTACAGAACTTTGTCTCTAGAGGC

((((.((((((((((((((((.....(((((((((.......))).))))))....)).)))))))))))))).))))..

************************************************TCAGTGCACTACAGAACTTTGT**********

Novel-m0040-3p

Novel-m0040-3p chr5_143497754_143497836_- 83(nt) - 40.00(kcal/mol)

CTTTGCTACTGTAAACATCCTTGACTGGAAGCTGTAAGGTGTTGAGAGGAGCTTTCAGTCGGATGTTTACAGCGGCAGGCTGC

.((((((.((((((((((((..(((((((((((................))))))))))))))))))))))).))))))....

**********GTAAACATCCTTGACTGGAAGCT**************************************************

Novel-m0041-3p

Novel-m0041-3p chr6_143765241_143765318_- 78(nt) -37.60(kcal/mol)

 CGCCGGGCGCACCCGTCCCGTTCGTCCCCGGACGTTGCTCTCTGCCCCGGGAACGTCGAGACTGGAGCGCCCGAACTG

...(((((((.((.(((.((..(((.(((((..((........)).))))).))).)).))).)).))))))).....

***********ACCCGTCCCGTTCGTCCCCG************************************************

Novel-m0072-5p

Novel-m0072-5p chr1_39612919_39612999_+ 81(nt) -30.39(kcal/mol)

TCTTACACAGGCTGACCGATTTCTCCTGGTGTTCAGAGTCTGTTTTTGTCTAGCACCATTTGAAATCGGTTATGATGTAGG

.((((((((...(((((((((((...(((((((.(((...........))))))))))...))))))))))))).))))))

***************************************************TAGCACCATTTGAAATCGGTT*********

Novel-m0120-5p

Novel-m0120-5p chr4_58099677_58099755_- 79(nt) **-**25.40(kcal/mol)

TAGAGGATGACTGATTTCTTTTGGTGTTCAGAGTCAATAGAATTTTCTAGCACCATCTGAAATCGGTTATAATGATTGG

......((((((((((((...(((((((.((((...........)))))))))))...)))))))))))).........

************************************************AGCACCATCTGAAATCGGTTA**********

Novel-m0125-3p

Novel-m0125-3p chr7_109228638_109228720_- 83(nt) **-**32.10(kcal/mol)

GTCTGTGTCTGTAAACATCCCCGACTGGAAGCTGTAAGCCACAGCCAAGCTTTCAGTCAGATGTTTGCTGCTACTGGCTCTT

(((.(((...(((((((((...(((((((((((....((....))..))))))))))).)))))))))...))).)))....

**********GTAAACATCCCCGACTGGAAGCT*************************************************

**Supplementary Figure 3**

**Supplementary Figure 4**


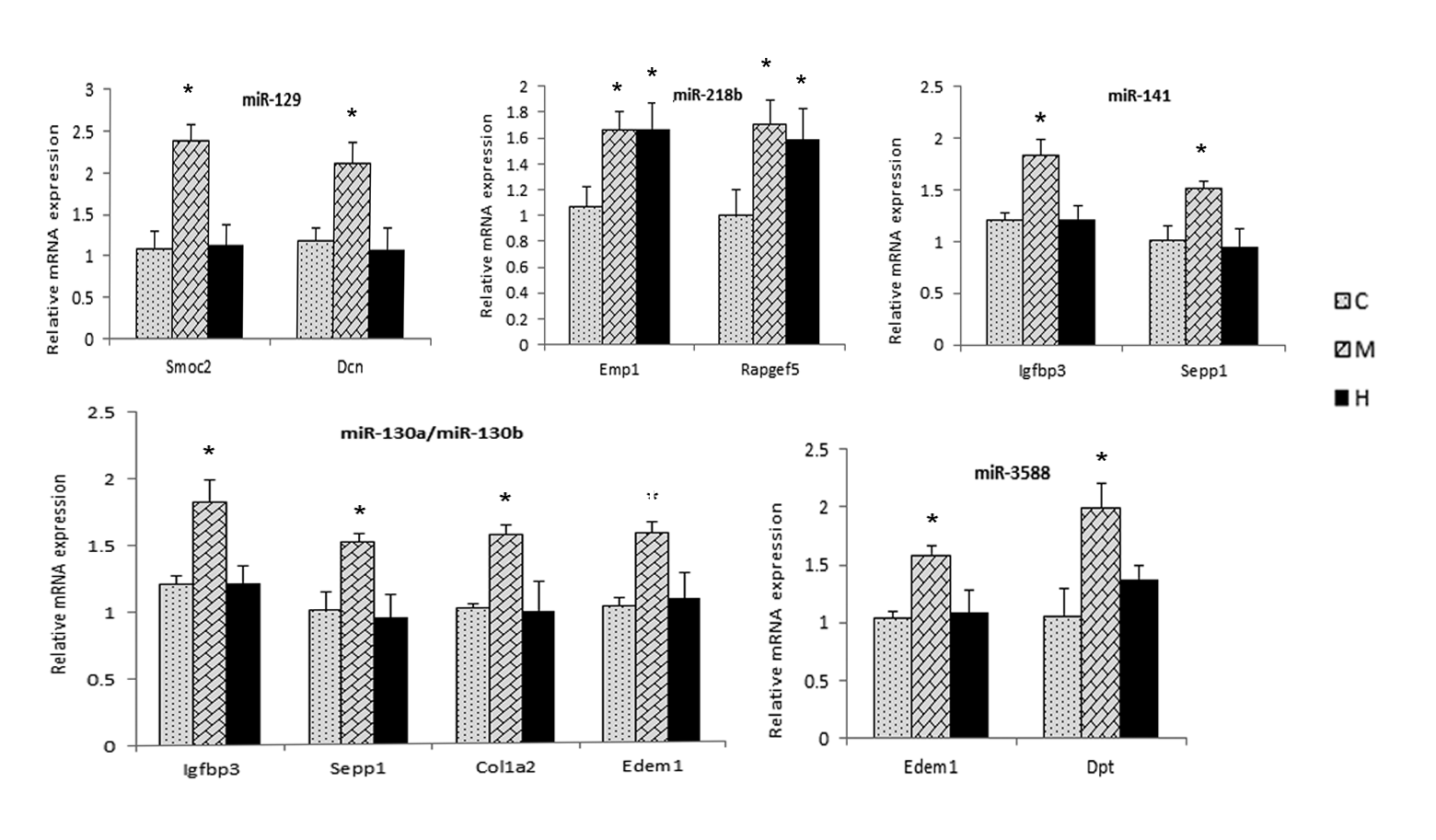


| **Supplementary table 1** | | | | | | |
| --- | --- | --- | --- | --- | --- | --- |
| **Name** | **Sequence** | **Length** | **Hairpin position** | **CK-TPM** | **CM-TPM** | **CH-TPM** |
| Novel-m0002-3p | TACCCTGTAGATCCGAATTTGT | 22 | chr10_83968693_83968777_+ | 25.4 | 0 | 24.8 |
| Novel-m0028-5p | TCGTACCGTGAGTAATAATGC | 21 | chr3_9402734_9402811_+ | 14.3 | 0 | 17.8 |
| Novel-m0037-5p | TCAGTGCACTACAGAACTTTGT | 22 | chr4_145680961_145681040_- | 78.5 | 63.7 | 70.4 |
| Novel-m0040-3p | GTAAACATCCTTGACTGGAAGCT | 23 | chr5_143497754_143497836_- | 20.0 | 0 | 17.9 |
| Novel-m0041-3p | ACCCGTCCCGTTCGTCCCCG | 20 | chr6_143765241_143765318_- | 0.9 | 9.3 | 1.1 |
| Novel-m0072-5p | TAGCACCATTTGAAATCGGTT | 21 | chr1_39612919_39612999_+ | 0 | 26.6 | 0 |
| Novel-m0120-5p | AGCACCATCTGAAATCGGTTA | 21 | chr4_58099677_58099755_- | 0 | 0 | 35.3 |
| Novel-m0125-3p | GTAAACATCCCCGACTGGAAGCT | 23 | chr7_109228638_109228720_- | 0 | 0 | 10.5 |

| **Supplementary Table 2** | | | |
| --- | --- | --- | --- |
| Mature miRNA | CK_Rds Exp TPM | CM_Rds Exp TPM | CH_Rds Exp TPM |
| rno-miR-7b | 39.10517704 | 29.37464209 | 36.00499293 |
| rno-miR-301a-3p | 0.455240711 | 0.365876325 | 0.712970157 |
| rno-miR-99a-3p | 12.01835476 | 10.97628975 | 13.01170537 |
| rno-miR-195-5p | 1524.510092 | 1297.449717 | 1609.218205 |
| rno-miR-582-5p | 43.02024715 | 32.1971166 | 35.24746214 |
| rno-miR-374-5p | 0.136572213 | 0.156804139 | 0.222803174 |
| rno-miR-21-5p | 13644.38353 | 11463.1666 | 15940.58677 |
| rno-miR-3596a | 697.1101001 | 343.6624053 | 5788.872069 |
| rno-miR-30b-5p | 41.06271209 | 40.56000403 | 52.75979162 |
| rno-miR-30e-5p | 4523.362748 | 3827.693576 | 4487.300486 |
| rno-miR-497-3p | 8.695097572 | 8.415155475 | 13.27906917 |
| rno-miR-199a-3p | 1030.847065 | 880.24617 | 1070.569251 |
| rno-miR-138-2-3p | 2.776968335 | 3.554227157 | 2.718198724 |
| rno-miR-3590-3p | 0.273144426 | 0.209072186 | 0.401045713 |
| rno-miR-877 | 0.364192568 | 0.261340232 | 0.445606348 |
| rno-miR-122-5p | 45.9337877 | 24.14783745 | 31.41524754 |
| rno-miR-204-3p | 1.320198061 | 1.097628975 | 1.559622219 |
| rno-miR-3591 | 0 | 0 | 0.267363809 |
| rno-miR-129-5p | 6.327845877 | 0.261340232 | 8.020914267 |
| rno-let-7a-1-3p | 160.1081579 | 127.7431055 | 148.9216415 |
| rno-miR-141-3p | 3.824021969 | 0.679484604 | 3.787653959 |
| rno-miR-450a-3p | 1.7299147 | 1.568041393 | 1.871546662 |
| rno-miR-133a-3p | 3.824021969 | 0.574948511 | 0.133681904 |
| rno-miR-196c-5p | 330.3226596 | 311.1516804 | 396.4114073 |
| rno-miR-152-5p | 0 | 0.104536093 | 0.267363809 |
| rno-miR-125b-5p | 5924.775752 | 4838.923471 | 6183.144566 |
| rno-miR-351-3p | 1.912010984 | 2.352062089 | 2.361713645 |
| rno-miR-194-5p | 5779.872634 | 4615.320768 | 4600.261696 |
| rno-miR-132-3p | 0.455240711 | 0.574948511 | 0.490166983 |
| rno-miR-329-5p | 2.412775766 | 1.515773347 | 1.425940314 |
| rno-miR-181a-1-3p | 9.104814211 | 7.787938918 | 8.778445058 |
| rno-miR-200b-3p | 0.682861066 | 0.679484604 | 0.846652061 |
| rno-miR-26b-3p | 5.918129237 | 4.808660272 | 4.277820942 |
| rno-miR-1843-5p | 6.191273664 | 5.592680968 | 1.826986027 |
| rno-miR-653-5p | 0.136572213 | 2.770206461 | 2.31715301 |
| rno-miR-101a-5p | 5.963653309 | 4.704124179 | 4.322381577 |
| rno-let-7f-2-3p | 36.00954021 | 29.58371428 | 29.1872158 |
| rno-miR-3553 | 148.0442791 | 108.7698046 | 67.28655857 |
| rno-miR-329-3p | 0.318668497 | 0.836288743 | 0.579288253 |
| rno-miR-652-3p | 9.05929014 | 8.885567893 | 6.996019666 |
| rno-miR-450a-5p | 85.22106102 | 85.14464764 | 78.73864172 |
| rno-miR-872-3p | 17.299147 | 19.75732155 | 12.61065965 |
| rno-miR-487b-3p | 1.047053634 | 0.627216557 | 0.980333966 |
| rno-miR-92b-3p | 28.77121291 | 18.39835234 | 20.63157392 |
| rno-miR-22-5p | 60.27387008 | 52.58165471 | 37.16356944 |
| rno-miR-30c-2-3p | 150.548103 | 133.4403225 | 143.3961228 |
| rno-miR-212-3p | 2.503823908 | 2.038453811 | 3.564850785 |
| rno-miR-192-3p | 60.0917738 | 44.48010751 | 34.48993135 |
| rno-miR-200a-3p | 0.956005492 | 0.679484604 | 2.40627428 |
| rno-let-7c-5p | 22446.41714 | 19210.65005 | 24307.0242 |
| rno-miR-135b-3p | 0.182096284 | 0 | 0 |
| rno-miR-130a-3p | 126.830062 | 1.986185764 | 139.2519838 |
| rno-miR-28-3p | 205.3590845 | 209.333526 | 176.9948415 |
| rno-miR-30c-5p | 484.8768808 | 2305.282188 | 2082.897753 |
| rno-miR-27b-3p | 3533.85154 | 3490.041996 | 3592.389257 |
| rno-miR-99b-5p | 937.4771953 | 764.6815193 | 815.6824203 |
| rno-miR-214-3p | 2.367251695 | 2.352062089 | 2.049789201 |
| rno-miR-6329 | 0 | 0.104536093 | 0 |
| rno-miR-32-5p | 0.136572213 | 0.104536093 | 0.178242539 |
| rno-miR-15b-3p | 4.233738608 | 4.129175668 | 3.342047611 |
| rno-miR-27b-5p | 4.32478675 | 4.129175668 | 4.589745386 |
| rno-miR-122-3p | 0.40971664 | 0 | 0.178242539 |
| rno-miR-215 | 242.2791062 | 164.6966143 | 131.1419483 |
| rno-miR-340-5p | 146.6330329 | 115.6169187 | 134.1720714 |
| rno-miR-352 | 10.15186785 | 13.27608379 | 12.78890219 |
| rno-miR-425-3p | 2.776968335 | 2.561134275 | 2.40627428 |
| rno-miR-323-3p | 1.957535055 | 1.411237254 | 1.648743488 |
| rno-miR-186-5p | 8.513001288 | 6.272165572 | 0.980333966 |
| rno-miR-133b-3p | 3.824021969 | 0.574948511 | 0.133681904 |
| rno-miR-433-3p | 0.136572213 | 0 | 0.133681904 |
| rno-miR-380-3p | 0.637336995 | 0.574948511 | 0.356485079 |
| rno-miR-299b-5p | 0.591812924 | 0.836288743 | 0.534727618 |
| rno-miR-488-3p | 0.136572213 | 0.104536093 | 0.133681904 |
| rno-miR-451-5p | 98.28646941 | 75.52732709 | 56.81480939 |
| rno-miR-300-3p | 12.88331211 | 9.983196868 | 11.22927997 |
| rno-miR-101b-3p | 333.9190612 | 279.0590999 | 265.3140197 |
| rno-miR-503-5p | 33.68781258 | 39.20103482 | 36.31691737 |
| rno-miR-124-3p | 0.182096284 | 0.209072186 | 0.267363809 |
| rno-miR-125a-5p | 1617.470245 | 1286.630231 | 1455.17209 |
| rno-miR-3571 | 4.871075603 | 3.65876325 | 6.951459031 |
| rno-miR-30a-3p | 198.9401905 | 177.3977496 | 183.7234973 |
| rno-let-7a-2-3p | 7.420423582 | 5.435876829 | 6.193928239 |
| rno-miR-504 | 0.500764782 | 0.209072186 | 4.188699673 |
| rno-miR-16-5p | 1524.510092 | 1297.449717 | 1609.218205 |
| rno-miR-23b-5p | 1.638866558 | 1.829381625 | 1.826986027 |
| rno-miR-322-3p | 27.08682228 | 25.87268298 | 26.73638089 |
| rno-miR-196c-3p | 0.091048142 | 0 | 0.08912127 |
| rno-miR-342-3p | 261.2626438 | 214.7171347 | 230.1111182 |
| rno-miR-499-5p | 567.9583105 | 495.4488121 | 406.3038682 |
| rno-miR-299a-5p | 2.959064619 | 1.515773347 | 1.604182853 |
| rno-miR-345-3p | 15.34161195 | 9.617320543 | 10.07070347 |
| rno-miR-3473 | 2.230679482 | 6.794846036 | 3.921335864 |
| rno-miR-148b-3p | 536.9108941 | 481.9636561 | 566.0091834 |
| rno-miR-146b-5p | 33.77886072 | 30.10639474 | 44.29327101 |
| rno-miR-190a-5p | 92.00414761 | 89.22155526 | 88.27461757 |
| rno-miR-874-3p | 51.21457994 | 41.29175668 | 44.33783164 |
| rno-miR-19a-5p | 0.455240711 | 1.045360929 | 0.623848887 |
| rno-miR-410-3p | 0.591812924 | 0.627216557 | 0.802091427 |
| rno-miR-106b-5p | 0.273144426 | 0.365876325 | 0.401045713 |
| rno-miR-378a-5p | 0.546288853 | 0.418144371 | 0.579288253 |
| rno-miR-17-5p | 171.1705072 | 151.2637264 | 133.5036619 |
| rno-miR-134-3p | 0 | 0 | 0.08912127 |
| rno-miR-210-3p | 0.273144426 | 0.156804139 | 0 |
| rno-miR-130b-5p | 0.227620355 | 0.627216557 | 0.623848887 |
| rno-miR-672-5p | 0.182096284 | 0.470412418 | 0.445606348 |
| rno-miR-494-3p | 6.100225522 | 7.212990408 | 4.233260307 |
| rno-miR-328a-3p | 2.003059127 | 1.306701161 | 0.935773331 |
| rno-miR-7a-5p | 39.10517704 | 29.37464209 | 36.00499293 |
| rno-miR-212-5p | 0.728385137 | 0.470412418 | 1.648743488 |
| rno-miR-23a-3p | 192.0205317 | 164.383006 | 176.9948415 |
| rno-miR-335 | 28.13387591 | 29.89732256 | 21.79015042 |
| rno-miR-23a-5p | 1.047053634 | 1.306701161 | 0.891212696 |
| rno-miR-375-3p | 0.136572213 | 0 | 0 |
| rno-miR-339-3p | 22.4888911 | 20.28000202 | 18.9382698 |
| rno-miR-181b-2-3p | 0.136572213 | 0.156804139 | 0.222803174 |
| rno-miR-125b-2-3p | 20.53135605 | 14.89639323 | 19.2056336 |
| rno-miR-350 | 3.277733116 | 3.867835436 | 1.782425393 |
| rno-miR-190b-5p | 6.46441809 | 6.324433618 | 8.555641884 |
| rno-miR-125a-3p | 5.553936669 | 5.331340736 | 4.990791099 |
| rno-miR-423-5p | 36.51030499 | 34.4446426 | 34.13344627 |
| rno-miR-137-3p | 1.047053634 | 1.149897021 | 1.737864758 |
| rno-miR-206-3p | 18.34620064 | 13.22381575 | 13.90291806 |
| rno-miR-532-5p | 6.646514374 | 6.219897525 | 7.619868553 |
| rno-let-7a-5p | 22446.41714 | 19210.65005 | 24307.0242 |
| rno-miR-1306-5p | 3.960594182 | 2.979278647 | 3.208365707 |
| rno-miR-216a-5p | 1.27467399 | 0.365876325 | 0.757530792 |
| rno-miR-543-3p | 0.091048142 | 0 | 0 |
| rno-miR-190a-3p | 1.411246203 | 3.449691064 | 0.133681904 |
| rno-miR-582-3p | 2.640396121 | 3.083814739 | 2.851880628 |
| rno-miR-191a-5p | 2226.582315 | 1678.745115 | 1902.694546 |
| rno-miR-3570 | 8.740621643 | 9.094640079 | 8.867566328 |
| rno-miR-92a-3p | 28.77121291 | 18.39835234 | 20.63157392 |
| rno-miR-99b-3p | 8.877193856 | 7.212990408 | 7.708989823 |
| rno-miR-541-5p | 14.4766546 | 12.387527 | 12.1650533 |
| rno-miR-181d-5p | 254.0698406 | 191.8759985 | 222.5358103 |
| rno-miR-26a-5p | 22718.37794 | 18280.59244 | 21372.34991 |
| rno-miR-6215 | 4.279262679 | 4.076907622 | 4.589745386 |
| rno-miR-376c-3p | 10.15186785 | 10.29680515 | 8.555641884 |
| rno-miR-509-5p | 1.957535055 | 1.724845532 | 1.29225841 |
| rno-miR-21-3p | 0.091048142 | 0 | 0.08912127 |
| rno-miR-221-5p | 3.915070111 | 3.81556739 | 4.366942212 |
| rno-miR-328b-3p | 0.591812924 | 0 | 0.133681904 |
| rno-miR-203b-3p | 0.136572213 | 0.209072186 | 0 |
| rno-miR-107-3p | 5125.964877 | 4053.700609 | 4656.898263 |
| rno-miR-34a-5p | 57.22375732 | 55.14278899 | 73.213123 |
| rno-miR-429 | 0.637336995 | 0.679484604 | 0.846652061 |
| rno-miR-31a-3p | 1.047053634 | 1.202165068 | 1.069455236 |
| rno-let-7f-5p | 22446.41714 | 19210.65005 | 24307.0242 |
| rno-let-7b-5p | 22446.41714 | 19210.65005 | 24307.0242 |
| rno-miR-151-5p | 2.868016477 | 2.352062089 | 1.604182853 |
| rno-miR-187-3p | 0 | 0 | 0.08912127 |
| rno-miR-455-3p | 174.1295718 | 156.8041393 | 184.0799824 |
| rno-let-7f-1-3p | 2.458299837 | 3.136082786 | 2.985562533 |
| rno-let-7c-1-3p | 1.138101776 | 1.4635053 | 1.11401587 |
| rno-miR-194-3p | 14.52217867 | 12.43979505 | 11.36296188 |
| rno-miR-16-3p | 0 | 0 | 0.08912127 |
| rno-miR-103-1-5p | 0.318668497 | 0.418144371 | 0.935773331 |
| rno-miR-19b-1-5p | 0.182096284 | 0.209072186 | 0.267363809 |
| rno-miR-142-5p | 0.136572213 | 0.156804139 | 0.178242539 |
| rno-miR-3556b | 30.81979611 | 23.20701262 | 311.2560342 |
| rno-miR-379-3p | 1.27467399 | 1.045360929 | 1.336819044 |
| rno-miR-28-5p | 37.37526234 | 33.86969409 | 29.98930723 |
| rno-miR-107-5p | 0.318668497 | 0.418144371 | 0.935773331 |
| rno-miR-339-5p | 156.8759489 | 154.9747577 | 143.4852441 |
| rno-miR-205 | 0 | 0 | 0.133681904 |
| rno-miR-151-3p | 1.866486913 | 1.620309439 | 0.490166983 |
| rno-miR-182 | 1.183625847 | 0.104536093 | 0.08912127 |
| rno-miR-542-5p | 39.33279739 | 34.54917869 | 41.04034466 |
| rno-miR-181a-5p | 1300.85033 | 1035.273196 | 1276.929551 |
| rno-miR-30b-3p | 4.32478675 | 2.508866229 | 3.342047611 |
| rno-miR-103-3p | 5125.964877 | 4053.700609 | 4656.898263 |
| rno-miR-10b-5p | 6.282321806 | 5.592680968 | 6.728655857 |
| rno-miR-203b-5p | 0.682861066 | 0.209072186 | 0.445606348 |
| rno-miR-674-5p | 19.75744684 | 23.36381675 | 21.38910471 |
| rno-miR-412-5p | 0.364192568 | 0.261340232 | 0.311924444 |
| rno-miR-449a-5p | 2.276203553 | 2.142989904 | 1.693304123 |
| rno-miR-20a-5p | 171.1705072 | 151.2637264 | 133.5036619 |
| rno-miR-30c-1-3p | 13.70274539 | 12.07391873 | 13.85835743 |
| rno-miR-376a-3p | 24.62852244 | 21.74350732 | 19.33931551 |
| rno-miR-144-3p | 1192.229897 | 842.4563724 | 797.4571206 |
| rno-miR-135a-5p | 74.75052468 | 29.00876577 | 8.154596171 |
| rno-miR-3596b | 1.092577705 | 11.81257849 | 16.84391996 |
| rno-miR-127-5p | 0.091048142 | 0.156804139 | 0.133681904 |
| rno-miR-20b-5p | 171.1705072 | 151.2637264 | 133.5036619 |
| rno-miR-222-3p | 9.696627135 | 11.23762998 | 9.357733311 |
| rno-miR-17-1-3p | 58.90814795 | 50.80454113 | 53.65100432 |
| rno-miR-499-3p | 1.775438771 | 2.456598182 | 1.425940314 |
| rno-miR-29c-5p | 30.00036283 | 32.9811373 | 19.02739107 |
| rno-miR-33-3p | 1.638866558 | 1.045360929 | 1.470500949 |
| rno-let-7c-2-3p | 160.1081579 | 127.7431055 | 148.9216415 |
| rno-miR-541-3p | 0.227620355 | 0.156804139 | 0.08912127 |
| rno-miR-92a-1-5p | 5.644984811 | 4.704124179 | 6.951459031 |
| rno-miR-342-5p | 0.364192568 | 0.313608279 | 0.668409522 |
| rno-miR-29a-3p | 8682.21426 | 6476.115489 | 7663.716218 |
| rno-miR-1249 | 0 | 0.209072186 | 0 |
| rno-miR-204-5p | 47.29950983 | 54.88144875 | 41.2185872 |
| rno-miR-30a-5p | 4523.362748 | 3827.693576 | 4487.300486 |
| rno-miR-130b-3p | 126.830062 | 1.986185764 | 139.2519838 |
| rno-miR-99a-5p | 2207.917446 | 1779.517909 | 2165.691413 |
| rno-miR-222-5p | 0.091048142 | 0 | 0 |
| rno-let-7i-5p | 9776.886073 | 7629.828078 | 8202.766217 |
| rno-miR-802-3p | 2.13963134 | 1.4635053 | 0.712970157 |
| rno-miR-3588 | 16.34314151 | 0.522680464 | 19.29475487 |
| rno-miR-18a-5p | 11.7907344 | 10.66268147 | 11.67488632 |
| rno-miR-494-5p | 0 | 0 | 0.08912127 |
| rno-miR-154-5p | 3.186684974 | 2.19525795 | 2.094349836 |
| rno-miR-361-5p | 0.318668497 | 0.156804139 | 0.133681904 |
| rno-miR-434-5p | 0.136572213 | 0 | 0.08912127 |
| rno-miR-19a-3p | 533.678685 | 2173.253103 | 508.2586007 |
| rno-miR-223-5p | 0.182096284 | 0.209072186 | 0.267363809 |
| rno-miR-301b-3p | 0.455240711 | 0.365876325 | 0.712970157 |
| rno-miR-802-5p | 5.59946074 | 2.717938414 | 1.515061584 |
| rno-miR-1843-3p | 0.637336995 | 0.209072186 | 0.311924444 |
| rno-miR-153-3p | 0.910481421 | 0.522680464 | 0.579288253 |
| rno-miR-376b-5p | 3.00458869 | 2.456598182 | 2.807319993 |
| rno-miR-146a-5p | 20.30373569 | 21.06402271 | 27.22654787 |
| rno-miR-196b-5p | 330.3226596 | 311.1516804 | 396.4114073 |
| rno-miR-320-3p | 624.635779 | 496.6509772 | 547.026353 |
| rno-miR-455-5p | 50.03095409 | 49.91598434 | 47.18971227 |
| rno-miR-539-3p | 0.273144426 | 0.365876325 | 0.222803174 |
| rno-miR-20a-3p | 0.364192568 | 0.261340232 | 0.311924444 |
| rno-let-7i-3p | 8.740621643 | 9.512784451 | 8.020914267 |
| rno-miR-24-3p | 762.5281902 | 719.365123 | 758.4220045 |
| rno-miR-330-5p | 2.003059127 | 1.986185764 | 2.272592376 |
| rno-miR-142-3p | 0.364192568 | 0.470412418 | 0.445606348 |
| rno-miR-211-5p | 47.29950983 | 54.88144875 | 41.2185872 |
| rno-miR-299a-3p | 1.138101776 | 0.679484604 | 0.623848887 |
| rno-miR-93-5p | 0.455240711 | 0.73175265 | 0.712970157 |
| rno-miR-6333 | 0.136572213 | 0 | 0.08912127 |
| rno-miR-362-3p | 0.364192568 | 0.156804139 | 0.133681904 |
| rno-miR-376c-5p | 2.412775766 | 2.142989904 | 2.138910471 |
| rno-miR-381-3p | 0.273144426 | 0.156804139 | 0.133681904 |
| rno-miR-330-3p | 0.86495735 | 0.993092882 | 1.069455236 |
| rno-let-7d-5p | 13215.68335 | 11112.34348 | 13374.11701 |
| rno-miR-136-3p | 0.591812924 | 0.104536093 | 0.356485079 |
| rno-miR-29a-5p | 0.500764782 | 0.156804139 | 0.133681904 |
| rno-miR-34a-3p | 0.500764782 | 0.209072186 | 0.579288253 |
| rno-miR-3585-5p | 0.136572213 | 0.156804139 | 0.178242539 |
| rno-miR-2964 | 0.591812924 | 0.470412418 | 0.08912127 |
| rno-miR-218a-5p | 813.1509572 | 617.9128449 | 589.8936837 |
| rno-miR-3574 | 239.3200415 | 204.4725976 | 209.3013017 |
| rno-miR-1839-5p | 117.7707718 | 90.8418647 | 95.49344041 |
| rno-miR-203a-3p | 470.8554669 | 420.6532377 | 396.2331648 |
| rno-miR-3587 | 46.88979319 | 46.10041695 | 4.767987925 |
| rno-miR-411-5p | 14.06693796 | 11.96938263 | 12.25417457 |
| rno-miR-186-3p | 0.136572213 | 0 | 0.222803174 |
| rno-miR-485-5p | 0.40971664 | 0.365876325 | 0.623848887 |
| rno-miR-1839-3p | 1.593342487 | 1.515773347 | 1.381379679 |
| rno-miR-22-3p | 1585.922063 | 1545.461597 | 1185.535689 |
| rno-miR-369-3p | 44.24939707 | 33.50381776 | 35.0692196 |
| rno-miR-511-3p | 1.365722132 | 0.993092882 | 1.960667932 |
| rno-miR-544-3p | 0.318668497 | 0.156804139 | 0 |
| rno-miR-221-3p | 222.6582315 | 186.1787814 | 225.2985696 |
| rno-miR-378b | 133.2034319 | 94.76196818 | 23.43889391 |
| rno-miR-128-3p | 148.1808513 | 122.6208369 | 10.82823426 |
| rno-miR-33-5p | 12.33702326 | 11.91711459 | 8.644763154 |
| rno-miR-126a-5p | 2842.431949 | 2516.497363 | 2761.021494 |
| rno-miR-9b-5p | 1.001529563 | 0.209072186 | 0.802091427 |
| rno-miR-598-3p | 12.24597511 | 8.833299847 | 10.51630982 |
| rno-miR-19b-3p | 533.678685 | 2173.253103 | 508.2586007 |
| rno-miR-532-3p | 0.227620355 | 0 | 0 |
| rno-miR-382-5p | 12.51911954 | 13.06701161 | 12.47697775 |
| rno-miR-708-5p | 43.29339158 | 34.28783846 | 41.12946593 |
| rno-miR-3590-5p | 32.18551824 | 128.2657859 | 32.75206659 |
| rno-miR-500-3p | 30.13693504 | 30.62907521 | 26.51357771 |
| rno-miR-138-5p | 5.417364456 | 0.470412418 | 1.782425393 |
| rno-miR-425-5p | 211.6869304 | 158.0063044 | 178.7772669 |
| rno-miR-341 | 0.136572213 | 0.209072186 | 0.133681904 |
| rno-miR-3596c | 697.1101001 | 137.8831065 | 5788.872069 |
| rno-miR-196a-5p | 330.3226596 | 311.1516804 | 396.4114073 |
| rno-miR-134-5p | 8.012236506 | 5.749485107 | 6.238488874 |
| rno-miR-379-5p | 19.07458577 | 14.21690863 | 15.64078282 |
| rno-miR-152-3p | 536.9108941 | 481.9636561 | 566.0091834 |
| rno-miR-183-5p | 0.136572213 | 0.365876325 | 0.08912127 |
| rno-miR-154-3p | 0.546288853 | 0.522680464 | 0.133681904 |
| rno-miR-138-1-3p | 0 | 0.104536093 | 0.267363809 |
| rno-miR-3075 | 0.227620355 | 0 | 0.133681904 |
| rno-miR-3589 | 3.277733116 | 4.3905159 | 2.272592376 |
| rno-miR-10a-5p | 8712.305671 | 6785.280984 | 8310.38015 |
| rno-miR-24-1-5p | 0.091048142 | 0.156804139 | 0 |
| rno-miR-411-3p | 0.591812924 | 0.679484604 | 0.712970157 |
| rno-miR-26b-5p | 3137.382405 | 2663.318306 | 2936.23391 |
| rno-miR-217-5p | 0.455240711 | 0.104536093 | 0.311924444 |
| rno-let-7b-3p | 22.85308367 | 17.71886774 | 15.95270726 |
| rno-miR-101b-5p | 0 | 0 | 0.08912127 |
| rno-miR-3556a | 0.273144426 | 23.20701262 | 0.222803174 |
| rno-miR-362-5p | 71.01755085 | 57.65165521 | 68.04408936 |
| rno-miR-201-3p | 0.182096284 | 0 | 0.178242539 |
| rno-miR-466b-1-3p | 0.364192568 | 0.156804139 | 0.08912127 |
| rno-miR-708-3p | 2.458299837 | 1.933917718 | 3.475729516 |
| rno-miR-501-3p | 2.776968335 | 3.345154972 | 3.119244437 |
| rno-miR-336-5p | 0.136572213 | 0 | 0.08912127 |
| rno-miR-218b | 20.62240419 | 0.209072186 | 13.76923616 |
| rno-miR-98-5p | 22446.41714 | 19210.65005 | 24307.0242 |
| rno-miR-150-5p | 51.53324844 | 57.8607274 | 47.01146973 |
| rno-miR-9a-5p | 201.4895385 | 176.1955845 | 234.2106966 |
| rno-miR-490-3p | 0.182096284 | 0.261340232 | 0.222803174 |
| rno-miR-135b-5p | 74.75052468 | 29.00876577 | 8.154596171 |
| rno-miR-29b-3p | 6316.191715 | 4851.467802 | 5400.347894 |
| rno-miR-1-3p | 0.728385137 | 0.836288743 | 0.802091427 |
| rno-miR-872-5p | 139.2581334 | 116.2441353 | 128.735674 |
| rno-miR-324-3p | 10.88025298 | 11.08082584 | 8.555641884 |
| rno-miR-15b-5p | 230.7615162 | 219.5780631 | 224.9420845 |
| rno-miR-668 | 0.227620355 | 0.313608279 | 0.490166983 |
| rno-miR-145-5p | 0.455240711 | 1.933917718 | 0.757530792 |
| rno-miR-503-3p | 4.097166395 | 4.076907622 | 4.589745386 |
| rno-miR-466b-2-3p | 0.136572213 | 0 | 0.08912127 |
| rno-miR-322-5p | 505.6813813 | 410.7223089 | 537.1338921 |
| rno-miR-542-3p | 55.03860191 | 47.04124179 | 59.93405383 |
| rno-miR-380-5p | 0.091048142 | 0.209072186 | 0.178242539 |
| rno-miR-200c-3p | 0.227620355 | 0.679484604 | 0.846652061 |
| rno-miR-144-5p | 20.03059127 | 17.30072337 | 12.87802346 |
| rno-miR-188-5p | 0 | 0.104536093 | 0.08912127 |
| rno-miR-10b-3p | 6.737562516 | 5.226804643 | 6.327610144 |
| rno-miR-147 | 3.368781258 | 2.717938414 | 3.342047611 |
| rno-miR-377-3p | 0.182096284 | 0.418144371 | 0.222803174 |
| rno-miR-374-3p | 0.091048142 | 0.104536093 | 0 |
| rno-miR-143-3p | 38.92308075 | 20.43680615 | 28.11776057 |
| rno-miR-874-5p | 1.684390629 | 1.515773347 | 1.024894601 |
| rno-miR-496-3p | 0.364192568 | 0.365876325 | 0.178242539 |
| rno-miR-223-3p | 19.07458577 | 18.55515648 | 21.16630154 |
| rno-miR-17-2-3p | 0 | 0 | 0.133681904 |
| rno-miR-25-3p | 0.227620355 | 0.104536093 | 0.178242539 |
| rno-miR-181b-5p | 254.0698406 | 191.8759985 | 222.5358103 |
| rno-miR-351-5p | 37.23869012 | 33.29474558 | 39.56984372 |
| rno-miR-509-3p | 0.364192568 | 0.209072186 | 0.178242539 |
| rno-miR-3072 | 0 | 0 | 0.08912127 |
| rno-miR-326-3p | 23.35384845 | 16.41216658 | 14.17028187 |
| rno-miR-140-5p | 109.6219631 | 91.46908126 | 98.25619977 |
| rno-miR-338-3p | 0.364192568 | 1.097628975 | 1.515061584 |
| rno-miR-30d-5p | 4523.362748 | 3827.693576 | 4487.300486 |
| rno-let-7e-5p | 22446.41714 | 19210.65005 | 24307.0242 |
| rno-miR-301a-5p | 0.318668497 | 0.209072186 | 0.356485079 |
| rno-miR-29b-1-5p | 0.091048142 | 0 | 0 |
| rno-miR-31a-5p | 72.42879705 | 58.90608833 | 78.24847473 |
| rno-miR-129-2-3p | 1.27467399 | 1.306701161 | 2.629077454 |
| rno-miR-3586-3p | 205.3590845 | 209.333526 | 176.9948415 |
| rno-miR-199a-5p | 309.1084425 | 299.8095143 | 356.9306849 |
| rno-miR-615 | 4.780027461 | 3.81556739 | 4.010457133 |
| rno-miR-30e-3p | 198.9401905 | 177.3977496 | 183.7234973 |
| rno-miR-484 | 0.136572213 | 0.261340232 | 0.08912127 |
| rno-miR-29b-2-5p | 1.866486913 | 1.672577486 | 1.515061584 |
| rno-miR-434-3p | 0.728385137 | 0.836288743 | 0.846652061 |
| rno-let-7e-3p | 7.420423582 | 5.435876829 | 6.193928239 |
| rno-miR-150-3p | 0.091048142 | 0.209072186 | 0.08912127 |
| rno-miR-497-5p | 191.3831947 | 143.005375 | 133.5927832 |
| rno-miR-185-5p | 618.899746 | 500.3620085 | 575.2332348 |
| rno-miR-674-3p | 14.43113053 | 12.02165068 | 15.1060552 |
| rno-miR-378a-3p | 133.2034319 | 94.76196818 | 23.43889391 |
| rno-miR-324-5p | 37.51183455 | 35.80361181 | 37.20813007 |
| rno-miR-3557-3p | 190.7458577 | 154.9747577 | 132.3005248 |
| rno-miR-23b-3p | 192.0205317 | 164.383006 | 176.9948415 |
| rno-miR-126a-3p | 4193.677426 | 4156.982269 | 4424.514552 |
| rno-miR-300-5p | 0 | 0.104536093 | 0 |
| rno-miR-485-3p | 2.276203553 | 1.411237254 | 1.29225841 |
| rno-miR-9a-3p | 18.02753214 | 15.99402221 | 19.02739107 |
| rno-miR-195-3p | 2.185155411 | 1.672577486 | 2.049789201 |
| rno-miR-3557-5p | 5407.030492 | 4649.033658 | 4326.12467 |
| rno-miR-100-5p | 2207.917446 | 1779.517909 | 2165.691413 |
| rno-miR-10a-3p | 122.7328956 | 102.8635154 | 98.96916992 |
| rno-miR-148b-5p | 2.822492406 | 3.449691064 | 2.629077454 |
| rno-miR-125b-1-3p | 5.098695958 | 4.756392225 | 5.035351734 |
| rno-miR-192-5p | 3469.617076 | 2581.518813 | 2261.67502 |
| rno-miR-7a-2-3p | 2.230679482 | 1.829381625 | 1.559622219 |
| rno-miR-132-5p | 2.549347979 | 1.881649672 | 2.629077454 |
| rno-miR-203a-5p | 10.0608197 | 7.108454315 | 3.787653959 |
| rno-miR-200b-5p | 0 | 0 | 0.08912127 |
| rno-miR-345-5p | 9.013766069 | 5.27907269 | 7.174262205 |
| rno-miR-3065-5p | 0.40971664 | 0.261340232 | 0.401045713 |
| rno-miR-3583-5p | 0.091048142 | 0.209072186 | 0.08912127 |
| rno-miR-30d-3p | 198.9401905 | 177.3977496 | 183.7234973 |
| rno-miR-139-5p | 8.330905004 | 9.042372033 | 8.199156806 |
| rno-miR-96-5p | 0.591812924 | 0.156804139 | 0.08912127 |
| rno-miR-409a-5p | 4.142690466 | 3.188350832 | 3.69853269 |
| rno-miR-196a-3p | 23.21727624 | 21.84804341 | 29.14265517 |
| rno-miR-547-3p | 0.136572213 | 0.209072186 | 0.08912127 |
| rno-miR-29c-3p | 8682.21426 | 6476.115489 | 7663.716218 |
| rno-miR-376b-3p | 4.916599674 | 4.076907622 | 3.252926341 |
| rno-let-7d-3p | 464.3000007 | 361.7994174 | 342.0028722 |
| rno-miR-1306-3p | 0.091048142 | 0.156804139 | 0 |
| rno-miR-3065-3p | 0.091048142 | 0.156804139 | 1.916107297 |
| rno-miR-490-5p | 0 | 0.104536093 | 0 |
| rno-miR-340-3p | 1.912010984 | 3.345154972 | 2.094349836 |
| rno-miR-3548 | 0.956005492 | 0.940824836 | 0.846652061 |
| rno-miR-181a-2-3p | 0.136572213 | 0 | 0.133681904 |
| rno-miR-129-1-3p | 0.091048142 | 0 | 0 |
| rno-miR-24-2-5p | 0.364192568 | 0.418144371 | 0.133681904 |
| rno-miR-3596d | 697.1101001 | 137.8831065 | 165.0080307 |
| rno-miR-101a-3p | 333.9190612 | 279.0590999 | 265.3140197 |
| rno-miR-27a-3p | 3533.85154 | 3490.041996 | 3592.389257 |
| rno-miR-27a-5p | 10.47053634 | 11.65577435 | 12.69978092 |
| rno-miR-331-3p | 0.091048142 | 0.156804139 | 0 |
| rno-miR-423-3p | 53.62735571 | 53.78381978 | 40.95122339 |
| rno-miR-106b-3p | 0 | 0 | 0.08912127 |

Supplementary table 3

A

| Pathways | Targeted by different miRNAs | rno-miR-129 | rno-miR-130a | rno-miR-130b | rno-miR-141 | rno-miR-218b | rno-miR-3588 |
| --- | --- | --- | --- | --- | --- | --- | --- |
| rno04070 Phosphatidylinositol signaling system | 4 | 1 | 1 | 1 | 0 | 1 | 0 |
| rno05212 Pancreatic cancer | 3 | 1 | 1 | 1 | 0 | 0 | 0 |
| rno04971 Gastric acid secretion | 3 | 1 | 1 | 1 | 0 | 0 | 0 |
| rno04010 MAPK signaling pathway | 3 | 1 | 1 | 1 | 0 | 0 | 0 |
| rno04622 RIG-I-like receptor signaling pathway | 3 | 1 | 1 | 1 | 0 | 0 | 0 |
| rno00380 Tryptophan metabolism | 3 | 1 | 1 | 1 | 0 | 0 | 0 |
| rno04730 Long-term depression | 3 | 1 | 1 | 1 | 0 | 0 | 0 |
| rno00410 beta-Alanine metabolism | 3 | 1 | 1 | 1 | 0 | 0 | 0 |
| rno04110 Cell cycle | 3 | 1 | 1 | 1 | 0 | 0 | 0 |
| rno04114 Oocyte meiosis | 3 | 1 | 1 | 1 | 0 | 0 | 0 |
| rno04062 Chemokine signaling pathway | 3 | 1 | 1 | 1 | 0 | 0 | 0 |
| rno04722 Neurotrophin signaling pathway | 3 | 1 | 1 | 1 | 0 | 0 | 0 |
| rno04530 Tight junction | 3 | 1 | 1 | 1 | 0 | 0 | 0 |
| rno04621 NOD-like receptor signaling pathway | 3 | 1 | 1 | 1 | 0 | 0 | 0 |
| rno00071 Fatty acid metabolism | 3 | 1 | 1 | 1 | 0 | 0 | 0 |
| rno04520 Adherens junction | 3 | 1 | 1 | 1 | 0 | 0 | 0 |
| rno04020 Calcium signaling pathway | 3 | 1 | 1 | 1 | 0 | 0 | 0 |
| rno00310 Lysine degradation | 3 | 1 | 1 | 1 | 0 | 0 | 0 |
| rno00640 Propanoate metabolism | 3 | 1 | 1 | 1 | 0 | 0 | 0 |
| rno00903 Limonene and pinene degradation | 3 | 1 | 1 | 1 | 0 | 0 | 0 |
| rno05142 Chagas disease | 3 | 1 | 1 | 1 | 0 | 0 | 0 |
| rno05200 Pathways in cancer | 3 | 1 | 1 | 1 | 0 | 0 | 0 |
| rno01100 Metabolic pathways | 3 | 1 | 1 | 1 | 0 | 0 | 0 |
| rno04210 Apoptosis | 3 | 1 | 1 | 1 | 0 | 0 | 0 |
| rno04910 Insulin signaling pathway | 3 | 1 | 1 | 1 | 0 | 0 | 0 |
| rno00120 Primary bile acid biosynthesis | 2 | 0 | 1 | 1 | 0 | 0 | 0 |
| rno04670 Leukocyte transendothelial migration | 2 | 0 | 1 | 1 | 0 | 0 | 0 |
| rno04966 Collecting duct acid secretion | 2 | 0 | 1 | 1 | 0 | 0 | 0 |
| rno00520 Amino sugar and nucleotide sugar metabolism | 2 | 0 | 1 | 1 | 0 | 0 | 0 |
| rno04146 Peroxisome | 2 | 0 | 1 | 1 | 0 | 0 | 0 |
| rno05215 Prostate cancer | 2 | 1 | 1 | 0 | 0 | 0 | 0 |
| rno05100 Bacterial invasion of epithelial cells | 2 | 0 | 1 | 1 | 0 | 0 | 0 |
| rno05219 Bladder cancer | 2 | 0 | 1 | 1 | 0 | 0 | 0 |
| rno04060 Cytokine-cytokine receptor interaction | 2 | 0 | 1 | 1 | 0 | 0 | 0 |
| rno02010 ABC transporters | 2 | 0 | 1 | 1 | 0 | 0 | 0 |
| rno03320 PPAR signaling pathway | 2 | 0 | 1 | 1 | 0 | 0 | 0 |
| rno04540 Gap junction | 2 | 0 | 1 | 1 | 0 | 0 | 0 |
| rno05211 Renal cell carcinoma | 2 | 0 | 1 | 1 | 0 | 0 | 0 |
| rno00650 Butanoate metabolism | 2 | 0 | 1 | 1 | 0 | 0 | 0 |
| rno04144 Endocytosis | 2 | 0 | 1 | 1 | 0 | 0 | 0 |
| rno03030 DNA replication | 2 | 0 | 1 | 1 | 0 | 0 | 0 |
| rno04012 ErbB signaling pathway | 2 | 0 | 1 | 1 | 0 | 0 | 0 |
| rno04130 SNARE interactions in vesicular transport | 2 | 0 | 1 | 1 | 0 | 0 | 0 |
| rno05214 Glioma | 2 | 0 | 1 | 1 | 0 | 0 | 0 |
| rno04810 Regulation of actin cytoskeleton | 2 | 0 | 1 | 1 | 0 | 0 | 0 |
| rno04350 TGF-beta signaling pathway | 2 | 0 | 1 | 1 | 0 | 0 | 0 |
| rno04320 Dorso-ventral axis formation | 2 | 0 | 1 | 1 | 0 | 0 | 0 |
| rno00062 Fatty acid elongation in mitochondria | 2 | 0 | 1 | 1 | 0 | 0 | 0 |
| rno04370 VEGF signaling pathway | 1 | 1 | 0 | 0 | 0 | 0 | 0 |
| rno00100 Steroid biosynthesis | 1 | 1 | 0 | 0 | 0 | 0 | 0 |
| rno00280 Valine, leucine and isoleucine degradation | 1 | 1 | 0 | 0 | 0 | 0 | 0 |
| rno05140 Leishmaniasis | 1 | 1 | 0 | 0 | 0 | 0 | 0 |
| rno00270 Cysteine and methionine metabolism | 1 | 1 | 0 | 0 | 0 | 0 | 0 |
| rno00562 Inositol phosphate metabolism | 1 | 1 | 0 | 0 | 0 | 0 | 0 |
| rno04141 Protein processing in endoplasmic reticulum | 1 | 1 | 0 | 0 | 0 | 0 | 0 |
| rno04950 Maturity onset diabetes of the young | 1 | 1 | 0 | 0 | 0 | 0 | 0 |
| rno05014 Amyotrophic lateral sclerosis (ALS) | 1 | 1 | 0 | 0 | 0 | 0 | 0 |
| rno04310 Wnt signaling pathway | 1 | 1 | 0 | 0 | 0 | 0 | 0 |
| rno00340 Histidine metabolism | 1 | 1 | 0 | 0 | 0 | 0 | 0 |
| rno04920 Adipocytokine signaling pathway | 1 | 1 | 0 | 0 | 0 | 0 | 0 |
| rno04962 Vasopressin-regulated water reabsorption | 1 | 1 | 0 | 0 | 0 | 0 | 0 |
| rno04270 Vascular smooth muscle contraction | 1 | 1 | 0 | 0 | 0 | 0 | 0 |
| rno00330 Arginine and proline metabolism | 1 | 1 | 0 | 0 | 0 | 0 | 0 |
| rno00561 Glycerolipid metabolism | 1 | 1 | 0 | 0 | 0 | 0 | 0 |
| rno05146 Amoebiasis | 1 | 1 | 0 | 0 | 0 | 0 | 0 |
| rno00140 Steroid hormone biosynthesis | 1 | 1 | 0 | 0 | 0 | 0 | 0 |
| rno04720 Long-term potentiation | 1 | 1 | 0 | 0 | 0 | 0 | 0 |
| rno00601 Glycosphingolipid biosynthesis - lacto and neolacto series | 1 | 1 | 0 | 0 | 0 | 0 | 0 |
| rno05410 Hypertrophic cardiomyopathy (HCM) | 1 | 1 | 0 | 0 | 0 | 0 | 0 |
| **Total pathways targeted by each miRNA** | | 47 | 48 | 47 | 0 | 1 | 0 |

B

| Pathways | Targeted by different miRNAs | rno-miR-129 | rno-miR-130a | rno-miR-130b | rno-miR-218b | rno-miR-141 | rno-miR-3588 |
| --- | --- | --- | --- | --- | --- | --- | --- |
| GO:0016043~cellular component organization and biogenesis | 5 | 1 | 1 | 1 | 1 | 0 | 1 |
| GO:0050789~regulation of biological process | 5 | 1 | 1 | 1 | 1 | 0 | 1 |
| GO:0008283~cell proliferation | 5 | 1 | 1 | 1 | 1 | 0 | 1 |
| GO:0065007~biological regulation | 5 | 1 | 1 | 1 | 1 | 0 | 1 |
| GO:0048519~negative regulation of biological process | 5 | 1 | 1 | 1 | 1 | 0 | 1 |
| GO:0006796~phosphate metabolic process | 4 | 1 | 1 | 1 | 0 | 0 | 1 |
| GO:0030154~cell differentiation | 4 | 1 | 1 | 1 | 0 | 0 | 1 |
| GO:0008104~protein localization | 4 | 1 | 1 | 1 | 0 | 0 | 1 |
| GO:0051336~regulation of hydrolase activity | 4 | 1 | 1 | 1 | 0 | 0 | 1 |
| GO:0051179~localization | 4 | 1 | 1 | 1 | 0 | 0 | 1 |
| GO:0007399~nervous system development | 4 | 1 | 1 | 1 | 0 | 0 | 1 |
| GO:0033036~macromolecule localization | 4 | 1 | 1 | 1 | 0 | 0 | 1 |
| GO:0048518~positive regulation of biological process | 4 | 1 | 1 | 1 | 0 | 0 | 1 |
| GO:0007242~intracellular signaling cascade | 4 | 1 | 1 | 1 | 0 | 0 | 1 |
| GO:0043687~post-translational protein modification | 4 | 1 | 1 | 1 | 0 | 0 | 1 |
| GO:0043283~biopolymer metabolic process | 4 | 1 | 1 | 1 | 0 | 0 | 1 |
| GO:0006464~protein modification process | 4 | 1 | 1 | 1 | 0 | 0 | 1 |
| GO:0007275~multicellular organismal development | 4 | 1 | 1 | 1 | 0 | 0 | 1 |
| GO:0006793~phosphorus metabolic process | 4 | 1 | 1 | 1 | 0 | 0 | 1 |
| GO:0007155~cell adhesion | 4 | 1 | 1 | 1 | 0 | 0 | 1 |
| GO:0048731~system development | 4 | 1 | 1 | 1 | 0 | 0 | 1 |
| GO:0051674~localization of cell | 4 | 1 | 1 | 1 | 0 | 0 | 1 |
| GO:0048869~cellular developmental process | 4 | 1 | 1 | 1 | 0 | 0 | 1 |
| GO:0048856~anatomical structure development | 4 | 1 | 1 | 1 | 0 | 0 | 1 |
| GO:0043412~biopolymer modification | 4 | 1 | 1 | 1 | 0 | 0 | 1 |
| GO:0032502~developmental process | 4 | 1 | 1 | 1 | 0 | 0 | 1 |
| GO:0022610~biological adhesion | 4 | 1 | 1 | 1 | 0 | 0 | 1 |
| GO:0043170~macromolecule metabolic process | 3 | 1 | 1 | 1 | 0 | 0 | 0 |
| GO:0030029~actin filament-based process | 3 | 1 | 1 | 1 | 0 | 0 | 0 |
| GO:0007179~transforming growth factor beta receptor signaling pathway | 3 | 1 | 1 | 1 | 0 | 0 | 0 |
| GO:0043086~negative regulation of catalytic activity | 3 | 1 | 1 | 1 | 0 | 0 | 0 |
| GO:0006259~DNA metabolic process | 3 | 1 | 1 | 1 | 0 | 0 | 0 |
| GO:0007243~protein kinase cascade | 3 | 1 | 1 | 1 | 0 | 0 | 0 |
| GO:0019933~cAMP-mediated signaling | 3 | 1 | 1 | 1 | 0 | 0 | 0 |
| GO:0007249~I-kappaB kinase/NF-kappaB cascade | 3 | 1 | 1 | 1 | 0 | 0 | 0 |
| GO:0065009~regulation of a molecular function | 3 | 1 | 1 | 1 | 0 | 0 | 0 |
| GO:0044248~cellular catabolic process | 3 | 1 | 1 | 1 | 0 | 0 | 0 |
| GO:0050801~ion homeostasis | 3 | 1 | 1 | 1 | 0 | 0 | 0 |
| GO:0006260~DNA replication | 3 | 1 | 1 | 1 | 0 | 0 | 0 |
| GO:0055086~nucleobase, nucleoside and nucleotide metabolic process | 3 | 1 | 1 | 1 | 0 | 0 | 0 |
| GO:0044262~cellular carbohydrate metabolic process | 3 | 1 | 1 | 1 | 0 | 0 | 0 |
| GO:0055065~metal ion homeostasis | 3 | 1 | 1 | 1 | 0 | 0 | 0 |
| GO:0007610~behavior | 3 | 1 | 1 | 1 | 0 | 0 | 0 |
| GO:0048771~tissue remodeling | 3 | 1 | 1 | 1 | 0 | 0 | 0 |
| GO:0003013~circulatory system process | 3 | 1 | 1 | 1 | 0 | 0 | 0 |
| GO:0006732~coenzyme metabolic process | 3 | 1 | 1 | 1 | 0 | 0 | 0 |
| GO:0042592~homeostatic process | 3 | 1 | 1 | 1 | 0 | 0 | 0 |
| GO:0016265~death | 3 | 1 | 1 | 1 | 0 | 0 | 0 |
| GO:0044260~cellular macromolecule metabolic process | 3 | 1 | 1 | 1 | 0 | 0 | 0 |
| GO:0050953~sensory perception of light stimulus | 3 | 1 | 1 | 1 | 0 | 0 | 0 |
| GO:0006519~amino acid and derivative metabolic process | 3 | 1 | 1 | 1 | 0 | 0 | 0 |
| GO:0009605~response to external stimulus | 3 | 1 | 1 | 1 | 0 | 0 | 0 |
| GO:0008152~metabolic process | 3 | 1 | 1 | 1 | 0 | 0 | 0 |
| GO:0019752~carboxylic acid metabolic process | 3 | 1 | 1 | 1 | 0 | 0 | 0 |
| GO:0007264~small GTPase mediated signal transduction | 3 | 1 | 1 | 1 | 0 | 0 | 0 |
| GO:0007611~learning and/or memory | 3 | 1 | 1 | 1 | 0 | 0 | 0 |
| GO:0006950~response to stress | 3 | 1 | 1 | 1 | 0 | 0 | 0 |
| GO:0055074~calcium ion homeostasis | 3 | 1 | 1 | 1 | 0 | 0 | 0 |
| GO:0008015~blood circulation | 3 | 1 | 1 | 1 | 0 | 0 | 0 |
| GO:0048878~chemical homeostasis | 3 | 1 | 1 | 1 | 0 | 0 | 0 |
| GO:0031279~regulation of cyclase activity | 3 | 1 | 1 | 1 | 0 | 0 | 0 |
| GO:0042493~response to drug | 3 | 1 | 1 | 1 | 0 | 0 | 0 |
| GO:0042060~wound healing | 3 | 1 | 1 | 1 | 0 | 0 | 0 |
| GO:0007010~cytoskeleton organization and biogenesis | 3 | 1 | 1 | 1 | 0 | 0 | 0 |
| GO:0051704~multi-organism process | 3 | 1 | 1 | 1 | 0 | 0 | 0 |
| GO:0042157~lipoprotein metabolic process | 3 | 1 | 1 | 1 | 0 | 0 | 0 |
| GO:0007267~cell-cell signaling | 3 | 1 | 1 | 1 | 0 | 0 | 0 |
| GO:0044238~primary metabolic process | 3 | 1 | 1 | 1 | 0 | 0 | 0 |
| GO:0044267~cellular protein metabolic process | 3 | 1 | 1 | 1 | 0 | 0 | 0 |
| GO:0019226~transmission of nerve impulse | 3 | 1 | 1 | 1 | 0 | 0 | 0 |
| GO:0055066~di-, tri-valent inorganic cation homeostasis | 3 | 1 | 1 | 1 | 0 | 0 | 0 |
| GO:0000278~mitotic cell cycle | 3 | 1 | 1 | 1 | 0 | 0 | 0 |
| GO:0051301~cell division | 3 | 1 | 1 | 1 | 0 | 0 | 0 |
| GO:0006512~ubiquitin cycle | 3 | 1 | 1 | 1 | 0 | 0 | 0 |
| GO:0009987~cellular process | 3 | 1 | 1 | 1 | 0 | 0 | 0 |
| GO:0009582~detection of abiotic stimulus | 3 | 1 | 1 | 1 | 0 | 0 | 0 |
| GO:0009117~nucleotide metabolic process | 3 | 1 | 1 | 1 | 0 | 0 | 0 |
| GO:0019953~sexual reproduction | 3 | 1 | 1 | 1 | 0 | 0 | 0 |
| GO:0065008~regulation of biological quality | 3 | 1 | 1 | 1 | 0 | 0 | 0 |
| GO:0006979~response to oxidative stress | 3 | 1 | 1 | 1 | 0 | 0 | 0 |
| GO:0009628~response to abiotic stimulus | 3 | 1 | 1 | 1 | 0 | 0 | 0 |
| GO:0016070~RNA metabolic process | 3 | 1 | 1 | 1 | 0 | 0 | 0 |
| GO:0032787~monocarboxylic acid metabolic process | 3 | 1 | 1 | 1 | 0 | 0 | 0 |
| GO:0030036~actin cytoskeleton organization and biogenesis | 3 | 1 | 1 | 1 | 0 | 0 | 0 |
| GO:0009058~biosynthetic process | 3 | 1 | 1 | 1 | 0 | 0 | 0 |
| GO:0016311~dephosphorylation | 3 | 1 | 1 | 1 | 0 | 0 | 0 |
| GO:0016044~membrane organization and biogenesis | 3 | 1 | 1 | 1 | 0 | 0 | 0 |
| GO:0007276~gamete generation | 3 | 1 | 1 | 1 | 0 | 0 | 0 |
| GO:0009611~response to wounding | 3 | 1 | 1 | 1 | 0 | 0 | 0 |
| GO:0019538~protein metabolic process | 3 | 1 | 1 | 1 | 0 | 0 | 0 |
| GO:0032501~multicellular organismal process | 3 | 1 | 1 | 1 | 0 | 0 | 0 |
| GO:0003015~heart process | 3 | 1 | 1 | 1 | 0 | 0 | 0 |
| GO:0044237~cellular metabolic process | 3 | 1 | 1 | 1 | 0 | 0 | 0 |
| GO:0032504~multicellular organism reproduction | 3 | 1 | 1 | 1 | 0 | 0 | 0 |
| GO:0040007~growth | 3 | 1 | 1 | 1 | 0 | 0 | 0 |
| GO:0010467~gene expression | 3 | 1 | 1 | 1 | 0 | 0 | 0 |
| GO:0007049~cell cycle | 3 | 1 | 1 | 1 | 0 | 0 | 0 |
| GO:0006996~organelle organization and biogenesis | 3 | 1 | 1 | 1 | 0 | 0 | 0 |
| GO:0051186~cofactor metabolic process | 3 | 1 | 1 | 1 | 0 | 0 | 0 |
| GO:0006066~alcohol metabolic process | 3 | 1 | 1 | 1 | 0 | 0 | 0 |
| GO:0043085~positive regulation of catalytic activity | 3 | 1 | 1 | 1 | 0 | 0 | 0 |
| GO:0000003~reproduction | 3 | 1 | 1 | 1 | 0 | 0 | 0 |
| GO:0005975~carbohydrate metabolic process | 3 | 1 | 1 | 1 | 0 | 0 | 0 |
| GO:0006807~nitrogen compound metabolic process | 3 | 1 | 1 | 1 | 0 | 0 | 0 |
| GO:0022607~cellular component assembly | 3 | 1 | 1 | 1 | 0 | 0 | 0 |
| GO:0046849~bone remodeling | 3 | 1 | 1 | 1 | 0 | 0 | 0 |
| GO:0051339~regulation of lyase activity | 3 | 1 | 1 | 1 | 0 | 0 | 0 |
| GO:0006139~nucleobase, nucleoside, nucleotide and nucleic acid metabolic process | 3 | 1 | 1 | 1 | 0 | 0 | 0 |
| GO:0010324~membrane invagination | 3 | 1 | 1 | 1 | 0 | 0 | 0 |
| GO:0043285~biopolymer catabolic process | 3 | 1 | 1 | 1 | 0 | 0 | 0 |
| GO:0006944~membrane fusion | 3 | 1 | 1 | 1 | 0 | 0 | 0 |
| GO:0060047~heart contraction | 3 | 1 | 1 | 1 | 0 | 0 | 0 |
| GO:0035295~tube development | 3 | 1 | 1 | 1 | 0 | 0 | 0 |
| GO:0007169~transmembrane receptor protein tyrosine kinase signaling pathway | 3 | 1 | 1 | 1 | 0 | 0 | 0 |
| GO:0007626~locomotory behavior | 3 | 1 | 1 | 1 | 0 | 0 | 0 |
| GO:0006955~immune response | 3 | 1 | 1 | 1 | 0 | 0 | 0 |
| GO:0007167~enzyme linked receptor protein signaling pathway | 3 | 1 | 1 | 1 | 0 | 0 | 0 |
| GO:0007178~transmembrane receptor protein serine/threonine kinase signaling pathway | 3 | 1 | 1 | 1 | 0 | 0 | 0 |
| GO:0002376~immune system process | 3 | 1 | 1 | 1 | 0 | 0 | 0 |
| GO:0032774~RNA biosynthetic process | 3 | 1 | 1 | 1 | 0 | 0 | 0 |
| GO:0055080~cation homeostasis | 3 | 1 | 1 | 1 | 0 | 0 | 0 |
| GO:0007005~mitochondrion organization and biogenesis | 3 | 1 | 1 | 1 | 0 | 0 | 0 |
| GO:0006629~lipid metabolic process | 3 | 1 | 1 | 1 | 0 | 0 | 0 |
| GO:0009416~response to light stimulus | 3 | 1 | 1 | 1 | 0 | 0 | 0 |
| GO:0001775~cell activation | 3 | 1 | 1 | 1 | 0 | 0 | 0 |
| GO:0050790~regulation of catalytic activity | 3 | 1 | 1 | 1 | 0 | 0 | 0 |
| GO:0009056~catabolic process | 3 | 1 | 1 | 1 | 0 | 0 | 0 |
| GO:0006082~organic acid metabolic process | 3 | 1 | 1 | 1 | 0 | 0 | 0 |
| GO:0006952~defense response | 3 | 1 | 1 | 1 | 0 | 0 | 0 |
| GO:0009308~amine metabolic process | 3 | 1 | 1 | 1 | 0 | 0 | 0 |
| GO:0009725~response to hormone stimulus | 3 | 1 | 1 | 1 | 0 | 0 | 0 |
| GO:0009581~detection of external stimulus | 3 | 1 | 1 | 1 | 0 | 0 | 0 |
| GO:0007601~visual perception | 3 | 1 | 1 | 1 | 0 | 0 | 0 |
| GO:0019932~second-messenger-mediated signaling | 3 | 1 | 1 | 1 | 0 | 0 | 0 |
| GO:0009719~response to endogenous stimulus | 3 | 1 | 1 | 1 | 0 | 0 | 0 |
| GO:0045859~regulation of protein kinase activity | 2 | 0 | 1 | 1 | 0 | 0 | 0 |
| GO:0045860~positive regulation of protein kinase activity | 2 | 0 | 1 | 1 | 0 | 0 | 0 |
| GO:0048589~developmental growth | 2 | 0 | 1 | 1 | 0 | 0 | 0 |
| GO:0019935~cyclic-nucleotide-mediated signaling | 2 | 0 | 1 | 1 | 0 | 0 | 0 |
| GO:0016051~carbohydrate biosynthetic process | 2 | 0 | 1 | 1 | 0 | 0 | 0 |
| GO:0007015~actin filament organization | 2 | 0 | 1 | 1 | 0 | 0 | 0 |
| GO:0016310~phosphorylation | 2 | 0 | 1 | 1 | 0 | 0 | 0 |
| GO:0031098~stress-activated protein kinase signaling pathway | 2 | 0 | 1 | 1 | 0 | 0 | 0 |
| GO:0051347~positive regulation of transferase activity | 2 | 0 | 1 | 1 | 0 | 0 | 0 |
| GO:0051338~regulation of transferase activity | 2 | 0 | 1 | 1 | 0 | 0 | 0 |
| GO:0007605~sensory perception of sound | 2 | 0 | 1 | 1 | 0 | 0 | 0 |
| GO:0006790~sulfur metabolic process | 2 | 0 | 1 | 1 | 0 | 0 | 0 |
| GO:0051348~negative regulation of transferase activity | 2 | 0 | 1 | 1 | 0 | 0 | 0 |
| GO:0031589~cell-substrate adhesion | 2 | 0 | 1 | 1 | 0 | 0 | 0 |
| GO:0043549~regulation of kinase activity | 2 | 0 | 1 | 1 | 0 | 0 | 0 |
| GO:0007265~Ras protein signal transduction | 2 | 0 | 1 | 1 | 0 | 0 | 0 |
| GO:0007160~cell-matrix adhesion | 2 | 0 | 1 | 1 | 0 | 0 | 0 |
| GO:0000165~MAPKKK cascade | 2 | 0 | 1 | 1 | 0 | 0 | 0 |
| GO:0050954~sensory perception of mechanical stimulus | 2 | 0 | 1 | 1 | 0 | 0 | 0 |
| GO:0000226~microtubule cytoskeleton organization and biogenesis | 2 | 0 | 1 | 1 | 0 | 0 | 0 |
| GO:0033674~positive regulation of kinase activity | 2 | 0 | 1 | 1 | 0 | 0 | 0 |
| GO:0001666~response to hypoxia | 1 | 1 | 0 | 0 | 0 | 0 | 0 |
| GO:0010038~response to metal ion | 1 | 1 | 0 | 0 | 0 | 0 | 0 |
| GO:0008213~protein amino acid alkylation | 1 | 1 | 0 | 0 | 0 | 0 | 0 |
| GO:0006457~protein folding | 1 | 1 | 0 | 0 | 0 | 0 | 0 |
| GO:0010035~response to inorganic substance | 1 | 1 | 0 | 0 | 0 | 0 | 0 |
| GO:0000302~response to reactive oxygen species | 1 | 1 | 0 | 0 | 0 | 0 | 0 |
| GO:0048511~rhythmic process | 1 | 1 | 0 | 0 | 0 | 0 | 0 |
| GO:0006974~response to DNA damage stimulus | 1 | 1 | 0 | 0 | 0 | 0 | 0 |
| GO:0016071~mRNA metabolic process | 1 | 1 | 0 | 0 | 0 | 0 | 0 |
| GO:0007283~spermatogenesis | 1 | 1 | 0 | 0 | 0 | 0 | 0 |
| GO:0006091~generation of precursor metabolites and energy | 1 | 1 | 0 | 0 | 0 | 0 | 0 |
| GO:0048232~male gamete generation | 1 | 1 | 0 | 0 | 0 | 0 | 0 |
| GO:0009259~ribonucleotide metabolic process | 1 | 0 | 1 | 0 | 0 | 0 | 0 |
| GO:0009266~response to temperature stimulus | 1 | 1 | 0 | 0 | 0 | 0 | 0 |
| GO:0010033~response to organic substance | 1 | 1 | 0 | 0 | 0 | 0 | 0 |
| GO:0048015~phosphoinositide-mediated signaling | 1 | 1 | 0 | 0 | 0 | 0 | 0 |
| GO:0009314~response to radiation | 1 | 1 | 0 | 0 | 0 | 0 | 0 |
| GO:0016337~cell-cell adhesion | 1 | 1 | 0 | 0 | 0 | 0 | 0 |
| GO:0009991~response to extracellular stimulus | 1 | 1 | 0 | 0 | 0 | 0 | 0 |
| GO:0043414~biopolymer methylation | 1 | 1 | 0 | 0 | 0 | 0 | 0 |
| GO:0050817~coagulation | 1 | 1 | 0 | 0 | 0 | 0 | 0 |
| GO:0007631~feeding behavior | 1 | 1 | 0 | 0 | 0 | 0 | 0 |
| GO:0009607~response to biotic stimulus | 1 | 1 | 0 | 0 | 0 | 0 | 0 |
| GO:0031667~response to nutrient levels | 1 | 1 | 0 | 0 | 0 | 0 | 0 |
| GO:0042445~hormone metabolic process | 1 | 1 | 0 | 0 | 0 | 0 | 0 |
| GO:0030518~steroid hormone receptor signaling pathway | 1 | 1 | 0 | 0 | 0 | 0 | 0 |
| GO:0051345~positive regulation of hydrolase activity | 1 | 1 | 0 | 0 | 0 | 0 | 0 |
| GO:0043434~response to peptide hormone stimulus | 1 | 1 | 0 | 0 | 0 | 0 | 0 |
| Different pathways predicted by each miRNA | | 162 | 157 | 156 | 5 | 0 | 27 |

**Supplementary Table 4A**

| Pathway | Number of genes identified in a given pathway | P value | BH |
| --- | --- | --- | --- |
| rno01100 Metabolic pathways | 529 | 1.74E-10 | 1.83E-08 |
| rno04070 Phosphatidylinositol signaling system | 48 | 1.40E-06 | 9.83E-05 |
| rno04020 Calcium signaling pathway | 97 | 1.13E-05 | 0.000457977 |
| rno04310 Wnt signaling pathway | 80 | 1.31E-05 | 0.000457977 |
| rno05212 Pancreatic cancer | 43 | 1.27E-05 | 0.000457977 |
| rno04010 MAPK signaling pathway | 132 | 2.29E-05 | 0.00068687 |
| rno04110 Cell cycle | 67 | 7.95E-05 | 0.001518228 |
| rno04144 Endocytosis | 113 | 6.79E-05 | 0.001518228 |
| rno04520 Adherens junction | 43 | 5.81E-05 | 0.001518228 |
| rno05200 Pathways in cancer | 151 | 7.72E-05 | 0.001518228 |
| rno04210 Apoptosis | 52 | 9.38E-05 | 0.001640669 |
| rno04722 Neurotrophin signaling pathway | 68 | 0.000258653 | 0.003621138 |
| rno04910 Insulin signaling pathway | 68 | 0.000258653 | 0.003621138 |
| rno05215 Prostate cancer | 49 | 0.000237094 | 0.003621138 |
| rno00562 Inositol phosphate metabolism | 35 | 0.000303672 | 0.003985699 |
| rno00380 Tryptophan metabolism | 28 | 0.000347808 | 0.004057765 |
| rno04114 Oocyte meiosis | 60 | 0.000329287 | 0.004057765 |
| rno04730 Long-term depression | 39 | 0.000402023 | 0.004443409 |
| rno00330 Arginine and proline metabolism | 32 | 0.000535648 | 0.005624306 |
| rno04350 TGF-beta signaling pathway | 46 | 0.000586968 | 0.005869683 |
| rno00410 beta-Alanine metabolism | 16 | 0.000783998 | 0.00748362 |
| rno04720 Long-term potentiation | 39 | 0.000893106 | 0.008154443 |
| rno04130 SNARE interactions in vesicular transport | 22 | 0.001070217 | 0.008658842 |
| rno04510 Focal adhesion | 90 | 0.001050412 | 0.008658842 |
| rno05219 Bladder cancer | 23 | 0.001072047 | 0.008658842 |
| rno04062 Chemokine signaling pathway | 85 | 0.001211512 | 0.009422868 |
| rno04670 Leukocyte transendothelial migration | 59 | 0.001502506 | 0.011268795 |
| rno04622 RIG-I-like receptor signaling pathway | 35 | 0.001650128 | 0.011949201 |
| rno04120 Ubiquitin mediated proteolysis | 66 | 0.001928995 | 0.013502962 |
| rno04650 Natural killer cell mediated cytotoxicity | 53 | 0.002325261 | 0.015751766 |
| rno04530 Tight junction | 65 | 0.002631321 | 0.016744768 |
| rno04971 Gastric acid secretion | 39 | 0.0025909 | 0.016744768 |
| rno04962 Vasopressin-regulated water reabsorption | 25 | 0.002791519 | 0.016749114 |
| rno05100 Bacterial invasion of epithelial cells | 38 | 0.00277399 | 0.016749114 |
| rno00270 Cysteine and methionine metabolism | 21 | 0.003215607 | 0.01875771 |
| rno00250 Alanine, aspartate and glutamate metabolism | 19 | 0.003344299 | 0.018981156 |
| rno04666 Fc gamma R-mediated phagocytosis | 46 | 0.003887589 | 0.021484047 |
| rno04141 Protein processing in endoplasmic reticulum | 77 | 0.003999698 | 0.021536836 |
| rno04810 Regulation of actin cytoskeleton | 97 | 0.004310122 | 0.022628143 |
| rno04621 NOD-like receptor signaling pathway | 31 | 0.004437843 | 0.022730418 |
| rno05142 Chagas disease | 51 | 0.004560291 | 0.022801453 |
| rno00120 Primary bile acid biosynthesis | 11 | 0.004979891 | 0.024320399 |
| rno00280 Valine, leucine and isoleucine degradation | 28 | 0.005390324 | 0.024857322 |
| rno00640 Propanoate metabolism | 20 | 0.005444937 | 0.024857322 |
| rno05146 Amoebiasis | 49 | 0.005295962 | 0.024857322 |
| rno05211 Renal cell carcinoma | 37 | 0.00563638 | 0.025183826 |
| rno04966 Collecting duct acid secretion | 17 | 0.005937563 | 0.025976837 |
| rno04920 Adipocytokine signaling pathway | 36 | 0.006063682 | 0.02598721 |
| rno04370 VEGF signaling pathway | 39 | 0.00653546 | 0.027448933 |
| rno00400 Phenylalanine, tyrosine and tryptophan biosynthesis | 5 | 0.007168626 | 0.028950221 |
| rno05220 Chronic myeloid leukemia | 38 | 0.007042359 | 0.028950221 |
| rno04660 T cell receptor signaling pathway | 54 | 0.007482017 | 0.029645728 |
| rno04270 Vascular smooth muscle contraction | 56 | 0.008052719 | 0.031316129 |
| rno04012 ErbB signaling pathway | 43 | 0.008328064 | 0.031798061 |
| rno04662 B cell receptor signaling pathway | 39 | 0.008651585 | 0.031874259 |
| rno04970 Salivary secretion | 39 | 0.008651585 | 0.031874259 |
| rno00071 Fatty acid metabolism | 25 | 0.009501394 | 0.033818522 |
| rno05216 Thyroid cancer | 18 | 0.009498796 | 0.033818522 |
| rno05410 Hypertrophic cardiomyopathy (HCM) | 41 | 0.009699068 | 0.033946739 |
| rno00230 Purine metabolism | 74 | 0.010465341 | 0.035618079 |
| rno00600 Sphingolipid metabolism | 23 | 0.010855034 | 0.035618079 |
| rno04142 Lysosome | 58 | 0.010602754 | 0.035618079 |
| rno04320 Dorso-ventral axis formation | 14 | 0.010814211 | 0.035618079 |
| rno04360 Axon guidance | 60 | 0.011198495 | 0.036179753 |
| rno04912 GnRH signaling pathway | 47 | 0.012683844 | 0.040357687 |
| rno04330 Notch signaling pathway | 25 | 0.013535356 | 0.042186246 |
| rno04540 Gap junction | 43 | 0.013660308 | 0.042186246 |
| rno00350 Tyrosine metabolism | 19 | 0.013988898 | 0.042574908 |
| rno00310 Lysine degradation | 24 | 0.014551376 | 0.04303928 |
| rno02010 ABC transporters | 24 | 0.014551376 | 0.04303928 |
| rno05214 Glioma | 32 | 0.014793645 | 0.043148132 |
| rno00903 Limonene and pinene degradation | 7 | 0.016332501 | 0.046983908 |

**Supplementary Table 4B**

| GOBP | n sig in BP | P value | BH |
| --- | --- | --- | --- |
| GO:0032502~developmental process | 1320 | 4.51E-80 | 1.12E-76 |
| GO:0065007~biological regulation | 1801 | 5.64E-76 | 9.31E-73 |
| GO:0050789~regulation of biological process | 1574 | 1.54E-66 | 1.91E-63 |
| GO:0051179~localization | 1283 | 6.73E-65 | 6.68E-62 |
| GO:0048856~anatomical structure development | 950 | 7.88E-63 | 6.51E-60 |
| GO:0007275~multicellular organismal development | 962 | 4.60E-60 | 3.26E-57 |
| GO:0016043~cellular component organization and biogenesis | 1118 | 7.31E-60 | 4.53E-57 |
| GO:0048731~system development | 816 | 1.12E-55 | 6.16E-53 |
| GO:0030154~cell differentiation | 761 | 5.96E-54 | 2.69E-51 |
| GO:0048869~cellular developmental process | 761 | 5.96E-54 | 2.69E-51 |
| GO:0044238~primary metabolic process | 2652 | 1.96E-46 | 8.11E-44 |
| GO:0044237~cellular metabolic process | 2633 | 4.57E-46 | 1.74E-43 |
| GO:0008152~metabolic process | 2924 | 1.00E-44 | 3.54E-42 |
| GO:0007242~intracellular signaling cascade | 607 | 1.24E-39 | 4.09E-37 |
| GO:0043283~biopolymer metabolic process | 1602 | 3.76E-39 | 1.16E-36 |
| GO:0048519~negative regulation of biological process | 493 | 1.13E-37 | 3.30E-35 |
| GO:0006950~response to stress | 493 | 8.27E-37 | 2.28E-34 |
| GO:0007399~nervous system development | 391 | 3.98E-36 | 1.04E-33 |
| GO:0043170~macromolecule metabolic process | 2224 | 2.19E-33 | 5.44E-31 |
| GO:0048518~positive regulation of biological process | 484 | 3.42E-33 | 8.07E-31 |
| GO:0065008~regulation of biological quality | 418 | 6.08E-32 | 1.37E-29 |
| GO:0008104~protein localization | 328 | 4.38E-30 | 9.44E-28 |
| GO:0008283~cell proliferation | 357 | 7.99E-29 | 1.65E-26 |
| GO:0006139~nucleobase, nucleoside, nucleotide and nucleic acid metabolic process | 1135 | 2.58E-28 | 5.12E-26 |
| GO:0033036~macromolecule localization | 335 | 5.54E-28 | 1.06E-25 |
| GO:0016265~death | 349 | 2.09E-27 | 3.84E-25 |
| GO:0009987~cellular process | 4173 | 2.19E-26 | 3.87E-24 |
| GO:0043412~biopolymer modification | 657 | 2.52E-24 | 4.32E-22 |
| GO:0006464~protein modification process | 635 | 8.00E-24 | 1.32E-21 |
| GO:0009605~response to external stimulus | 320 | 8.41E-23 | 1.34E-20 |
| GO:0065009~regulation of a molecular function | 239 | 8.89E-22 | 1.38E-19 |
| GO:0016070~RNA metabolic process | 789 | 2.03E-21 | 3.05E-19 |
| GO:0006629~lipid metabolic process | 307 | 5.27E-21 | 7.68E-19 |
| GO:0007267~cell-cell signaling | 312 | 1.07E-20 | 1.52E-18 |
| GO:0016044~membrane organization and biogenesis | 148 | 6.23E-20 | 8.58E-18 |
| GO:0043687~post-translational protein modification | 525 | 1.74E-19 | 2.33E-17 |
| GO:0019538~protein metabolic process | 1268 | 3.27E-19 | 4.26E-17 |
| GO:0032774~RNA biosynthetic process | 641 | 2.87E-18 | 3.65E-16 |
| GO:0019226~transmission of nerve impulse | 209 | 2.06E-17 | 2.55E-15 |
| GO:0050790~regulation of catalytic activity | 206 | 3.29E-17 | 3.98E-15 |
| GO:0044260~cellular macromolecule metabolic process | 1201 | 5.44E-17 | 6.33E-15 |
| GO:0006082~organic acid metabolic process | 253 | 5.49E-17 | 6.33E-15 |
| GO:0019752~carboxylic acid metabolic process | 252 | 6.41E-17 | 7.23E-15 |
| GO:0051674~localization of cell | 196 | 7.92E-17 | 8.73E-15 |
| GO:0009611~response to wounding | 213 | 1.14E-16 | 1.23E-14 |
| GO:0006996~organelle organization and biogenesis | 417 | 1.25E-16 | 1.32E-14 |
| GO:0007167~enzyme linked receptor protein signaling pathway | 141 | 3.45E-16 | 3.56E-14 |
| GO:0044267~cellular protein metabolic process | 1173 | 1.17E-15 | 1.18E-13 |
| GO:0006793~phosphorus metabolic process | 396 | 1.51E-15 | 1.47E-13 |
| GO:0006796~phosphate metabolic process | 396 | 1.51E-15 | 1.47E-13 |
| GO:0007243~protein kinase cascade | 164 | 4.15E-15 | 3.96E-13 |
| GO:0040007~growth | 147 | 1.32E-14 | 1.23E-12 |
| GO:0000003~reproduction | 225 | 5.37E-14 | 4.93E-12 |
| GO:0007049~cell cycle | 217 | 6.21E-14 | 5.60E-12 |
| GO:0009056~catabolic process | 287 | 1.39E-13 | 1.23E-11 |
| GO:0042592~homeostatic process | 204 | 1.54E-13 | 1.34E-11 |
| GO:0007610~behavior | 162 | 1.91E-13 | 1.63E-11 |
| GO:0016311~dephosphorylation | 82 | 1.21E-12 | 1.02E-10 |
| GO:0006807~nitrogen compound metabolic process | 205 | 2.03E-12 | 1.68E-10 |
| GO:0010467~gene expression | 982 | 2.28E-12 | 1.86E-10 |
| GO:0007155~cell adhesion | 258 | 4.90E-12 | 3.86E-10 |
| GO:0022610~biological adhesion | 258 | 4.90E-12 | 3.86E-10 |
| GO:0009628~response to abiotic stimulus | 104 | 6.87E-12 | 5.32E-10 |
| GO:0022607~cellular component assembly | 229 | 9.53E-12 | 7.27E-10 |
| GO:0048878~chemical homeostasis | 146 | 1.27E-11 | 9.55E-10 |
| GO:0009719~response to endogenous stimulus | 180 | 3.43E-11 | 2.54E-09 |
| GO:0006979~response to oxidative stress | 67 | 3.84E-11 | 2.80E-09 |
| GO:0009308~amine metabolic process | 190 | 7.41E-11 | 5.32E-09 |
| GO:0007264~small GTPase mediated signal transduction | 174 | 1.48E-10 | 1.00E-08 |
| GO:0006519~amino acid and derivative metabolic process | 169 | 1.47E-10 | 1.00E-08 |
| GO:0001666~response to hypoxia | 60 | 1.48E-10 | 1.00E-08 |
| GO:0006944~membrane fusion | 37 | 1.46E-10 | 1.00E-08 |
| GO:0051704~multi-organism process | 111 | 2.43E-10 | 1.63E-08 |
| GO:0007169~transmembrane receptor protein tyrosine kinase signaling pathway | 91 | 2.76E-10 | 1.82E-08 |
| GO:0002376~immune system process | 285 | 3.16E-10 | 2.06E-08 |
| GO:0007005~mitochondrion organization and biogenesis | 50 | 3.49E-10 | 2.25E-08 |
| GO:0007010~cytoskeleton organization and biogenesis | 203 | 5.71E-10 | 3.63E-08 |
| GO:0051336~regulation of hydrolase activity | 81 | 2.00E-09 | 1.25E-07 |
| GO:0050801~ion homeostasis | 125 | 2.38E-09 | 1.48E-07 |
| GO:0044248~cellular catabolic process | 231 | 2.73E-09 | 1.67E-07 |
| GO:0009725~response to hormone stimulus | 83 | 4.94E-09 | 2.99E-07 |
| GO:0048771~tissue remodeling | 77 | 5.68E-09 | 3.39E-07 |
| GO:0042493~response to drug | 71 | 6.09E-09 | 3.60E-07 |
| GO:0006066~alcohol metabolic process | 150 | 1.64E-08 | 9.60E-07 |
| GO:0016310~phosphorylation | 308 | 2.24E-08 | 1.29E-06 |
| GO:0010324~membrane invagination | 78 | 2.41E-08 | 1.37E-06 |
| GO:0003013~circulatory system process | 87 | 3.45E-08 | 1.92E-06 |
| GO:0008015~blood circulation | 87 | 3.45E-08 | 1.92E-06 |
| GO:0046849~bone remodeling | 70 | 3.93E-08 | 2.17E-06 |
| GO:0042060~wound healing | 82 | 5.51E-08 | 3.00E-06 |
| GO:0055080~cation homeostasis | 98 | 5.64E-08 | 3.04E-06 |
| GO:0000278~mitotic cell cycle | 105 | 6.03E-08 | 3.22E-06 |
| GO:0007249~I-kappaB kinase/NF-kappaB cascade | 49 | 9.39E-08 | 4.95E-06 |
| GO:0030036~actin cytoskeleton organization and biogenesis | 89 | 9.52E-08 | 4.97E-06 |
| GO:0032787~monocarboxylic acid metabolic process | 113 | 1.19E-07 | 6.15E-06 |
| GO:0043086~negative regulation of catalytic activity | 47 | 1.23E-07 | 6.27E-06 |
| GO:0007626~locomotory behavior | 83 | 1.32E-07 | 6.67E-06 |
| GO:0030029~actin filament-based process | 92 | 1.50E-07 | 7.54E-06 |
| GO:0007178~transmembrane receptor protein serine/threonine kinase signaling pathway | 46 | 2.39E-07 | 1.19E-05 |
| GO:0035295~tube development | 84 | 2.99E-07 | 1.46E-05 |
| GO:0009607~response to biotic stimulus | 82 | 3.01E-07 | 1.46E-05 |
| GO:0032504~multicellular organism reproduction | 43 | 3.57E-07 | 1.72E-05 |
| GO:0043085~positive regulation of catalytic activity | 106 | 3.64E-07 | 1.73E-05 |
| GO:0009058~biosynthetic process | 550 | 3.72E-07 | 1.76E-05 |
| GO:0005975~carbohydrate metabolic process | 204 | 4.52E-07 | 2.12E-05 |
| GO:0007283~spermatogenesis | 87 | 4.64E-07 | 2.13E-05 |
| GO:0048232~male gamete generation | 87 | 4.64E-07 | 2.13E-05 |
| GO:0043285~biopolymer catabolic process | 113 | 9.19E-07 | 4.18E-05 |
| GO:0007276~gamete generation | 113 | 1.17E-06 | 5.27E-05 |
| GO:0006952~defense response | 177 | 1.24E-06 | 5.55E-05 |
| GO:0043434~response to peptide hormone stimulus | 32 | 1.28E-06 | 5.67E-05 |
| GO:0010033~response to organic substance | 68 | 1.57E-06 | 6.90E-05 |
| GO:0055086~nucleobase, nucleoside and nucleotide metabolic process | 112 | 1.73E-06 | 7.51E-05 |
| GO:0009117~nucleotide metabolic process | 105 | 1.97E-06 | 8.47E-05 |
| GO:0006732~coenzyme metabolic process | 86 | 2.33E-06 | 9.89E-05 |
| GO:0007611~learning and/or memory | 44 | 2.33E-06 | 9.89E-05 |
| GO:0000165~MAPKKK cascade | 75 | 2.46E-06 | 0.000103498 |
| GO:0051338~regulation of transferase activity | 95 | 2.72E-06 | 0.00011348 |
| GO:0019932~second-messenger-mediated signaling | 114 | 3.18E-06 | 0.000130501 |
| GO:0042157~lipoprotein metabolic process | 42 | 3.16E-06 | 0.000130501 |
| GO:0007179~transforming growth factor beta receptor signaling pathway | 33 | 3.92E-06 | 0.000159498 |
| GO:0006091~generation of precursor metabolites and energy | 230 | 4.41E-06 | 0.000177602 |
| GO:0051186~cofactor metabolic process | 101 | 4.66E-06 | 0.000186488 |
| GO:0019953~sexual reproduction | 127 | 4.81E-06 | 0.000190616 |
| GO:0003015~heart process | 31 | 4.98E-06 | 0.000194524 |
| GO:0060047~heart contraction | 31 | 4.98E-06 | 0.000194524 |
| GO:0055065~metal ion homeostasis | 70 | 5.63E-06 | 0.000218247 |
| GO:0055066~di-, tri-valent inorganic cation homeostasis | 82 | 5.87E-06 | 0.000225709 |
| GO:0007601~visual perception | 60 | 5.97E-06 | 0.000225794 |
| GO:0050953~sensory perception of light stimulus | 60 | 5.97E-06 | 0.000225794 |
| GO:0016051~carbohydrate biosynthetic process | 51 | 9.21E-06 | 0.000345872 |
| GO:0031667~response to nutrient levels | 55 | 9.75E-06 | 0.000363296 |
| GO:0006512~ubiquitin cycle | 119 | 1.03E-05 | 0.000380132 |
| GO:0044262~cellular carbohydrate metabolic process | 147 | 1.04E-05 | 0.000381759 |
| GO:0007613~memory | 21 | 1.10E-05 | 0.000401723 |
| GO:0009266~response to temperature stimulus | 29 | 1.15E-05 | 0.00041473 |
| GO:0043549~regulation of kinase activity | 90 | 1.27E-05 | 0.000454757 |
| GO:0010035~response to inorganic substance | 38 | 1.49E-05 | 0.000531894 |
| GO:0055074~calcium ion homeostasis | 64 | 1.53E-05 | 0.00054055 |
| GO:0016337~cell-cell adhesion | 110 | 1.70E-05 | 0.000596737 |
| GO:0007631~feeding behavior | 33 | 1.98E-05 | 0.000692648 |
| GO:0009314~response to radiation | 44 | 2.01E-05 | 0.000696869 |
| GO:0006259~DNA metabolic process | 228 | 2.10E-05 | 0.000723318 |
| GO:0009991~response to extracellular stimulus | 56 | 2.27E-05 | 0.000776832 |
| GO:0003012~muscle system process | 66 | 3.50E-05 | 0.00118204 |
| GO:0006936~muscle contraction | 66 | 3.50E-05 | 0.00118204 |
| GO:0006955~immune response | 190 | 3.54E-05 | 0.00118469 |
| GO:0051301~cell division | 55 | 3.70E-05 | 0.001232725 |
| GO:0045859~regulation of protein kinase activity | 86 | 3.78E-05 | 0.001241293 |
| GO:0010038~response to metal ion | 35 | 3.78E-05 | 0.001241293 |
| GO:0048511~rhythmic process | 51 | 5.28E-05 | 0.001710289 |
| GO:0006766~vitamin metabolic process | 45 | 5.25E-05 | 0.001710289 |
| GO:0006457~protein folding | 88 | 6.79E-05 | 0.002185031 |
| GO:0006790~sulfur metabolic process | 44 | 8.85E-05 | 0.002831492 |
| GO:0031279~regulation of cyclase activity | 26 | 9.21E-05 | 0.002903305 |
| GO:0051339~regulation of lyase activity | 26 | 9.21E-05 | 0.002903305 |
| GO:0006974~response to DNA damage stimulus | 95 | 0.000102154 | 0.003185403 |
| GO:0051261~protein depolymerization | 20 | 0.000108021 | 0.003347316 |
| GO:0016071~mRNA metabolic process | 82 | 0.000109358 | 0.003367681 |
| GO:0007265~Ras protein signal transduction | 76 | 0.000112712 | 0.003449546 |
| GO:0009057~macromolecule catabolic process | 154 | 0.000137858 | 0.004193263 |
| GO:0040011~locomotion | 40 | 0.000175213 | 0.005297001 |
| GO:0006725~aromatic compound metabolic process | 61 | 0.000176925 | 0.005316336 |
| GO:0048589~developmental growth | 41 | 0.000207942 | 0.00621069 |
| GO:0048659~smooth muscle cell proliferation | 18 | 0.000255872 | 0.007470417 |
| GO:0000302~response to reactive oxygen species | 22 | 0.000279321 | 0.008051584 |
| GO:0051345~positive regulation of hydrolase activity | 35 | 0.000289063 | 0.008284235 |
| GO:0051348~negative regulation of transferase activity | 29 | 0.00037108 | 0.01045349 |
| GO:0051347~positive regulation of transferase activity | 59 | 0.000401807 | 0.011255136 |
| GO:0008286~insulin receptor signaling pathway | 19 | 0.00042266 | 0.011706975 |
| GO:0032535~regulation of cellular component size | 19 | 0.00042266 | 0.011706975 |
| GO:0007015~actin filament organization | 28 | 0.000439528 | 0.012106553 |
| GO:0048545~response to steroid hormone stimulus | 47 | 0.000469262 | 0.012854145 |
| GO:0050817~coagulation | 40 | 0.000472799 | 0.012879878 |
| GO:0033002~muscle cell proliferation | 18 | 0.000480762 | 0.013025223 |
| GO:0006575~amino acid derivative metabolic process | 50 | 0.000490729 | 0.013223001 |
| GO:0031589~cell-substrate adhesion | 41 | 0.000542231 | 0.014453659 |
| GO:0001775~cell activation | 83 | 0.000577325 | 0.015225422 |
| GO:0000226~microtubule cytoskeleton organization and biogenesis | 37 | 0.000576005 | 0.015225422 |
| GO:0009108~coenzyme biosynthetic process | 44 | 0.00058631 | 0.015380554 |
| GO:0019933~cAMP-mediated signaling | 52 | 0.000593381 | 0.015484119 |
| GO:0031109~microtubule polymerization or depolymerization | 15 | 0.000675504 | 0.017353111 |
| GO:0043543~protein amino acid acylation | 15 | 0.000675504 | 0.017353111 |
| GO:0051098~regulation of binding | 15 | 0.000675504 | 0.017353111 |
| GO:0005976~polysaccharide metabolic process | 30 | 0.00068545 | 0.017517852 |
| GO:0032446~protein modification by small protein conjugation | 39 | 0.00076361 | 0.019415272 |
| GO:0006118~electron transport | 165 | 0.000768663 | 0.019444032 |
| GO:0018193~peptidyl-amino acid modification | 47 | 0.000806434 | 0.020295932 |
| GO:0033674~positive regulation of kinase activity | 56 | 0.000821447 | 0.020569358 |
| GO:0009416~response to light stimulus | 31 | 0.00082887 | 0.020618128 |
| GO:0051716~cellular response to stimulus | 18 | 0.000857178 | 0.021143731 |
| GO:0048015~phosphoinositide-mediated signaling | 48 | 0.00088602 | 0.021746965 |
| GO:0032501~multicellular organismal process | 1353 | 0.000904186 | 0.02208352 |
| GO:0001655~urogenital system development | 28 | 0.000970667 | 0.023591017 |
| GO:0007017~microtubule-based process | 85 | 0.001064075 | 0.025735042 |
| GO:0008213~protein amino acid alkylation | 16 | 0.001132156 | 0.02724869 |
| GO:0019935~cyclic-nucleotide-mediated signaling | 61 | 0.001139602 | 0.027295393 |
| GO:0006469~negative regulation of protein kinase activity | 27 | 0.001155807 | 0.027418625 |
| GO:0033673~negative regulation of kinase activity | 27 | 0.001155807 | 0.027418625 |
| GO:0001816~cytokine production | 43 | 0.001204375 | 0.028434722 |
| GO:0030522~intracellular receptor-mediated signaling pathway | 22 | 0.001213938 | 0.028524676 |
| GO:0007160~cell-matrix adhesion | 39 | 0.00136772 | 0.03198658 |
| GO:0043087~regulation of GTPase activity | 30 | 0.001397565 | 0.032531108 |
| GO:0009582~detection of abiotic stimulus | 21 | 0.001437436 | 0.033302832 |
| GO:0007612~learning | 18 | 0.001458819 | 0.033485298 |
| GO:0046165~alcohol biosynthetic process | 18 | 0.001458819 | 0.033485298 |
| GO:0016567~protein ubiquitination | 35 | 0.001519681 | 0.034562294 |
| GO:0006260~DNA replication | 64 | 0.00163701 | 0.036892246 |
| GO:0006997~nuclear organization and biogenesis | 17 | 0.001705444 | 0.0382606 |
| GO:0009100~glycoprotein metabolic process | 54 | 0.001762374 | 0.039359696 |
| GO:0009581~detection of external stimulus | 24 | 0.001956692 | 0.043116791 |
| GO:0003018~vascular process in circulatory system | 26 | 0.00199356 | 0.043734819 |
| GO:0045860~positive regulation of protein kinase activity | 53 | 0.002058377 | 0.044760665 |

**Supplementary Table 5A**

| Pathways | Sum | rno-miR-200c-3p | rno-miR-3065-3p | rno-miR-30c-5p | rno-miR-338-3p | rno-miR-3556b | rno-miR-3596a | rno-miR-3596b | rno-miR-3596c | rno-miR-504 | rno-miR-653-5p |
| --- | --- | --- | --- | --- | --- | --- | --- | --- | --- | --- | --- |
| rno04310 Wnt signaling pathway | 5 | 1 | 0 | 1 | 1 | 0 | 0 | 0 | 1 | 1 | 0 |
| rno04114 Oocyte meiosis | 5 | 1 | 0 | 1 | 1 | 0 | 1 | 0 | 1 | 0 | 0 |
| rno04720 Long-term potentiation | 5 | 1 | 0 | 1 | 1 | 0 | 1 | 0 | 1 | 0 | 0 |
| rno05200 Pathways in cancer | 4 | 1 | 0 | 1 | 1 | 0 | 0 | 0 | 0 | 1 | 0 |
| rno04010 MAPK signaling pathway | 4 | 1 | 1 | 1 | 1 | 0 | 0 | 0 | 0 | 0 | 0 |
| rno04130 SNARE interactions in vesicular transport | 4 | 1 | 1 | 1 | 1 | 0 | 0 | 0 | 0 | 0 | 0 |
| rno04510 Focal adhesion | 4 | 1 | 0 | 1 | 1 | 0 | 0 | 0 | 0 | 1 | 0 |
| rno04110 Cell cycle | 4 | 1 | 0 | 1 | 1 | 0 | 0 | 0 | 1 | 0 | 0 |
| rno05212 Pancreatic cancer | 3 | 0 | 0 | 1 | 1 | 0 | 0 | 0 | 0 | 1 | 0 |
| rno05211 Renal cell carcinoma | 3 | 1 | 0 | 1 | 1 | 0 | 0 | 0 | 0 | 0 | 0 |
| rno04062 Chemokine signaling pathway | 3 | 0 | 0 | 1 | 1 | 0 | 0 | 0 | 0 | 1 | 0 |
| rno04144 Endocytosis | 3 | 1 | 0 | 1 | 1 | 0 | 0 | 0 | 0 | 0 | 0 |
| rno04012 ErbB signaling pathway | 3 | 1 | 0 | 1 | 1 | 0 | 0 | 0 | 0 | 0 | 0 |
| rno05220 Chronic myeloid leukemia | 3 | 0 | 0 | 1 | 1 | 0 | 0 | 0 | 0 | 1 | 0 |
| rno01100 Metabolic pathways | 3 | 1 | 0 | 1 | 1 | 0 | 0 | 0 | 0 | 0 | 0 |
| rno04730 Long-term depression | 3 | 0 | 0 | 0 | 1 | 0 | 1 | 0 | 1 | 0 | 0 |
| rno05219 Bladder cancer | 3 | 0 | 0 | 1 | 1 | 0 | 0 | 0 | 0 | 1 | 0 |
| rno04070 Phosphatidylinositol signaling system | 3 | 0 | 0 | 1 | 1 | 0 | 0 | 0 | 1 | 0 | 0 |
| rno04920 Adipocytokine signaling pathway | 3 | 0 | 0 | 1 | 1 | 0 | 0 | 0 | 0 | 1 | 0 |
| rno04270 Vascular smooth muscle contraction | 3 | 0 | 0 | 0 | 1 | 0 | 1 | 0 | 1 | 0 | 0 |
| rno05142 Chagas disease | 2 | 0 | 0 | 1 | 1 | 0 | 0 | 0 | 0 | 0 | 0 |
| rno04060 Cytokine-cytokine receptor interaction | 2 | 0 | 0 | 1 | 0 | 0 | 0 | 0 | 0 | 1 | 0 |
| rno04666 Fc gamma R-mediated phagocytosis | 2 | 1 | 0 | 0 | 1 | 0 | 0 | 0 | 0 | 0 | 0 |
| rno04722 Neurotrophin signaling pathway | 2 | 0 | 0 | 1 | 1 | 0 | 0 | 0 | 0 | 0 | 0 |
| rno04210 Apoptosis | 2 | 0 | 0 | 1 | 1 | 0 | 0 | 0 | 0 | 0 | 0 |
| rno04360 Axon guidance | 2 | 0 | 1 | 1 | 0 | 0 | 0 | 0 | 0 | 0 | 0 |
| rno04540 Gap junction | 2 | 1 | 0 | 0 | 1 | 0 | 0 | 0 | 0 | 0 | 0 |
| rno04120 Ubiquitin mediated proteolysis | 2 | 0 | 0 | 1 | 0 | 0 | 0 | 0 | 1 | 0 | 0 |
| rno04350 TGF-beta signaling pathway | 2 | 1 | 0 | 1 | 0 | 0 | 0 | 0 | 0 | 0 | 0 |
| rno04660 T cell receptor signaling pathway | 2 | 0 | 0 | 1 | 1 | 0 | 0 | 0 | 0 | 0 | 0 |
| rno04662 B cell receptor signaling pathway | 2 | 0 | 0 | 1 | 1 | 0 | 0 | 0 | 0 | 0 | 0 |
| rno00564 Glycerophospholipid metabolism | 2 | 0 | 0 | 0 | 1 | 0 | 0 | 0 | 0 | 1 | 0 |
| rno00410 beta-Alanine metabolism | 2 | 1 | 0 | 0 | 1 | 0 | 0 | 0 | 0 | 0 | 0 |
| rno04115 p53 signaling pathway | 2 | 1 | 0 | 1 | 0 | 0 | 0 | 0 | 0 | 0 | 0 |
| rno00770 Pantothenate and CoA biosynthesis | 2 | 0 | 0 | 1 | 1 | 0 | 0 | 0 | 0 | 0 | 0 |
| rno04960 Aldosterone-regulated sodium reabsorption | 2 | 0 | 0 | 0 | 0 | 0 | 1 | 0 | 1 | 0 | 0 |
| rno04020 Calcium signaling pathway | 1 | 0 | 0 | 0 | 1 | 0 | 0 | 0 | 0 | 0 | 0 |
| rno04370 VEGF signaling pathway | 1 | 0 | 0 | 0 | 1 | 0 | 0 | 0 | 0 | 0 | 0 |
| rno00100 Steroid biosynthesis | 1 | 0 | 0 | 1 | 0 | 0 | 0 | 0 | 0 | 0 | 0 |
| rno04810 Regulation of actin cytoskeleton | 1 | 0 | 0 | 0 | 1 | 0 | 0 | 0 | 0 | 0 | 0 |
| rno04141 Protein processing in endoplasmic reticulum | 1 | 0 | 0 | 1 | 0 | 0 | 0 | 0 | 0 | 0 | 0 |
| rno04520 Adherens junction | 1 | 0 | 0 | 0 | 0 | 0 | 0 | 0 | 1 | 0 | 0 |
| rno05215 Prostate cancer | 1 | 0 | 0 | 0 | 1 | 0 | 0 | 0 | 0 | 0 | 0 |
| rno04916 Melanogenesis | 1 | 0 | 0 | 0 | 0 | 0 | 0 | 0 | 0 | 1 | 0 |
| rno04080 Neuroactive ligand-receptor interaction | 1 | 0 | 0 | 0 | 1 | 0 | 0 | 0 | 0 | 0 | 0 |
| rno00140 Steroid hormone biosynthesis | 1 | 1 | 0 | 0 | 0 | 0 | 0 | 0 | 0 | 0 | 0 |
| rno04962 Vasopressin-regulated water reabsorption | 1 | 0 | 0 | 0 | 1 | 0 | 0 | 0 | 0 | 0 | 0 |
| rno05214 Glioma | 1 | 0 | 0 | 0 | 0 | 0 | 0 | 0 | 0 | 1 | 0 |
| rno04910 Insulin signaling pathway | 1 | 0 | 0 | 0 | 1 | 0 | 0 | 0 | 0 | 0 | 0 |
| rno00330 Arginine and proline metabolism | 1 | 1 | 0 | 0 | 0 | 0 | 0 | 0 | 0 | 0 | 0 |
| rno04621 NOD-like receptor signaling pathway | 1 | 0 | 0 | 1 | 0 | 0 | 0 | 0 | 0 | 0 | 0 |
| rno05221 Acute myeloid leukemia | 1 | 0 | 0 | 0 | 1 | 0 | 0 | 0 | 0 | 0 | 0 |
| rno00270 Cysteine and methionine metabolism | 1 | 0 | 0 | 1 | 0 | 0 | 0 | 0 | 0 | 0 | 0 |
| rno05213 Endometrial cancer | 1 | 0 | 0 | 0 | 1 | 0 | 0 | 0 | 0 | 0 | 0 |
| rno05210 Colorectal cancer | 1 | 0 | 0 | 0 | 1 | 0 | 0 | 0 | 0 | 0 | 0 |
| rno00561 Glycerolipid metabolism | 1 | 0 | 0 | 0 | 1 | 0 | 0 | 0 | 0 | 0 | 0 |
| rno00053 Ascorbate and aldarate metabolism | 1 | 1 | 0 | 0 | 0 | 0 | 0 | 0 | 0 | 0 | 0 |
| rno04320 Dorso-ventral axis formation | 1 | 0 | 0 | 0 | 1 | 0 | 0 | 0 | 0 | 0 | 0 |
| rno04912 GnRH signaling pathway | 1 | 0 | 0 | 0 | 1 | 0 | 0 | 0 | 0 | 0 | 0 |
| rno00040 Pentose and glucuronate interconversions | 1 | 1 | 0 | 0 | 0 | 0 | 0 | 0 | 0 | 0 | 0 |
| rno00510 N-Glycan biosynthesis | 1 | 0 | 0 | 1 | 0 | 0 | 0 | 0 | 0 | 0 | 0 |
| rno04146 Peroxisome | 1 | 1 | 0 | 0 | 0 | 0 | 0 | 0 | 0 | 0 | 0 |
| rno00130 Ubiquinone and other terpenoid-quinone biosynthesis | 1 | 0 | 0 | 0 | 1 | 0 | 0 | 0 | 0 | 0 | 0 |
| rno00785 Lipoic acid metabolism | 1 | 0 | 0 | 0 | 1 | 0 | 0 | 0 | 0 | 0 | 0 |
| rno04970 Salivary secretion | 1 | 0 | 0 | 0 | 0 | 0 | 0 | 0 | 1 | 0 | 0 |
| rno05216 Thyroid cancer | 1 | 0 | 0 | 0 | 1 | 0 | 0 | 0 | 0 | 0 | 0 |
| rno04664 Fc epsilon RI signaling pathway | 1 | 0 | 0 | 0 | 1 | 0 | 0 | 0 | 0 | 0 | 0 |
| rno00120 Primary bile acid biosynthesis | 1 | 0 | 0 | 1 | 0 | 0 | 0 | 0 | 0 | 0 | 0 |
| rno00620 Pyruvate metabolism | 1 | 1 | 0 | 0 | 0 | 0 | 0 | 0 | 0 | 0 | 0 |
| rno02010 ABC transporters | 1 | 0 | 0 | 0 | 1 | 0 | 0 | 0 | 0 | 0 | 0 |
| rno00533 Glycosaminoglycan biosynthesis - keratan sulfate | 1 | 1 | 0 | 0 | 0 | 0 | 0 | 0 | 0 | 0 | 0 |
| rno03320 PPAR signaling pathway | 1 | 0 | 0 | 0 | 1 | 0 | 0 | 0 | 0 | 0 | 0 |
| rno00471 D-Glutamine and D-glutamate metabolism | 1 | 1 | 0 | 0 | 0 | 0 | 0 | 0 | 0 | 0 | 0 |
| rno05223 Non-small cell lung cancer | 1 | 0 | 0 | 0 | 1 | 0 | 0 | 0 | 0 | 0 | 0 |
| rno04930 Type II diabetes mellitus | 1 | 0 | 0 | 0 | 1 | 0 | 0 | 0 | 0 | 0 | 0 |
| rno00903 Limonene and pinene degradation | 1 | 1 | 0 | 0 | 0 | 0 | 0 | 0 | 0 | 0 | 0 |
| rno00601 Glycosphingolipid biosynthesis - lacto and neolacto series | 1 | 1 | 0 | 0 | 0 | 0 | 0 | 0 | 0 | 0 | 0 |
| rno05410 Hypertrophic cardiomyopathy (HCM) | 1 | 0 | 0 | 0 | 1 | 0 | 0 | 0 | 0 | 0 | 0 |
| Total pathways predicted by each miRNA | | 27 | 3 | 35 | 52 | 0 | 5 | 0 | 11 | 12 | 0 |

Sum: it denotes how many miRNAs are putatively targeting a given pathway. Total pathways predicted by each miRNA: how many pathways are predicted by each miRNA.

1: Predicted to target; 0: not targeted.

**Supplementary Table 5B**

| Pathways | Sum | rno-miR-128-3p | rno-miR-133a-3p | rno-miR-133b-3p | rno-miR-135a-5p | rno-miR-135b-5p | rno-miR-138-5p | rno-miR-151-3p | rno-miR-154-3p | rno-miR-182 | rno-miR-186-5p | rno-miR-190a-3p | rno-miR-2964 | rno-miR-29a-5p | rno-miR-328b-3p | rno-miR-3587 | rno-miR-3596d | rno-miR-378a-3p | rno-miR-466b-1-3p | rno-miR-802-3p | rno-miR-802-5p | rno-miR-96-5p |
| --- | --- | --- | --- | --- | --- | --- | --- | --- | --- | --- | --- | --- | --- | --- | --- | --- | --- | --- | --- | --- | --- | --- |
| rno05200 Pathways in cancer | 11 | 1 | 1 | 1 | 1 | 1 | 1 | 0 | 0 | 1 | 1 | 0 | 0 | 0 | 0 | 0 | 0 | 1 | 0 | 0 | 1 | 1 |
| rno04310 Wnt signaling pathway | 10 | 1 | 1 | 1 | 1 | 1 | 1 | 0 | 0 | 1 | 1 | 0 | 0 | 0 | 0 | 0 | 0 | 1 | 0 | 0 | 1 | 0 |
| rno04010 MAPK signaling pathway | 10 | 1 | 1 | 1 | 1 | 1 | 1 | 0 | 0 | 1 | 1 | 0 | 0 | 0 | 0 | 0 | 0 | 1 | 0 | 0 | 0 | 1 |
| rno05212 Pancreatic cancer | 10 | 1 | 1 | 1 | 1 | 1 | 1 | 0 | 0 | 1 | 1 | 0 | 0 | 0 | 0 | 0 | 0 | 1 | 0 | 0 | 0 | 1 |
| rno05211 Renal cell carcinoma | 10 | 1 | 1 | 1 | 1 | 1 | 1 | 0 | 0 | 1 | 1 | 0 | 0 | 0 | 0 | 0 | 0 | 1 | 0 | 0 | 1 | 0 |
| rno05142 Chagas disease | 10 | 1 | 1 | 1 | 1 | 1 | 1 | 0 | 0 | 1 | 1 | 0 | 0 | 0 | 0 | 0 | 0 | 1 | 0 | 0 | 0 | 1 |
| rno04062 Chemokine signaling pathway | 9 | 1 | 0 | 0 | 1 | 1 | 1 | 0 | 0 | 1 | 1 | 0 | 0 | 0 | 0 | 0 | 0 | 1 | 0 | 0 | 1 | 1 |
| rno04144 Endocytosis | 9 | 1 | 1 | 1 | 0 | 0 | 1 | 0 | 0 | 1 | 1 | 0 | 0 | 0 | 0 | 0 | 0 | 1 | 0 | 0 | 1 | 1 |
| rno04012 ErbB signaling pathway | 9 | 1 | 0 | 0 | 1 | 1 | 1 | 0 | 0 | 1 | 1 | 0 | 0 | 0 | 0 | 0 | 0 | 1 | 0 | 0 | 1 | 1 |
| rno05220 Chronic myeloid leukemia | 9 | 1 | 1 | 1 | 1 | 1 | 0 | 0 | 0 | 0 | 1 | 0 | 0 | 0 | 0 | 0 | 0 | 1 | 0 | 0 | 1 | 1 |
| rno01100 Metabolic pathways | 9 | 1 | 1 | 1 | 1 | 1 | 0 | 0 | 0 | 1 | 1 | 0 | 0 | 0 | 0 | 0 | 0 | 1 | 0 | 0 | 0 | 1 |
| rno04060 Cytokine-cytokine receptor interaction | 9 | 1 | 1 | 1 | 1 | 1 | 0 | 0 | 0 | 1 | 1 | 0 | 0 | 0 | 0 | 0 | 0 | 1 | 0 | 0 | 0 | 1 |
| rno04666 Fc gamma R-mediated phagocytosis | 9 | 1 | 1 | 1 | 1 | 1 | 1 | 0 | 0 | 1 | 1 | 0 | 0 | 0 | 0 | 0 | 0 | 1 | 0 | 0 | 0 | 0 |
| rno04020 Calcium signaling pathway | 9 | 1 | 1 | 1 | 1 | 1 | 1 | 0 | 0 | 1 | 0 | 0 | 0 | 0 | 0 | 0 | 0 | 1 | 0 | 0 | 0 | 1 |
| rno04114 Oocyte meiosis | 8 | 1 | 1 | 1 | 0 | 0 | 0 | 0 | 0 | 1 | 1 | 0 | 0 | 0 | 0 | 0 | 0 | 1 | 0 | 0 | 1 | 1 |
| rno04130 SNARE interactions in vesicular transport | 8 | 0 | 1 | 1 | 1 | 1 | 1 | 0 | 0 | 1 | 0 | 0 | 0 | 0 | 0 | 1 | 0 | 0 | 0 | 0 | 0 | 1 |
| rno04730 Long-term depression | 8 | 1 | 1 | 1 | 0 | 0 | 1 | 0 | 0 | 1 | 1 | 0 | 0 | 0 | 0 | 0 | 0 | 0 | 0 | 0 | 1 | 1 |
| rno05219 Bladder cancer | 8 | 0 | 1 | 1 | 1 | 1 | 1 | 0 | 0 | 1 | 0 | 0 | 0 | 0 | 0 | 0 | 0 | 1 | 0 | 0 | 0 | 1 |
| rno04070 Phosphatidylinositol signaling system | 8 | 1 | 1 | 1 | 1 | 1 | 0 | 0 | 0 | 0 | 1 | 0 | 0 | 0 | 0 | 0 | 0 | 1 | 0 | 0 | 0 | 1 |
| rno04510 Focal adhesion | 7 | 0 | 1 | 1 | 0 | 0 | 1 | 0 | 0 | 1 | 1 | 0 | 0 | 0 | 0 | 0 | 0 | 1 | 0 | 0 | 0 | 1 |
| rno04722 Neurotrophin signaling pathway | 7 | 1 | 0 | 0 | 0 | 0 | 1 | 0 | 0 | 1 | 1 | 0 | 0 | 0 | 0 | 0 | 0 | 1 | 0 | 0 | 1 | 1 |
| rno04210 Apoptosis | 7 | 0 | 1 | 1 | 0 | 0 | 1 | 0 | 0 | 1 | 1 | 0 | 0 | 0 | 0 | 0 | 0 | 1 | 0 | 0 | 0 | 1 |
| rno04370 VEGF signaling pathway | 7 | 1 | 1 | 1 | 1 | 1 | 1 | 0 | 0 | 0 | 0 | 0 | 0 | 0 | 0 | 0 | 0 | 1 | 0 | 0 | 0 | 0 |
| rno00100 Steroid biosynthesis | 7 | 1 | 1 | 1 | 1 | 1 | 0 | 0 | 0 | 1 | 0 | 0 | 0 | 0 | 0 | 0 | 0 | 1 | 0 | 0 | 0 | 0 |
| rno04810 Regulation of actin cytoskeleton | 7 | 0 | 1 | 1 | 1 | 1 | 1 | 0 | 0 | 0 | 1 | 0 | 0 | 0 | 0 | 0 | 0 | 1 | 0 | 0 | 0 | 0 |
| rno04360 Axon guidance | 6 | 0 | 1 | 1 | 1 | 1 | 1 | 0 | 0 | 1 | 0 | 0 | 0 | 0 | 0 | 0 | 0 | 0 | 0 | 0 | 0 | 0 |
| rno04141 Protein processing in endoplasmic reticulum | 6 | 1 | 0 | 0 | 1 | 1 | 0 | 0 | 0 | 1 | 1 | 0 | 0 | 0 | 0 | 0 | 0 | 0 | 0 | 0 | 0 | 1 |
| rno04520 Adherens junction | 6 | 1 | 0 | 0 | 1 | 1 | 0 | 0 | 0 | 0 | 1 | 0 | 0 | 0 | 0 | 0 | 0 | 1 | 0 | 0 | 1 | 0 |
| rno04670 Leukocyte transendothelial migration | 6 | 1 | 1 | 1 | 0 | 0 | 0 | 0 | 0 | 1 | 1 | 0 | 0 | 0 | 0 | 0 | 0 | 0 | 0 | 0 | 1 | 0 |
| rno05140 Leishmaniasis | 6 | 1 | 1 | 1 | 1 | 1 | 0 | 0 | 0 | 0 | 0 | 0 | 0 | 0 | 0 | 0 | 0 | 1 | 0 | 0 | 0 | 0 |
| rno04920 Adipocytokine signaling pathway | 5 | 0 | 1 | 1 | 0 | 0 | 0 | 0 | 0 | 1 | 1 | 0 | 0 | 0 | 0 | 0 | 0 | 0 | 0 | 0 | 0 | 1 |
| rno04540 Gap junction | 5 | 1 | 1 | 1 | 0 | 0 | 0 | 0 | 0 | 0 | 0 | 0 | 0 | 0 | 0 | 0 | 0 | 0 | 0 | 0 | 1 | 1 |
| rno04120 Ubiquitin mediated proteolysis | 5 | 1 | 0 | 0 | 1 | 1 | 0 | 0 | 0 | 0 | 0 | 0 | 0 | 0 | 0 | 0 | 0 | 0 | 0 | 0 | 1 | 1 |
| rno05215 Prostate cancer | 5 | 1 | 0 | 0 | 1 | 1 | 1 | 0 | 0 | 0 | 1 | 0 | 0 | 0 | 0 | 0 | 0 | 0 | 0 | 0 | 0 | 0 |
| rno04916 Melanogenesis | 5 | 1 | 1 | 1 | 0 | 0 | 1 | 0 | 0 | 0 | 1 | 0 | 0 | 0 | 0 | 0 | 0 | 0 | 0 | 0 | 0 | 0 |
| rno04080 Neuroactive ligand-receptor interaction | 5 | 0 | 1 | 1 | 1 | 1 | 0 | 0 | 0 | 0 | 0 | 0 | 0 | 0 | 0 | 0 | 0 | 1 | 0 | 0 | 0 | 0 |
| rno00140 Steroid hormone biosynthesis | 5 | 1 | 0 | 0 | 1 | 1 | 0 | 0 | 0 | 0 | 1 | 0 | 0 | 1 | 0 | 0 | 0 | 0 | 0 | 0 | 0 | 0 |
| rno04720 Long-term potentiation | 4 | 1 | 0 | 0 | 0 | 0 | 0 | 0 | 0 | 1 | 1 | 0 | 0 | 0 | 0 | 0 | 0 | 0 | 0 | 0 | 1 | 0 |
| rno04350 TGF-beta signaling pathway | 4 | 1 | 0 | 0 | 0 | 0 | 0 | 0 | 0 | 0 | 1 | 0 | 0 | 0 | 0 | 0 | 0 | 1 | 0 | 0 | 0 | 1 |
| rno04962 Vasopressin-regulated water reabsorption | 4 | 1 | 1 | 1 | 0 | 0 | 0 | 0 | 0 | 0 | 0 | 0 | 0 | 0 | 0 | 0 | 0 | 0 | 0 | 0 | 0 | 1 |
| rno05214 Glioma | 4 | 1 | 0 | 0 | 0 | 0 | 1 | 0 | 0 | 0 | 1 | 0 | 0 | 0 | 0 | 0 | 0 | 0 | 0 | 0 | 1 | 0 |
| rno04910 Insulin signaling pathway | 4 | 1 | 0 | 0 | 0 | 0 | 0 | 0 | 0 | 0 | 1 | 0 | 0 | 0 | 0 | 0 | 0 | 1 | 0 | 0 | 1 | 0 |
| rno04971 Gastric acid secretion | 4 | 0 | 1 | 1 | 0 | 0 | 0 | 0 | 0 | 0 | 0 | 0 | 0 | 0 | 0 | 0 | 0 | 0 | 0 | 0 | 1 | 1 |
| rno00240 Pyrimidine metabolism | 4 | 0 | 1 | 1 | 1 | 1 | 0 | 0 | 0 | 0 | 0 | 0 | 0 | 0 | 0 | 0 | 0 | 0 | 0 | 0 | 0 | 0 |
| rno05100 Bacterial invasion of epithelial cells | 4 | 0 | 1 | 1 | 0 | 0 | 0 | 0 | 0 | 0 | 1 | 0 | 0 | 0 | 0 | 0 | 0 | 1 | 0 | 0 | 0 | 0 |
| rno04530 Tight junction | 4 | 1 | 0 | 0 | 0 | 0 | 1 | 0 | 0 | 0 | 1 | 0 | 0 | 0 | 0 | 0 | 0 | 0 | 0 | 0 | 1 | 0 |
| rno00230 Purine metabolism | 4 | 1 | 1 | 1 | 0 | 0 | 0 | 0 | 0 | 0 | 1 | 0 | 0 | 0 | 0 | 0 | 0 | 0 | 0 | 0 | 0 | 0 |
| rno04110 Cell cycle | 3 | 1 | 0 | 0 | 0 | 0 | 0 | 0 | 0 | 0 | 1 | 0 | 0 | 0 | 0 | 0 | 0 | 0 | 0 | 0 | 1 | 0 |
| rno04270 Vascular smooth muscle contraction | 3 | 0 | 1 | 1 | 0 | 0 | 1 | 0 | 0 | 0 | 0 | 0 | 0 | 0 | 0 | 0 | 0 | 0 | 0 | 0 | 0 | 0 |
| rno04660 T cell receptor signaling pathway | 3 | 0 | 0 | 0 | 1 | 1 | 0 | 0 | 0 | 0 | 1 | 0 | 0 | 0 | 0 | 0 | 0 | 0 | 0 | 0 | 0 | 0 |
| rno04662 B cell receptor signaling pathway | 3 | 1 | 0 | 0 | 0 | 0 | 1 | 0 | 0 | 0 | 0 | 0 | 0 | 0 | 0 | 0 | 0 | 1 | 0 | 0 | 0 | 0 |
| rno00564 Glycerophospholipid metabolism | 3 | 1 | 1 | 1 | 0 | 0 | 0 | 0 | 0 | 0 | 0 | 0 | 0 | 0 | 0 | 0 | 0 | 0 | 0 | 0 | 0 | 0 |
| rno00410 beta-Alanine metabolism | 3 | 1 | 0 | 0 | 1 | 1 | 0 | 0 | 0 | 0 | 0 | 0 | 0 | 0 | 0 | 0 | 0 | 0 | 0 | 0 | 0 | 0 |
| rno00330 Arginine and proline metabolism | 3 | 1 | 0 | 0 | 1 | 1 | 0 | 0 | 0 | 0 | 0 | 0 | 0 | 0 | 0 | 0 | 0 | 0 | 0 | 0 | 0 | 0 |
| rno04621 NOD-like receptor signaling pathway | 3 | 1 | 0 | 0 | 0 | 0 | 0 | 0 | 0 | 0 | 1 | 0 | 0 | 0 | 0 | 0 | 0 | 0 | 0 | 0 | 0 | 1 |
| rno05221 Acute myeloid leukemia | 3 | 0 | 0 | 0 | 1 | 1 | 1 | 0 | 0 | 0 | 0 | 0 | 0 | 0 | 0 | 0 | 0 | 0 | 0 | 0 | 0 | 0 |
| rno00270 Cysteine and methionine metabolism | 2 | 1 | 0 | 0 | 0 | 0 | 0 | 0 | 0 | 0 | 1 | 0 | 0 | 0 | 0 | 0 | 0 | 0 | 0 | 0 | 0 | 0 |
| rno05213 Endometrial cancer | 2 | 0 | 0 | 0 | 0 | 0 | 1 | 0 | 0 | 0 | 0 | 0 | 0 | 0 | 0 | 0 | 0 | 1 | 0 | 0 | 0 | 0 |
| rno05210 Colorectal cancer | 2 | 0 | 0 | 0 | 0 | 0 | 1 | 0 | 0 | 0 | 0 | 0 | 0 | 0 | 0 | 0 | 0 | 0 | 0 | 0 | 0 | 1 |
| rno00561 Glycerolipid metabolism | 2 | 0 | 1 | 1 | 0 | 0 | 0 | 0 | 0 | 0 | 0 | 0 | 0 | 0 | 0 | 0 | 0 | 0 | 0 | 0 | 0 | 0 |
| rno00053 Ascorbate and aldarate metabolism | 2 | 1 | 0 | 0 | 0 | 0 | 0 | 0 | 0 | 0 | 0 | 0 | 0 | 1 | 0 | 0 | 0 | 0 | 0 | 0 | 0 | 0 |
| rno04320 Dorso-ventral axis formation | 2 | 1 | 0 | 0 | 0 | 0 | 0 | 0 | 0 | 0 | 0 | 0 | 0 | 0 | 0 | 0 | 0 | 1 | 0 | 0 | 0 | 0 |
| rno04912 GnRH signaling pathway | 2 | 1 | 0 | 0 | 0 | 0 | 0 | 0 | 0 | 0 | 0 | 0 | 0 | 0 | 0 | 0 | 0 | 0 | 0 | 0 | 0 | 1 |
| rno00040 Pentose and glucuronate interconversions | 2 | 1 | 0 | 0 | 0 | 0 | 0 | 0 | 0 | 0 | 0 | 0 | 0 | 1 | 0 | 0 | 0 | 0 | 0 | 0 | 0 | 0 |
| rno00280 Valine, leucine and isoleucine degradation | 2 | 0 | 0 | 0 | 0 | 1 | 0 | 0 | 0 | 1 | 0 | 0 | 0 | 0 | 0 | 0 | 0 | 0 | 0 | 0 | 0 | 0 |
| rno04512 ECM-receptor interaction | 2 | 0 | 1 | 1 | 0 | 0 | 0 | 0 | 0 | 0 | 0 | 0 | 0 | 0 | 0 | 0 | 0 | 0 | 0 | 0 | 0 | 0 |
| rno00562 Inositol phosphate metabolism | 2 | 0 | 0 | 0 | 1 | 0 | 0 | 0 | 0 | 0 | 0 | 0 | 0 | 0 | 0 | 0 | 0 | 0 | 0 | 0 | 0 | 1 |
| rno00500 Starch and sucrose metabolism | 2 | 1 | 0 | 0 | 0 | 0 | 0 | 0 | 0 | 0 | 0 | 0 | 0 | 1 | 0 | 0 | 0 | 0 | 0 | 0 | 0 | 0 |
| rno00514 O-Mannosyl glycan biosynthesis | 2 | 0 | 1 | 1 | 0 | 0 | 0 | 0 | 0 | 0 | 0 | 0 | 0 | 0 | 0 | 0 | 0 | 0 | 0 | 0 | 0 | 0 |
| rno00982 Drug metabolism - cytochrome P450 | 2 | 1 | 0 | 0 | 0 | 0 | 0 | 0 | 0 | 0 | 0 | 0 | 0 | 1 | 0 | 0 | 0 | 0 | 0 | 0 | 0 | 0 |
| rno00980 Metabolism of xenobiotics by cytochrome P450 | 2 | 1 | 0 | 0 | 0 | 0 | 0 | 0 | 0 | 0 | 0 | 0 | 0 | 1 | 0 | 0 | 0 | 0 | 0 | 0 | 0 | 0 |
| rno04115 p53 signaling pathway | 1 | 0 | 0 | 0 | 0 | 0 | 0 | 0 | 0 | 0 | 1 | 0 | 0 | 0 | 0 | 0 | 0 | 0 | 0 | 0 | 0 | 0 |
| rno00770 Pantothenate and CoA biosynthesis | 1 | 0 | 0 | 0 | 0 | 0 | 0 | 0 | 0 | 0 | 1 | 0 | 0 | 0 | 0 | 0 | 0 | 0 | 0 | 0 | 0 | 0 |
| rno00510 N-Glycan biosynthesis | 1 | 1 | 0 | 0 | 0 | 0 | 0 | 0 | 0 | 0 | 0 | 0 | 0 | 0 | 0 | 0 | 0 | 0 | 0 | 0 | 0 | 0 |
| rno04146 Peroxisome | 1 | 1 | 0 | 0 | 0 | 0 | 0 | 0 | 0 | 0 | 0 | 0 | 0 | 0 | 0 | 0 | 0 | 0 | 0 | 0 | 0 | 0 |
| rno00130 Ubiquinone and other terpenoid-quinone biosynthesis | 1 | 0 | 0 | 0 | 0 | 0 | 1 | 0 | 0 | 0 | 0 | 0 | 0 | 0 | 0 | 0 | 0 | 0 | 0 | 0 | 0 | 0 |
| rno00785 Lipoic acid metabolism | 1 | 0 | 0 | 0 | 0 | 0 | 0 | 0 | 0 | 0 | 0 | 0 | 0 | 0 | 0 | 0 | 0 | 1 | 0 | 0 | 0 | 0 |
| rno00860 Porphyrin and chlorophyll metabolism | 1 | 0 | 0 | 0 | 0 | 0 | 0 | 0 | 0 | 0 | 0 | 0 | 0 | 1 | 0 | 0 | 0 | 0 | 0 | 0 | 0 | 0 |
| rno00983 Drug metabolism - other enzymes | 1 | 0 | 0 | 0 | 0 | 0 | 0 | 0 | 0 | 0 | 0 | 0 | 0 | 1 | 0 | 0 | 0 | 0 | 0 | 0 | 0 | 0 |
| rno04622 RIG-I-like receptor signaling pathway | 1 | 0 | 0 | 0 | 0 | 0 | 1 | 0 | 0 | 0 | 0 | 0 | 0 | 0 | 0 | 0 | 0 | 0 | 0 | 0 | 0 | 0 |
| rno04966 Collecting duct acid secretion | 1 | 1 | 0 | 0 | 0 | 0 | 0 | 0 | 0 | 0 | 0 | 0 | 0 | 0 | 0 | 0 | 0 | 0 | 0 | 0 | 0 | 0 |
| rno00520 Amino sugar and nucleotide sugar metabolism | 1 | 0 | 0 | 0 | 0 | 0 | 0 | 0 | 0 | 0 | 0 | 0 | 0 | 0 | 0 | 0 | 0 | 1 | 0 | 0 | 0 | 0 |
| rno00920 Sulfur metabolism | 1 | 1 | 0 | 0 | 0 | 0 | 0 | 0 | 0 | 0 | 0 | 0 | 0 | 0 | 0 | 0 | 0 | 0 | 0 | 0 | 0 | 0 |
| rno04914 Progesterone-mediated oocyte maturation | 1 | 1 | 0 | 0 | 0 | 0 | 0 | 0 | 0 | 0 | 0 | 0 | 0 | 0 | 0 | 0 | 0 | 0 | 0 | 0 | 0 | 0 |
| rno04620 Toll-like receptor signaling pathway | 1 | 0 | 0 | 0 | 0 | 0 | 0 | 0 | 0 | 0 | 0 | 0 | 0 | 0 | 0 | 0 | 0 | 1 | 0 | 0 | 0 | 0 |
| rno05014 Amyotrophic lateral sclerosis (ALS) | 1 | 0 | 0 | 0 | 0 | 0 | 0 | 0 | 0 | 1 | 0 | 0 | 0 | 0 | 0 | 0 | 0 | 0 | 0 | 0 | 0 | 0 |
| rno04640 Hematopoietic cell lineage | 1 | 0 | 0 | 0 | 0 | 0 | 0 | 0 | 0 | 0 | 1 | 0 | 0 | 0 | 0 | 0 | 0 | 0 | 0 | 0 | 0 | 0 |
| rno00650 Butanoate metabolism | 1 | 1 | 0 | 0 | 0 | 0 | 0 | 0 | 0 | 0 | 0 | 0 | 0 | 0 | 0 | 0 | 0 | 0 | 0 | 0 | 0 | 0 |
| rno00051 Fructose and mannose metabolism | 1 | 0 | 0 | 0 | 0 | 0 | 0 | 0 | 0 | 0 | 0 | 0 | 0 | 0 | 0 | 0 | 0 | 1 | 0 | 0 | 0 | 0 |
| rno05146 Amoebiasis | 1 | 0 | 0 | 0 | 0 | 0 | 0 | 0 | 0 | 0 | 1 | 0 | 0 | 0 | 0 | 0 | 0 | 0 | 0 | 0 | 0 | 0 |
| rno00071 Fatty acid metabolism | 1 | 0 | 0 | 0 | 0 | 1 | 0 | 0 | 0 | 0 | 0 | 0 | 0 | 0 | 0 | 0 | 0 | 0 | 0 | 0 | 0 | 0 |
| rno00830 Retinol metabolism | 1 | 0 | 0 | 0 | 0 | 0 | 0 | 0 | 0 | 0 | 0 | 0 | 0 | 1 | 0 | 0 | 0 | 0 | 0 | 0 | 0 | 0 |
| rno00591 Linoleic acid metabolism | 1 | 1 | 0 | 0 | 0 | 0 | 0 | 0 | 0 | 0 | 0 | 0 | 0 | 0 | 0 | 0 | 0 | 0 | 0 | 0 | 0 | 0 |
| rno05020 Prion diseases | 1 | 0 | 0 | 0 | 0 | 0 | 1 | 0 | 0 | 0 | 0 | 0 | 0 | 0 | 0 | 0 | 0 | 0 | 0 | 0 | 0 | 0 |
| Total pathways predicted to target by each miRNA | | 57 | 39 | 39 | 33 | 34 | 32 | 0 | 0 | 28 | 42 | 0 | 0 | 9 | 0 | 1 | 0 | 36 | 0 | 0 | 20 | 30 |

Sum: it denotes how many miRNAs are putatively targeting a given pathway. Total pathways predicted by each miRNA: how many pathways are predicted by each miRNA.

1: Predicted to target; 0: not targeted.

**Supplementary Table 5C**

| GOBP | Sum | rno-miR-200c-3p | rno-miR-3065-3p | rno-miR-30c-5p | rno-miR-338-3p | rno-miR-3556b | rno-miR-3596a | rno-miR-3596b | rno-miR-3596c | rno-miR-504 | rno-miR-653-5p |
| --- | --- | --- | --- | --- | --- | --- | --- | --- | --- | --- | --- |
| GO:0065007~biological regulation | 8 | 1 | 1 | 1 | 1 | 0 | 1 | 0 | 1 | 1 | 1 |
| GO:0030154~cell differentiation | 8 | 1 | 1 | 1 | 1 | 0 | 1 | 0 | 1 | 1 | 1 |
| GO:0007399~nervous system development | 8 | 1 | 1 | 1 | 1 | 0 | 1 | 0 | 1 | 1 | 1 |
| GO:0048731~system development | 8 | 1 | 1 | 1 | 1 | 0 | 1 | 0 | 1 | 1 | 1 |
| GO:0048869~cellular developmental process | 8 | 1 | 1 | 1 | 1 | 0 | 1 | 0 | 1 | 1 | 1 |
| GO:0048856~anatomical structure development | 8 | 1 | 1 | 1 | 1 | 0 | 1 | 0 | 1 | 1 | 1 |
| GO:0032502~developmental process | 8 | 1 | 1 | 1 | 1 | 0 | 1 | 0 | 1 | 1 | 1 |
| GO:0051179~localization | 7 | 1 | 1 | 1 | 1 | 0 | 0 | 0 | 1 | 1 | 1 |
| GO:0048518~positive regulation of biological process | 7 | 1 | 1 | 1 | 1 | 0 | 1 | 0 | 0 | 1 | 1 |
| GO:0050789~regulation of biological process | 7 | 1 | 1 | 1 | 1 | 0 | 0 | 0 | 1 | 1 | 1 |
| GO:0007275~multicellular organismal development | 7 | 1 | 1 | 1 | 1 | 0 | 0 | 0 | 1 | 1 | 1 |
| GO:0007155~cell adhesion | 7 | 1 | 1 | 1 | 1 | 0 | 1 | 0 | 0 | 1 | 1 |
| GO:0022610~biological adhesion | 7 | 1 | 1 | 1 | 1 | 0 | 1 | 0 | 0 | 1 | 1 |
| GO:0016337~cell-cell adhesion | 7 | 1 | 1 | 0 | 1 | 0 | 1 | 1 | 0 | 1 | 1 |
| GO:0007242~intracellular signaling cascade | 6 | 1 | 1 | 1 | 1 | 0 | 0 | 0 | 0 | 1 | 1 |
| GO:0016043~cellular component organization and biogenesis | 6 | 1 | 1 | 1 | 1 | 0 | 0 | 0 | 1 | 1 | 0 |
| GO:0048519~negative regulation of biological process | 6 | 1 | 1 | 1 | 1 | 0 | 0 | 0 | 0 | 1 | 1 |
| GO:0007267~cell-cell signaling | 6 | 1 | 1 | 1 | 1 | 0 | 0 | 0 | 0 | 1 | 1 |
| GO:0043283~biopolymer metabolic process | 6 | 1 | 1 | 1 | 1 | 0 | 1 | 0 | 0 | 0 | 1 |
| GO:0008283~cell proliferation | 5 | 1 | 0 | 1 | 1 | 0 | 1 | 0 | 0 | 0 | 1 |
| GO:0007264~small GTPase mediated signal transduction | 5 | 1 | 0 | 1 | 1 | 0 | 0 | 0 | 0 | 1 | 1 |
| GO:0044238~primary metabolic process | 5 | 1 | 0 | 1 | 1 | 0 | 0 | 0 | 0 | 1 | 1 |
| GO:0044237~cellular metabolic process | 5 | 1 | 0 | 1 | 1 | 0 | 0 | 0 | 0 | 1 | 1 |
| GO:0006464~protein modification process | 5 | 1 | 0 | 1 | 1 | 0 | 1 | 0 | 0 | 0 | 1 |
| GO:0043412~biopolymer modification | 5 | 1 | 0 | 1 | 1 | 0 | 1 | 0 | 0 | 0 | 1 |
| GO:0006950~response to stress | 5 | 1 | 0 | 1 | 1 | 0 | 0 | 0 | 0 | 1 | 1 |
| GO:0065008~regulation of biological quality | 5 | 1 | 0 | 1 | 1 | 0 | 0 | 0 | 0 | 1 | 1 |
| GO:0007243~protein kinase cascade | 5 | 1 | 1 | 1 | 1 | 0 | 0 | 0 | 0 | 1 | 0 |
| GO:0051674~localization of cell | 4 | 1 | 0 | 1 | 1 | 0 | 0 | 0 | 1 | 0 | 0 |
| GO:0008104~protein localization | 4 | 1 | 0 | 1 | 1 | 0 | 1 | 0 | 0 | 0 | 0 |
| GO:0033036~macromolecule localization | 4 | 1 | 0 | 1 | 1 | 0 | 1 | 0 | 0 | 0 | 0 |
| GO:0043170~macromolecule metabolic process | 4 | 1 | 0 | 1 | 1 | 0 | 0 | 0 | 0 | 0 | 1 |
| GO:0006796~phosphate metabolic process | 4 | 1 | 0 | 1 | 1 | 0 | 0 | 0 | 0 | 0 | 1 |
| GO:0008152~metabolic process | 4 | 1 | 0 | 1 | 1 | 0 | 0 | 0 | 0 | 1 | 0 |
| GO:0009987~cellular process | 4 | 1 | 0 | 1 | 1 | 0 | 0 | 0 | 0 | 1 | 0 |
| GO:0043687~post-translational protein modification | 4 | 1 | 0 | 1 | 1 | 0 | 0 | 0 | 0 | 0 | 1 |
| GO:0007049~cell cycle | 4 | 1 | 0 | 1 | 1 | 0 | 0 | 0 | 0 | 0 | 1 |
| GO:0006793~phosphorus metabolic process | 4 | 1 | 0 | 1 | 1 | 0 | 0 | 0 | 0 | 0 | 1 |
| GO:0016310~phosphorylation | 4 | 1 | 0 | 1 | 1 | 0 | 0 | 0 | 0 | 0 | 1 |
| GO:0065009~regulation of a molecular function | 4 | 1 | 1 | 1 | 1 | 0 | 0 | 0 | 0 | 0 | 0 |
| GO:0000278~mitotic cell cycle | 4 | 1 | 0 | 1 | 1 | 0 | 0 | 0 | 1 | 0 | 0 |
| GO:0007167~enzyme linked receptor protein signaling pathway | 4 | 1 | 0 | 1 | 1 | 0 | 0 | 0 | 0 | 1 | 0 |
| GO:0006629~lipid metabolic process | 4 | 1 | 0 | 1 | 1 | 0 | 0 | 0 | 0 | 1 | 0 |
| GO:0007610~behavior | 4 | 1 | 0 | 1 | 1 | 0 | 0 | 0 | 0 | 1 | 0 |
| GO:0016265~death | 4 | 1 | 0 | 1 | 1 | 0 | 0 | 0 | 0 | 0 | 1 |
| GO:0009605~response to external stimulus | 4 | 1 | 0 | 1 | 1 | 0 | 0 | 0 | 0 | 1 | 0 |
| GO:0019752~carboxylic acid metabolic process | 4 | 1 | 0 | 1 | 1 | 0 | 0 | 0 | 0 | 1 | 0 |
| GO:0019226~transmission of nerve impulse | 4 | 1 | 0 | 1 | 1 | 0 | 0 | 0 | 0 | 0 | 1 |
| GO:0009611~response to wounding | 4 | 1 | 0 | 1 | 1 | 0 | 0 | 0 | 0 | 1 | 0 |
| GO:0040007~growth | 4 | 1 | 0 | 1 | 1 | 0 | 0 | 0 | 0 | 1 | 0 |
| GO:0035295~tube development | 4 | 1 | 0 | 1 | 1 | 0 | 0 | 0 | 0 | 0 | 1 |
| GO:0007626~locomotory behavior | 4 | 1 | 0 | 1 | 1 | 0 | 0 | 0 | 0 | 1 | 0 |
| GO:0007169~transmembrane receptor protein tyrosine kinase signaling pathway | 4 | 1 | 0 | 1 | 1 | 0 | 0 | 0 | 0 | 1 | 0 |
| GO:0000165~MAPKKK cascade | 4 | 1 | 0 | 1 | 1 | 0 | 0 | 0 | 0 | 1 | 0 |
| GO:0006082~organic acid metabolic process | 4 | 1 | 0 | 1 | 1 | 0 | 0 | 0 | 0 | 1 | 0 |
| GO:0009719~response to endogenous stimulus | 4 | 1 | 0 | 1 | 1 | 0 | 0 | 0 | 0 | 0 | 1 |
| GO:0032787~monocarboxylic acid metabolic process | 4 | 1 | 0 | 1 | 1 | 0 | 0 | 0 | 0 | 1 | 0 |
| GO:0006139~nucleobase, nucleoside, nucleotide and nucleic acid metabolic process | 3 | 1 | 0 | 1 | 1 | 0 | 0 | 0 | 0 | 0 | 0 |
| GO:0032774~RNA biosynthetic process | 3 | 1 | 0 | 1 | 1 | 0 | 0 | 0 | 0 | 0 | 0 |
| GO:0042592~homeostatic process | 3 | 1 | 0 | 1 | 1 | 0 | 0 | 0 | 0 | 0 | 0 |
| GO:0044260~cellular macromolecule metabolic process | 3 | 1 | 0 | 1 | 1 | 0 | 0 | 0 | 0 | 0 | 0 |
| GO:0044267~cellular protein metabolic process | 3 | 1 | 0 | 1 | 1 | 0 | 0 | 0 | 0 | 0 | 0 |
| GO:0051301~cell division | 3 | 1 | 0 | 1 | 1 | 0 | 0 | 0 | 0 | 0 | 0 |
| GO:0016070~RNA metabolic process | 3 | 1 | 0 | 1 | 1 | 0 | 0 | 0 | 0 | 0 | 0 |
| GO:0016311~dephosphorylation | 3 | 1 | 0 | 1 | 1 | 0 | 0 | 0 | 0 | 0 | 0 |
| GO:0016044~membrane organization and biogenesis | 3 | 1 | 0 | 1 | 1 | 0 | 0 | 0 | 0 | 0 | 0 |
| GO:0019538~protein metabolic process | 3 | 1 | 0 | 1 | 1 | 0 | 0 | 0 | 0 | 0 | 0 |
| GO:0010467~gene expression | 3 | 1 | 0 | 1 | 1 | 0 | 0 | 0 | 0 | 0 | 0 |
| GO:0006996~organelle organization and biogenesis | 3 | 1 | 0 | 1 | 1 | 0 | 0 | 0 | 0 | 0 | 0 |
| GO:0043085~positive regulation of catalytic activity | 3 | 1 | 0 | 1 | 1 | 0 | 0 | 0 | 0 | 0 | 0 |
| GO:0022607~cellular component assembly | 3 | 1 | 0 | 1 | 1 | 0 | 0 | 0 | 0 | 0 | 0 |
| GO:0050790~regulation of catalytic activity | 3 | 1 | 0 | 1 | 1 | 0 | 0 | 0 | 0 | 0 | 0 |
| GO:0050801~ion homeostasis | 3 | 1 | 0 | 1 | 1 | 0 | 0 | 0 | 0 | 0 | 0 |
| GO:0055065~metal ion homeostasis | 3 | 1 | 0 | 1 | 1 | 0 | 0 | 0 | 0 | 0 | 0 |
| GO:0048771~tissue remodeling | 3 | 1 | 0 | 1 | 1 | 0 | 0 | 0 | 0 | 0 | 0 |
| GO:0003013~circulatory system process | 3 | 1 | 0 | 1 | 1 | 0 | 0 | 0 | 0 | 0 | 0 |
| GO:0006519~amino acid and derivative metabolic process | 3 | 1 | 0 | 1 | 1 | 0 | 0 | 0 | 0 | 0 | 0 |
| GO:0055074~calcium ion homeostasis | 3 | 1 | 0 | 1 | 1 | 0 | 0 | 0 | 0 | 0 | 0 |
| GO:0008015~blood circulation | 3 | 1 | 0 | 1 | 1 | 0 | 0 | 0 | 0 | 0 | 0 |
| GO:0048878~chemical homeostasis | 3 | 1 | 0 | 1 | 1 | 0 | 0 | 0 | 0 | 0 | 0 |
| GO:0042493~response to drug | 3 | 1 | 0 | 1 | 1 | 0 | 0 | 0 | 0 | 0 | 0 |
| GO:0051704~multi-organism process | 3 | 1 | 0 | 1 | 1 | 0 | 0 | 0 | 0 | 0 | 0 |
| GO:0007010~cytoskeleton organization and biogenesis | 3 | 1 | 0 | 1 | 1 | 0 | 0 | 0 | 0 | 0 | 0 |
| GO:0009628~response to abiotic stimulus | 3 | 1 | 0 | 1 | 1 | 0 | 0 | 0 | 0 | 0 | 0 |
| GO:0000003~reproduction | 3 | 1 | 0 | 1 | 1 | 0 | 0 | 0 | 0 | 0 | 0 |
| GO:0006807~nitrogen compound metabolic process | 3 | 1 | 0 | 1 | 1 | 0 | 0 | 0 | 0 | 0 | 0 |
| GO:0046849~bone remodeling | 3 | 1 | 0 | 1 | 1 | 0 | 0 | 0 | 0 | 0 | 0 |
| GO:0010324~membrane invagination | 3 | 1 | 0 | 1 | 1 | 0 | 0 | 0 | 0 | 0 | 0 |
| GO:0009308~amine metabolic process | 3 | 1 | 0 | 1 | 1 | 0 | 0 | 0 | 0 | 0 | 0 |
| GO:0055066~di-, tri-valent inorganic cation homeostasis | 3 | 1 | 0 | 1 | 1 | 0 | 0 | 0 | 0 | 0 | 0 |
| GO:0051338~regulation of transferase activity | 3 | 1 | 0 | 1 | 1 | 0 | 0 | 0 | 0 | 0 | 0 |
| GO:0043549~regulation of kinase activity | 3 | 1 | 0 | 1 | 1 | 0 | 0 | 0 | 0 | 0 | 0 |
| GO:0055080~cation homeostasis | 3 | 1 | 0 | 1 | 1 | 0 | 0 | 0 | 0 | 0 | 0 |
| GO:0009100~glycoprotein metabolic process | 3 | 1 | 0 | 1 | 1 | 0 | 0 | 0 | 0 | 0 | 0 |
| GO:0009056~catabolic process | 3 | 1 | 0 | 1 | 1 | 0 | 0 | 0 | 0 | 0 | 0 |
| GO:0018193~peptidyl-amino acid modification | 3 | 1 | 0 | 1 | 1 | 0 | 0 | 0 | 0 | 0 | 0 |
| GO:0044248~cellular catabolic process | 3 | 1 | 0 | 1 | 1 | 0 | 0 | 0 | 0 | 0 | 0 |
| GO:0006944~membrane fusion | 3 | 1 | 0 | 1 | 1 | 0 | 0 | 0 | 0 | 0 | 0 |
| GO:0043285~biopolymer catabolic process | 3 | 1 | 0 | 1 | 1 | 0 | 0 | 0 | 0 | 0 | 0 |
| GO:0007611~learning and/or memory | 3 | 1 | 0 | 1 | 1 | 0 | 0 | 0 | 0 | 0 | 0 |
| GO:0007156~homophilic cell adhesion | 3 | 0 | 0 | 0 | 1 | 0 | 1 | 0 | 0 | 1 | 0 |
| GO:0030029~actin filament-based process | 2 | 1 | 0 | 1 | 0 | 0 | 0 | 0 | 0 | 0 | 0 |
| GO:0045859~regulation of protein kinase activity | 2 | 1 | 0 | 0 | 1 | 0 | 0 | 0 | 0 | 0 | 0 |
| GO:0051336~regulation of hydrolase activity | 2 | 0 | 0 | 1 | 1 | 0 | 0 | 0 | 0 | 0 | 0 |
| GO:0030036~actin cytoskeleton organization and biogenesis | 2 | 1 | 0 | 1 | 0 | 0 | 0 | 0 | 0 | 0 | 0 |
| GO:0006790~sulfur metabolic process | 2 | 1 | 0 | 1 | 0 | 0 | 0 | 0 | 0 | 0 | 0 |
| GO:0007178~transmembrane receptor protein serine/threonine kinase signaling pathway | 2 | 1 | 0 | 1 | 0 | 0 | 0 | 0 | 0 | 0 | 0 |
| GO:0009607~response to biotic stimulus | 2 | 0 | 0 | 1 | 1 | 0 | 0 | 0 | 0 | 0 | 0 |
| GO:0002376~immune system process | 2 | 0 | 0 | 1 | 1 | 0 | 0 | 0 | 0 | 0 | 0 |
| GO:0019932~second-messenger-mediated signaling | 2 | 1 | 0 | 0 | 1 | 0 | 0 | 0 | 0 | 0 | 0 |
| GO:0001666~response to hypoxia | 2 | 1 | 0 | 0 | 1 | 0 | 0 | 0 | 0 | 0 | 0 |
| GO:0010033~response to organic substance | 2 | 0 | 0 | 1 | 1 | 0 | 0 | 0 | 0 | 0 | 0 |
| GO:0006979~response to oxidative stress | 2 | 1 | 0 | 1 | 0 | 0 | 0 | 0 | 0 | 0 | 0 |
| GO:0007005~mitochondrion organization and biogenesis | 2 | 0 | 0 | 1 | 1 | 0 | 0 | 0 | 0 | 0 | 0 |
| GO:0043413~biopolymer glycosylation | 2 | 1 | 0 | 1 | 0 | 0 | 0 | 0 | 0 | 0 | 0 |
| GO:0007179~transforming growth factor beta receptor signaling pathway | 2 | 1 | 0 | 1 | 0 | 0 | 0 | 0 | 0 | 0 | 0 |
| GO:0006259~DNA metabolic process | 2 | 1 | 0 | 1 | 0 | 0 | 0 | 0 | 0 | 0 | 0 |
| GO:0010035~response to inorganic substance | 2 | 1 | 0 | 1 | 0 | 0 | 0 | 0 | 0 | 0 | 0 |
| GO:0045860~positive regulation of protein kinase activity | 2 | 1 | 0 | 0 | 1 | 0 | 0 | 0 | 0 | 0 | 0 |
| GO:0006575~amino acid derivative metabolic process | 2 | 0 | 0 | 1 | 1 | 0 | 0 | 0 | 0 | 0 | 0 |
| GO:0009266~response to temperature stimulus | 2 | 0 | 0 | 1 | 1 | 0 | 0 | 0 | 0 | 0 | 0 |
| GO:0051347~positive regulation of transferase activity | 2 | 1 | 0 | 0 | 1 | 0 | 0 | 0 | 0 | 0 | 0 |
| GO:0007265~Ras protein signal transduction | 2 | 1 | 0 | 0 | 1 | 0 | 0 | 0 | 0 | 0 | 0 |
| GO:0009725~response to hormone stimulus | 2 | 0 | 0 | 1 | 1 | 0 | 0 | 0 | 0 | 0 | 0 |
| GO:0033674~positive regulation of kinase activity | 2 | 1 | 0 | 0 | 1 | 0 | 0 | 0 | 0 | 0 | 0 |
| GO:0010038~response to metal ion | 2 | 1 | 0 | 1 | 0 | 0 | 0 | 0 | 0 | 0 | 0 |
| GO:0042060~wound healing | 2 | 1 | 0 | 0 | 1 | 0 | 0 | 0 | 0 | 0 | 0 |
| GO:0009314~response to radiation | 2 | 1 | 0 | 0 | 1 | 0 | 0 | 0 | 0 | 0 | 0 |
| GO:0009416~response to light stimulus | 2 | 1 | 0 | 0 | 1 | 0 | 0 | 0 | 0 | 0 | 0 |
| GO:0007249~I-kappaB kinase/NF-kappaB cascade | 2 | 0 | 0 | 1 | 1 | 0 | 0 | 0 | 0 | 0 | 0 |
| GO:0055086~nucleobase, nucleoside and nucleotide metabolic process | 2 | 1 | 0 | 0 | 1 | 0 | 0 | 0 | 0 | 0 | 0 |
| GO:0006952~defense response | 2 | 0 | 0 | 1 | 1 | 0 | 0 | 0 | 0 | 0 | 0 |
| GO:0009117~nucleotide metabolic process | 2 | 1 | 0 | 0 | 1 | 0 | 0 | 0 | 0 | 0 | 0 |
| GO:0003015~heart process | 2 | 0 | 0 | 1 | 1 | 0 | 0 | 0 | 0 | 0 | 0 |
| GO:0060047~heart contraction | 2 | 0 | 0 | 1 | 1 | 0 | 0 | 0 | 0 | 0 | 0 |
| GO:0048015~phosphoinositide-mediated signaling | 2 | 0 | 0 | 1 | 1 | 0 | 0 | 0 | 0 | 0 | 0 |
| GO:0018212~peptidyl-tyrosine modification | 2 | 1 | 0 | 0 | 1 | 0 | 0 | 0 | 0 | 0 | 0 |
| GO:0051345~positive regulation of hydrolase activity | 2 | 0 | 0 | 1 | 1 | 0 | 0 | 0 | 0 | 0 | 0 |
| GO:0001816~cytokine production | 2 | 0 | 0 | 1 | 1 | 0 | 0 | 0 | 0 | 0 | 0 |
| GO:0050673~epithelial cell proliferation | 2 | 1 | 0 | 0 | 1 | 0 | 0 | 0 | 0 | 0 | 0 |
| GO:0032501~multicellular organismal process | 1 | 0 | 0 | 0 | 1 | 0 | 0 | 0 | 0 | 0 | 0 |
| GO:0005975~carbohydrate metabolic process | 1 | 0 | 0 | 0 | 1 | 0 | 0 | 0 | 0 | 0 | 0 |
| GO:0001775~cell activation | 1 | 0 | 0 | 0 | 1 | 0 | 0 | 0 | 0 | 0 | 0 |
| GO:0031279~regulation of cyclase activity | 1 | 0 | 0 | 0 | 1 | 0 | 0 | 0 | 0 | 0 | 0 |
| GO:0006066~alcohol metabolic process | 1 | 0 | 0 | 0 | 1 | 0 | 0 | 0 | 0 | 0 | 0 |
| GO:0051339~regulation of lyase activity | 1 | 0 | 0 | 0 | 1 | 0 | 0 | 0 | 0 | 0 | 0 |
| GO:0006725~aromatic compound metabolic process | 1 | 0 | 0 | 0 | 1 | 0 | 0 | 0 | 0 | 0 | 0 |
| GO:0009058~biosynthetic process | 1 | 0 | 0 | 0 | 1 | 0 | 0 | 0 | 0 | 0 | 0 |
| GO:0019933~cAMP-mediated signaling | 1 | 0 | 0 | 0 | 1 | 0 | 0 | 0 | 0 | 0 | 0 |
| GO:0006974~response to DNA damage stimulus | 1 | 0 | 0 | 1 | 0 | 0 | 0 | 0 | 0 | 0 | 0 |
| GO:0048511~rhythmic process | 1 | 0 | 0 | 0 | 1 | 0 | 0 | 0 | 0 | 0 | 0 |
| GO:0042157~lipoprotein metabolic process | 1 | 1 | 0 | 0 | 0 | 0 | 0 | 0 | 0 | 0 | 0 |
| GO:0043086~negative regulation of catalytic activity | 1 | 0 | 0 | 0 | 1 | 0 | 0 | 0 | 0 | 0 | 0 |
| GO:0003018~vascular process in circulatory system | 1 | 0 | 0 | 0 | 1 | 0 | 0 | 0 | 0 | 0 | 0 |
| GO:0006936~muscle contraction | 1 | 0 | 0 | 1 | 0 | 0 | 0 | 0 | 0 | 0 | 0 |
| GO:0007283~spermatogenesis | 1 | 1 | 0 | 0 | 0 | 0 | 0 | 0 | 0 | 0 | 0 |
| GO:0048232~male gamete generation | 1 | 1 | 0 | 0 | 0 | 0 | 0 | 0 | 0 | 0 | 0 |
| GO:0019935~cyclic-nucleotide-mediated signaling | 1 | 0 | 0 | 0 | 1 | 0 | 0 | 0 | 0 | 0 | 0 |
| GO:0003012~muscle system process | 1 | 0 | 0 | 1 | 0 | 0 | 0 | 0 | 0 | 0 | 0 |
| GO:0019953~sexual reproduction | 1 | 1 | 0 | 0 | 0 | 0 | 0 | 0 | 0 | 0 | 0 |
| GO:0009582~detection of abiotic stimulus | 1 | 0 | 0 | 0 | 1 | 0 | 0 | 0 | 0 | 0 | 0 |
| GO:0007276~gamete generation | 1 | 1 | 0 | 0 | 0 | 0 | 0 | 0 | 0 | 0 | 0 |
| GO:0009991~response to extracellular stimulus | 1 | 0 | 0 | 0 | 1 | 0 | 0 | 0 | 0 | 0 | 0 |
| GO:0031667~response to nutrient levels | 1 | 0 | 0 | 0 | 1 | 0 | 0 | 0 | 0 | 0 | 0 |
| GO:0019221~cytokine and chemokine mediated signaling pathway | 1 | 0 | 0 | 1 | 0 | 0 | 0 | 0 | 0 | 0 | 0 |
| GO:0009581~detection of external stimulus | 1 | 0 | 0 | 0 | 1 | 0 | 0 | 0 | 0 | 0 | 0 |
| GO:0050953~sensory perception of light stimulus | 1 | 1 | 0 | 0 | 0 | 0 | 0 | 0 | 0 | 0 | 0 |
| GO:0006512~ubiquitin cycle | 1 | 0 | 0 | 1 | 0 | 0 | 0 | 0 | 0 | 0 | 0 |
| GO:0032504~multicellular organism reproduction | 1 | 0 | 0 | 0 | 1 | 0 | 0 | 0 | 0 | 0 | 0 |
| GO:0007601~visual perception | 1 | 1 | 0 | 0 | 0 | 0 | 0 | 0 | 0 | 0 | 0 |
| GO:0043434~response to peptide hormone stimulus | 1 | 0 | 0 | 0 | 1 | 0 | 0 | 0 | 0 | 0 | 0 |
| GO:0032943~mononuclear cell proliferation | 1 | 0 | 0 | 0 | 1 | 0 | 0 | 0 | 0 | 0 | 0 |
| GO:0009259~ribonucleotide metabolic process | 1 | 1 | 0 | 0 | 0 | 0 | 0 | 0 | 0 | 0 | 0 |
| GO:0032446~protein modification by small protein conjugation | 1 | 0 | 0 | 1 | 0 | 0 | 0 | 0 | 0 | 0 | 0 |
| GO:0005976~polysaccharide metabolic process | 1 | 0 | 0 | 0 | 1 | 0 | 0 | 0 | 0 | 0 | 0 |
| GO:0006260~DNA replication | 1 | 0 | 0 | 1 | 0 | 0 | 0 | 0 | 0 | 0 | 0 |
| GO:0044262~cellular carbohydrate metabolic process | 1 | 0 | 0 | 0 | 1 | 0 | 0 | 0 | 0 | 0 | 0 |
| GO:0008213~protein amino acid alkylation | 1 | 0 | 0 | 1 | 0 | 0 | 0 | 0 | 0 | 0 | 0 |
| GO:0048736~appendage development | 1 | 0 | 0 | 1 | 0 | 0 | 0 | 0 | 0 | 0 | 0 |
| GO:0016053~organic acid biosynthetic process | 1 | 0 | 0 | 0 | 1 | 0 | 0 | 0 | 0 | 0 | 0 |
| GO:0016051~carbohydrate biosynthetic process | 1 | 0 | 0 | 0 | 1 | 0 | 0 | 0 | 0 | 0 | 0 |
| GO:0046394~carboxylic acid biosynthetic process | 1 | 0 | 0 | 0 | 1 | 0 | 0 | 0 | 0 | 0 | 0 |
| GO:0043414~biopolymer methylation | 1 | 0 | 0 | 1 | 0 | 0 | 0 | 0 | 0 | 0 | 0 |
| GO:0043062~extracellular structure organization and biogenesis | 1 | 1 | 0 | 0 | 0 | 0 | 0 | 0 | 0 | 0 | 0 |
| GO:0000910~cytokinesis | 1 | 0 | 0 | 1 | 0 | 0 | 0 | 0 | 0 | 0 | 0 |
| GO:0040011~locomotion | 1 | 1 | 0 | 0 | 0 | 0 | 0 | 0 | 0 | 0 | 0 |
| GO:0007631~feeding behavior | 1 | 0 | 0 | 1 | 0 | 0 | 0 | 0 | 0 | 0 | 0 |
| GO:0042446~hormone biosynthetic process | 1 | 0 | 0 | 0 | 1 | 0 | 0 | 0 | 0 | 0 | 0 |
| GO:0060173~limb development | 1 | 0 | 0 | 1 | 0 | 0 | 0 | 0 | 0 | 0 | 0 |
| GO:0016071~mRNA metabolic process | 1 | 0 | 0 | 1 | 0 | 0 | 0 | 0 | 0 | 0 | 0 |
| GO:0048589~developmental growth | 1 | 1 | 0 | 0 | 0 | 0 | 0 | 0 | 0 | 0 | 0 |
| GO:0021700~developmental maturation | 1 | 0 | 0 | 0 | 1 | 0 | 0 | 0 | 0 | 0 | 0 |
| GO:0030534~adult behavior | 1 | 0 | 0 | 0 | 1 | 0 | 0 | 0 | 0 | 0 | 0 |
| GO:0001655~urogenital system development | 1 | 0 | 0 | 1 | 0 | 0 | 0 | 0 | 0 | 0 | 0 |
| GO:0007612~learning | 1 | 0 | 0 | 0 | 1 | 0 | 0 | 0 | 0 | 0 | 0 |
| GO:0019233~sensory perception of pain | 1 | 0 | 0 | 1 | 0 | 0 | 0 | 0 | 0 | 0 | 0 |
| GO:0014070~response to organic cyclic substance | 1 | 0 | 0 | 0 | 1 | 0 | 0 | 0 | 0 | 0 | 0 |
| GO:0000226~microtubule cytoskeleton organization and biogenesis | 1 | 0 | 0 | 1 | 0 | 0 | 0 | 0 | 0 | 0 | 0 |
| GO:0006730~one-carbon compound metabolic process | 1 | 0 | 0 | 1 | 0 | 0 | 0 | 0 | 0 | 0 | 0 |
| GO:0009150~purine ribonucleotide metabolic process | 1 | 1 | 0 | 0 | 0 | 0 | 0 | 0 | 0 | 0 | 0 |
| GO:0043284~biopolymer biosynthetic process | 1 | 0 | 0 | 0 | 1 | 0 | 0 | 0 | 0 | 0 | 0 |
| GO:0016485~protein processing | 1 | 0 | 0 | 0 | 1 | 0 | 0 | 0 | 0 | 0 | 0 |
| GO:0016567~protein ubiquitination | 1 | 0 | 0 | 1 | 0 | 0 | 0 | 0 | 0 | 0 | 0 |
| GO:0016540~protein autoprocessing | 1 | 0 | 0 | 0 | 1 | 0 | 0 | 0 | 0 | 0 | 0 |
| GO:0043632~modification-dependent macromolecule catabolic process | 1 | 0 | 0 | 1 | 0 | 0 | 0 | 0 | 0 | 0 | 0 |
| GO:0030522~intracellular receptor-mediated signaling pathway | 1 | 0 | 0 | 1 | 0 | 0 | 0 | 0 | 0 | 0 | 0 |
| GO:0006084~acetyl-CoA metabolic process | 1 | 0 | 0 | 0 | 1 | 0 | 0 | 0 | 0 | 0 | 0 |
| GO:0006163~purine nucleotide metabolic process | 1 | 1 | 0 | 0 | 0 | 0 | 0 | 0 | 0 | 0 | 0 |
| Total pathways predicted to target by each miRNA | | 137 | 21 | 145 | 164 | 0 | 18 | 1 | 13 | 39 | 36 |

Sum: it denotes how many miRNAs are putatively targeting a given pathway. Total pathways predicted by each miRNA: how many pathways are predicted by each miRNA.

1: Predicted to target; 0: not targeted.

**Supplementary Table 5D**

| GOBP | Sum | rno-miR-128-3p | rno-miR-133a-3p | rno-miR-133b-3p | rno-miR-135a-5p | rno-miR-135b-5p | rno-miR-138-5p | rno-miR-151-3p | rno-miR-154-3p | rno-miR-182 | rno-miR-186-5p | rno-miR-190a-3p | rno-miR-2964 | rno-miR-29a-5p | rno-miR-328b-3p | rno-miR-3587 | rno-miR-3596d | rno-miR-378a-3p | rno-miR-466b-1-3p | rno-miR-802-3p | rno-miR-802-5p | rno-miR-96-5p |
| --- | --- | --- | --- | --- | --- | --- | --- | --- | --- | --- | --- | --- | --- | --- | --- | --- | --- | --- | --- | --- | --- | --- |
| GO:0065007~biological regulation | 17 | 1 | 1 | 1 | 1 | 1 | 1 | 1 | 0 | 1 | 1 | 0 | 1 | 1 | 1 | 1 | 1 | 1 | 0 | 0 | 1 | 1 |
| GO:0030154~cell differentiation | 16 | 1 | 1 | 1 | 1 | 1 | 1 | 1 | 0 | 1 | 1 | 0 | 0 | 1 | 1 | 1 | 1 | 1 | 0 | 0 | 1 | 1 |
| GO:0007399~nervous system development | 16 | 1 | 1 | 1 | 1 | 1 | 1 | 1 | 0 | 1 | 1 | 0 | 0 | 1 | 1 | 1 | 1 | 1 | 0 | 0 | 1 | 1 |
| GO:0048731~system development | 16 | 1 | 1 | 1 | 1 | 1 | 1 | 1 | 0 | 1 | 1 | 0 | 0 | 1 | 1 | 1 | 1 | 1 | 0 | 0 | 1 | 1 |
| GO:0048869~cellular developmental process | 16 | 1 | 1 | 1 | 1 | 1 | 1 | 1 | 0 | 1 | 1 | 0 | 0 | 1 | 1 | 1 | 1 | 1 | 0 | 0 | 1 | 1 |
| GO:0048856~anatomical structure development | 16 | 1 | 1 | 1 | 1 | 1 | 1 | 1 | 0 | 1 | 1 | 0 | 0 | 1 | 1 | 1 | 1 | 1 | 0 | 0 | 1 | 1 |
| GO:0032502~developmental process | 16 | 1 | 1 | 1 | 1 | 1 | 1 | 1 | 0 | 1 | 1 | 0 | 0 | 1 | 1 | 1 | 1 | 1 | 0 | 0 | 1 | 1 |
| GO:0051179~localization | 16 | 1 | 1 | 1 | 1 | 1 | 1 | 1 | 0 | 1 | 1 | 0 | 0 | 1 | 1 | 1 | 1 | 1 | 0 | 0 | 1 | 1 |
| GO:0048518~positive regulation of biological process | 16 | 1 | 1 | 1 | 1 | 1 | 1 | 1 | 0 | 1 | 1 | 0 | 0 | 1 | 1 | 1 | 1 | 1 | 0 | 0 | 1 | 1 |
| GO:0050789~regulation of biological process | 16 | 1 | 1 | 1 | 1 | 1 | 1 | 1 | 0 | 1 | 1 | 0 | 0 | 1 | 1 | 1 | 1 | 1 | 0 | 0 | 1 | 1 |
| GO:0007275~multicellular organismal development | 16 | 1 | 1 | 1 | 1 | 1 | 1 | 1 | 0 | 1 | 1 | 0 | 0 | 1 | 1 | 1 | 1 | 1 | 0 | 0 | 1 | 1 |
| GO:0007242~intracellular signaling cascade | 16 | 1 | 1 | 1 | 1 | 1 | 1 | 1 | 0 | 1 | 1 | 0 | 1 | 0 | 1 | 1 | 1 | 1 | 0 | 0 | 1 | 1 |
| GO:0051674~localization of cell | 16 | 1 | 1 | 1 | 1 | 1 | 1 | 1 | 0 | 1 | 1 | 0 | 0 | 1 | 1 | 1 | 1 | 1 | 0 | 0 | 1 | 1 |
| GO:0016043~cellular component organization and biogenesis | 15 | 1 | 1 | 1 | 1 | 1 | 1 | 1 | 0 | 1 | 1 | 0 | 0 | 1 | 1 | 1 | 0 | 1 | 0 | 0 | 1 | 1 |
| GO:0048519~negative regulation of biological process | 15 | 1 | 1 | 1 | 1 | 1 | 1 | 1 | 0 | 1 | 1 | 0 | 0 | 1 | 0 | 1 | 1 | 1 | 0 | 0 | 1 | 1 |
| GO:0007267~cell-cell signaling | 14 | 1 | 1 | 1 | 1 | 1 | 1 | 1 | 0 | 1 | 1 | 0 | 0 | 1 | 0 | 1 | 0 | 1 | 0 | 0 | 1 | 1 |
| GO:0008283~cell proliferation | 14 | 1 | 1 | 1 | 1 | 1 | 1 | 1 | 0 | 1 | 1 | 0 | 0 | 1 | 0 | 1 | 0 | 1 | 0 | 0 | 1 | 1 |
| GO:0008104~protein localization | 14 | 1 | 1 | 1 | 1 | 1 | 1 | 1 | 0 | 1 | 1 | 0 | 0 | 1 | 0 | 1 | 0 | 1 | 0 | 0 | 1 | 1 |
| GO:0033036~macromolecule localization | 14 | 1 | 1 | 1 | 1 | 1 | 1 | 1 | 0 | 1 | 1 | 0 | 0 | 1 | 0 | 1 | 0 | 1 | 0 | 0 | 1 | 1 |
| GO:0043283~biopolymer metabolic process | 13 | 1 | 1 | 1 | 1 | 1 | 1 | 0 | 0 | 1 | 1 | 0 | 0 | 0 | 0 | 1 | 1 | 1 | 0 | 0 | 1 | 1 |
| GO:0007264~small GTPase mediated signal transduction | 13 | 1 | 1 | 1 | 1 | 1 | 1 | 0 | 0 | 1 | 1 | 0 | 0 | 0 | 1 | 1 | 0 | 1 | 0 | 0 | 1 | 1 |
| GO:0044238~primary metabolic process | 13 | 1 | 1 | 1 | 1 | 1 | 1 | 0 | 0 | 1 | 1 | 0 | 0 | 0 | 0 | 1 | 1 | 1 | 0 | 0 | 1 | 1 |
| GO:0044237~cellular metabolic process | 13 | 1 | 1 | 1 | 1 | 1 | 1 | 0 | 0 | 1 | 1 | 0 | 0 | 0 | 0 | 1 | 1 | 1 | 0 | 0 | 1 | 1 |
| GO:0043170~macromolecule metabolic process | 13 | 1 | 1 | 1 | 1 | 1 | 1 | 0 | 0 | 1 | 1 | 0 | 0 | 0 | 0 | 1 | 1 | 1 | 0 | 0 | 1 | 1 |
| GO:0006796~phosphate metabolic process | 13 | 1 | 1 | 1 | 1 | 1 | 1 | 0 | 0 | 1 | 1 | 0 | 0 | 0 | 1 | 1 | 0 | 1 | 0 | 0 | 1 | 1 |
| GO:0008152~metabolic process | 13 | 1 | 1 | 1 | 1 | 1 | 1 | 0 | 0 | 1 | 1 | 0 | 0 | 1 | 0 | 1 | 0 | 1 | 0 | 0 | 1 | 1 |
| GO:0009987~cellular process | 13 | 1 | 1 | 1 | 1 | 1 | 1 | 1 | 0 | 1 | 1 | 0 | 0 | 1 | 0 | 0 | 0 | 1 | 0 | 0 | 1 | 1 |
| GO:0043687~post-translational protein modification | 13 | 1 | 1 | 1 | 1 | 1 | 1 | 0 | 0 | 1 | 1 | 0 | 0 | 0 | 1 | 1 | 0 | 1 | 0 | 0 | 1 | 1 |
| GO:0007049~cell cycle | 13 | 1 | 1 | 1 | 1 | 1 | 1 | 0 | 0 | 1 | 1 | 0 | 0 | 0 | 0 | 1 | 1 | 1 | 0 | 0 | 1 | 1 |
| GO:0006793~phosphorus metabolic process | 13 | 1 | 1 | 1 | 1 | 1 | 1 | 0 | 0 | 1 | 1 | 0 | 0 | 0 | 1 | 1 | 0 | 1 | 0 | 0 | 1 | 1 |
| GO:0006464~protein modification process | 12 | 1 | 1 | 1 | 1 | 1 | 1 | 0 | 0 | 1 | 1 | 0 | 0 | 0 | 0 | 1 | 0 | 1 | 0 | 0 | 1 | 1 |
| GO:0043412~biopolymer modification | 12 | 1 | 1 | 1 | 1 | 1 | 1 | 0 | 0 | 1 | 1 | 0 | 0 | 0 | 0 | 1 | 0 | 1 | 0 | 0 | 1 | 1 |
| GO:0016310~phosphorylation | 12 | 1 | 1 | 1 | 1 | 1 | 1 | 0 | 0 | 1 | 1 | 0 | 0 | 0 | 1 | 0 | 0 | 1 | 0 | 0 | 1 | 1 |
| GO:0006139~nucleobase, nucleoside, nucleotide and nucleic acid metabolic process | 12 | 1 | 1 | 1 | 1 | 1 | 1 | 0 | 0 | 1 | 1 | 0 | 0 | 0 | 0 | 0 | 1 | 1 | 0 | 0 | 1 | 1 |
| GO:0032774~RNA biosynthetic process | 12 | 1 | 1 | 1 | 1 | 1 | 1 | 0 | 0 | 1 | 1 | 0 | 0 | 0 | 0 | 0 | 1 | 1 | 0 | 0 | 1 | 1 |
| GO:0007155~cell adhesion | 11 | 1 | 1 | 1 | 1 | 1 | 1 | 1 | 0 | 1 | 1 | 0 | 0 | 0 | 0 | 0 | 0 | 1 | 0 | 0 | 0 | 1 |
| GO:0022610~biological adhesion | 11 | 1 | 1 | 1 | 1 | 1 | 1 | 1 | 0 | 1 | 1 | 0 | 0 | 0 | 0 | 0 | 0 | 1 | 0 | 0 | 0 | 1 |
| GO:0006950~response to stress | 11 | 1 | 1 | 1 | 1 | 1 | 1 | 0 | 0 | 1 | 1 | 0 | 0 | 1 | 0 | 0 | 0 | 1 | 0 | 0 | 0 | 1 |
| GO:0065008~regulation of biological quality | 11 | 1 | 1 | 1 | 1 | 1 | 1 | 1 | 0 | 1 | 1 | 0 | 0 | 0 | 0 | 0 | 0 | 1 | 0 | 0 | 0 | 1 |
| GO:0065009~regulation of a molecular function | 11 | 1 | 1 | 1 | 1 | 1 | 1 | 0 | 0 | 1 | 1 | 0 | 1 | 0 | 0 | 0 | 0 | 1 | 0 | 0 | 0 | 1 |
| GO:0000278~mitotic cell cycle | 11 | 1 | 1 | 1 | 1 | 1 | 1 | 0 | 0 | 1 | 1 | 0 | 0 | 0 | 0 | 0 | 0 | 1 | 0 | 0 | 1 | 1 |
| GO:0007167~enzyme linked receptor protein signaling pathway | 11 | 1 | 1 | 1 | 1 | 1 | 1 | 0 | 0 | 1 | 1 | 0 | 0 | 0 | 0 | 1 | 0 | 1 | 0 | 0 | 0 | 1 |
| GO:0006629~lipid metabolic process | 11 | 1 | 1 | 1 | 1 | 1 | 1 | 1 | 0 | 1 | 1 | 0 | 0 | 0 | 0 | 0 | 0 | 1 | 0 | 0 | 0 | 1 |
| GO:0042592~homeostatic process | 11 | 1 | 1 | 1 | 1 | 1 | 1 | 0 | 0 | 1 | 1 | 0 | 0 | 0 | 0 | 0 | 0 | 1 | 0 | 0 | 1 | 1 |
| GO:0044260~cellular macromolecule metabolic process | 11 | 1 | 1 | 1 | 1 | 1 | 1 | 0 | 0 | 1 | 1 | 0 | 0 | 0 | 0 | 0 | 0 | 1 | 0 | 0 | 1 | 1 |
| GO:0044267~cellular protein metabolic process | 11 | 1 | 1 | 1 | 1 | 1 | 1 | 0 | 0 | 1 | 1 | 0 | 0 | 0 | 0 | 0 | 0 | 1 | 0 | 0 | 1 | 1 |
| GO:0051301~cell division | 11 | 1 | 1 | 1 | 1 | 1 | 1 | 0 | 0 | 1 | 1 | 0 | 0 | 0 | 0 | 0 | 0 | 1 | 0 | 0 | 1 | 1 |
| GO:0016070~RNA metabolic process | 11 | 1 | 1 | 1 | 1 | 1 | 1 | 0 | 0 | 1 | 1 | 0 | 0 | 0 | 0 | 0 | 1 | 1 | 0 | 0 | 0 | 1 |
| GO:0016311~dephosphorylation | 11 | 1 | 1 | 1 | 1 | 1 | 1 | 0 | 0 | 1 | 1 | 0 | 0 | 0 | 0 | 0 | 0 | 1 | 0 | 0 | 1 | 1 |
| GO:0016044~membrane organization and biogenesis | 11 | 1 | 1 | 1 | 1 | 1 | 1 | 0 | 0 | 1 | 1 | 0 | 0 | 0 | 0 | 1 | 0 | 1 | 0 | 0 | 0 | 1 |
| GO:0019538~protein metabolic process | 11 | 1 | 1 | 1 | 1 | 1 | 1 | 0 | 0 | 1 | 1 | 0 | 0 | 0 | 0 | 0 | 0 | 1 | 0 | 0 | 1 | 1 |
| GO:0010467~gene expression | 11 | 1 | 1 | 1 | 1 | 1 | 1 | 0 | 0 | 1 | 1 | 0 | 0 | 0 | 0 | 0 | 1 | 1 | 0 | 0 | 0 | 1 |
| GO:0006996~organelle organization and biogenesis | 11 | 1 | 1 | 1 | 1 | 1 | 1 | 0 | 0 | 1 | 1 | 0 | 0 | 0 | 0 | 0 | 0 | 1 | 0 | 0 | 1 | 1 |
| GO:0043085~positive regulation of catalytic activity | 11 | 1 | 1 | 1 | 1 | 1 | 1 | 0 | 0 | 1 | 1 | 0 | 1 | 0 | 0 | 0 | 0 | 1 | 0 | 0 | 0 | 1 |
| GO:0022607~cellular component assembly | 11 | 1 | 1 | 1 | 1 | 1 | 1 | 0 | 0 | 1 | 1 | 0 | 0 | 0 | 0 | 1 | 0 | 1 | 0 | 0 | 0 | 1 |
| GO:0050790~regulation of catalytic activity | 11 | 1 | 1 | 1 | 1 | 1 | 1 | 0 | 0 | 1 | 1 | 0 | 1 | 0 | 0 | 0 | 0 | 1 | 0 | 0 | 0 | 1 |
| GO:0032501~multicellular organismal process | 11 | 1 | 1 | 1 | 1 | 1 | 1 | 1 | 0 | 1 | 1 | 0 | 0 | 0 | 0 | 0 | 0 | 1 | 0 | 0 | 0 | 1 |
| GO:0007243~protein kinase cascade | 10 | 1 | 1 | 1 | 1 | 1 | 1 | 0 | 0 | 1 | 1 | 0 | 0 | 0 | 0 | 0 | 0 | 1 | 0 | 0 | 0 | 1 |
| GO:0007610~behavior | 10 | 1 | 1 | 1 | 1 | 1 | 1 | 0 | 0 | 1 | 1 | 0 | 0 | 0 | 0 | 0 | 0 | 1 | 0 | 0 | 0 | 1 |
| GO:0016265~death | 10 | 1 | 1 | 1 | 1 | 1 | 1 | 0 | 0 | 1 | 1 | 0 | 0 | 0 | 0 | 0 | 0 | 1 | 0 | 0 | 0 | 1 |
| GO:0009605~response to external stimulus | 10 | 1 | 1 | 1 | 1 | 1 | 1 | 0 | 0 | 1 | 1 | 0 | 0 | 0 | 0 | 0 | 0 | 1 | 0 | 0 | 0 | 1 |
| GO:0019752~carboxylic acid metabolic process | 10 | 1 | 1 | 1 | 1 | 1 | 1 | 0 | 0 | 1 | 1 | 0 | 0 | 0 | 0 | 0 | 0 | 1 | 0 | 0 | 0 | 1 |
| GO:0019226~transmission of nerve impulse | 10 | 1 | 1 | 1 | 1 | 1 | 1 | 0 | 0 | 1 | 1 | 0 | 0 | 0 | 0 | 0 | 0 | 1 | 0 | 0 | 0 | 1 |
| GO:0009611~response to wounding | 10 | 1 | 1 | 1 | 1 | 1 | 1 | 0 | 0 | 1 | 1 | 0 | 0 | 0 | 0 | 0 | 0 | 1 | 0 | 0 | 0 | 1 |
| GO:0040007~growth | 10 | 1 | 1 | 1 | 1 | 1 | 1 | 0 | 0 | 1 | 1 | 0 | 0 | 0 | 0 | 0 | 0 | 1 | 0 | 0 | 0 | 1 |
| GO:0035295~tube development | 10 | 1 | 1 | 1 | 1 | 1 | 1 | 0 | 0 | 1 | 1 | 0 | 0 | 0 | 0 | 0 | 0 | 1 | 0 | 0 | 0 | 1 |
| GO:0007626~locomotory behavior | 10 | 1 | 1 | 1 | 1 | 1 | 1 | 0 | 0 | 1 | 1 | 0 | 0 | 0 | 0 | 0 | 0 | 1 | 0 | 0 | 0 | 1 |
| GO:0007169~transmembrane receptor protein tyrosine kinase signaling pathway | 10 | 1 | 1 | 1 | 1 | 1 | 1 | 0 | 0 | 1 | 1 | 0 | 0 | 0 | 0 | 0 | 0 | 1 | 0 | 0 | 0 | 1 |
| GO:0000165~MAPKKK cascade | 10 | 1 | 1 | 1 | 1 | 1 | 1 | 0 | 0 | 1 | 1 | 0 | 0 | 0 | 0 | 0 | 0 | 1 | 0 | 0 | 0 | 1 |
| GO:0006082~organic acid metabolic process | 10 | 1 | 1 | 1 | 1 | 1 | 1 | 0 | 0 | 1 | 1 | 0 | 0 | 0 | 0 | 0 | 0 | 1 | 0 | 0 | 0 | 1 |
| GO:0009719~response to endogenous stimulus | 10 | 1 | 1 | 1 | 1 | 1 | 1 | 0 | 0 | 1 | 1 | 0 | 0 | 0 | 0 | 0 | 0 | 1 | 0 | 0 | 0 | 1 |
| GO:0050801~ion homeostasis | 10 | 1 | 1 | 1 | 1 | 1 | 1 | 0 | 0 | 1 | 1 | 0 | 0 | 0 | 0 | 0 | 0 | 1 | 0 | 0 | 0 | 1 |
| GO:0055065~metal ion homeostasis | 10 | 1 | 1 | 1 | 1 | 1 | 1 | 0 | 0 | 1 | 1 | 0 | 0 | 0 | 0 | 0 | 0 | 1 | 0 | 0 | 0 | 1 |
| GO:0048771~tissue remodeling | 10 | 1 | 1 | 1 | 1 | 1 | 1 | 0 | 0 | 1 | 1 | 0 | 0 | 0 | 0 | 0 | 0 | 1 | 0 | 0 | 0 | 1 |
| GO:0003013~circulatory system process | 10 | 1 | 1 | 1 | 1 | 1 | 1 | 0 | 0 | 1 | 1 | 0 | 0 | 0 | 0 | 0 | 0 | 1 | 0 | 0 | 0 | 1 |
| GO:0006519~amino acid and derivative metabolic process | 10 | 1 | 1 | 1 | 1 | 1 | 1 | 0 | 0 | 1 | 1 | 0 | 0 | 0 | 0 | 0 | 0 | 1 | 0 | 0 | 0 | 1 |
| GO:0055074~calcium ion homeostasis | 10 | 1 | 1 | 1 | 1 | 1 | 1 | 0 | 0 | 1 | 1 | 0 | 0 | 0 | 0 | 0 | 0 | 1 | 0 | 0 | 0 | 1 |
| GO:0008015~blood circulation | 10 | 1 | 1 | 1 | 1 | 1 | 1 | 0 | 0 | 1 | 1 | 0 | 0 | 0 | 0 | 0 | 0 | 1 | 0 | 0 | 0 | 1 |
| GO:0048878~chemical homeostasis | 10 | 1 | 1 | 1 | 1 | 1 | 1 | 0 | 0 | 1 | 1 | 0 | 0 | 0 | 0 | 0 | 0 | 1 | 0 | 0 | 0 | 1 |
| GO:0042493~response to drug | 10 | 1 | 1 | 1 | 1 | 1 | 1 | 0 | 0 | 1 | 1 | 0 | 0 | 0 | 0 | 0 | 0 | 1 | 0 | 0 | 0 | 1 |
| GO:0051704~multi-organism process | 10 | 1 | 1 | 1 | 1 | 1 | 1 | 0 | 0 | 1 | 1 | 0 | 0 | 0 | 0 | 0 | 0 | 1 | 0 | 0 | 0 | 1 |
| GO:0007010~cytoskeleton organization and biogenesis | 10 | 1 | 1 | 1 | 1 | 1 | 1 | 0 | 0 | 1 | 1 | 0 | 0 | 0 | 0 | 0 | 0 | 1 | 0 | 0 | 0 | 1 |
| GO:0009628~response to abiotic stimulus | 10 | 1 | 1 | 1 | 1 | 1 | 1 | 0 | 0 | 1 | 1 | 0 | 0 | 0 | 0 | 0 | 0 | 1 | 0 | 0 | 0 | 1 |
| GO:0000003~reproduction | 10 | 1 | 1 | 1 | 1 | 1 | 1 | 0 | 0 | 1 | 1 | 0 | 0 | 0 | 0 | 0 | 0 | 1 | 0 | 0 | 0 | 1 |
| GO:0006807~nitrogen compound metabolic process | 10 | 1 | 1 | 1 | 1 | 1 | 1 | 0 | 0 | 1 | 1 | 0 | 0 | 0 | 0 | 0 | 0 | 1 | 0 | 0 | 0 | 1 |
| GO:0046849~bone remodeling | 10 | 1 | 1 | 1 | 1 | 1 | 1 | 0 | 0 | 1 | 1 | 0 | 0 | 0 | 0 | 0 | 0 | 1 | 0 | 0 | 0 | 1 |
| GO:0010324~membrane invagination | 10 | 1 | 1 | 1 | 1 | 1 | 1 | 0 | 0 | 1 | 1 | 0 | 0 | 0 | 0 | 0 | 0 | 1 | 0 | 0 | 0 | 1 |
| GO:0009308~amine metabolic process | 10 | 1 | 1 | 1 | 1 | 1 | 1 | 0 | 0 | 1 | 1 | 0 | 0 | 0 | 0 | 0 | 0 | 1 | 0 | 0 | 0 | 1 |
| GO:0030029~actin filament-based process | 10 | 1 | 1 | 1 | 1 | 1 | 1 | 0 | 0 | 1 | 1 | 0 | 0 | 0 | 0 | 0 | 0 | 1 | 0 | 0 | 0 | 1 |
| GO:0045859~regulation of protein kinase activity | 10 | 1 | 1 | 1 | 1 | 1 | 1 | 0 | 0 | 1 | 1 | 0 | 0 | 0 | 0 | 0 | 0 | 1 | 0 | 0 | 0 | 1 |
| GO:0051336~regulation of hydrolase activity | 10 | 1 | 1 | 1 | 1 | 1 | 1 | 0 | 0 | 1 | 1 | 0 | 0 | 0 | 0 | 0 | 0 | 1 | 0 | 0 | 0 | 1 |
| GO:0030036~actin cytoskeleton organization and biogenesis | 10 | 1 | 1 | 1 | 1 | 1 | 1 | 0 | 0 | 1 | 1 | 0 | 0 | 0 | 0 | 0 | 0 | 1 | 0 | 0 | 0 | 1 |
| GO:0006790~sulfur metabolic process | 10 | 1 | 1 | 1 | 1 | 1 | 1 | 0 | 0 | 1 | 1 | 0 | 0 | 0 | 0 | 0 | 0 | 1 | 0 | 0 | 0 | 1 |
| GO:0007178~transmembrane receptor protein serine/threonine kinase signaling pathway | 10 | 1 | 1 | 1 | 1 | 1 | 1 | 0 | 0 | 1 | 1 | 0 | 0 | 0 | 0 | 0 | 0 | 1 | 0 | 0 | 0 | 1 |
| GO:0009607~response to biotic stimulus | 10 | 1 | 1 | 1 | 1 | 1 | 1 | 0 | 0 | 1 | 1 | 0 | 0 | 0 | 0 | 0 | 0 | 1 | 0 | 0 | 0 | 1 |
| GO:0002376~immune system process | 10 | 1 | 1 | 1 | 1 | 1 | 1 | 0 | 0 | 1 | 1 | 0 | 0 | 0 | 0 | 0 | 0 | 1 | 0 | 0 | 0 | 1 |
| GO:0019932~second-messenger-mediated signaling | 10 | 1 | 1 | 1 | 1 | 1 | 1 | 0 | 0 | 1 | 1 | 0 | 0 | 0 | 0 | 0 | 0 | 1 | 0 | 0 | 0 | 1 |
| GO:0005975~carbohydrate metabolic process | 10 | 1 | 1 | 1 | 1 | 1 | 1 | 0 | 0 | 1 | 1 | 0 | 0 | 0 | 0 | 0 | 0 | 1 | 0 | 0 | 0 | 1 |
| GO:0001775~cell activation | 10 | 1 | 1 | 1 | 1 | 1 | 1 | 0 | 0 | 1 | 1 | 0 | 0 | 0 | 0 | 0 | 0 | 1 | 0 | 0 | 0 | 1 |
| GO:0055066~di-, tri-valent inorganic cation homeostasis | 9 | 1 | 1 | 1 | 1 | 1 | 1 | 0 | 0 | 0 | 1 | 0 | 0 | 0 | 0 | 0 | 0 | 1 | 0 | 0 | 0 | 1 |
| GO:0051338~regulation of transferase activity | 9 | 1 | 1 | 1 | 1 | 1 | 0 | 0 | 0 | 1 | 1 | 0 | 0 | 0 | 0 | 0 | 0 | 1 | 0 | 0 | 0 | 1 |
| GO:0043549~regulation of kinase activity | 9 | 1 | 1 | 1 | 1 | 1 | 0 | 0 | 0 | 1 | 1 | 0 | 0 | 0 | 0 | 0 | 0 | 1 | 0 | 0 | 0 | 1 |
| GO:0055080~cation homeostasis | 9 | 1 | 1 | 1 | 1 | 1 | 1 | 0 | 0 | 0 | 1 | 0 | 0 | 0 | 0 | 0 | 0 | 1 | 0 | 0 | 0 | 1 |
| GO:0009100~glycoprotein metabolic process | 9 | 0 | 1 | 1 | 1 | 1 | 1 | 0 | 0 | 1 | 1 | 0 | 0 | 0 | 0 | 0 | 0 | 1 | 0 | 0 | 0 | 1 |
| GO:0009056~catabolic process | 9 | 1 | 1 | 1 | 1 | 1 | 1 | 0 | 0 | 0 | 1 | 0 | 0 | 0 | 0 | 0 | 0 | 1 | 0 | 0 | 0 | 1 |
| GO:0018193~peptidyl-amino acid modification | 9 | 1 | 1 | 1 | 1 | 1 | 1 | 0 | 0 | 1 | 1 | 0 | 0 | 0 | 0 | 0 | 0 | 1 | 0 | 0 | 0 | 0 |
| GO:0001666~response to hypoxia | 9 | 1 | 1 | 1 | 1 | 1 | 1 | 0 | 0 | 0 | 1 | 0 | 0 | 0 | 0 | 0 | 0 | 1 | 0 | 0 | 0 | 1 |
| GO:0010033~response to organic substance | 9 | 1 | 1 | 1 | 1 | 1 | 1 | 0 | 0 | 1 | 1 | 0 | 0 | 0 | 0 | 0 | 0 | 0 | 0 | 0 | 0 | 1 |
| GO:0006979~response to oxidative stress | 9 | 1 | 1 | 1 | 1 | 1 | 1 | 0 | 0 | 1 | 1 | 0 | 0 | 0 | 0 | 0 | 0 | 0 | 0 | 0 | 0 | 1 |
| GO:0007005~mitochondrion organization and biogenesis | 9 | 1 | 1 | 1 | 1 | 1 | 1 | 0 | 0 | 0 | 1 | 0 | 0 | 0 | 0 | 0 | 0 | 1 | 0 | 0 | 0 | 1 |
| GO:0043413~biopolymer glycosylation | 9 | 0 | 1 | 1 | 1 | 1 | 1 | 0 | 0 | 1 | 1 | 0 | 0 | 0 | 0 | 0 | 0 | 1 | 0 | 0 | 0 | 1 |
| GO:0031279~regulation of cyclase activity | 9 | 1 | 1 | 1 | 1 | 1 | 1 | 0 | 0 | 0 | 1 | 0 | 0 | 0 | 0 | 0 | 0 | 1 | 0 | 0 | 0 | 1 |
| GO:0006066~alcohol metabolic process | 9 | 1 | 1 | 1 | 1 | 1 | 1 | 0 | 0 | 1 | 1 | 0 | 0 | 0 | 0 | 0 | 0 | 0 | 0 | 0 | 0 | 1 |
| GO:0051339~regulation of lyase activity | 9 | 1 | 1 | 1 | 1 | 1 | 1 | 0 | 0 | 0 | 1 | 0 | 0 | 0 | 0 | 0 | 0 | 1 | 0 | 0 | 0 | 1 |
| GO:0006725~aromatic compound metabolic process | 9 | 1 | 1 | 1 | 1 | 1 | 1 | 0 | 0 | 0 | 1 | 0 | 0 | 0 | 0 | 0 | 0 | 1 | 0 | 0 | 0 | 1 |
| GO:0044248~cellular catabolic process | 8 | 1 | 1 | 1 | 1 | 1 | 1 | 0 | 0 | 0 | 1 | 0 | 0 | 0 | 0 | 0 | 0 | 1 | 0 | 0 | 0 | 0 |
| GO:0007179~transforming growth factor beta receptor signaling pathway | 8 | 1 | 1 | 1 | 1 | 1 | 0 | 0 | 0 | 0 | 1 | 0 | 0 | 0 | 0 | 0 | 0 | 1 | 0 | 0 | 0 | 1 |
| GO:0006259~DNA metabolic process | 8 | 1 | 1 | 1 | 1 | 1 | 1 | 0 | 0 | 0 | 1 | 0 | 0 | 0 | 0 | 0 | 0 | 0 | 0 | 0 | 0 | 1 |
| GO:0010035~response to inorganic substance | 8 | 1 | 1 | 1 | 1 | 1 | 0 | 0 | 0 | 1 | 1 | 0 | 0 | 0 | 0 | 0 | 0 | 0 | 0 | 0 | 0 | 1 |
| GO:0045860~positive regulation of protein kinase activity | 8 | 1 | 1 | 1 | 1 | 1 | 0 | 0 | 0 | 1 | 0 | 0 | 0 | 0 | 0 | 0 | 0 | 1 | 0 | 0 | 0 | 1 |
| GO:0006575~amino acid derivative metabolic process | 8 | 1 | 0 | 0 | 1 | 1 | 1 | 0 | 0 | 1 | 1 | 0 | 0 | 0 | 0 | 0 | 0 | 1 | 0 | 0 | 0 | 1 |
| GO:0009266~response to temperature stimulus | 8 | 0 | 1 | 1 | 1 | 1 | 1 | 0 | 0 | 0 | 1 | 0 | 0 | 0 | 0 | 0 | 0 | 1 | 0 | 0 | 0 | 1 |
| GO:0051347~positive regulation of transferase activity | 8 | 1 | 1 | 1 | 1 | 1 | 0 | 0 | 0 | 1 | 0 | 0 | 0 | 0 | 0 | 0 | 0 | 1 | 0 | 0 | 0 | 1 |
| GO:0007265~Ras protein signal transduction | 8 | 1 | 0 | 0 | 1 | 1 | 1 | 0 | 0 | 1 | 1 | 0 | 0 | 0 | 0 | 0 | 0 | 1 | 0 | 0 | 0 | 1 |
| GO:0009725~response to hormone stimulus | 8 | 1 | 1 | 1 | 1 | 1 | 1 | 0 | 0 | 1 | 0 | 0 | 0 | 0 | 0 | 0 | 0 | 0 | 0 | 0 | 0 | 1 |
| GO:0033674~positive regulation of kinase activity | 8 | 1 | 1 | 1 | 1 | 1 | 0 | 0 | 0 | 1 | 0 | 0 | 0 | 0 | 0 | 0 | 0 | 1 | 0 | 0 | 0 | 1 |
| GO:0009058~biosynthetic process | 8 | 1 | 0 | 0 | 1 | 1 | 1 | 0 | 0 | 1 | 1 | 0 | 0 | 0 | 0 | 0 | 0 | 1 | 0 | 0 | 0 | 1 |
| GO:0006732~coenzyme metabolic process | 8 | 1 | 0 | 0 | 1 | 1 | 1 | 0 | 0 | 1 | 1 | 0 | 0 | 0 | 0 | 0 | 0 | 0 | 0 | 0 | 1 | 1 |
| GO:0032787~monocarboxylic acid metabolic process | 7 | 1 | 0 | 0 | 1 | 1 | 0 | 0 | 0 | 1 | 1 | 0 | 0 | 0 | 0 | 0 | 0 | 1 | 0 | 0 | 0 | 1 |
| GO:0010038~response to metal ion | 7 | 1 | 1 | 1 | 1 | 1 | 0 | 0 | 0 | 1 | 0 | 0 | 0 | 0 | 0 | 0 | 0 | 0 | 0 | 0 | 0 | 1 |
| GO:0042060~wound healing | 7 | 1 | 0 | 0 | 1 | 1 | 1 | 0 | 0 | 0 | 1 | 0 | 0 | 0 | 0 | 0 | 0 | 1 | 0 | 0 | 0 | 1 |
| GO:0009314~response to radiation | 7 | 1 | 1 | 1 | 1 | 1 | 1 | 0 | 0 | 0 | 1 | 0 | 0 | 0 | 0 | 0 | 0 | 0 | 0 | 0 | 0 | 0 |
| GO:0009416~response to light stimulus | 7 | 1 | 1 | 1 | 1 | 1 | 1 | 0 | 0 | 0 | 1 | 0 | 0 | 0 | 0 | 0 | 0 | 0 | 0 | 0 | 0 | 0 |
| GO:0019933~cAMP-mediated signaling | 7 | 1 | 1 | 1 | 1 | 1 | 0 | 0 | 0 | 0 | 1 | 0 | 0 | 0 | 0 | 0 | 0 | 0 | 0 | 0 | 0 | 1 |
| GO:0006974~response to DNA damage stimulus | 7 | 1 | 1 | 1 | 1 | 1 | 1 | 0 | 0 | 0 | 1 | 0 | 0 | 0 | 0 | 0 | 0 | 0 | 0 | 0 | 0 | 0 |
| GO:0006091~generation of precursor metabolites and energy | 7 | 1 | 0 | 0 | 1 | 1 | 1 | 0 | 0 | 1 | 1 | 0 | 0 | 0 | 0 | 0 | 0 | 0 | 0 | 0 | 0 | 1 |
| GO:0016337~cell-cell adhesion | 6 | 0 | 1 | 1 | 0 | 0 | 0 | 1 | 0 | 1 | 1 | 0 | 0 | 0 | 0 | 0 | 0 | 1 | 0 | 0 | 0 | 0 |
| GO:0006944~membrane fusion | 6 | 0 | 1 | 1 | 0 | 0 | 1 | 0 | 0 | 1 | 1 | 0 | 0 | 0 | 0 | 0 | 0 | 0 | 0 | 0 | 0 | 1 |
| GO:0007249~I-kappaB kinase/NF-kappaB cascade | 6 | 0 | 0 | 0 | 1 | 1 | 1 | 0 | 0 | 1 | 1 | 0 | 0 | 0 | 0 | 0 | 0 | 0 | 0 | 0 | 0 | 1 |
| GO:0055086~nucleobase, nucleoside and nucleotide metabolic process | 6 | 1 | 1 | 1 | 1 | 1 | 0 | 0 | 0 | 0 | 1 | 0 | 0 | 0 | 0 | 0 | 0 | 0 | 0 | 0 | 0 | 0 |
| GO:0048511~rhythmic process | 6 | 1 | 1 | 1 | 1 | 1 | 0 | 0 | 0 | 0 | 0 | 0 | 0 | 0 | 0 | 0 | 0 | 0 | 0 | 0 | 0 | 1 |
| GO:0042157~lipoprotein metabolic process | 6 | 0 | 1 | 1 | 1 | 1 | 1 | 0 | 0 | 1 | 0 | 0 | 0 | 0 | 0 | 0 | 0 | 0 | 0 | 0 | 0 | 0 |
| GO:0043406~positive regulation of MAP kinase activity | 6 | 1 | 1 | 1 | 1 | 1 | 0 | 0 | 0 | 1 | 0 | 0 | 0 | 0 | 0 | 0 | 0 | 0 | 0 | 0 | 0 | 0 |
| GO:0043285~biopolymer catabolic process | 5 | 1 | 0 | 0 | 1 | 1 | 1 | 0 | 0 | 0 | 1 | 0 | 0 | 0 | 0 | 0 | 0 | 0 | 0 | 0 | 0 | 0 |
| GO:0006952~defense response | 5 | 1 | 0 | 0 | 1 | 1 | 0 | 0 | 0 | 0 | 1 | 0 | 0 | 0 | 0 | 0 | 0 | 1 | 0 | 0 | 0 | 0 |
| GO:0043086~negative regulation of catalytic activity | 5 | 1 | 1 | 1 | 0 | 0 | 0 | 0 | 0 | 1 | 1 | 0 | 0 | 0 | 0 | 0 | 0 | 0 | 0 | 0 | 0 | 0 |
| GO:0003018~vascular process in circulatory system | 5 | 0 | 0 | 0 | 1 | 1 | 1 | 0 | 0 | 0 | 1 | 0 | 0 | 0 | 0 | 0 | 0 | 1 | 0 | 0 | 0 | 0 |
| GO:0006936~muscle contraction | 5 | 0 | 0 | 0 | 1 | 1 | 1 | 0 | 0 | 0 | 0 | 0 | 0 | 0 | 0 | 0 | 0 | 1 | 0 | 0 | 0 | 1 |
| GO:0007283~spermatogenesis | 5 | 0 | 0 | 0 | 1 | 1 | 1 | 0 | 0 | 1 | 0 | 0 | 0 | 0 | 0 | 0 | 0 | 0 | 0 | 0 | 0 | 1 |
| GO:0048232~male gamete generation | 5 | 0 | 0 | 0 | 1 | 1 | 1 | 0 | 0 | 1 | 0 | 0 | 0 | 0 | 0 | 0 | 0 | 0 | 0 | 0 | 0 | 1 |
| GO:0019935~cyclic-nucleotide-mediated signaling | 5 | 1 | 1 | 1 | 0 | 0 | 1 | 0 | 0 | 0 | 1 | 0 | 0 | 0 | 0 | 0 | 0 | 0 | 0 | 0 | 0 | 0 |
| GO:0003012~muscle system process | 5 | 0 | 0 | 0 | 1 | 1 | 1 | 0 | 0 | 0 | 0 | 0 | 0 | 0 | 0 | 0 | 0 | 1 | 0 | 0 | 0 | 1 |
| GO:0019953~sexual reproduction | 5 | 0 | 0 | 0 | 1 | 1 | 1 | 0 | 0 | 1 | 0 | 0 | 0 | 0 | 0 | 0 | 0 | 0 | 0 | 0 | 0 | 1 |
| GO:0009582~detection of abiotic stimulus | 5 | 0 | 1 | 1 | 1 | 0 | 0 | 0 | 0 | 0 | 1 | 0 | 0 | 0 | 0 | 0 | 0 | 0 | 0 | 0 | 0 | 1 |
| GO:0007276~gamete generation | 5 | 0 | 0 | 0 | 1 | 1 | 1 | 0 | 0 | 1 | 0 | 0 | 0 | 0 | 0 | 0 | 0 | 0 | 0 | 0 | 0 | 1 |
| GO:0009991~response to extracellular stimulus | 5 | 1 | 0 | 0 | 1 | 1 | 0 | 0 | 0 | 0 | 0 | 0 | 0 | 0 | 0 | 0 | 0 | 1 | 0 | 0 | 0 | 1 |
| GO:0031667~response to nutrient levels | 5 | 1 | 0 | 0 | 1 | 1 | 0 | 0 | 0 | 0 | 0 | 0 | 0 | 0 | 0 | 0 | 0 | 1 | 0 | 0 | 0 | 1 |
| GO:0019221~cytokine and chemokine mediated signaling pathway | 5 | 1 | 0 | 0 | 1 | 1 | 1 | 0 | 0 | 1 | 0 | 0 | 0 | 0 | 0 | 0 | 0 | 0 | 0 | 0 | 0 | 0 |
| GO:0009581~detection of external stimulus | 5 | 0 | 1 | 1 | 1 | 1 | 0 | 0 | 0 | 0 | 1 | 0 | 0 | 0 | 0 | 0 | 0 | 0 | 0 | 0 | 0 | 0 |
| GO:0046483~heterocycle metabolic process | 5 | 1 | 0 | 0 | 1 | 1 | 0 | 0 | 0 | 0 | 1 | 0 | 0 | 0 | 0 | 0 | 0 | 1 | 0 | 0 | 0 | 0 |
| GO:0051186~cofactor metabolic process | 5 | 1 | 0 | 0 | 1 | 1 | 1 | 0 | 0 | 0 | 1 | 0 | 0 | 0 | 0 | 0 | 0 | 0 | 0 | 0 | 0 | 0 |
| GO:0007611~learning and/or memory | 4 | 0 | 0 | 0 | 1 | 1 | 1 | 0 | 0 | 0 | 1 | 0 | 0 | 0 | 0 | 0 | 0 | 0 | 0 | 0 | 0 | 0 |
| GO:0009117~nucleotide metabolic process | 4 | 1 | 0 | 0 | 1 | 1 | 0 | 0 | 0 | 0 | 1 | 0 | 0 | 0 | 0 | 0 | 0 | 0 | 0 | 0 | 0 | 0 |
| GO:0003015~heart process | 4 | 0 | 0 | 0 | 1 | 1 | 1 | 0 | 0 | 0 | 0 | 0 | 0 | 0 | 0 | 0 | 0 | 0 | 0 | 0 | 0 | 1 |
| GO:0060047~heart contraction | 4 | 0 | 0 | 0 | 1 | 1 | 1 | 0 | 0 | 0 | 0 | 0 | 0 | 0 | 0 | 0 | 0 | 0 | 0 | 0 | 0 | 1 |
| GO:0050953~sensory perception of light stimulus | 4 | 0 | 0 | 0 | 1 | 1 | 0 | 0 | 0 | 0 | 1 | 0 | 0 | 0 | 0 | 0 | 0 | 0 | 0 | 0 | 0 | 1 |
| GO:0006512~ubiquitin cycle | 4 | 1 | 0 | 0 | 0 | 0 | 0 | 0 | 0 | 1 | 1 | 0 | 0 | 0 | 0 | 0 | 0 | 0 | 0 | 0 | 0 | 1 |
| GO:0032504~multicellular organism reproduction | 4 | 0 | 0 | 0 | 1 | 1 | 0 | 0 | 0 | 0 | 0 | 0 | 0 | 0 | 0 | 0 | 0 | 1 | 0 | 0 | 0 | 1 |
| GO:0007601~visual perception | 4 | 0 | 0 | 0 | 1 | 1 | 0 | 0 | 0 | 0 | 1 | 0 | 0 | 0 | 0 | 0 | 0 | 0 | 0 | 0 | 0 | 1 |
| GO:0043434~response to peptide hormone stimulus | 4 | 1 | 0 | 0 | 0 | 0 | 1 | 0 | 0 | 0 | 1 | 0 | 0 | 0 | 0 | 0 | 0 | 0 | 0 | 0 | 0 | 1 |
| GO:0007015~actin filament organization | 4 | 0 | 1 | 1 | 0 | 0 | 0 | 0 | 0 | 0 | 1 | 0 | 0 | 0 | 0 | 0 | 0 | 0 | 0 | 0 | 0 | 1 |
| GO:0051789~response to protein stimulus | 4 | 1 | 0 | 0 | 0 | 0 | 1 | 0 | 0 | 1 | 1 | 0 | 0 | 0 | 0 | 0 | 0 | 0 | 0 | 0 | 0 | 0 |
| GO:0031098~stress-activated protein kinase signaling pathway | 4 | 0 | 0 | 0 | 1 | 1 | 0 | 0 | 0 | 0 | 1 | 0 | 0 | 0 | 0 | 0 | 0 | 1 | 0 | 0 | 0 | 0 |
| GO:0006955~immune response | 4 | 1 | 0 | 0 | 0 | 0 | 1 | 0 | 0 | 0 | 1 | 0 | 0 | 0 | 0 | 0 | 0 | 0 | 0 | 0 | 0 | 1 |
| GO:0007156~homophilic cell adhesion | 3 | 0 | 0 | 0 | 0 | 0 | 0 | 1 | 0 | 1 | 0 | 0 | 0 | 0 | 0 | 0 | 0 | 1 | 0 | 0 | 0 | 0 |
| GO:0048015~phosphoinositide-mediated signaling | 3 | 0 | 0 | 0 | 1 | 1 | 0 | 0 | 0 | 0 | 0 | 0 | 0 | 0 | 0 | 0 | 0 | 1 | 0 | 0 | 0 | 0 |
| GO:0018212~peptidyl-tyrosine modification | 3 | 0 | 0 | 0 | 1 | 1 | 0 | 0 | 0 | 0 | 0 | 0 | 0 | 0 | 0 | 0 | 0 | 1 | 0 | 0 | 0 | 0 |
| GO:0032943~mononuclear cell proliferation | 3 | 0 | 0 | 0 | 1 | 1 | 0 | 0 | 0 | 0 | 1 | 0 | 0 | 0 | 0 | 0 | 0 | 0 | 0 | 0 | 0 | 0 |
| GO:0009259~ribonucleotide metabolic process | 3 | 1 | 0 | 0 | 1 | 1 | 0 | 0 | 0 | 0 | 0 | 0 | 0 | 0 | 0 | 0 | 0 | 0 | 0 | 0 | 0 | 0 |
| GO:0032446~protein modification by small protein conjugation | 3 | 1 | 0 | 0 | 0 | 0 | 0 | 0 | 0 | 0 | 1 | 0 | 0 | 0 | 0 | 0 | 0 | 1 | 0 | 0 | 0 | 0 |
| GO:0005976~polysaccharide metabolic process | 3 | 1 | 0 | 0 | 0 | 0 | 0 | 0 | 0 | 0 | 1 | 0 | 0 | 0 | 0 | 0 | 0 | 1 | 0 | 0 | 0 | 0 |
| GO:0007218~neuropeptide signaling pathway | 3 | 0 | 0 | 0 | 0 | 0 | 1 | 0 | 0 | 1 | 0 | 0 | 0 | 0 | 0 | 0 | 0 | 0 | 0 | 0 | 0 | 1 |
| GO:0032535~regulation of cellular component size | 3 | 0 | 0 | 0 | 0 | 0 | 0 | 0 | 0 | 1 | 1 | 0 | 0 | 0 | 0 | 0 | 0 | 0 | 0 | 0 | 0 | 1 |
| GO:0006457~protein folding | 3 | 0 | 0 | 0 | 0 | 0 | 1 | 0 | 0 | 0 | 1 | 0 | 0 | 0 | 0 | 0 | 0 | 1 | 0 | 0 | 0 | 0 |
| GO:0043405~regulation of MAP kinase activity | 3 | 1 | 0 | 0 | 0 | 0 | 0 | 0 | 0 | 1 | 0 | 0 | 0 | 0 | 0 | 0 | 0 | 1 | 0 | 0 | 0 | 0 |
| GO:0044272~sulfur compound biosynthetic process | 3 | 1 | 0 | 0 | 1 | 1 | 0 | 0 | 0 | 0 | 0 | 0 | 0 | 0 | 0 | 0 | 0 | 0 | 0 | 0 | 0 | 0 |
| GO:0048659~smooth muscle cell proliferation | 3 | 0 | 0 | 0 | 1 | 1 | 0 | 0 | 0 | 0 | 1 | 0 | 0 | 0 | 0 | 0 | 0 | 0 | 0 | 0 | 0 | 0 |
| GO:0009108~coenzyme biosynthetic process | 3 | 1 | 0 | 0 | 1 | 1 | 0 | 0 | 0 | 0 | 0 | 0 | 0 | 0 | 0 | 0 | 0 | 0 | 0 | 0 | 0 | 0 |
| GO:0006939~smooth muscle contraction | 3 | 0 | 0 | 0 | 1 | 1 | 0 | 0 | 0 | 0 | 0 | 0 | 0 | 0 | 0 | 0 | 0 | 0 | 0 | 0 | 0 | 1 |
| GO:0031589~cell-substrate adhesion | 3 | 0 | 1 | 1 | 0 | 0 | 1 | 0 | 0 | 0 | 0 | 0 | 0 | 0 | 0 | 0 | 0 | 0 | 0 | 0 | 0 | 0 |
| GO:0033002~muscle cell proliferation | 3 | 0 | 0 | 0 | 1 | 1 | 0 | 0 | 0 | 0 | 1 | 0 | 0 | 0 | 0 | 0 | 0 | 0 | 0 | 0 | 0 | 0 |
| GO:0007266~Rho protein signal transduction | 3 | 0 | 0 | 0 | 1 | 1 | 0 | 0 | 0 | 1 | 0 | 0 | 0 | 0 | 0 | 0 | 0 | 0 | 0 | 0 | 0 | 0 |
| GO:0051345~positive regulation of hydrolase activity | 2 | 0 | 0 | 0 | 0 | 0 | 1 | 0 | 0 | 0 | 0 | 0 | 0 | 0 | 0 | 0 | 0 | 1 | 0 | 0 | 0 | 0 |
| GO:0006260~DNA replication | 2 | 0 | 0 | 0 | 0 | 0 | 0 | 0 | 0 | 0 | 1 | 0 | 0 | 0 | 0 | 0 | 0 | 0 | 0 | 0 | 0 | 1 |
| GO:0044262~cellular carbohydrate metabolic process | 2 | 1 | 0 | 0 | 0 | 0 | 0 | 0 | 0 | 0 | 0 | 0 | 0 | 0 | 0 | 0 | 0 | 1 | 0 | 0 | 0 | 0 |
| GO:0008213~protein amino acid alkylation | 2 | 1 | 0 | 0 | 0 | 0 | 0 | 0 | 0 | 0 | 1 | 0 | 0 | 0 | 0 | 0 | 0 | 0 | 0 | 0 | 0 | 0 |
| GO:0048736~appendage development | 2 | 0 | 0 | 0 | 0 | 0 | 1 | 0 | 0 | 0 | 0 | 0 | 0 | 0 | 0 | 0 | 0 | 0 | 0 | 0 | 0 | 1 |
| GO:0016053~organic acid biosynthetic process | 2 | 1 | 0 | 0 | 0 | 0 | 0 | 0 | 0 | 0 | 0 | 0 | 0 | 0 | 0 | 0 | 0 | 1 | 0 | 0 | 0 | 0 |
| GO:0016051~carbohydrate biosynthetic process | 2 | 0 | 0 | 0 | 0 | 0 | 0 | 0 | 0 | 1 | 0 | 0 | 0 | 0 | 0 | 0 | 0 | 1 | 0 | 0 | 0 | 0 |
| GO:0046394~carboxylic acid biosynthetic process | 2 | 1 | 0 | 0 | 0 | 0 | 0 | 0 | 0 | 0 | 0 | 0 | 0 | 0 | 0 | 0 | 0 | 1 | 0 | 0 | 0 | 0 |
| GO:0043414~biopolymer methylation | 2 | 1 | 0 | 0 | 0 | 0 | 1 | 0 | 0 | 0 | 0 | 0 | 0 | 0 | 0 | 0 | 0 | 0 | 0 | 0 | 0 | 0 |
| GO:0043062~extracellular structure organization and biogenesis | 2 | 1 | 0 | 0 | 0 | 0 | 0 | 0 | 0 | 0 | 0 | 0 | 0 | 0 | 0 | 0 | 0 | 0 | 0 | 0 | 1 | 0 |
| GO:0000910~cytokinesis | 2 | 0 | 0 | 0 | 1 | 1 | 0 | 0 | 0 | 0 | 0 | 0 | 0 | 0 | 0 | 0 | 0 | 0 | 0 | 0 | 0 | 0 |
| GO:0040011~locomotion | 2 | 0 | 0 | 0 | 0 | 0 | 0 | 0 | 0 | 1 | 1 | 0 | 0 | 0 | 0 | 0 | 0 | 0 | 0 | 0 | 0 | 0 |
| GO:0007631~feeding behavior | 2 | 0 | 0 | 0 | 0 | 0 | 0 | 0 | 0 | 0 | 0 | 0 | 0 | 0 | 0 | 0 | 0 | 1 | 0 | 0 | 0 | 1 |
| GO:0042446~hormone biosynthetic process | 2 | 1 | 0 | 0 | 0 | 0 | 1 | 0 | 0 | 0 | 0 | 0 | 0 | 0 | 0 | 0 | 0 | 0 | 0 | 0 | 0 | 0 |
| GO:0060173~limb development | 2 | 0 | 0 | 0 | 0 | 0 | 1 | 0 | 0 | 0 | 0 | 0 | 0 | 0 | 0 | 0 | 0 | 0 | 0 | 0 | 0 | 1 |
| GO:0016055~Wnt receptor signaling pathway | 2 | 0 | 0 | 0 | 0 | 0 | 1 | 0 | 0 | 0 | 0 | 0 | 0 | 0 | 0 | 0 | 0 | 1 | 0 | 0 | 0 | 0 |
| GO:0030198~extracellular matrix organization and biogenesis | 2 | 1 | 0 | 0 | 0 | 0 | 0 | 0 | 0 | 0 | 1 | 0 | 0 | 0 | 0 | 0 | 0 | 0 | 0 | 0 | 0 | 0 |
| GO:0006118~electron transport | 2 | 1 | 0 | 0 | 0 | 0 | 0 | 0 | 0 | 0 | 0 | 0 | 0 | 0 | 0 | 0 | 0 | 0 | 0 | 0 | 0 | 1 |
| GO:0051261~protein depolymerization | 2 | 0 | 0 | 0 | 0 | 0 | 0 | 0 | 0 | 0 | 1 | 0 | 0 | 0 | 0 | 0 | 0 | 0 | 0 | 0 | 0 | 1 |
| GO:0051258~protein polymerization | 2 | 0 | 0 | 0 | 0 | 0 | 0 | 0 | 0 | 1 | 0 | 0 | 0 | 0 | 0 | 0 | 0 | 0 | 0 | 0 | 0 | 1 |
| GO:0048762~mesenchymal cell differentiation | 2 | 0 | 0 | 0 | 0 | 0 | 0 | 0 | 0 | 1 | 0 | 0 | 0 | 0 | 0 | 0 | 0 | 0 | 0 | 0 | 0 | 1 |
| GO:0032147~activation of protein kinase activity | 2 | 0 | 1 | 1 | 0 | 0 | 0 | 0 | 0 | 0 | 0 | 0 | 0 | 0 | 0 | 0 | 0 | 0 | 0 | 0 | 0 | 0 |
| GO:0042445~hormone metabolic process | 2 | 0 | 0 | 0 | 0 | 0 | 1 | 0 | 0 | 0 | 0 | 0 | 0 | 0 | 0 | 0 | 0 | 0 | 0 | 0 | 0 | 1 |
| GO:0051716~cellular response to stimulus | 2 | 0 | 0 | 0 | 1 | 1 | 0 | 0 | 0 | 0 | 0 | 0 | 0 | 0 | 0 | 0 | 0 | 0 | 0 | 0 | 0 | 0 |
| GO:0001816~cytokine production | 1 | 0 | 0 | 0 | 0 | 0 | 0 | 0 | 0 | 0 | 1 | 0 | 0 | 0 | 0 | 0 | 0 | 0 | 0 | 0 | 0 | 0 |
| GO:0016071~mRNA metabolic process | 1 | 0 | 0 | 0 | 0 | 0 | 0 | 0 | 0 | 0 | 1 | 0 | 0 | 0 | 0 | 0 | 0 | 0 | 0 | 0 | 0 | 0 |
| GO:0048589~developmental growth | 1 | 0 | 0 | 0 | 0 | 0 | 1 | 0 | 0 | 0 | 0 | 0 | 0 | 0 | 0 | 0 | 0 | 0 | 0 | 0 | 0 | 0 |
| GO:0021700~developmental maturation | 1 | 1 | 0 | 0 | 0 | 0 | 0 | 0 | 0 | 0 | 0 | 0 | 0 | 0 | 0 | 0 | 0 | 0 | 0 | 0 | 0 | 0 |
| GO:0030534~adult behavior | 1 | 0 | 0 | 0 | 0 | 0 | 1 | 0 | 0 | 0 | 0 | 0 | 0 | 0 | 0 | 0 | 0 | 0 | 0 | 0 | 0 | 0 |
| GO:0001655~urogenital system development | 1 | 0 | 0 | 0 | 0 | 0 | 0 | 0 | 0 | 0 | 0 | 0 | 0 | 0 | 0 | 0 | 0 | 0 | 0 | 0 | 0 | 1 |
| GO:0007612~learning | 1 | 0 | 0 | 0 | 0 | 0 | 0 | 0 | 0 | 0 | 1 | 0 | 0 | 0 | 0 | 0 | 0 | 0 | 0 | 0 | 0 | 0 |
| GO:0019233~sensory perception of pain | 1 | 0 | 0 | 0 | 0 | 0 | 0 | 0 | 0 | 0 | 0 | 0 | 0 | 0 | 0 | 0 | 0 | 1 | 0 | 0 | 0 | 0 |
| GO:0014070~response to organic cyclic substance | 1 | 0 | 0 | 0 | 0 | 0 | 0 | 0 | 0 | 0 | 0 | 0 | 0 | 0 | 0 | 0 | 0 | 0 | 0 | 0 | 0 | 1 |
| GO:0000226~microtubule cytoskeleton organization and biogenesis | 1 | 0 | 0 | 0 | 0 | 0 | 0 | 0 | 0 | 0 | 0 | 0 | 0 | 0 | 0 | 0 | 0 | 0 | 0 | 0 | 0 | 1 |
| GO:0007017~microtubule-based process | 1 | 0 | 0 | 0 | 0 | 0 | 0 | 0 | 0 | 0 | 0 | 0 | 0 | 0 | 0 | 0 | 0 | 0 | 0 | 0 | 0 | 1 |
| GO:0033673~negative regulation of kinase activity | 1 | 0 | 0 | 0 | 0 | 0 | 0 | 0 | 0 | 0 | 1 | 0 | 0 | 0 | 0 | 0 | 0 | 0 | 0 | 0 | 0 | 0 |
| GO:0007154~cell communication | 1 | 0 | 0 | 0 | 0 | 0 | 1 | 0 | 0 | 0 | 0 | 0 | 0 | 0 | 0 | 0 | 0 | 0 | 0 | 0 | 0 | 0 |
| GO:0007613~memory | 1 | 0 | 0 | 0 | 0 | 0 | 0 | 0 | 0 | 0 | 1 | 0 | 0 | 0 | 0 | 0 | 0 | 0 | 0 | 0 | 0 | 0 |
| GO:0051348~negative regulation of transferase activity | 1 | 0 | 0 | 0 | 0 | 0 | 0 | 0 | 0 | 0 | 1 | 0 | 0 | 0 | 0 | 0 | 0 | 0 | 0 | 0 | 0 | 0 |
| GO:0009410~response to xenobiotic stimulus | 1 | 0 | 0 | 0 | 0 | 0 | 0 | 0 | 0 | 0 | 1 | 0 | 0 | 0 | 0 | 0 | 0 | 0 | 0 | 0 | 0 | 0 |
| GO:0048545~response to steroid hormone stimulus | 1 | 1 | 0 | 0 | 0 | 0 | 0 | 0 | 0 | 0 | 0 | 0 | 0 | 0 | 0 | 0 | 0 | 0 | 0 | 0 | 0 | 0 |
| GO:0050817~coagulation | 1 | 0 | 0 | 0 | 0 | 0 | 0 | 0 | 0 | 0 | 1 | 0 | 0 | 0 | 0 | 0 | 0 | 0 | 0 | 0 | 0 | 0 |
| GO:0007160~cell-matrix adhesion | 1 | 0 | 0 | 0 | 0 | 0 | 1 | 0 | 0 | 0 | 0 | 0 | 0 | 0 | 0 | 0 | 0 | 0 | 0 | 0 | 0 | 0 |
| GO:0006469~negative regulation of protein kinase activity | 1 | 0 | 0 | 0 | 0 | 0 | 0 | 0 | 0 | 0 | 1 | 0 | 0 | 0 | 0 | 0 | 0 | 0 | 0 | 0 | 0 | 0 |
| GO:0051349~positive regulation of lyase activity | 1 | 0 | 0 | 0 | 0 | 0 | 0 | 0 | 0 | 0 | 1 | 0 | 0 | 0 | 0 | 0 | 0 | 0 | 0 | 0 | 0 | 0 |
| GO:0007623~circadian rhythm | 1 | 1 | 0 | 0 | 0 | 0 | 0 | 0 | 0 | 0 | 0 | 0 | 0 | 0 | 0 | 0 | 0 | 0 | 0 | 0 | 0 | 0 |
| GO:0031281~positive regulation of cyclase activity | 1 | 0 | 0 | 0 | 0 | 0 | 0 | 0 | 0 | 0 | 1 | 0 | 0 | 0 | 0 | 0 | 0 | 0 | 0 | 0 | 0 | 0 |
| Total pathways predicted to target by each miRNA | | 168 | 142 | 142 | 177 | 176 | 160 | 27 | 0 | 141 | 177 | 0 | 5 | 21 | 19 | 34 | 23 | 148 | 0 | 0 | 45 | 168 |

Sum: it denotes how many miRNAs are putatively targeting a given pathway. Total pathways predicted by each miRNA: how many pathways are predicted by each miRNA.

1: Predicted to target; 0: not targeted.

**Supplementary Table 6A**

| Pathways | Sum | rno-miR-338 | rno-miR-19b | rno-miR-19a | rno-miR-30c | rno-miR-145 | rno-miR-653 | rno-miR-3556a | rno-miR-3596b | rno-miR-3590-5p |
| --- | --- | --- | --- | --- | --- | --- | --- | --- | --- | --- |
| rno04360 Axon guidance | 5 | 0 | 1 | 1 | 1 | 1 | 0 | 0 | 0 | 1 |
| rno05219 Bladder cancer | 5 | 1 | 1 | 1 | 1 | 1 | 0 | 0 | 0 | 0 |
| rno01100 Metabolic pathways | 5 | 1 | 1 | 1 | 1 | 1 | 0 | 0 | 0 | 0 |
| rno05212 Pancreatic cancer | 4 | 1 | 0 | 0 | 1 | 1 | 0 | 0 | 0 | 1 |
| rno05211 Renal cell carcinoma | 4 | 1 | 1 | 1 | 1 | 0 | 0 | 0 | 0 | 0 |
| rno04144 Endocytosis | 4 | 1 | 1 | 1 | 1 | 0 | 0 | 0 | 0 | 0 |
| rno04130 SNARE interactions in vesicular transport | 4 | 1 | 1 | 1 | 1 | 0 | 0 | 0 | 0 | 0 |
| rno04971 Gastric acid secretion | 3 | 0 | 1 | 1 | 0 | 1 | 0 | 0 | 0 | 0 |
| rno04010 MAPK signaling pathway | 3 | 1 | 0 | 0 | 1 | 1 | 0 | 0 | 0 | 0 |
| rno00410 beta-Alanine metabolism | 3 | 1 | 1 | 1 | 0 | 0 | 0 | 0 | 0 | 0 |
| rno04666 Fc gamma R-mediated phagocytosis | 3 | 1 | 1 | 1 | 0 | 0 | 0 | 0 | 0 | 0 |
| rno04920 Adipocytokine signaling pathway | 3 | 1 | 0 | 1 | 1 | 0 | 0 | 0 | 0 | 0 |
| rno04062 Chemokine signaling pathway | 3 | 1 | 0 | 1 | 1 | 0 | 0 | 0 | 0 | 0 |
| rno04722 Neurotrophin signaling pathway | 3 | 1 | 0 | 0 | 1 | 1 | 0 | 0 | 0 | 0 |
| rno05220 Chronic myeloid leukemia | 3 | 1 | 0 | 0 | 1 | 0 | 0 | 0 | 0 | 1 |
| rno04810 Regulation of actin cytoskeleton | 3 | 1 | 1 | 1 | 0 | 0 | 0 | 0 | 0 | 0 |
| rno05200 Pathways in cancer | 3 | 1 | 0 | 0 | 1 | 0 | 0 | 0 | 0 | 1 |
| rno04210 Apoptosis | 3 | 1 | 0 | 0 | 1 | 1 | 0 | 0 | 0 | 0 |
| rno04070 Phosphatidylinositol signaling system | 3 | 1 | 0 | 1 | 1 | 0 | 0 | 0 | 0 | 0 |
| rno04510 Focal adhesion | 2 | 1 | 0 | 0 | 1 | 0 | 0 | 0 | 0 | 0 |
| rno04660 T cell receptor signaling pathway | 2 | 1 | 0 | 0 | 1 | 0 | 0 | 0 | 0 | 0 |
| rno00120 Primary bile acid biosynthesis | 2 | 0 | 0 | 0 | 1 | 1 | 0 | 0 | 0 | 0 |
| rno00280 Valine, leucine and isoleucine degradation | 2 | 0 | 1 | 1 | 0 | 0 | 0 | 0 | 0 | 0 |
| rno04662 B cell receptor signaling pathway | 2 | 1 | 0 | 0 | 1 | 0 | 0 | 0 | 0 | 0 |
| rno04060 Cytokine-cytokine receptor interaction | 2 | 0 | 0 | 0 | 1 | 1 | 0 | 0 | 0 | 0 |
| rno04310 Wnt signaling pathway | 2 | 1 | 0 | 0 | 1 | 0 | 0 | 0 | 0 | 0 |
| rno04110 Cell cycle | 2 | 1 | 0 | 0 | 1 | 0 | 0 | 0 | 0 | 0 |
| rno04114 Oocyte meiosis | 2 | 1 | 0 | 0 | 1 | 0 | 0 | 0 | 0 | 0 |
| rno00770 Pantothenate and CoA biosynthesis | 2 | 1 | 0 | 0 | 1 | 0 | 0 | 0 | 0 | 0 |
| rno04962 Vasopressin-regulated water reabsorption | 2 | 1 | 0 | 0 | 0 | 1 | 0 | 0 | 0 | 0 |
| rno04012 ErbB signaling pathway | 2 | 1 | 0 | 0 | 1 | 0 | 0 | 0 | 0 | 0 |
| rno04020 Calcium signaling pathway | 2 | 1 | 0 | 0 | 0 | 1 | 0 | 0 | 0 | 0 |
| rno05223 Non-small cell lung cancer | 2 | 1 | 0 | 0 | 0 | 0 | 0 | 0 | 0 | 1 |
| rno00640 Propanoate metabolism | 2 | 0 | 1 | 1 | 0 | 0 | 0 | 0 | 0 | 0 |
| rno05142 Chagas disease | 2 | 1 | 0 | 0 | 1 | 0 | 0 | 0 | 0 | 0 |
| rno04720 Long-term potentiation | 2 | 1 | 0 | 0 | 1 | 0 | 0 | 0 | 0 | 0 |
| rno05216 Thyroid cancer | 1 | 1 | 0 | 0 | 0 | 0 | 0 | 0 | 0 | 0 |
| rno04664 Fc epsilon RI signaling pathway | 1 | 1 | 0 | 0 | 0 | 0 | 0 | 0 | 0 | 0 |
| rno00510 N-Glycan biosynthesis | 1 | 0 | 0 | 0 | 1 | 0 | 0 | 0 | 0 | 0 |
| rno04370 VEGF signaling pathway | 1 | 1 | 0 | 0 | 0 | 0 | 0 | 0 | 0 | 0 |
| rno00100 Steroid biosynthesis | 1 | 0 | 0 | 0 | 1 | 0 | 0 | 0 | 0 | 0 |
| rno00350 Tyrosine metabolism | 1 | 0 | 0 | 0 | 0 | 1 | 0 | 0 | 0 | 0 |
| rno00380 Tryptophan metabolism | 1 | 0 | 0 | 0 | 0 | 1 | 0 | 0 | 0 | 0 |
| rno04730 Long-term depression | 1 | 1 | 0 | 0 | 0 | 0 | 0 | 0 | 0 | 0 |
| rno05215 Prostate cancer | 1 | 1 | 0 | 0 | 0 | 0 | 0 | 0 | 0 | 0 |
| rno05140 Leishmaniasis | 1 | 0 | 0 | 0 | 0 | 1 | 0 | 0 | 0 | 0 |
| rno04115 p53 signaling pathway | 1 | 0 | 0 | 0 | 1 | 0 | 0 | 0 | 0 | 0 |
| rno00270 Cysteine and methionine metabolism | 1 | 0 | 0 | 0 | 1 | 0 | 0 | 0 | 0 | 0 |
| rno00564 Glycerophospholipid metabolism | 1 | 1 | 0 | 0 | 0 | 0 | 0 | 0 | 0 | 0 |
| rno05213 Endometrial cancer | 1 | 1 | 0 | 0 | 0 | 0 | 0 | 0 | 0 | 0 |
| rno04141 Protein processing in endoplasmic reticulum | 1 | 0 | 0 | 0 | 1 | 0 | 0 | 0 | 0 | 0 |
| rno02010 ABC transporters | 1 | 1 | 0 | 0 | 0 | 0 | 0 | 0 | 0 | 0 |
| rno00130 Ubiquinone and other terpenoid-quinone biosynthesis | 1 | 1 | 0 | 0 | 0 | 0 | 0 | 0 | 0 | 0 |
| rno00260 Glycine, serine and threonine metabolism | 1 | 0 | 0 | 0 | 0 | 1 | 0 | 0 | 0 | 0 |
| rno05210 Colorectal cancer | 1 | 1 | 0 | 0 | 0 | 0 | 0 | 0 | 0 | 0 |
| rno03320 PPAR signaling pathway | 1 | 1 | 0 | 0 | 0 | 0 | 0 | 0 | 0 | 0 |
| rno04540 Gap junction | 1 | 1 | 0 | 0 | 0 | 0 | 0 | 0 | 0 | 0 |
| rno04270 Vascular smooth muscle contraction | 1 | 1 | 0 | 0 | 0 | 0 | 0 | 0 | 0 | 0 |
| rno00561 Glycerolipid metabolism | 1 | 1 | 0 | 0 | 0 | 0 | 0 | 0 | 0 | 0 |
| rno04080 Neuroactive ligand-receptor interaction | 1 | 1 | 0 | 0 | 0 | 0 | 0 | 0 | 0 | 0 |
| rno04120 Ubiquitin mediated proteolysis | 1 | 0 | 0 | 0 | 1 | 0 | 0 | 0 | 0 | 0 |
| rno04621 NOD-like receptor signaling pathway | 1 | 0 | 0 | 0 | 1 | 0 | 0 | 0 | 0 | 0 |
| rno05214 Glioma | 1 | 0 | 0 | 0 | 0 | 0 | 0 | 0 | 0 | 1 |
| rno00071 Fatty acid metabolism | 1 | 0 | 0 | 0 | 0 | 1 | 0 | 0 | 0 | 0 |
| rno00140 Steroid hormone biosynthesis | 1 | 0 | 0 | 0 | 0 | 1 | 0 | 0 | 0 | 0 |
| rno04930 Type II diabetes mellitus | 1 | 1 | 0 | 0 | 0 | 0 | 0 | 0 | 0 | 0 |
| rno04350 TGF-beta signaling pathway | 1 | 0 | 0 | 0 | 1 | 0 | 0 | 0 | 0 | 0 |
| rno00785 Lipoic acid metabolism | 1 | 1 | 0 | 0 | 0 | 0 | 0 | 0 | 0 | 0 |
| rno04320 Dorso-ventral axis formation | 1 | 1 | 0 | 0 | 0 | 0 | 0 | 0 | 0 | 0 |
| rno04910 Insulin signaling pathway | 1 | 1 | 0 | 0 | 0 | 0 | 0 | 0 | 0 | 0 |
| rno04912 GnRH signaling pathway | 1 | 1 | 0 | 0 | 0 | 0 | 0 | 0 | 0 | 0 |
| rno05221 Acute myeloid leukemia | 1 | 1 | 0 | 0 | 0 | 0 | 0 | 0 | 0 | 0 |
| rno05410 Hypertrophic cardiomyopathy (HCM) | 1 | 1 | 0 | 0 | 0 | 0 | 0 | 0 | 0 | 0 |
| miRNA targeting different pathways | | 52 | 12 | 15 | 35 | 18 | 0 | 0 | 0 | 6 |

**Supplementary Table 6B**

| Pathways | Sum | rno-miR-129 | rno-miR-130a | rno-miR-130b | rno-miR-133a | rno-miR-138 | rno-miR-141 | rno-miR-216a | rno-miR-133b | rno-miR-136* | rno-miR-182 | rno-miR-3596d | rno-miR-3597-5p | rno-miR-3565 | rno-miR-3596c | rno-miR-3588 |
| --- | --- | --- | --- | --- | --- | --- | --- | --- | --- | --- | --- | --- | --- | --- | --- | --- |
| rno05200 Pathways in cancer | 9 | 1 | 1 | 1 | 1 | 1 | 1 | 1 | 1 | 0 | 1 | 0 | 0 | 0 | 0 | 0 |
| rno05212 Pancreatic cancer | 9 | 1 | 1 | 1 | 1 | 1 | 1 | 1 | 1 | 0 | 1 | 0 | 0 | 0 | 0 | 0 |
| rno04730 Long-term depression | 9 | 1 | 1 | 1 | 1 | 1 | 1 | 0 | 1 | 0 | 1 | 0 | 0 | 0 | 1 | 0 |
| rno05142 Chagas disease | 9 | 1 | 1 | 1 | 1 | 1 | 1 | 1 | 1 | 0 | 1 | 0 | 0 | 0 | 0 | 0 |
| rno04114 Oocyte meiosis | 8 | 1 | 1 | 1 | 1 | 0 | 1 | 0 | 1 | 0 | 1 | 0 | 0 | 0 | 1 | 0 |
| rno01100 Metabolic pathways | 8 | 1 | 1 | 1 | 1 | 0 | 1 | 1 | 1 | 0 | 1 | 0 | 0 | 0 | 0 | 0 |
| rno04010 MAPK signaling pathway | 8 | 1 | 1 | 1 | 1 | 1 | 1 | 0 | 1 | 0 | 1 | 0 | 0 | 0 | 0 | 0 |
| rno05219 Bladder cancer | 8 | 0 | 1 | 1 | 1 | 1 | 1 | 1 | 1 | 0 | 1 | 0 | 0 | 0 | 0 | 0 |
| rno04130 SNARE interactions in vesicular transport | 8 | 0 | 1 | 1 | 1 | 1 | 1 | 1 | 1 | 0 | 1 | 0 | 0 | 0 | 0 | 0 |
| rno04020 Calcium signaling pathway | 8 | 1 | 1 | 1 | 1 | 1 | 0 | 1 | 1 | 0 | 1 | 0 | 0 | 0 | 0 | 0 |
| rno04210 Apoptosis | 8 | 1 | 1 | 1 | 1 | 1 | 1 | 0 | 1 | 0 | 1 | 0 | 0 | 0 | 0 | 0 |
| rno04070 Phosphatidylinositol signaling system | 8 | 1 | 1 | 1 | 1 | 0 | 0 | 1 | 1 | 0 | 0 | 0 | 0 | 1 | 1 | 0 |
| rno04310 Wnt signaling pathway | 7 | 1 | 0 | 0 | 1 | 1 | 1 | 0 | 1 | 0 | 1 | 0 | 0 | 0 | 1 | 0 |
| rno04144 Endocytosis | 7 | 0 | 1 | 1 | 1 | 1 | 0 | 1 | 1 | 0 | 1 | 0 | 0 | 0 | 0 | 0 |
| rno05211 Renal cell carcinoma | 7 | 0 | 1 | 1 | 1 | 1 | 1 | 0 | 1 | 0 | 1 | 0 | 0 | 0 | 0 | 0 |
| rno04060 Cytokine-cytokine receptor interaction | 6 | 0 | 1 | 1 | 1 | 0 | 0 | 1 | 1 | 0 | 1 | 0 | 0 | 0 | 0 | 0 |
| rno04062 Chemokine signaling pathway | 6 | 1 | 1 | 1 | 0 | 1 | 0 | 1 | 0 | 0 | 1 | 0 | 0 | 0 | 0 | 0 |
| rno04722 Neurotrophin signaling pathway | 6 | 1 | 1 | 1 | 0 | 1 | 1 | 0 | 0 | 0 | 1 | 0 | 0 | 0 | 0 | 0 |
| rno04110 Cell cycle | 6 | 1 | 1 | 1 | 0 | 0 | 1 | 1 | 0 | 0 | 0 | 0 | 0 | 0 | 1 | 0 |
| rno04971 Gastric acid secretion | 5 | 1 | 1 | 1 | 1 | 0 | 0 | 0 | 1 | 0 | 0 | 0 | 0 | 0 | 0 | 0 |
| rno04012 ErbB signaling pathway | 5 | 0 | 1 | 1 | 0 | 1 | 1 | 0 | 0 | 0 | 1 | 0 | 0 | 0 | 0 | 0 |
| rno04370 VEGF signaling pathway | 5 | 1 | 0 | 0 | 1 | 1 | 1 | 0 | 1 | 0 | 0 | 0 | 0 | 0 | 0 | 0 |
| rno00100 Steroid biosynthesis | 5 | 1 | 0 | 0 | 1 | 0 | 1 | 0 | 1 | 0 | 1 | 0 | 0 | 0 | 0 | 0 |
| rno04670 Leukocyte transendothelial migration | 5 | 0 | 1 | 1 | 1 | 0 | 0 | 0 | 1 | 0 | 1 | 0 | 0 | 0 | 0 | 0 |
| rno04360 Axon guidance | 5 | 0 | 0 | 0 | 1 | 1 | 1 | 0 | 1 | 0 | 1 | 0 | 0 | 0 | 0 | 0 |
| rno04270 Vascular smooth muscle contraction | 5 | 1 | 0 | 0 | 1 | 1 | 0 | 0 | 1 | 0 | 0 | 0 | 0 | 0 | 1 | 0 |
| rno04530 Tight junction | 5 | 1 | 1 | 1 | 0 | 1 | 1 | 0 | 0 | 0 | 0 | 0 | 0 | 0 | 0 | 0 |
| rno04810 Regulation of actin cytoskeleton | 5 | 0 | 1 | 1 | 1 | 1 | 0 | 0 | 1 | 0 | 0 | 0 | 0 | 0 | 0 | 0 |
| rno04510 Focal adhesion | 4 | 0 | 0 | 0 | 1 | 1 | 0 | 0 | 1 | 0 | 1 | 0 | 0 | 0 | 0 | 0 |
| rno04540 Gap junction | 4 | 0 | 1 | 1 | 1 | 0 | 0 | 0 | 1 | 0 | 0 | 0 | 0 | 0 | 0 | 0 |
| rno04920 Adipocytokine signaling pathway | 4 | 1 | 0 | 0 | 1 | 0 | 0 | 0 | 1 | 0 | 1 | 0 | 0 | 0 | 0 | 0 |
| rno04622 RIG-I-like receptor signaling pathway | 4 | 1 | 1 | 1 | 0 | 1 | 0 | 0 | 0 | 0 | 0 | 0 | 0 | 0 | 0 | 0 |
| rno05215 Prostate cancer | 4 | 1 | 1 | 0 | 0 | 1 | 1 | 0 | 0 | 0 | 0 | 0 | 0 | 0 | 0 | 0 |
| rno05140 Leishmaniasis | 4 | 1 | 0 | 0 | 1 | 0 | 1 | 0 | 1 | 0 | 0 | 0 | 0 | 0 | 0 | 0 |
| rno05100 Bacterial invasion of epithelial cells | 4 | 0 | 1 | 1 | 1 | 0 | 0 | 0 | 1 | 0 | 0 | 0 | 0 | 0 | 0 | 0 |
| rno04666 Fc gamma R-mediated phagocytosis | 4 | 0 | 0 | 0 | 1 | 1 | 0 | 0 | 1 | 0 | 1 | 0 | 0 | 0 | 0 | 0 |
| rno04520 Adherens junction | 4 | 1 | 1 | 1 | 0 | 0 | 0 | 0 | 0 | 0 | 0 | 0 | 0 | 0 | 1 | 0 |
| rno04962 Vasopressin-regulated water reabsorption | 3 | 1 | 0 | 0 | 1 | 0 | 0 | 0 | 1 | 0 | 0 | 0 | 0 | 0 | 0 | 0 |
| rno05220 Chronic myeloid leukemia | 3 | 0 | 0 | 0 | 1 | 0 | 1 | 0 | 1 | 0 | 0 | 0 | 0 | 0 | 0 | 0 |
| rno04621 NOD-like receptor signaling pathway | 3 | 1 | 1 | 1 | 0 | 0 | 0 | 0 | 0 | 0 | 0 | 0 | 0 | 0 | 0 | 0 |
| rno04966 Collecting duct acid secretion | 3 | 0 | 1 | 1 | 0 | 0 | 1 | 0 | 0 | 0 | 0 | 0 | 0 | 0 | 0 | 0 |
| rno00380 Tryptophan metabolism | 3 | 1 | 1 | 1 | 0 | 0 | 0 | 0 | 0 | 0 | 0 | 0 | 0 | 0 | 0 | 0 |
| rno00564 Glycerophospholipid metabolism | 3 | 0 | 0 | 0 | 1 | 0 | 0 | 1 | 1 | 0 | 0 | 0 | 0 | 0 | 0 | 0 |
| rno00410 beta-Alanine metabolism | 3 | 1 | 1 | 1 | 0 | 0 | 0 | 0 | 0 | 0 | 0 | 0 | 0 | 0 | 0 | 0 |
| rno04916 Melanogenesis | 3 | 0 | 0 | 0 | 1 | 1 | 0 | 0 | 1 | 0 | 0 | 0 | 0 | 0 | 0 | 0 |
| rno04080 Neuroactive ligand-receptor interaction | 3 | 0 | 0 | 0 | 1 | 0 | 1 | 0 | 1 | 0 | 0 | 0 | 0 | 0 | 0 | 0 |
| rno00561 Glycerolipid metabolism | 3 | 1 | 0 | 0 | 1 | 0 | 0 | 0 | 1 | 0 | 0 | 0 | 0 | 0 | 0 | 0 |
| rno05214 Glioma | 3 | 0 | 1 | 1 | 0 | 1 | 0 | 0 | 0 | 0 | 0 | 0 | 0 | 0 | 0 | 0 |
| rno00071 Fatty acid metabolism | 3 | 1 | 1 | 1 | 0 | 0 | 0 | 0 | 0 | 0 | 0 | 0 | 0 | 0 | 0 | 0 |
| rno00310 Lysine degradation | 3 | 1 | 1 | 1 | 0 | 0 | 0 | 0 | 0 | 0 | 0 | 0 | 0 | 0 | 0 | 0 |
| rno00640 Propanoate metabolism | 3 | 1 | 1 | 1 | 0 | 0 | 0 | 0 | 0 | 0 | 0 | 0 | 0 | 0 | 0 | 0 |
| rno00903 Limonene and pinene degradation | 3 | 1 | 1 | 1 | 0 | 0 | 0 | 0 | 0 | 0 | 0 | 0 | 0 | 0 | 0 | 0 |
| rno04720 Long-term potentiation | 3 | 1 | 0 | 0 | 0 | 0 | 0 | 0 | 0 | 0 | 1 | 0 | 0 | 0 | 1 | 0 |
| rno04910 Insulin signaling pathway | 3 | 1 | 1 | 1 | 0 | 0 | 0 | 0 | 0 | 0 | 0 | 0 | 0 | 0 | 0 | 0 |
| rno00230 Purine metabolism | 3 | 0 | 0 | 0 | 1 | 0 | 0 | 1 | 1 | 0 | 0 | 0 | 0 | 0 | 0 | 0 |
| rno00562 Inositol phosphate metabolism | 2 | 1 | 0 | 0 | 0 | 0 | 0 | 1 | 0 | 0 | 0 | 0 | 0 | 0 | 0 | 0 |
| rno04141 Protein processing in endoplasmic reticulum | 2 | 1 | 0 | 0 | 0 | 0 | 0 | 0 | 0 | 0 | 1 | 0 | 0 | 0 | 0 | 0 |
| rno00140 Steroid hormone biosynthesis | 2 | 1 | 0 | 0 | 0 | 0 | 1 | 0 | 0 | 0 | 0 | 0 | 0 | 0 | 0 | 0 |
| rno04350 TGF-beta signaling pathway | 2 | 0 | 1 | 1 | 0 | 0 | 0 | 0 | 0 | 0 | 0 | 0 | 0 | 0 | 0 | 0 |
| rno00240 Pyrimidine metabolism | 2 | 0 | 0 | 0 | 1 | 0 | 0 | 0 | 1 | 0 | 0 | 0 | 0 | 0 | 0 | 0 |
| rno00120 Primary bile acid biosynthesis | 2 | 0 | 1 | 1 | 0 | 0 | 0 | 0 | 0 | 0 | 0 | 0 | 0 | 0 | 0 | 0 |
| rno00280 Valine, leucine and isoleucine degradation | 2 | 1 | 0 | 0 | 0 | 0 | 0 | 0 | 0 | 0 | 1 | 0 | 0 | 0 | 0 | 0 |
| rno04512 ECM-receptor interaction | 2 | 0 | 0 | 0 | 1 | 0 | 0 | 0 | 1 | 0 | 0 | 0 | 0 | 0 | 0 | 0 |
| rno00520 Amino sugar and nucleotide sugar metabolism | 2 | 0 | 1 | 1 | 0 | 0 | 0 | 0 | 0 | 0 | 0 | 0 | 0 | 0 | 0 | 0 |
| rno04146 Peroxisome | 2 | 0 | 1 | 1 | 0 | 0 | 0 | 0 | 0 | 0 | 0 | 0 | 0 | 0 | 0 | 0 |
| rno00514 O-Mannosyl glycan biosynthesis | 2 | 0 | 0 | 0 | 1 | 0 | 0 | 0 | 1 | 0 | 0 | 0 | 0 | 0 | 0 | 0 |
| rno02010 ABC transporters | 2 | 0 | 1 | 1 | 0 | 0 | 0 | 0 | 0 | 0 | 0 | 0 | 0 | 0 | 0 | 0 |
| rno03320 PPAR signaling pathway | 2 | 0 | 1 | 1 | 0 | 0 | 0 | 0 | 0 | 0 | 0 | 0 | 0 | 0 | 0 | 0 |
| rno05014 Amyotrophic lateral sclerosis (ALS) | 2 | 1 | 0 | 0 | 0 | 0 | 0 | 0 | 0 | 0 | 1 | 0 | 0 | 0 | 0 | 0 |
| rno00650 Butanoate metabolism | 2 | 0 | 1 | 1 | 0 | 0 | 0 | 0 | 0 | 0 | 0 | 0 | 0 | 0 | 0 | 0 |
| rno03030 DNA replication | 2 | 0 | 1 | 1 | 0 | 0 | 0 | 0 | 0 | 0 | 0 | 0 | 0 | 0 | 0 | 0 |
| rno04320 Dorso-ventral axis formation | 2 | 0 | 1 | 1 | 0 | 0 | 0 | 0 | 0 | 0 | 0 | 0 | 0 | 0 | 0 | 0 |
| rno00062 Fatty acid elongation in mitochondria | 2 | 0 | 1 | 1 | 0 | 0 | 0 | 0 | 0 | 0 | 0 | 0 | 0 | 0 | 0 | 0 |
| rno05221 Acute myeloid leukemia | 2 | 0 | 0 | 0 | 0 | 1 | 1 | 0 | 0 | 0 | 0 | 0 | 0 | 0 | 0 | 0 |
| rno05210 Colorectal cancer | 1 | 0 | 0 | 0 | 0 | 1 | 0 | 0 | 0 | 0 | 0 | 0 | 0 | 0 | 0 | 0 |
| rno00053 Ascorbate and aldarate metabolism | 1 | 0 | 0 | 0 | 0 | 0 | 1 | 0 | 0 | 0 | 0 | 0 | 0 | 0 | 0 | 0 |
| rno04120 Ubiquitin mediated proteolysis | 1 | 0 | 0 | 0 | 0 | 0 | 0 | 0 | 0 | 0 | 0 | 0 | 0 | 0 | 1 | 0 |
| rno00830 Retinol metabolism | 1 | 0 | 0 | 0 | 0 | 0 | 1 | 0 | 0 | 0 | 0 | 0 | 0 | 0 | 0 | 0 |
| rno04970 Salivary secretion | 1 | 0 | 0 | 0 | 0 | 0 | 0 | 0 | 0 | 0 | 0 | 0 | 0 | 0 | 1 | 0 |
| rno00920 Sulfur metabolism | 1 | 0 | 0 | 0 | 0 | 0 | 0 | 1 | 0 | 0 | 0 | 0 | 0 | 0 | 0 | 0 |
| rno00270 Cysteine and methionine metabolism | 1 | 1 | 0 | 0 | 0 | 0 | 0 | 0 | 0 | 0 | 0 | 0 | 0 | 0 | 0 | 0 |
| rno04662 B cell receptor signaling pathway | 1 | 0 | 0 | 0 | 0 | 1 | 0 | 0 | 0 | 0 | 0 | 0 | 0 | 0 | 0 | 0 |
| rno05213 Endometrial cancer | 1 | 0 | 0 | 0 | 0 | 1 | 0 | 0 | 0 | 0 | 0 | 0 | 0 | 0 | 0 | 0 |
| rno00130 Ubiquinone and other terpenoid-quinone biosynthesis | 1 | 0 | 0 | 0 | 0 | 1 | 0 | 0 | 0 | 0 | 0 | 0 | 0 | 0 | 0 | 0 |
| rno04950 Maturity onset diabetes of the young | 1 | 1 | 0 | 0 | 0 | 0 | 0 | 0 | 0 | 0 | 0 | 0 | 0 | 0 | 0 | 0 |
| rno00340 Histidine metabolism | 1 | 1 | 0 | 0 | 0 | 0 | 0 | 0 | 0 | 0 | 0 | 0 | 0 | 0 | 0 | 0 |
| rno00330 Arginine and proline metabolism | 1 | 1 | 0 | 0 | 0 | 0 | 0 | 0 | 0 | 0 | 0 | 0 | 0 | 0 | 0 | 0 |
| rno04960 Aldosterone-regulated sodium reabsorption | 1 | 0 | 0 | 0 | 0 | 0 | 0 | 0 | 0 | 0 | 0 | 0 | 0 | 0 | 1 | 0 |
| rno05222 Small cell lung cancer | 1 | 0 | 0 | 0 | 0 | 0 | 1 | 0 | 0 | 0 | 0 | 0 | 0 | 0 | 0 | 0 |
| rno05146 Amoebiasis | 1 | 1 | 0 | 0 | 0 | 0 | 0 | 0 | 0 | 0 | 0 | 0 | 0 | 0 | 0 | 0 |
| rno05223 Non-small cell lung cancer | 1 | 0 | 0 | 0 | 0 | 0 | 1 | 0 | 0 | 0 | 0 | 0 | 0 | 0 | 0 | 0 |
| rno04142 Lysosome | 1 | 0 | 0 | 0 | 0 | 0 | 1 | 0 | 0 | 0 | 0 | 0 | 0 | 0 | 0 | 0 |
| rno00601 Glycosphingolipid biosynthesis - lacto and neolacto series | 1 | 1 | 0 | 0 | 0 | 0 | 0 | 0 | 0 | 0 | 0 | 0 | 0 | 0 | 0 | 0 |
| rno05020 Prion diseases | 1 | 0 | 0 | 0 | 0 | 1 | 0 | 0 | 0 | 0 | 0 | 0 | 0 | 0 | 0 | 0 |
| rno05410 Hypertrophic cardiomyopathy (HCM) | 1 | 1 | 0 | 0 | 0 | 0 | 0 | 0 | 0 | 0 | 0 | 0 | 0 | 0 | 0 | 0 |
| miRNA targeting different pathways | | 47 | 48 | 47 | 39 | 32 | 31 | 16 | 39 | 0 | 28 | 0 | 0 | 1 | 11 | 0 |

**Supplementary Table 6C**

| Pathways | Sum | rno-miR-338 | rno-miR-19b | rno-miR-19a | rno-miR-30c | rno-miR-145 | rno-miR-653 | rno-miR-3556a | rno-miR-3596b | rno-miR-3590-5p |
| --- | --- | --- | --- | --- | --- | --- | --- | --- | --- | --- |
| GO:0050789~regulation of biological process | 8 | 1 | 1 | 1 | 1 | 1 | 1 | 1 | 0 | 1 |
| GO:0007275~multicellular organismal development | 8 | 1 | 1 | 1 | 1 | 1 | 1 | 1 | 0 | 1 |
| GO:0065007~biological regulation | 8 | 1 | 1 | 1 | 1 | 1 | 1 | 1 | 0 | 1 |
| GO:0032502~developmental process | 8 | 1 | 1 | 1 | 1 | 1 | 1 | 1 | 0 | 1 |
| GO:0030154~cell differentiation | 7 | 1 | 1 | 1 | 1 | 1 | 1 | 0 | 0 | 1 |
| GO:0051179~localization | 7 | 1 | 1 | 1 | 1 | 1 | 1 | 0 | 0 | 1 |
| GO:0007267~cell-cell signaling | 7 | 1 | 1 | 1 | 1 | 1 | 1 | 1 | 0 | 0 |
| GO:0007399~nervous system development | 7 | 1 | 1 | 1 | 1 | 1 | 1 | 0 | 0 | 1 |
| GO:0044238~primary metabolic process | 7 | 1 | 1 | 1 | 1 | 1 | 1 | 1 | 0 | 0 |
| GO:0019226~transmission of nerve impulse | 7 | 1 | 1 | 1 | 1 | 1 | 1 | 1 | 0 | 0 |
| GO:0048518~positive regulation of biological process | 7 | 1 | 1 | 1 | 1 | 1 | 1 | 0 | 0 | 1 |
| GO:0007242~intracellular signaling cascade | 7 | 1 | 1 | 1 | 1 | 1 | 1 | 0 | 0 | 1 |
| GO:0043687~post-translational protein modification | 7 | 1 | 1 | 1 | 1 | 1 | 1 | 0 | 0 | 1 |
| GO:0043283~biopolymer metabolic process | 7 | 1 | 1 | 1 | 1 | 1 | 1 | 1 | 0 | 0 |
| GO:0044237~cellular metabolic process | 7 | 1 | 1 | 1 | 1 | 1 | 1 | 1 | 0 | 0 |
| GO:0007049~cell cycle | 7 | 1 | 1 | 1 | 1 | 1 | 1 | 0 | 0 | 1 |
| GO:0006464~protein modification process | 7 | 1 | 1 | 1 | 1 | 1 | 1 | 0 | 0 | 1 |
| GO:0048731~system development | 7 | 1 | 1 | 1 | 1 | 1 | 1 | 0 | 0 | 1 |
| GO:0048869~cellular developmental process | 7 | 1 | 1 | 1 | 1 | 1 | 1 | 0 | 0 | 1 |
| GO:0048856~anatomical structure development | 7 | 1 | 1 | 1 | 1 | 1 | 1 | 0 | 0 | 1 |
| GO:0043412~biopolymer modification | 7 | 1 | 1 | 1 | 1 | 1 | 1 | 0 | 0 | 1 |
| GO:0043170~macromolecule metabolic process | 6 | 1 | 1 | 1 | 1 | 1 | 1 | 0 | 0 | 0 |
| GO:0006796~phosphate metabolic process | 6 | 1 | 1 | 1 | 1 | 1 | 1 | 0 | 0 | 0 |
| GO:0065009~regulation of a molecular function | 6 | 1 | 1 | 1 | 1 | 1 | 0 | 0 | 0 | 1 |
| GO:0016265~death | 6 | 1 | 1 | 1 | 1 | 1 | 1 | 0 | 0 | 0 |
| GO:0007264~small GTPase mediated signal transduction | 6 | 1 | 1 | 1 | 1 | 1 | 1 | 0 | 0 | 0 |
| GO:0006950~response to stress | 6 | 1 | 1 | 1 | 1 | 1 | 1 | 0 | 0 | 0 |
| GO:0016310~phosphorylation | 6 | 1 | 1 | 1 | 1 | 1 | 1 | 0 | 0 | 0 |
| GO:0000278~mitotic cell cycle | 6 | 1 | 1 | 1 | 1 | 1 | 0 | 0 | 0 | 1 |
| GO:0065008~regulation of biological quality | 6 | 1 | 1 | 1 | 1 | 1 | 1 | 0 | 0 | 0 |
| GO:0016311~dephosphorylation | 6 | 1 | 1 | 1 | 1 | 1 | 0 | 0 | 0 | 1 |
| GO:0016337~cell-cell adhesion | 6 | 1 | 1 | 1 | 0 | 1 | 1 | 0 | 1 | 0 |
| GO:0043085~positive regulation of catalytic activity | 6 | 1 | 1 | 1 | 1 | 1 | 0 | 0 | 0 | 1 |
| GO:0006793~phosphorus metabolic process | 6 | 1 | 1 | 1 | 1 | 1 | 1 | 0 | 0 | 0 |
| GO:0035295~tube development | 6 | 1 | 1 | 1 | 1 | 1 | 1 | 0 | 0 | 0 |
| GO:0007167~enzyme linked receptor protein signaling pathway | 6 | 1 | 1 | 1 | 1 | 1 | 0 | 0 | 0 | 1 |
| GO:0007155~cell adhesion | 6 | 1 | 1 | 1 | 1 | 1 | 1 | 0 | 0 | 0 |
| GO:0006629~lipid metabolic process | 6 | 1 | 1 | 1 | 1 | 1 | 0 | 0 | 0 | 1 |
| GO:0008283~cell proliferation | 6 | 1 | 1 | 1 | 1 | 1 | 1 | 0 | 0 | 0 |
| GO:0048519~negative regulation of biological process | 6 | 1 | 1 | 1 | 1 | 1 | 1 | 0 | 0 | 0 |
| GO:0009719~response to endogenous stimulus | 6 | 1 | 1 | 1 | 1 | 1 | 1 | 0 | 0 | 0 |
| GO:0022610~biological adhesion | 6 | 1 | 1 | 1 | 1 | 1 | 1 | 0 | 0 | 0 |
| GO:0007243~protein kinase cascade | 5 | 1 | 1 | 1 | 1 | 1 | 0 | 0 | 0 | 0 |
| GO:0016043~cellular component organization and biogenesis | 5 | 1 | 1 | 1 | 1 | 1 | 0 | 0 | 0 | 0 |
| GO:0050801~ion homeostasis | 5 | 1 | 1 | 1 | 1 | 1 | 0 | 0 | 0 | 0 |
| GO:0055065~metal ion homeostasis | 5 | 1 | 1 | 1 | 1 | 1 | 0 | 0 | 0 | 0 |
| GO:0007610~behavior | 5 | 1 | 1 | 1 | 1 | 1 | 0 | 0 | 0 | 0 |
| GO:0008104~protein localization | 5 | 1 | 1 | 1 | 1 | 1 | 0 | 0 | 0 | 0 |
| GO:0048771~tissue remodeling | 5 | 1 | 1 | 1 | 1 | 1 | 0 | 0 | 0 | 0 |
| GO:0042592~homeostatic process | 5 | 1 | 1 | 1 | 1 | 1 | 0 | 0 | 0 | 0 |
| GO:0044260~cellular macromolecule metabolic process | 5 | 1 | 1 | 1 | 1 | 1 | 0 | 0 | 0 | 0 |
| GO:0006519~amino acid and derivative metabolic process | 5 | 1 | 1 | 1 | 1 | 1 | 0 | 0 | 0 | 0 |
| GO:0009605~response to external stimulus | 5 | 1 | 1 | 1 | 1 | 1 | 0 | 0 | 0 | 0 |
| GO:0008152~metabolic process | 5 | 1 | 1 | 1 | 1 | 1 | 0 | 0 | 0 | 0 |
| GO:0019752~carboxylic acid metabolic process | 5 | 1 | 1 | 1 | 1 | 1 | 0 | 0 | 0 | 0 |
| GO:0048878~chemical homeostasis | 5 | 1 | 1 | 1 | 1 | 1 | 0 | 0 | 0 | 0 |
| GO:0006575~amino acid derivative metabolic process | 5 | 1 | 1 | 1 | 1 | 1 | 0 | 0 | 0 | 0 |
| GO:0051336~regulation of hydrolase activity | 5 | 1 | 1 | 1 | 1 | 1 | 0 | 0 | 0 | 0 |
| GO:0042493~response to drug | 5 | 1 | 1 | 1 | 1 | 1 | 0 | 0 | 0 | 0 |
| GO:0007010~cytoskeleton organization and biogenesis | 5 | 1 | 1 | 1 | 1 | 1 | 0 | 0 | 0 | 0 |
| GO:0051704~multi-organism process | 5 | 1 | 1 | 1 | 1 | 1 | 0 | 0 | 0 | 0 |
| GO:0033036~macromolecule localization | 5 | 1 | 1 | 1 | 1 | 1 | 0 | 0 | 0 | 0 |
| GO:0044267~cellular protein metabolic process | 5 | 1 | 1 | 1 | 1 | 1 | 0 | 0 | 0 | 0 |
| GO:0051301~cell division | 5 | 1 | 1 | 1 | 1 | 1 | 0 | 0 | 0 | 0 |
| GO:0009987~cellular process | 5 | 1 | 1 | 1 | 1 | 1 | 0 | 0 | 0 | 0 |
| GO:0048015~phosphoinositide-mediated signaling | 5 | 1 | 1 | 1 | 1 | 1 | 0 | 0 | 0 | 0 |
| GO:0009628~response to abiotic stimulus | 5 | 1 | 1 | 1 | 1 | 1 | 0 | 0 | 0 | 0 |
| GO:0016070~RNA metabolic process | 5 | 1 | 1 | 1 | 1 | 1 | 0 | 0 | 0 | 0 |
| GO:0032787~monocarboxylic acid metabolic process | 5 | 1 | 1 | 1 | 1 | 1 | 0 | 0 | 0 | 0 |
| GO:0016044~membrane organization and biogenesis | 5 | 1 | 1 | 1 | 1 | 1 | 0 | 0 | 0 | 0 |
| GO:0009611~response to wounding | 5 | 1 | 1 | 1 | 1 | 1 | 0 | 0 | 0 | 0 |
| GO:0019538~protein metabolic process | 5 | 1 | 1 | 1 | 1 | 1 | 0 | 0 | 0 | 0 |
| GO:0051338~regulation of transferase activity | 5 | 1 | 1 | 1 | 1 | 1 | 0 | 0 | 0 | 0 |
| GO:0040007~growth | 5 | 1 | 1 | 1 | 1 | 1 | 0 | 0 | 0 | 0 |
| GO:0010467~gene expression | 5 | 1 | 1 | 1 | 1 | 1 | 0 | 0 | 0 | 0 |
| GO:0006996~organelle organization and biogenesis | 5 | 1 | 1 | 1 | 1 | 1 | 0 | 0 | 0 | 0 |
| GO:0000003~reproduction | 5 | 1 | 1 | 1 | 1 | 1 | 0 | 0 | 0 | 0 |
| GO:0006807~nitrogen compound metabolic process | 5 | 1 | 1 | 1 | 1 | 1 | 0 | 0 | 0 | 0 |
| GO:0022607~cellular component assembly | 5 | 1 | 1 | 1 | 1 | 1 | 0 | 0 | 0 | 0 |
| GO:0046849~bone remodeling | 5 | 1 | 1 | 1 | 1 | 1 | 0 | 0 | 0 | 0 |
| GO:0006139~nucleobase, nucleoside, nucleotide and nucleic acid metabolic process | 5 | 1 | 1 | 1 | 1 | 1 | 0 | 0 | 0 | 0 |
| GO:0043549~regulation of kinase activity | 5 | 1 | 1 | 1 | 1 | 1 | 0 | 0 | 0 | 0 |
| GO:0006944~membrane fusion | 5 | 1 | 1 | 1 | 1 | 1 | 0 | 0 | 0 | 0 |
| GO:0007169~transmembrane receptor protein tyrosine kinase signaling pathway | 5 | 1 | 1 | 1 | 1 | 1 | 0 | 0 | 0 | 0 |
| GO:0007626~locomotory behavior | 5 | 1 | 1 | 1 | 1 | 1 | 0 | 0 | 0 | 0 |
| GO:0009607~response to biotic stimulus | 5 | 1 | 1 | 1 | 1 | 1 | 0 | 0 | 0 | 0 |
| GO:0002376~immune system process | 5 | 1 | 1 | 1 | 1 | 1 | 0 | 0 | 0 | 0 |
| GO:0032774~RNA biosynthetic process | 5 | 1 | 1 | 1 | 1 | 1 | 0 | 0 | 0 | 0 |
| GO:0000165~MAPKKK cascade | 5 | 1 | 1 | 1 | 1 | 1 | 0 | 0 | 0 | 0 |
| GO:0055080~cation homeostasis | 5 | 1 | 1 | 1 | 1 | 1 | 0 | 0 | 0 | 0 |
| GO:0007005~mitochondrion organization and biogenesis | 5 | 1 | 1 | 1 | 1 | 1 | 0 | 0 | 0 | 0 |
| GO:0009100~glycoprotein metabolic process | 5 | 1 | 1 | 1 | 1 | 1 | 0 | 0 | 0 | 0 |
| GO:0050790~regulation of catalytic activity | 5 | 1 | 1 | 1 | 1 | 1 | 0 | 0 | 0 | 0 |
| GO:0006082~organic acid metabolic process | 5 | 1 | 1 | 1 | 1 | 1 | 0 | 0 | 0 | 0 |
| GO:0051674~localization of cell | 5 | 1 | 1 | 1 | 1 | 1 | 0 | 0 | 0 | 0 |
| GO:0018193~peptidyl-amino acid modification | 5 | 1 | 1 | 1 | 1 | 1 | 0 | 0 | 0 | 0 |
| GO:0051345~positive regulation of hydrolase activity | 5 | 1 | 1 | 1 | 1 | 1 | 0 | 0 | 0 | 0 |
| GO:0009308~amine metabolic process | 5 | 1 | 1 | 1 | 1 | 1 | 0 | 0 | 0 | 0 |
| GO:0009725~response to hormone stimulus | 5 | 1 | 1 | 1 | 1 | 1 | 0 | 0 | 0 | 0 |
| GO:0006259~DNA metabolic process | 4 | 0 | 1 | 1 | 1 | 1 | 0 | 0 | 0 | 0 |
| GO:0043086~negative regulation of catalytic activity | 4 | 1 | 1 | 1 | 0 | 1 | 0 | 0 | 0 | 0 |
| GO:0001666~response to hypoxia | 4 | 1 | 1 | 1 | 0 | 1 | 0 | 0 | 0 | 0 |
| GO:0010038~response to metal ion | 4 | 0 | 1 | 1 | 1 | 1 | 0 | 0 | 0 | 0 |
| GO:0055086~nucleobase, nucleoside and nucleotide metabolic process | 4 | 1 | 1 | 1 | 0 | 1 | 0 | 0 | 0 | 0 |
| GO:0010035~response to inorganic substance | 4 | 0 | 1 | 1 | 1 | 1 | 0 | 0 | 0 | 0 |
| GO:0045859~regulation of protein kinase activity | 4 | 1 | 1 | 1 | 0 | 1 | 0 | 0 | 0 | 0 |
| GO:0006974~response to DNA damage stimulus | 4 | 0 | 1 | 1 | 1 | 1 | 0 | 0 | 0 | 0 |
| GO:0031279~regulation of cyclase activity | 4 | 1 | 1 | 1 | 0 | 1 | 0 | 0 | 0 | 0 |
| GO:0042060~wound healing | 4 | 1 | 1 | 1 | 0 | 1 | 0 | 0 | 0 | 0 |
| GO:0006512~ubiquitin cycle | 4 | 0 | 1 | 1 | 1 | 1 | 0 | 0 | 0 | 0 |
| GO:0016567~protein ubiquitination | 4 | 0 | 1 | 1 | 1 | 1 | 0 | 0 | 0 | 0 |
| GO:0032504~multicellular organism reproduction | 4 | 1 | 1 | 1 | 0 | 1 | 0 | 0 | 0 | 0 |
| GO:0006790~sulfur metabolic process | 4 | 0 | 1 | 1 | 1 | 1 | 0 | 0 | 0 | 0 |
| GO:0006066~alcohol metabolic process | 4 | 1 | 1 | 1 | 0 | 1 | 0 | 0 | 0 | 0 |
| GO:0051339~regulation of lyase activity | 4 | 1 | 1 | 1 | 0 | 1 | 0 | 0 | 0 | 0 |
| GO:0007265~Ras protein signal transduction | 4 | 1 | 1 | 1 | 0 | 1 | 0 | 0 | 0 | 0 |
| GO:0031667~response to nutrient levels | 4 | 1 | 1 | 1 | 0 | 1 | 0 | 0 | 0 | 0 |
| GO:0032446~protein modification by small protein conjugation | 4 | 0 | 1 | 1 | 1 | 1 | 0 | 0 | 0 | 0 |
| GO:0006725~aromatic compound metabolic process | 4 | 1 | 1 | 1 | 0 | 1 | 0 | 0 | 0 | 0 |
| GO:0019932~second-messenger-mediated signaling | 4 | 1 | 1 | 1 | 0 | 1 | 0 | 0 | 0 | 0 |
| GO:0043413~biopolymer glycosylation | 4 | 0 | 1 | 1 | 1 | 1 | 0 | 0 | 0 | 0 |
| GO:0007249~I-kappaB kinase/NF-kappaB cascade | 3 | 1 | 0 | 0 | 1 | 1 | 0 | 0 | 0 | 0 |
| GO:0044248~cellular catabolic process | 3 | 1 | 0 | 0 | 1 | 1 | 0 | 0 | 0 | 0 |
| GO:0006260~DNA replication | 3 | 0 | 1 | 1 | 1 | 0 | 0 | 0 | 0 | 0 |
| GO:0003013~circulatory system process | 3 | 1 | 0 | 0 | 1 | 1 | 0 | 0 | 0 | 0 |
| GO:0016071~mRNA metabolic process | 3 | 0 | 1 | 1 | 1 | 0 | 0 | 0 | 0 | 0 |
| GO:0007611~learning and/or memory | 3 | 1 | 0 | 0 | 1 | 1 | 0 | 0 | 0 | 0 |
| GO:0055074~calcium ion homeostasis | 3 | 1 | 0 | 0 | 1 | 1 | 0 | 0 | 0 | 0 |
| GO:0008015~blood circulation | 3 | 1 | 0 | 0 | 1 | 1 | 0 | 0 | 0 | 0 |
| GO:0055066~di-, tri-valent inorganic cation homeostasis | 3 | 1 | 0 | 0 | 1 | 1 | 0 | 0 | 0 | 0 |
| GO:0009266~response to temperature stimulus | 3 | 1 | 0 | 0 | 1 | 1 | 0 | 0 | 0 | 0 |
| GO:0010033~response to organic substance | 3 | 1 | 0 | 0 | 1 | 1 | 0 | 0 | 0 | 0 |
| GO:0009117~nucleotide metabolic process | 3 | 1 | 1 | 1 | 0 | 0 | 0 | 0 | 0 | 0 |
| GO:0010324~membrane invagination | 3 | 1 | 0 | 0 | 1 | 1 | 0 | 0 | 0 | 0 |
| GO:0007156~homophilic cell adhesion | 3 | 1 | 1 | 1 | 0 | 0 | 0 | 0 | 0 | 0 |
| GO:0009056~catabolic process | 3 | 1 | 0 | 0 | 1 | 1 | 0 | 0 | 0 | 0 |
| GO:0006952~defense response | 3 | 1 | 0 | 0 | 1 | 1 | 0 | 0 | 0 | 0 |
| GO:0030029~actin filament-based process | 2 | 0 | 0 | 0 | 1 | 1 | 0 | 0 | 0 | 0 |
| GO:0003018~vascular process in circulatory system | 2 | 1 | 0 | 0 | 0 | 1 | 0 | 0 | 0 | 0 |
| GO:0001816~cytokine production | 2 | 1 | 0 | 0 | 1 | 0 | 0 | 0 | 0 | 0 |
| GO:0019933~cAMP-mediated signaling | 2 | 1 | 0 | 0 | 0 | 1 | 0 | 0 | 0 | 0 |
| GO:0006936~muscle contraction | 2 | 0 | 0 | 0 | 1 | 1 | 0 | 0 | 0 | 0 |
| GO:0050953~sensory perception of light stimulus | 2 | 0 | 0 | 1 | 0 | 1 | 0 | 0 | 0 | 0 |
| GO:0048511~rhythmic process | 2 | 1 | 0 | 0 | 0 | 1 | 0 | 0 | 0 | 0 |
| GO:0048736~appendage development | 2 | 0 | 0 | 0 | 1 | 1 | 0 | 0 | 0 | 0 |
| GO:0045860~positive regulation of protein kinase activity | 2 | 1 | 0 | 0 | 0 | 1 | 0 | 0 | 0 | 0 |
| GO:0019935~cyclic-nucleotide-mediated signaling | 2 | 1 | 0 | 0 | 0 | 1 | 0 | 0 | 0 | 0 |
| GO:0050673~epithelial cell proliferation | 2 | 1 | 0 | 0 | 0 | 1 | 0 | 0 | 0 | 0 |
| GO:0021700~developmental maturation | 2 | 1 | 0 | 0 | 0 | 1 | 0 | 0 | 0 | 0 |
| GO:0003012~muscle system process | 2 | 0 | 0 | 0 | 1 | 1 | 0 | 0 | 0 | 0 |
| GO:0009582~detection of abiotic stimulus | 2 | 1 | 0 | 0 | 0 | 1 | 0 | 0 | 0 | 0 |
| GO:0006979~response to oxidative stress | 2 | 0 | 0 | 0 | 1 | 1 | 0 | 0 | 0 | 0 |
| GO:0030036~actin cytoskeleton organization and biogenesis | 2 | 0 | 0 | 0 | 1 | 1 | 0 | 0 | 0 | 0 |
| GO:0009058~biosynthetic process | 2 | 1 | 0 | 0 | 0 | 1 | 0 | 0 | 0 | 0 |
| GO:0009314~response to radiation | 2 | 1 | 0 | 0 | 0 | 1 | 0 | 0 | 0 | 0 |
| GO:0051347~positive regulation of transferase activity | 2 | 1 | 0 | 0 | 0 | 1 | 0 | 0 | 0 | 0 |
| GO:0032501~multicellular organismal process | 2 | 1 | 0 | 0 | 0 | 1 | 0 | 0 | 0 | 0 |
| GO:0003015~heart process | 2 | 1 | 0 | 0 | 1 | 0 | 0 | 0 | 0 | 0 |
| GO:0009991~response to extracellular stimulus | 2 | 1 | 0 | 0 | 0 | 1 | 0 | 0 | 0 | 0 |
| GO:0051348~negative regulation of transferase activity | 2 | 0 | 1 | 1 | 0 | 0 | 0 | 0 | 0 | 0 |
| GO:0005975~carbohydrate metabolic process | 2 | 1 | 0 | 0 | 0 | 1 | 0 | 0 | 0 | 0 |
| GO:0043285~biopolymer catabolic process | 2 | 1 | 0 | 0 | 1 | 0 | 0 | 0 | 0 | 0 |
| GO:0060047~heart contraction | 2 | 1 | 0 | 0 | 1 | 0 | 0 | 0 | 0 | 0 |
| GO:0009416~response to light stimulus | 2 | 1 | 0 | 0 | 0 | 1 | 0 | 0 | 0 | 0 |
| GO:0001775~cell activation | 2 | 1 | 0 | 0 | 0 | 1 | 0 | 0 | 0 | 0 |
| GO:0000226~microtubule cytoskeleton organization and biogenesis | 2 | 0 | 0 | 0 | 1 | 1 | 0 | 0 | 0 | 0 |
| GO:0007601~visual perception | 2 | 0 | 0 | 1 | 0 | 1 | 0 | 0 | 0 | 0 |
| GO:0043434~response to peptide hormone stimulus | 2 | 1 | 0 | 0 | 0 | 1 | 0 | 0 | 0 | 0 |
| GO:0009581~detection of external stimulus | 2 | 1 | 0 | 0 | 0 | 1 | 0 | 0 | 0 | 0 |
| GO:0060173~limb development | 2 | 0 | 0 | 0 | 1 | 1 | 0 | 0 | 0 | 0 |
| GO:0033674~positive regulation of kinase activity | 2 | 1 | 0 | 0 | 0 | 1 | 0 | 0 | 0 | 0 |
| GO:0007179~transforming growth factor beta receptor signaling pathway | 1 | 0 | 0 | 0 | 1 | 0 | 0 | 0 | 0 | 0 |
| GO:0044262~cellular carbohydrate metabolic process | 1 | 1 | 0 | 0 | 0 | 0 | 0 | 0 | 0 | 0 |
| GO:0008213~protein amino acid alkylation | 1 | 0 | 0 | 0 | 1 | 0 | 0 | 0 | 0 | 0 |
| GO:0006732~coenzyme metabolic process | 1 | 0 | 0 | 0 | 0 | 1 | 0 | 0 | 0 | 0 |
| GO:0006457~protein folding | 1 | 0 | 0 | 0 | 0 | 1 | 0 | 0 | 0 | 0 |
| GO:0032943~mononuclear cell proliferation | 1 | 1 | 0 | 0 | 0 | 0 | 0 | 0 | 0 | 0 |
| GO:0007283~spermatogenesis | 1 | 0 | 0 | 0 | 0 | 1 | 0 | 0 | 0 | 0 |
| GO:0006730~one-carbon compound metabolic process | 1 | 0 | 0 | 0 | 1 | 0 | 0 | 0 | 0 | 0 |
| GO:0006091~generation of precursor metabolites and energy | 1 | 0 | 0 | 0 | 0 | 1 | 0 | 0 | 0 | 0 |
| GO:0048232~male gamete generation | 1 | 0 | 0 | 0 | 0 | 1 | 0 | 0 | 0 | 0 |
| GO:0016053~organic acid biosynthetic process | 1 | 1 | 0 | 0 | 0 | 0 | 0 | 0 | 0 | 0 |
| GO:0043405~regulation of MAP kinase activity | 1 | 0 | 0 | 0 | 0 | 1 | 0 | 0 | 0 | 0 |
| GO:0042157~lipoprotein metabolic process | 1 | 0 | 0 | 0 | 0 | 1 | 0 | 0 | 0 | 0 |
| GO:0006118~electron transport | 1 | 0 | 0 | 0 | 0 | 1 | 0 | 0 | 0 | 0 |
| GO:0030534~adult behavior | 1 | 1 | 0 | 0 | 0 | 0 | 0 | 0 | 0 | 0 |
| GO:0016051~carbohydrate biosynthetic process | 1 | 1 | 0 | 0 | 0 | 0 | 0 | 0 | 0 | 0 |
| GO:0001655~urogenital system development | 1 | 0 | 0 | 0 | 1 | 0 | 0 | 0 | 0 | 0 |
| GO:0043284~biopolymer biosynthetic process | 1 | 1 | 0 | 0 | 0 | 0 | 0 | 0 | 0 | 0 |
| GO:0007612~learning | 1 | 1 | 0 | 0 | 0 | 0 | 0 | 0 | 0 | 0 |
| GO:0046394~carboxylic acid biosynthetic process | 1 | 1 | 0 | 0 | 0 | 0 | 0 | 0 | 0 | 0 |
| GO:0019953~sexual reproduction | 1 | 0 | 0 | 0 | 0 | 1 | 0 | 0 | 0 | 0 |
| GO:0016485~protein processing | 1 | 1 | 0 | 0 | 0 | 0 | 0 | 0 | 0 | 0 |
| GO:0046483~heterocycle metabolic process | 1 | 0 | 0 | 0 | 0 | 1 | 0 | 0 | 0 | 0 |
| GO:0008037~cell recognition | 1 | 0 | 0 | 0 | 0 | 1 | 0 | 0 | 0 | 0 |
| GO:0007276~gamete generation | 1 | 0 | 0 | 0 | 0 | 1 | 0 | 0 | 0 | 0 |
| GO:0018212~peptidyl-tyrosine modification | 1 | 1 | 0 | 0 | 0 | 0 | 0 | 0 | 0 | 0 |
| GO:0035264~multicellular organism growth | 1 | 0 | 0 | 0 | 0 | 1 | 0 | 0 | 0 | 0 |
| GO:0016540~protein autoprocessing | 1 | 1 | 0 | 0 | 0 | 0 | 0 | 0 | 0 | 0 |
| GO:0006399~tRNA metabolic process | 1 | 0 | 0 | 0 | 0 | 1 | 0 | 0 | 0 | 0 |
| GO:0048545~response to steroid hormone stimulus | 1 | 0 | 0 | 0 | 0 | 1 | 0 | 0 | 0 | 0 |
| GO:0031589~cell-substrate adhesion | 1 | 0 | 0 | 0 | 0 | 1 | 0 | 0 | 0 | 0 |
| GO:0043632~modification-dependent macromolecule catabolic process | 1 | 0 | 0 | 0 | 1 | 0 | 0 | 0 | 0 | 0 |
| GO:0043414~biopolymer methylation | 1 | 0 | 0 | 0 | 1 | 0 | 0 | 0 | 0 | 0 |
| GO:0050817~coagulation | 1 | 0 | 0 | 1 | 0 | 0 | 0 | 0 | 0 | 0 |
| GO:0000910~cytokinesis | 1 | 0 | 0 | 0 | 1 | 0 | 0 | 0 | 0 | 0 |
| GO:0030522~intracellular receptor-mediated signaling pathway | 1 | 0 | 0 | 0 | 1 | 0 | 0 | 0 | 0 | 0 |
| GO:0040011~locomotion | 1 | 0 | 0 | 0 | 0 | 1 | 0 | 0 | 0 | 0 |
| GO:0007631~feeding behavior | 1 | 0 | 0 | 0 | 1 | 0 | 0 | 0 | 0 | 0 |
| GO:0006955~immune response | 1 | 0 | 0 | 0 | 0 | 1 | 0 | 0 | 0 | 0 |
| GO:0019233~sensory perception of pain | 1 | 0 | 0 | 0 | 1 | 0 | 0 | 0 | 0 | 0 |
| GO:0007160~cell-matrix adhesion | 1 | 0 | 0 | 0 | 0 | 1 | 0 | 0 | 0 | 0 |
| GO:0007178~transmembrane receptor protein serine/threonine kinase signaling pathway | 1 | 0 | 0 | 0 | 1 | 0 | 0 | 0 | 0 | 0 |
| GO:0006084~acetyl-CoA metabolic process | 1 | 1 | 0 | 0 | 0 | 0 | 0 | 0 | 0 | 0 |
| GO:0014070~response to organic cyclic substance | 1 | 1 | 0 | 0 | 0 | 0 | 0 | 0 | 0 | 0 |
| GO:0042446~hormone biosynthetic process | 1 | 1 | 0 | 0 | 0 | 0 | 0 | 0 | 0 | 0 |
| GO:0019221~cytokine and chemokine mediated signaling pathway | 1 | 0 | 0 | 0 | 1 | 0 | 0 | 0 | 0 | 0 |
| GO:0005976~polysaccharide metabolic process | 1 | 1 | 0 | 0 | 0 | 0 | 0 | 0 | 0 | 0 |
| GO:0043406~positive regulation of MAP kinase activity | 1 | 0 | 0 | 0 | 0 | 1 | 0 | 0 | 0 | 0 |
| miRNA targeting different GOBPs | | 164 | 126 | 129 | 145 | 182 | 36 | 9 | 1 | 22 |

**Supplementary Table 6D**

| Pathways | Sum | rno-miR-129 | rno-miR-130a | rno-miR-130b | rno-miR-133a | rno-miR-138 | rno-miR-141 | rno-miR-216a | rno-miR-133b | rno-miR-136* | rno-miR-182 | rno-miR-3596d | rno-miR-3597-5p | rno-miR-3565 | rno-miR-3596c | rno-miR-3588 |
| --- | --- | --- | --- | --- | --- | --- | --- | --- | --- | --- | --- | --- | --- | --- | --- | --- |
| GO:0050789~regulation of biological process | 14 | 1 | 1 | 1 | 1 | 1 | 1 | 1 | 1 | 1 | 1 | 1 | 0 | 1 | 1 | 1 |
| GO:0065007~biological regulation | 14 | 1 | 1 | 1 | 1 | 1 | 1 | 1 | 1 | 1 | 1 | 1 | 0 | 1 | 1 | 1 |
| GO:0007275~multicellular organismal development | 14 | 1 | 1 | 1 | 1 | 1 | 1 | 1 | 1 | 1 | 1 | 1 | 1 | 0 | 1 | 1 |
| GO:0032502~developmental process | 13 | 1 | 1 | 1 | 1 | 1 | 1 | 1 | 1 | 1 | 1 | 1 | 0 | 0 | 1 | 1 |
| GO:0048731~system development | 13 | 1 | 1 | 1 | 1 | 1 | 1 | 1 | 1 | 1 | 1 | 1 | 0 | 0 | 1 | 1 |
| GO:0048856~anatomical structure development | 13 | 1 | 1 | 1 | 1 | 1 | 1 | 1 | 1 | 1 | 1 | 1 | 0 | 0 | 1 | 1 |
| GO:0016043~cellular component organization and biogenesis | 12 | 1 | 1 | 1 | 1 | 1 | 1 | 1 | 1 | 0 | 1 | 0 | 0 | 1 | 1 | 1 |
| GO:0030154~cell differentiation | 12 | 1 | 1 | 1 | 1 | 1 | 1 | 1 | 1 | 0 | 1 | 1 | 0 | 0 | 1 | 1 |
| GO:0051179~localization | 12 | 1 | 1 | 1 | 1 | 1 | 1 | 1 | 1 | 0 | 1 | 1 | 0 | 0 | 1 | 1 |
| GO:0048869~cellular developmental process | 12 | 1 | 1 | 1 | 1 | 1 | 1 | 1 | 1 | 0 | 1 | 1 | 0 | 0 | 1 | 1 |
| GO:0007399~nervous system development | 12 | 1 | 1 | 1 | 1 | 1 | 1 | 1 | 1 | 0 | 1 | 1 | 0 | 0 | 1 | 1 |
| GO:0048518~positive regulation of biological process | 12 | 1 | 1 | 1 | 1 | 1 | 1 | 1 | 1 | 1 | 1 | 1 | 0 | 0 | 0 | 1 |
| GO:0008283~cell proliferation | 12 | 1 | 1 | 1 | 1 | 1 | 1 | 1 | 1 | 1 | 1 | 0 | 0 | 1 | 0 | 1 |
| GO:0051674~localization of cell | 12 | 1 | 1 | 1 | 1 | 1 | 1 | 1 | 1 | 0 | 1 | 1 | 0 | 0 | 1 | 1 |
| GO:0048519~negative regulation of biological process | 12 | 1 | 1 | 1 | 1 | 1 | 1 | 1 | 1 | 0 | 1 | 1 | 0 | 1 | 0 | 1 |
| GO:0007242~intracellular signaling cascade | 11 | 1 | 1 | 1 | 1 | 1 | 1 | 1 | 1 | 0 | 1 | 1 | 0 | 0 | 0 | 1 |
| GO:0043283~biopolymer metabolic process | 11 | 1 | 1 | 1 | 1 | 1 | 1 | 1 | 1 | 0 | 1 | 1 | 0 | 0 | 0 | 1 |
| GO:0008104~protein localization | 10 | 1 | 1 | 1 | 1 | 1 | 1 | 1 | 1 | 0 | 1 | 0 | 0 | 0 | 0 | 1 |
| GO:0033036~macromolecule localization | 10 | 1 | 1 | 1 | 1 | 1 | 1 | 1 | 1 | 0 | 1 | 0 | 0 | 0 | 0 | 1 |
| GO:0043170~macromolecule metabolic process | 10 | 1 | 1 | 1 | 1 | 1 | 1 | 1 | 1 | 0 | 1 | 1 | 0 | 0 | 0 | 0 |
| GO:0006796~phosphate metabolic process | 10 | 1 | 1 | 1 | 1 | 1 | 1 | 1 | 1 | 0 | 1 | 0 | 0 | 0 | 0 | 1 |
| GO:0044238~primary metabolic process | 10 | 1 | 1 | 1 | 1 | 1 | 1 | 1 | 1 | 0 | 1 | 1 | 0 | 0 | 0 | 0 |
| GO:0000278~mitotic cell cycle | 10 | 1 | 1 | 1 | 1 | 1 | 1 | 1 | 1 | 0 | 1 | 0 | 0 | 0 | 1 | 0 |
| GO:0065008~regulation of biological quality | 10 | 1 | 1 | 1 | 1 | 1 | 1 | 1 | 1 | 1 | 1 | 0 | 0 | 0 | 0 | 0 |
| GO:0043687~post-translational protein modification | 10 | 1 | 1 | 1 | 1 | 1 | 1 | 1 | 1 | 0 | 1 | 0 | 0 | 0 | 0 | 1 |
| GO:0016070~RNA metabolic process | 10 | 1 | 1 | 1 | 1 | 1 | 1 | 1 | 1 | 0 | 1 | 1 | 0 | 0 | 0 | 0 |
| GO:0044237~cellular metabolic process | 10 | 1 | 1 | 1 | 1 | 1 | 1 | 1 | 1 | 0 | 1 | 1 | 0 | 0 | 0 | 0 |
| GO:0010467~gene expression | 10 | 1 | 1 | 1 | 1 | 1 | 1 | 1 | 1 | 0 | 1 | 1 | 0 | 0 | 0 | 0 |
| GO:0007049~cell cycle | 10 | 1 | 1 | 1 | 1 | 1 | 1 | 1 | 1 | 0 | 1 | 1 | 0 | 0 | 0 | 0 |
| GO:0006464~protein modification process | 10 | 1 | 1 | 1 | 1 | 1 | 1 | 1 | 1 | 0 | 1 | 0 | 0 | 0 | 0 | 1 |
| GO:0006793~phosphorus metabolic process | 10 | 1 | 1 | 1 | 1 | 1 | 1 | 1 | 1 | 0 | 1 | 0 | 0 | 0 | 0 | 1 |
| GO:0006139~nucleobase, nucleoside, nucleotide and nucleic acid metabolic process | 10 | 1 | 1 | 1 | 1 | 1 | 1 | 1 | 1 | 0 | 1 | 1 | 0 | 0 | 0 | 0 |
| GO:0032774~RNA biosynthetic process | 10 | 1 | 1 | 1 | 1 | 1 | 1 | 1 | 1 | 0 | 1 | 1 | 0 | 0 | 0 | 0 |
| GO:0007155~cell adhesion | 10 | 1 | 1 | 1 | 1 | 1 | 1 | 1 | 1 | 0 | 1 | 0 | 0 | 0 | 0 | 1 |
| GO:0043412~biopolymer modification | 10 | 1 | 1 | 1 | 1 | 1 | 1 | 1 | 1 | 0 | 1 | 0 | 0 | 0 | 0 | 1 |
| GO:0022610~biological adhesion | 10 | 1 | 1 | 1 | 1 | 1 | 1 | 1 | 1 | 0 | 1 | 0 | 0 | 0 | 0 | 1 |
| GO:0051336~regulation of hydrolase activity | 10 | 1 | 1 | 1 | 1 | 1 | 1 | 1 | 1 | 0 | 1 | 0 | 0 | 0 | 0 | 1 |
| GO:0008152~metabolic process | 9 | 1 | 1 | 1 | 1 | 1 | 1 | 1 | 1 | 0 | 1 | 0 | 0 | 0 | 0 | 0 |
| GO:0006950~response to stress | 9 | 1 | 1 | 1 | 1 | 1 | 1 | 1 | 1 | 0 | 1 | 0 | 0 | 0 | 0 | 0 |
| GO:0007267~cell-cell signaling | 9 | 1 | 1 | 1 | 1 | 1 | 1 | 1 | 1 | 0 | 1 | 0 | 0 | 0 | 0 | 0 |
| GO:0009987~cellular process | 9 | 1 | 1 | 1 | 1 | 1 | 1 | 1 | 1 | 0 | 1 | 0 | 0 | 0 | 0 | 0 |
| GO:0007243~protein kinase cascade | 9 | 1 | 1 | 1 | 1 | 1 | 1 | 1 | 1 | 0 | 1 | 0 | 0 | 0 | 0 | 0 |
| GO:0065009~regulation of a molecular function | 9 | 1 | 1 | 1 | 1 | 1 | 1 | 1 | 1 | 0 | 1 | 0 | 0 | 0 | 0 | 0 |
| GO:0050801~ion homeostasis | 9 | 1 | 1 | 1 | 1 | 1 | 1 | 1 | 1 | 0 | 1 | 0 | 0 | 0 | 0 | 0 |
| GO:0007610~behavior | 9 | 1 | 1 | 1 | 1 | 1 | 1 | 1 | 1 | 0 | 1 | 0 | 0 | 0 | 0 | 0 |
| GO:0048771~tissue remodeling | 9 | 1 | 1 | 1 | 1 | 1 | 1 | 1 | 1 | 0 | 1 | 0 | 0 | 0 | 0 | 0 |
| GO:0042592~homeostatic process | 9 | 1 | 1 | 1 | 1 | 1 | 1 | 1 | 1 | 0 | 1 | 0 | 0 | 0 | 0 | 0 |
| GO:0016265~death | 9 | 1 | 1 | 1 | 1 | 1 | 1 | 1 | 1 | 0 | 1 | 0 | 0 | 0 | 0 | 0 |
| GO:0044260~cellular macromolecule metabolic process | 9 | 1 | 1 | 1 | 1 | 1 | 1 | 1 | 1 | 0 | 1 | 0 | 0 | 0 | 0 | 0 |
| GO:0006519~amino acid and derivative metabolic process | 9 | 1 | 1 | 1 | 1 | 1 | 1 | 1 | 1 | 0 | 1 | 0 | 0 | 0 | 0 | 0 |
| GO:0009605~response to external stimulus | 9 | 1 | 1 | 1 | 1 | 1 | 1 | 1 | 1 | 0 | 1 | 0 | 0 | 0 | 0 | 0 |
| GO:0019752~carboxylic acid metabolic process | 9 | 1 | 1 | 1 | 1 | 1 | 1 | 1 | 1 | 0 | 1 | 0 | 0 | 0 | 0 | 0 |
| GO:0007264~small GTPase mediated signal transduction | 9 | 1 | 1 | 1 | 1 | 1 | 1 | 1 | 1 | 0 | 1 | 0 | 0 | 0 | 0 | 0 |
| GO:0048878~chemical homeostasis | 9 | 1 | 1 | 1 | 1 | 1 | 1 | 1 | 1 | 0 | 1 | 0 | 0 | 0 | 0 | 0 |
| GO:0042493~response to drug | 9 | 1 | 1 | 1 | 1 | 1 | 1 | 1 | 1 | 0 | 1 | 0 | 0 | 0 | 0 | 0 |
| GO:0044267~cellular protein metabolic process | 9 | 1 | 1 | 1 | 1 | 1 | 1 | 1 | 1 | 0 | 1 | 0 | 0 | 0 | 0 | 0 |
| GO:0019226~transmission of nerve impulse | 9 | 1 | 1 | 1 | 1 | 1 | 1 | 1 | 1 | 0 | 1 | 0 | 0 | 0 | 0 | 0 |
| GO:0051301~cell division | 9 | 1 | 1 | 1 | 1 | 1 | 1 | 1 | 1 | 0 | 1 | 0 | 0 | 0 | 0 | 0 |
| GO:0006979~response to oxidative stress | 9 | 1 | 1 | 1 | 1 | 1 | 1 | 1 | 1 | 0 | 1 | 0 | 0 | 0 | 0 | 0 |
| GO:0009628~response to abiotic stimulus | 9 | 1 | 1 | 1 | 1 | 1 | 1 | 1 | 1 | 0 | 1 | 0 | 0 | 0 | 0 | 0 |
| GO:0016311~dephosphorylation | 9 | 1 | 1 | 1 | 1 | 1 | 1 | 1 | 1 | 0 | 1 | 0 | 0 | 0 | 0 | 0 |
| GO:0016044~membrane organization and biogenesis | 9 | 1 | 1 | 1 | 1 | 1 | 1 | 1 | 1 | 0 | 1 | 0 | 0 | 0 | 0 | 0 |
| GO:0009611~response to wounding | 9 | 1 | 1 | 1 | 1 | 1 | 1 | 1 | 1 | 0 | 1 | 0 | 0 | 0 | 0 | 0 |
| GO:0019538~protein metabolic process | 9 | 1 | 1 | 1 | 1 | 1 | 1 | 1 | 1 | 0 | 1 | 0 | 0 | 0 | 0 | 0 |
| GO:0040007~growth | 9 | 1 | 1 | 1 | 1 | 1 | 1 | 1 | 1 | 0 | 1 | 0 | 0 | 0 | 0 | 0 |
| GO:0006996~organelle organization and biogenesis | 9 | 1 | 1 | 1 | 1 | 1 | 1 | 1 | 1 | 0 | 1 | 0 | 0 | 0 | 0 | 0 |
| GO:0006807~nitrogen compound metabolic process | 9 | 1 | 1 | 1 | 1 | 1 | 1 | 1 | 1 | 0 | 1 | 0 | 0 | 0 | 0 | 0 |
| GO:0022607~cellular component assembly | 9 | 1 | 1 | 1 | 1 | 1 | 1 | 1 | 1 | 0 | 1 | 0 | 0 | 0 | 0 | 0 |
| GO:0046849~bone remodeling | 9 | 1 | 1 | 1 | 1 | 1 | 1 | 1 | 1 | 0 | 1 | 0 | 0 | 0 | 0 | 0 |
| GO:0006944~membrane fusion | 9 | 1 | 1 | 1 | 1 | 1 | 1 | 1 | 1 | 0 | 1 | 0 | 0 | 0 | 0 | 0 |
| GO:0007626~locomotory behavior | 9 | 1 | 1 | 1 | 1 | 1 | 1 | 1 | 1 | 0 | 1 | 0 | 0 | 0 | 0 | 0 |
| GO:0007167~enzyme linked receptor protein signaling pathway | 9 | 1 | 1 | 1 | 1 | 1 | 1 | 1 | 1 | 0 | 1 | 0 | 0 | 0 | 0 | 0 |
| GO:0002376~immune system process | 9 | 1 | 1 | 1 | 1 | 1 | 1 | 1 | 1 | 0 | 1 | 0 | 0 | 0 | 0 | 0 |
| GO:0006629~lipid metabolic process | 9 | 1 | 1 | 1 | 1 | 1 | 1 | 1 | 1 | 0 | 1 | 0 | 0 | 0 | 0 | 0 |
| GO:0001775~cell activation | 9 | 1 | 1 | 1 | 1 | 1 | 1 | 1 | 1 | 0 | 1 | 0 | 0 | 0 | 0 | 0 |
| GO:0050790~regulation of catalytic activity | 9 | 1 | 1 | 1 | 1 | 1 | 1 | 1 | 1 | 0 | 1 | 0 | 0 | 0 | 0 | 0 |
| GO:0006082~organic acid metabolic process | 9 | 1 | 1 | 1 | 1 | 1 | 1 | 1 | 1 | 0 | 1 | 0 | 0 | 0 | 0 | 0 |
| GO:0009308~amine metabolic process | 9 | 1 | 1 | 1 | 1 | 1 | 1 | 1 | 1 | 0 | 1 | 0 | 0 | 0 | 0 | 0 |
| GO:0009719~response to endogenous stimulus | 9 | 1 | 1 | 1 | 1 | 1 | 1 | 1 | 1 | 0 | 1 | 0 | 0 | 0 | 0 | 0 |
| GO:0055065~metal ion homeostasis | 9 | 1 | 1 | 1 | 1 | 1 | 1 | 1 | 1 | 0 | 1 | 0 | 0 | 0 | 0 | 0 |
| GO:0003013~circulatory system process | 9 | 1 | 1 | 1 | 1 | 1 | 1 | 1 | 1 | 0 | 1 | 0 | 0 | 0 | 0 | 0 |
| GO:0055074~calcium ion homeostasis | 9 | 1 | 1 | 1 | 1 | 1 | 1 | 1 | 1 | 0 | 1 | 0 | 0 | 0 | 0 | 0 |
| GO:0008015~blood circulation | 9 | 1 | 1 | 1 | 1 | 1 | 1 | 1 | 1 | 0 | 1 | 0 | 0 | 0 | 0 | 0 |
| GO:0006066~alcohol metabolic process | 9 | 1 | 1 | 1 | 1 | 1 | 1 | 1 | 1 | 0 | 1 | 0 | 0 | 0 | 0 | 0 |
| GO:0043085~positive regulation of catalytic activity | 9 | 1 | 1 | 1 | 1 | 1 | 1 | 1 | 1 | 0 | 1 | 0 | 0 | 0 | 0 | 0 |
| GO:0035295~tube development | 9 | 1 | 1 | 1 | 1 | 1 | 1 | 1 | 1 | 0 | 1 | 0 | 0 | 0 | 0 | 0 |
| GO:0007169~transmembrane receptor protein tyrosine kinase signaling pathway | 9 | 1 | 1 | 1 | 1 | 1 | 1 | 1 | 1 | 0 | 1 | 0 | 0 | 0 | 0 | 0 |
| GO:0019932~second-messenger-mediated signaling | 9 | 1 | 1 | 1 | 1 | 1 | 1 | 1 | 1 | 0 | 1 | 0 | 0 | 0 | 0 | 0 |
| GO:0007010~cytoskeleton organization and biogenesis | 8 | 1 | 1 | 1 | 1 | 1 | 0 | 1 | 1 | 0 | 1 | 0 | 0 | 0 | 0 | 0 |
| GO:0000003~reproduction | 8 | 1 | 1 | 1 | 1 | 1 | 1 | 0 | 1 | 0 | 1 | 0 | 0 | 0 | 0 | 0 |
| GO:0010324~membrane invagination | 8 | 1 | 1 | 1 | 1 | 1 | 0 | 1 | 1 | 0 | 1 | 0 | 0 | 0 | 0 | 0 |
| GO:0007178~transmembrane receptor protein serine/threonine kinase signaling pathway | 8 | 1 | 1 | 1 | 1 | 1 | 1 | 0 | 1 | 0 | 1 | 0 | 0 | 0 | 0 | 0 |
| GO:0000165~MAPKKK cascade | 8 | 0 | 1 | 1 | 1 | 1 | 1 | 1 | 1 | 0 | 1 | 0 | 0 | 0 | 0 | 0 |
| GO:0055080~cation homeostasis | 8 | 1 | 1 | 1 | 1 | 1 | 1 | 1 | 1 | 0 | 0 | 0 | 0 | 0 | 0 | 0 |
| GO:0045859~regulation of protein kinase activity | 8 | 0 | 1 | 1 | 1 | 1 | 1 | 1 | 1 | 0 | 1 | 0 | 0 | 0 | 0 | 0 |
| GO:0051704~multi-organism process | 8 | 1 | 1 | 1 | 1 | 1 | 0 | 1 | 1 | 0 | 1 | 0 | 0 | 0 | 0 | 0 |
| GO:0042157~lipoprotein metabolic process | 8 | 1 | 1 | 1 | 1 | 1 | 1 | 0 | 1 | 0 | 1 | 0 | 0 | 0 | 0 | 0 |
| GO:0055066~di-, tri-valent inorganic cation homeostasis | 8 | 1 | 1 | 1 | 1 | 1 | 1 | 1 | 1 | 0 | 0 | 0 | 0 | 0 | 0 | 0 |
| GO:0032501~multicellular organismal process | 8 | 1 | 1 | 1 | 1 | 1 | 0 | 1 | 1 | 0 | 1 | 0 | 0 | 0 | 0 | 0 |
| GO:0006790~sulfur metabolic process | 8 | 0 | 1 | 1 | 1 | 1 | 1 | 1 | 1 | 0 | 1 | 0 | 0 | 0 | 0 | 0 |
| GO:0005975~carbohydrate metabolic process | 8 | 1 | 1 | 1 | 1 | 1 | 0 | 1 | 1 | 0 | 1 | 0 | 0 | 0 | 0 | 0 |
| GO:0009056~catabolic process | 8 | 1 | 1 | 1 | 1 | 1 | 1 | 1 | 1 | 0 | 0 | 0 | 0 | 0 | 0 | 0 |
| GO:0009725~response to hormone stimulus | 8 | 1 | 1 | 1 | 1 | 1 | 1 | 0 | 1 | 0 | 1 | 0 | 0 | 0 | 0 | 0 |
| GO:0044248~cellular catabolic process | 8 | 1 | 1 | 1 | 1 | 1 | 1 | 1 | 1 | 0 | 0 | 0 | 0 | 0 | 0 | 0 |
| GO:0006259~DNA metabolic process | 7 | 1 | 1 | 1 | 1 | 1 | 0 | 1 | 1 | 0 | 0 | 0 | 0 | 0 | 0 | 0 |
| GO:0007249~I-kappaB kinase/NF-kappaB cascade | 7 | 1 | 1 | 1 | 0 | 1 | 1 | 1 | 0 | 0 | 1 | 0 | 0 | 0 | 0 | 0 |
| GO:0016310~phosphorylation | 7 | 0 | 1 | 1 | 1 | 1 | 1 | 0 | 1 | 0 | 1 | 0 | 0 | 0 | 0 | 0 |
| GO:0030029~actin filament-based process | 7 | 1 | 1 | 1 | 1 | 1 | 0 | 0 | 1 | 0 | 1 | 0 | 0 | 0 | 0 | 0 |
| GO:0045860~positive regulation of protein kinase activity | 7 | 0 | 1 | 1 | 1 | 0 | 1 | 1 | 1 | 0 | 1 | 0 | 0 | 0 | 0 | 0 |
| GO:0031279~regulation of cyclase activity | 7 | 1 | 1 | 1 | 1 | 1 | 0 | 1 | 1 | 0 | 0 | 0 | 0 | 0 | 0 | 0 |
| GO:0030036~actin cytoskeleton organization and biogenesis | 7 | 1 | 1 | 1 | 1 | 1 | 0 | 0 | 1 | 0 | 1 | 0 | 0 | 0 | 0 | 0 |
| GO:0051347~positive regulation of transferase activity | 7 | 0 | 1 | 1 | 1 | 0 | 1 | 1 | 1 | 0 | 1 | 0 | 0 | 0 | 0 | 0 |
| GO:0051338~regulation of transferase activity | 7 | 0 | 1 | 1 | 1 | 0 | 1 | 1 | 1 | 0 | 1 | 0 | 0 | 0 | 0 | 0 |
| GO:0051339~regulation of lyase activity | 7 | 1 | 1 | 1 | 1 | 1 | 0 | 1 | 1 | 0 | 0 | 0 | 0 | 0 | 0 | 0 |
| GO:0007005~mitochondrion organization and biogenesis | 7 | 1 | 1 | 1 | 1 | 1 | 0 | 1 | 1 | 0 | 0 | 0 | 0 | 0 | 0 | 0 |
| GO:0033674~positive regulation of kinase activity | 7 | 0 | 1 | 1 | 1 | 0 | 1 | 1 | 1 | 0 | 1 | 0 | 0 | 0 | 0 | 0 |
| GO:0043086~negative regulation of catalytic activity | 7 | 1 | 1 | 1 | 1 | 0 | 1 | 0 | 1 | 0 | 1 | 0 | 0 | 0 | 0 | 0 |
| GO:0009416~response to light stimulus | 7 | 1 | 1 | 1 | 1 | 1 | 1 | 0 | 1 | 0 | 0 | 0 | 0 | 0 | 0 | 0 |
| GO:0001666~response to hypoxia | 6 | 1 | 0 | 0 | 1 | 1 | 1 | 1 | 1 | 0 | 0 | 0 | 0 | 0 | 0 | 0 |
| GO:0019933~cAMP-mediated signaling | 6 | 1 | 1 | 1 | 1 | 0 | 1 | 0 | 1 | 0 | 0 | 0 | 0 | 0 | 0 | 0 |
| GO:0006732~coenzyme metabolic process | 6 | 1 | 1 | 1 | 0 | 1 | 0 | 1 | 0 | 0 | 1 | 0 | 0 | 0 | 0 | 0 |
| GO:0042060~wound healing | 6 | 1 | 1 | 1 | 0 | 1 | 1 | 1 | 0 | 0 | 0 | 0 | 0 | 0 | 0 | 0 |
| GO:0010033~response to organic substance | 6 | 1 | 0 | 0 | 1 | 1 | 1 | 0 | 1 | 0 | 1 | 0 | 0 | 0 | 0 | 0 |
| GO:0009582~detection of abiotic stimulus | 6 | 1 | 1 | 1 | 1 | 0 | 1 | 0 | 1 | 0 | 0 | 0 | 0 | 0 | 0 | 0 |
| GO:0009058~biosynthetic process | 6 | 1 | 1 | 1 | 0 | 1 | 0 | 1 | 0 | 0 | 1 | 0 | 0 | 0 | 0 | 0 |
| GO:0003015~heart process | 6 | 1 | 1 | 1 | 0 | 1 | 1 | 1 | 0 | 0 | 0 | 0 | 0 | 0 | 0 | 0 |
| GO:0043549~regulation of kinase activity | 6 | 0 | 1 | 1 | 1 | 0 | 1 | 0 | 1 | 0 | 1 | 0 | 0 | 0 | 0 | 0 |
| GO:0060047~heart contraction | 6 | 1 | 1 | 1 | 0 | 1 | 1 | 1 | 0 | 0 | 0 | 0 | 0 | 0 | 0 | 0 |
| GO:0019935~cyclic-nucleotide-mediated signaling | 6 | 0 | 1 | 1 | 1 | 1 | 1 | 0 | 1 | 0 | 0 | 0 | 0 | 0 | 0 | 0 |
| GO:0055086~nucleobase, nucleoside and nucleotide metabolic process | 6 | 1 | 1 | 1 | 1 | 0 | 0 | 1 | 1 | 0 | 0 | 0 | 0 | 0 | 0 | 0 |
| GO:0018193~peptidyl-amino acid modification | 6 | 0 | 0 | 0 | 1 | 1 | 1 | 1 | 1 | 0 | 1 | 0 | 0 | 0 | 0 | 0 |
| GO:0009581~detection of external stimulus | 6 | 1 | 1 | 1 | 1 | 0 | 1 | 0 | 1 | 0 | 0 | 0 | 0 | 0 | 0 | 0 |
| GO:0032787~monocarboxylic acid metabolic process | 5 | 1 | 1 | 1 | 0 | 0 | 1 | 0 | 0 | 0 | 1 | 0 | 0 | 0 | 0 | 0 |
| GO:0007179~transforming growth factor beta receptor signaling pathway | 5 | 1 | 1 | 1 | 1 | 0 | 0 | 0 | 1 | 0 | 0 | 0 | 0 | 0 | 0 | 0 |
| GO:0009100~glycoprotein metabolic process | 5 | 0 | 0 | 0 | 1 | 1 | 1 | 0 | 1 | 0 | 1 | 0 | 0 | 0 | 0 | 0 |
| GO:0043413~biopolymer glycosylation | 5 | 0 | 0 | 0 | 1 | 1 | 1 | 0 | 1 | 0 | 1 | 0 | 0 | 0 | 0 | 0 |
| GO:0048511~rhythmic process | 5 | 1 | 0 | 0 | 1 | 0 | 1 | 1 | 1 | 0 | 0 | 0 | 0 | 0 | 0 | 0 |
| GO:0006974~response to DNA damage stimulus | 5 | 1 | 0 | 0 | 1 | 1 | 0 | 1 | 1 | 0 | 0 | 0 | 0 | 0 | 0 | 0 |
| GO:0019953~sexual reproduction | 5 | 1 | 1 | 1 | 0 | 1 | 0 | 0 | 0 | 0 | 1 | 0 | 0 | 0 | 0 | 0 |
| GO:0007276~gamete generation | 5 | 1 | 1 | 1 | 0 | 1 | 0 | 0 | 0 | 0 | 1 | 0 | 0 | 0 | 0 | 0 |
| GO:0009314~response to radiation | 5 | 1 | 0 | 0 | 1 | 1 | 1 | 0 | 1 | 0 | 0 | 0 | 0 | 0 | 0 | 0 |
| GO:0043285~biopolymer catabolic process | 5 | 1 | 1 | 1 | 0 | 1 | 1 | 0 | 0 | 0 | 0 | 0 | 0 | 0 | 0 | 0 |
| GO:0007265~Ras protein signal transduction | 5 | 0 | 1 | 1 | 0 | 1 | 0 | 1 | 0 | 0 | 1 | 0 | 0 | 0 | 0 | 0 |
| GO:0009607~response to biotic stimulus | 5 | 1 | 0 | 0 | 1 | 1 | 0 | 0 | 1 | 0 | 1 | 0 | 0 | 0 | 0 | 0 |
| GO:0007611~learning and/or memory | 5 | 1 | 1 | 1 | 0 | 1 | 1 | 0 | 0 | 0 | 0 | 0 | 0 | 0 | 0 | 0 |
| GO:0051186~cofactor metabolic process | 5 | 1 | 1 | 1 | 0 | 1 | 0 | 1 | 0 | 0 | 0 | 0 | 0 | 0 | 0 | 0 |
| GO:0031589~cell-substrate adhesion | 5 | 0 | 1 | 1 | 1 | 1 | 0 | 0 | 1 | 0 | 0 | 0 | 0 | 0 | 0 | 0 |
| GO:0007015~actin filament organization | 4 | 0 | 1 | 1 | 1 | 0 | 0 | 0 | 1 | 0 | 0 | 0 | 0 | 0 | 0 | 0 |
| GO:0009266~response to temperature stimulus | 4 | 1 | 0 | 0 | 1 | 1 | 0 | 0 | 1 | 0 | 0 | 0 | 0 | 0 | 0 | 0 |
| GO:0032504~multicellular organism reproduction | 4 | 1 | 1 | 1 | 0 | 0 | 1 | 0 | 0 | 0 | 0 | 0 | 0 | 0 | 0 | 0 |
| GO:0006725~aromatic compound metabolic process | 4 | 0 | 0 | 0 | 1 | 1 | 1 | 0 | 1 | 0 | 0 | 0 | 0 | 0 | 0 | 0 |
| GO:0007601~visual perception | 4 | 1 | 1 | 1 | 0 | 0 | 0 | 1 | 0 | 0 | 0 | 0 | 0 | 0 | 0 | 0 |
| GO:0006512~ubiquitin cycle | 4 | 1 | 1 | 1 | 0 | 0 | 0 | 0 | 0 | 0 | 1 | 0 | 0 | 0 | 0 | 0 |
| GO:0010038~response to metal ion | 4 | 1 | 0 | 0 | 1 | 0 | 0 | 0 | 1 | 0 | 1 | 0 | 0 | 0 | 0 | 0 |
| GO:0010035~response to inorganic substance | 4 | 1 | 0 | 0 | 1 | 0 | 0 | 0 | 1 | 0 | 1 | 0 | 0 | 0 | 0 | 0 |
| GO:0006955~immune response | 4 | 1 | 1 | 1 | 0 | 1 | 0 | 0 | 0 | 0 | 0 | 0 | 0 | 0 | 0 | 0 |
| GO:0050953~sensory perception of light stimulus | 4 | 1 | 1 | 1 | 0 | 0 | 0 | 1 | 0 | 0 | 0 | 0 | 0 | 0 | 0 | 0 |
| GO:0009117~nucleotide metabolic process | 4 | 1 | 1 | 1 | 0 | 0 | 0 | 1 | 0 | 0 | 0 | 0 | 0 | 0 | 0 | 0 |
| GO:0016337~cell-cell adhesion | 4 | 1 | 0 | 0 | 1 | 0 | 0 | 0 | 1 | 0 | 1 | 0 | 0 | 0 | 0 | 0 |
| GO:0043406~positive regulation of MAP kinase activity | 4 | 0 | 0 | 0 | 1 | 0 | 1 | 0 | 1 | 0 | 1 | 0 | 0 | 0 | 0 | 0 |
| GO:0007283~spermatogenesis | 3 | 1 | 0 | 0 | 0 | 1 | 0 | 0 | 0 | 0 | 1 | 0 | 0 | 0 | 0 | 0 |
| GO:0048232~male gamete generation | 3 | 1 | 0 | 0 | 0 | 1 | 0 | 0 | 0 | 0 | 1 | 0 | 0 | 0 | 0 | 0 |
| GO:0006575~amino acid derivative metabolic process | 3 | 0 | 0 | 0 | 0 | 1 | 1 | 0 | 0 | 0 | 1 | 0 | 0 | 0 | 0 | 0 |
| GO:0006260~DNA replication | 3 | 1 | 1 | 1 | 0 | 0 | 0 | 0 | 0 | 0 | 0 | 0 | 0 | 0 | 0 | 0 |
| GO:0006091~generation of precursor metabolites and energy | 3 | 1 | 0 | 0 | 0 | 1 | 0 | 0 | 0 | 0 | 1 | 0 | 0 | 0 | 0 | 0 |
| GO:0042445~hormone metabolic process | 3 | 1 | 0 | 0 | 0 | 1 | 1 | 0 | 0 | 0 | 0 | 0 | 0 | 0 | 0 | 0 |
| GO:0044262~cellular carbohydrate metabolic process | 3 | 1 | 1 | 1 | 0 | 0 | 0 | 0 | 0 | 0 | 0 | 0 | 0 | 0 | 0 | 0 |
| GO:0048589~developmental growth | 3 | 0 | 1 | 1 | 0 | 1 | 0 | 0 | 0 | 0 | 0 | 0 | 0 | 0 | 0 | 0 |
| GO:0016051~carbohydrate biosynthetic process | 3 | 0 | 1 | 1 | 0 | 0 | 0 | 0 | 0 | 0 | 1 | 0 | 0 | 0 | 0 | 0 |
| GO:0048015~phosphoinositide-mediated signaling | 3 | 1 | 0 | 0 | 0 | 0 | 1 | 1 | 0 | 0 | 0 | 0 | 0 | 0 | 0 | 0 |
| GO:0040011~locomotion | 3 | 0 | 0 | 0 | 0 | 0 | 1 | 1 | 0 | 0 | 1 | 0 | 0 | 0 | 0 | 0 |
| GO:0007160~cell-matrix adhesion | 3 | 0 | 1 | 1 | 0 | 1 | 0 | 0 | 0 | 0 | 0 | 0 | 0 | 0 | 0 | 0 |
| GO:0006952~defense response | 3 | 1 | 1 | 1 | 0 | 0 | 0 | 0 | 0 | 0 | 0 | 0 | 0 | 0 | 0 | 0 |
| GO:0007218~neuropeptide signaling pathway | 2 | 0 | 0 | 0 | 0 | 1 | 0 | 0 | 0 | 0 | 1 | 0 | 0 | 0 | 0 | 0 |
| GO:0000226~microtubule cytoskeleton organization and biogenesis | 2 | 0 | 1 | 1 | 0 | 0 | 0 | 0 | 0 | 0 | 0 | 0 | 0 | 0 | 0 | 0 |
| GO:0043434~response to peptide hormone stimulus | 2 | 1 | 0 | 0 | 0 | 1 | 0 | 0 | 0 | 0 | 0 | 0 | 0 | 0 | 0 | 0 |
| GO:0006457~protein folding | 2 | 1 | 0 | 0 | 0 | 1 | 0 | 0 | 0 | 0 | 0 | 0 | 0 | 0 | 0 | 0 |
| GO:0043405~regulation of MAP kinase activity | 2 | 0 | 0 | 0 | 0 | 0 | 1 | 0 | 0 | 0 | 1 | 0 | 0 | 0 | 0 | 0 |
| GO:0016071~mRNA metabolic process | 2 | 1 | 0 | 0 | 0 | 0 | 1 | 0 | 0 | 0 | 0 | 0 | 0 | 0 | 0 | 0 |
| GO:0051789~response to protein stimulus | 2 | 0 | 0 | 0 | 0 | 1 | 0 | 0 | 0 | 0 | 1 | 0 | 0 | 0 | 0 | 0 |
| GO:0031098~stress-activated protein kinase signaling pathway | 2 | 0 | 1 | 1 | 0 | 0 | 0 | 0 | 0 | 0 | 0 | 0 | 0 | 0 | 0 | 0 |
| GO:0007605~sensory perception of sound | 2 | 0 | 1 | 1 | 0 | 0 | 0 | 0 | 0 | 0 | 0 | 0 | 0 | 0 | 0 | 0 |
| GO:0051348~negative regulation of transferase activity | 2 | 0 | 1 | 1 | 0 | 0 | 0 | 0 | 0 | 0 | 0 | 0 | 0 | 0 | 0 | 0 |
| GO:0043414~biopolymer methylation | 2 | 1 | 0 | 0 | 0 | 1 | 0 | 0 | 0 | 0 | 0 | 0 | 0 | 0 | 0 | 0 |
| GO:0050817~coagulation | 2 | 1 | 0 | 0 | 0 | 0 | 1 | 0 | 0 | 0 | 0 | 0 | 0 | 0 | 0 | 0 |
| GO:0032147~activation of protein kinase activity | 2 | 0 | 0 | 0 | 1 | 0 | 0 | 0 | 1 | 0 | 0 | 0 | 0 | 0 | 0 | 0 |
| GO:0050954~sensory perception of mechanical stimulus | 2 | 0 | 1 | 1 | 0 | 0 | 0 | 0 | 0 | 0 | 0 | 0 | 0 | 0 | 0 | 0 |
| GO:0051345~positive regulation of hydrolase activity | 2 | 1 | 0 | 0 | 0 | 1 | 0 | 0 | 0 | 0 | 0 | 0 | 0 | 0 | 0 | 0 |
| GO:0019221~cytokine and chemokine mediated signaling pathway | 2 | 0 | 0 | 0 | 0 | 1 | 0 | 0 | 0 | 0 | 1 | 0 | 0 | 0 | 0 | 0 |
| GO:0006936~muscle contraction | 1 | 0 | 0 | 0 | 0 | 1 | 0 | 0 | 0 | 0 | 0 | 0 | 0 | 0 | 0 | 0 |
| GO:0032535~regulation of cellular component size | 1 | 0 | 0 | 0 | 0 | 0 | 0 | 0 | 0 | 0 | 1 | 0 | 0 | 0 | 0 | 0 |
| GO:0048736~appendage development | 1 | 0 | 0 | 0 | 0 | 1 | 0 | 0 | 0 | 0 | 0 | 0 | 0 | 0 | 0 | 0 |
| GO:0016053~organic acid biosynthetic process | 1 | 0 | 0 | 0 | 0 | 0 | 0 | 1 | 0 | 0 | 0 | 0 | 0 | 0 | 0 | 0 |
| GO:0003012~muscle system process | 1 | 0 | 0 | 0 | 0 | 1 | 0 | 0 | 0 | 0 | 0 | 0 | 0 | 0 | 0 | 0 |
| GO:0046394~carboxylic acid biosynthetic process | 1 | 0 | 0 | 0 | 0 | 0 | 0 | 1 | 0 | 0 | 0 | 0 | 0 | 0 | 0 | 0 |
| GO:0051258~protein polymerization | 1 | 0 | 0 | 0 | 0 | 0 | 0 | 0 | 0 | 0 | 1 | 0 | 0 | 0 | 0 | 0 |
| GO:0048762~mesenchymal cell differentiation | 1 | 0 | 0 | 0 | 0 | 0 | 0 | 0 | 0 | 0 | 1 | 0 | 0 | 0 | 0 | 0 |
| GO:0009991~response to extracellular stimulus | 1 | 1 | 0 | 0 | 0 | 0 | 0 | 0 | 0 | 0 | 0 | 0 | 0 | 0 | 0 | 0 |
| GO:0007631~feeding behavior | 1 | 1 | 0 | 0 | 0 | 0 | 0 | 0 | 0 | 0 | 0 | 0 | 0 | 0 | 0 | 0 |
| GO:0031667~response to nutrient levels | 1 | 1 | 0 | 0 | 0 | 0 | 0 | 0 | 0 | 0 | 0 | 0 | 0 | 0 | 0 | 0 |
| GO:0060173~limb development | 1 | 0 | 0 | 0 | 0 | 1 | 0 | 0 | 0 | 0 | 0 | 0 | 0 | 0 | 0 | 0 |
| GO:0003018~vascular process in circulatory system | 1 | 0 | 0 | 0 | 0 | 1 | 0 | 0 | 0 | 0 | 0 | 0 | 0 | 0 | 0 | 0 |
| GO:0008213~protein amino acid alkylation | 1 | 1 | 0 | 0 | 0 | 0 | 0 | 0 | 0 | 0 | 0 | 0 | 0 | 0 | 0 | 0 |
| GO:0018958~phenol metabolic process | 1 | 0 | 0 | 0 | 0 | 0 | 1 | 0 | 0 | 0 | 0 | 0 | 0 | 0 | 0 | 0 |
| GO:0016055~Wnt receptor signaling pathway | 1 | 0 | 0 | 0 | 0 | 1 | 0 | 0 | 0 | 0 | 0 | 0 | 0 | 0 | 0 | 0 |
| GO:0000302~response to reactive oxygen species | 1 | 1 | 0 | 0 | 0 | 0 | 0 | 0 | 0 | 0 | 0 | 0 | 0 | 0 | 0 | 0 |
| GO:0032943~mononuclear cell proliferation | 1 | 0 | 0 | 0 | 0 | 0 | 1 | 0 | 0 | 0 | 0 | 0 | 0 | 0 | 0 | 0 |
| GO:0030198~extracellular matrix organization and biogenesis | 1 | 0 | 0 | 0 | 0 | 0 | 1 | 0 | 0 | 0 | 0 | 0 | 0 | 0 | 0 | 0 |
| GO:0030534~adult behavior | 1 | 0 | 0 | 0 | 0 | 1 | 0 | 0 | 0 | 0 | 0 | 0 | 0 | 0 | 0 | 0 |
| GO:0009259~ribonucleotide metabolic process | 1 | 0 | 1 | 0 | 0 | 0 | 0 | 0 | 0 | 0 | 0 | 0 | 0 | 0 | 0 | 0 |
| GO:0007154~cell communication | 1 | 0 | 0 | 0 | 0 | 1 | 0 | 0 | 0 | 0 | 0 | 0 | 0 | 0 | 0 | 0 |
| GO:0007259~JAK-STAT cascade | 1 | 0 | 0 | 0 | 0 | 0 | 1 | 0 | 0 | 0 | 0 | 0 | 0 | 0 | 0 | 0 |
| GO:0018212~peptidyl-tyrosine modification | 1 | 0 | 0 | 0 | 0 | 0 | 0 | 1 | 0 | 0 | 0 | 0 | 0 | 0 | 0 | 0 |
| GO:0006766~vitamin metabolic process | 1 | 0 | 0 | 0 | 0 | 0 | 1 | 0 | 0 | 0 | 0 | 0 | 0 | 0 | 0 | 0 |
| GO:0043062~extracellular structure organization and biogenesis | 1 | 0 | 0 | 0 | 0 | 0 | 1 | 0 | 0 | 0 | 0 | 0 | 0 | 0 | 0 | 0 |
| GO:0030522~intracellular receptor-mediated signaling pathway | 1 | 0 | 0 | 0 | 0 | 0 | 1 | 0 | 0 | 0 | 0 | 0 | 0 | 0 | 0 | 0 |
| GO:0007266~Rho protein signal transduction | 1 | 0 | 0 | 0 | 0 | 0 | 0 | 0 | 0 | 0 | 1 | 0 | 0 | 0 | 0 | 0 |
| GO:0042446~hormone biosynthetic process | 1 | 0 | 0 | 0 | 0 | 1 | 0 | 0 | 0 | 0 | 0 | 0 | 0 | 0 | 0 | 0 |
| GO:0007156~homophilic cell adhesion | 1 | 0 | 0 | 0 | 0 | 0 | 0 | 0 | 0 | 0 | 1 | 0 | 0 | 0 | 0 | 0 |
| GO:0030518~steroid hormone receptor signaling pathway | 1 | 1 | 0 | 0 | 0 | 0 | 0 | 0 | 0 | 0 | 0 | 0 | 0 | 0 | 0 | 0 |
| miRNA targeting different GOBP | | 162 | 157 | 156 | 142 | 160 | 142 | 129 | 142 | 9 | 141 | 23 | 1 | 5 | 13 | 27 |

| **Supplemntal Table 7.** | |
| --- | --- |
| Name | Sequence |
| 5S | GTCTACGGCCATACCACCCTGAAC |
| Drosha Forward | AGAGGCAATCAAGCCCTGTC |
| Drosha Reverse | GACTGTTGGCCTGTCCGTTA |
| Dicer1 Forward | GGAGAGTTACCCCAAACCCG |
| Dicer1 Reverse | TGTCCTCAGGAGGGTAGAGC |
| DGCR Forward | CTTGCTGCGCATGTATGGTC |
| DGCR Reverse | TCAGGATGTGAAGGTTGGGC |
| Keap1 Forward | CAGGAAGGGCTGGAACCG |
| Keap1 Reverse | GTGTCTCTGTTGGTGCCACG |
| Nrf2 Forward | GACTTGGAATTGCCACCGC |
| Nrf2 Reverse | GCCTTCTCCTGTTCCTTCTGG |
| rno-miR-141 | CTTCTAACACTGTCTGGTAAAGATGG |
| rno-miR-130a | TCCCAGTGCAATGTTAAAAGGGCAT |
| rno-miR-130b | TCCAGTGCAATGATGAAAGGGCAT |
| rno-miR-129 | TTCCTTTTTGCGGTCTGGGCTTGC |
| Novel-m0037-5p | GCGTCAGTGCACTACAGAACTTTGT |
| Novel-m0002-3p | GCGCTACCCTGTAGATCCGAATTTGT |
| Novel-m0040-3p | CGCGTAAACATCCTTGACTGGAAGCT |
| Novel-m0125-3p | GTAAACATCCCCGACTGGAAGCT |
| Novel-m0120-5p | GCGCAGCACCATCTGAAATCGGTTA |
| Novel-m0028-5p | CCGCTCGTACCGTGAGTAATAATGC |
| Novel-m0072-5p | GCGTAGCACCATTTGAAATCGGTT |
| Novel-m0041-3p | ACCCGTCCCGTTCGTCCCCG |

**Supplemntal Table 8**

| miRNAs | Genes | Foward sequence (5’–3’) | Reverse sequence (5’–3’) | Reference |
| --- | --- | --- | --- | --- |
| miR-129 | Smoc2 | AAACTGTTCATGGTCCCCAG | AGCGTTGATGTGAGCTGGTC | [1](#_ENREF_1) |
|  | Dcn | ACGCATGAGACAACCATGAA | TCGAAGCTCCTGGAGTGTTT | [2](#_ENREF_2) |
| miRNA-130a/miR-130b | Igfbp3 | AGCCGTCTCCTGGAAACACC | CCCGCTTTCTGCCTTTGG | [3](#_ENREF_3) |
|  | Sepp1 | GACAGTGGTTGCTCTTCTTCAA | TCGCAGGTCTTCCAATCTG | [4](#_ENREF_4) |
|  | Col1a2 | ATGGTGTTGATGGTCCCG | AATACTGAGCAGTTCC | [5](#_ENREF_5) |
|  | Edem1 | ACTGATTCCAAACAGCCCTT | GGATCCCTGTCTTGGTGTTT | [6](#_ENREF_6) |
| miR-218b | Emp1 | GATGTTGGTGCTACTGGCCG | CCCAGATGCCCACGAGCTTA | [7](#_ENREF_7) |
|  | Rapgef5 | GCCTTTGTTAGGCACCGTTC | CTGCCCCATTCATCGTCCTC | This article |
| miRNA-141 | Igfbp3 | AGCCGTCTCCTGGAAACACC | CCCGCTTTCTGCCTTTGG | [3](#_ENREF_3) |
|  | Sepp1 | GACAGTGGTTGCTCTTCTTCAA | TCGCAGGTCTTCCAATCTG | [4](#_ENREF_4) |
| miR-3588 | Edem1 | ACTGATTCCAAACAGCCCTT | GGATCCCTGTCTTGGTGTTT | [6](#_ENREF_6) |
|  | Dpt | CGGTATAGCAAGAGGTGCCC | TGATATGACACCCTCAGGGAC | This article |

Reference

1 Zhang, M., Pritchard, M. R., Middleton, F. A., Horton, J. A. & Damron, T. A. Microarray analysis of perichondral and reserve growth plate zones identifies differential gene expressions and signal pathways. *Bone*. **43**, 511-520 (2008).

2 He, F. *et al.* Upregulation of decorin by FXR in vascular smooth muscle cells. *Biochem Biophys Res Commun*. **372**, 746-751 (2008).

3 Zhou, Z. Y. *et al.* Increased expression of insulin-like growth factor-binding protein-3 is implicated in erectile dysfunction in two-kidney one-clip hypertensive rats after propranolol treatment. *Asian journal of andrology*. **13**, 851-855 (2011).

4 Steinbrenner, H. *et al.* Localization and regulation of pancreatic selenoprotein P. *J Mol Endocrinol*. **50**, 31-42 (2013).

5 Yan, X. *et al.* Fluoride induces apoptosis and alters collagen I expression in rat osteoblasts. *Toxicol Lett*. **200**, 133-138 (2011).

6 Tiberio, L. *et al.* Mechanisms of interleukin-6 protection against ischemia-reperfusion injury in rat liver. *Cytokine*. **34**, 131-142 (2006).

7 Lee, H.-S. *et al.* EMP-1 is a junctional protein in a liver stem cell line and in the liver. *Biochem Bioph Res Co*. **334**, 996-1003 (2005).
